# Lewis acid-mediated transformations of 5-acyl-*N*-fluoroalkyl-1,2,3-triazoles to cyclopentenones, indenones, or oxazoles†

**DOI:** 10.1039/d4ra01707b

**Published:** 2024-04-25

**Authors:** Lukáš Janecký, Petr Beier

**Affiliations:** a The Institute of Organic Chemistry and Biochemistry of the Czech Academy of Sciences Flemingovo nam. 2 16610 Prague 6 Czech Republic beier@uochb.cas.cz; b Department of Organic Chemistry, Faculty of Science, Charles University Hlavova 2030/8 128 43 Prague 2 Czech Republic

## Abstract

We present a transition metal-free approach to 2-*N*-substituted indenones, cyclopentenones, and 4-carbonyl oxazoles, based on the reaction of 5-acylated *N*-fluoroalkyl substituted 1,2,3-triazoles (prepared by a three-component click reaction of copper acetylides, fluoroalkyl azides, and acyl chlorides) with Lewis acids aluminium trichloride or boron trifluoride etherate, proceeding *via* the generation and cyclization of vinyl cations.

## Abstract 2

2-*N*-Substituted indenones, cyclopentenones or 4-carbonyl oxazoles are prepared from 5-acylated *N*-fluoroalkyl substituted 1,2,3-triazoles and Lewis acids.

## Graphical abstract

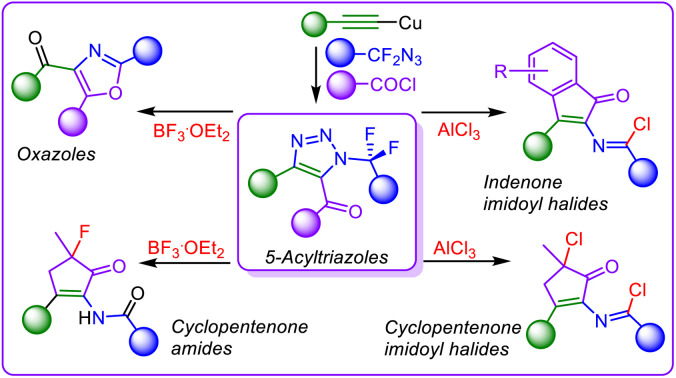

## Introduction

**Fig. 1:**
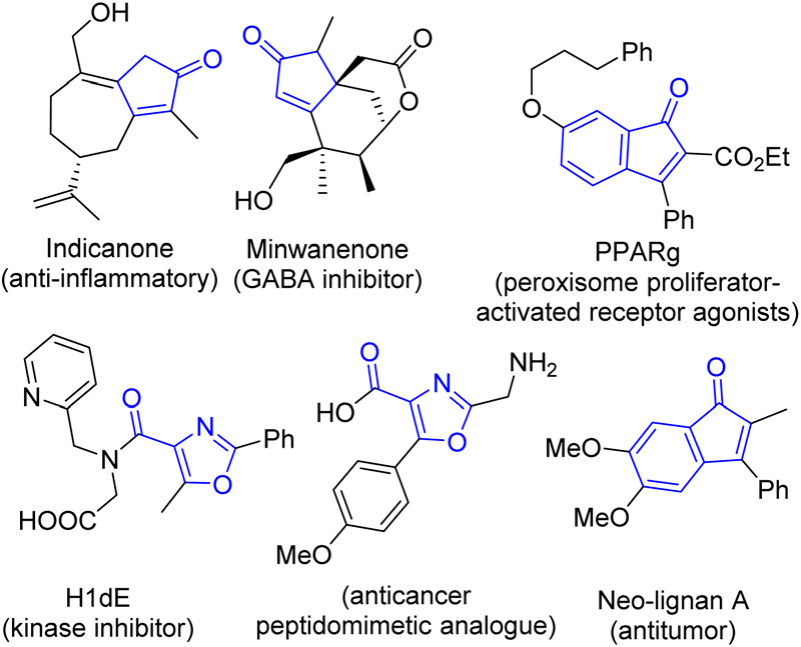
Selected examples of bioactive 4-carbonyl oxazoles, cyclopentenones and indenones.

Multi-substituted cyclopentenones, indenones and 4-carbonyl oxazoles constitute important classes of biologically active compounds known as anti-inflammatory/anticancer agents or enzyme inhibitors (Fig. 1).^1–6^ Synthetic strategies for obtaining their *N*-alkenyl derivatives (2-*N*-substituted cyclopentenones and indenones) or fully substituted oxazoles are limited because of the low availability of the starting materials and the necessity to use transition metal complexes or harsh reaction conditions. There is no general synthetic approach leading to these structures and each type of product requires a specific methodology. Despite the availability of numerous synthetic methods for the preparation of mono- or di-substituted oxazoles,^7^ access to tri-substituted 4-carbonyl oxazoles is not well explored and relies mainly on the intramolecular Cu-catalyzed cyclization of (thio)enamides^8^ or bromo(thio)enones,^9^ or on a protocol starting from 2-azido enones^10^ or alkynyl ketones.^11^

2-*N*-substituted indenones can be accessed from ynamides,^12^ 2-alkynylbenzoyl cyanides,^13^ or 2-hydroxy-substituted internal alkynes.^14^ 2-Amino indenones were also prepared *via* Au-catalyzed intermolecular oxidation of 2-carbonyl-1-ethynyl benzenes^15^ or by co-catalyzed annulation of thioamides with ynamides.^16^ Approaches leading to 2-amino-substituted cyclopentenones are limited to methods starting from previously modified cyclopentenone or cyclopentane rings.^17–19^ Other procedures, starting from ynamides,^20^ α-aminoenals^21^ or vinyl ketenes,^22^ are highly substrate-specific and do not allow for the further modification of the amino position in cyclopentenones.

**Scheme 1:**
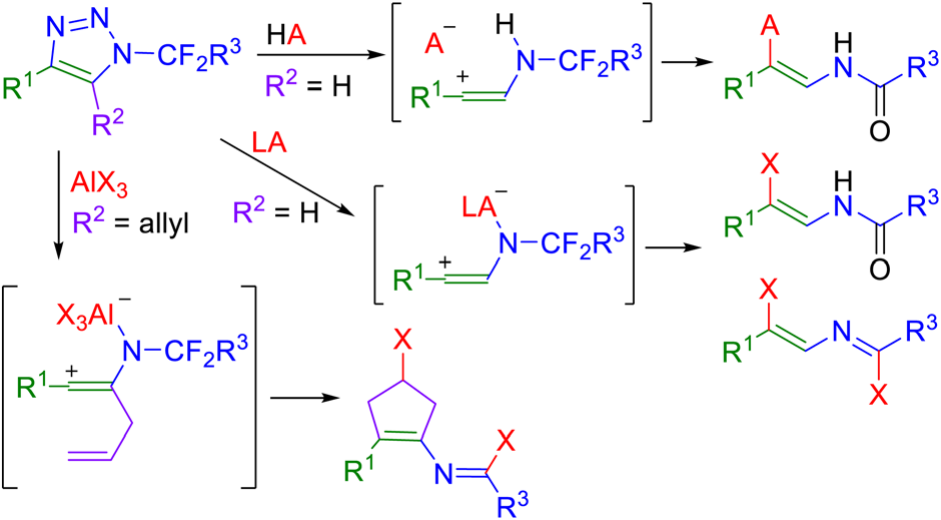
Brønsted and Lewis acid-mediated denitrogenation of *N*-fluoroalkyl-1,2,3-triazoles (HA = TfOH or FSO_3_H, LA = BF_3_^.^OEt_2_ or AlX_3_).

We recently reported Brønsted and Lewis acid-mediated reactions of *N*-fluoroalkylated 1,2,3-triazoles,^23–25^ as an efficient approach to the generation of vinyl cation intermediates which then reacted with various nucleophiles providing *N*-alkenyl compounds such as β-enamido triflates,^26^ β-fluoro enamides^27^ and β-halo alkenyl imidoyl halides,^28^ or undergo cyclization reactions to form multisubstituted cyclopentenes (Scheme 1).^29^

**Scheme 2:**
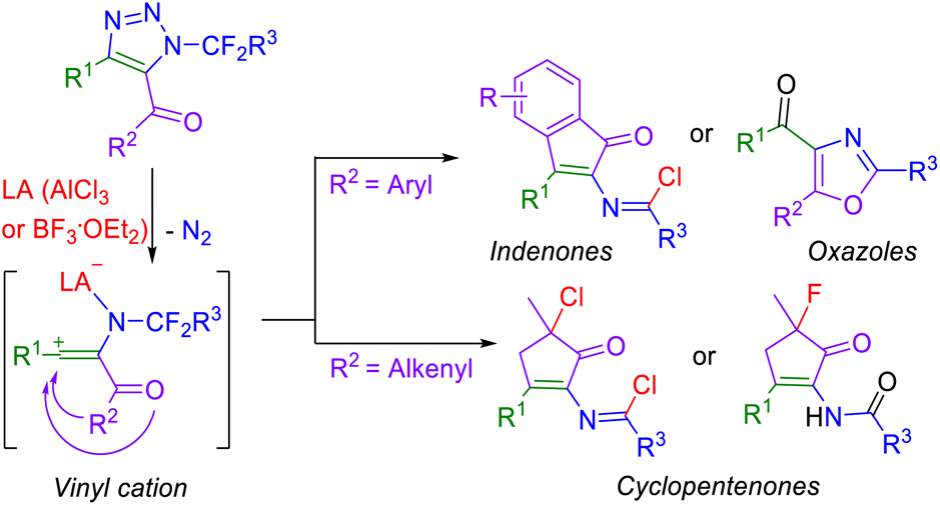
Proposed Lewis acid-mediated transformation of 5-acyl-1,2,3-triazoles into new cyclic products.

Herein, we propose a new synthetic methodology to 2-*N*-substituted cyclopentenones, indenones and 4-carbonyl oxazoles from 5-acyl-*N*-fluoroalkyl-1,2,3-triazoles by a treatment with a Lewis acid (AlCl_3_ or BF_3_·OEt_2_), proceeding *via* vinyl cations and their cyclization onto alkenes or arenes (Scheme 2). This type of cyclization is very rare.^30^

## Results and discussion

Synthesis of 5-acetyl- and 5-benzoyl-substituted 1,2,3-triazoles by an intercepted click reaction was briefly described in the literature.^31^ For our study it was necessary to prepare a library of 1-(per)fluoroalkyl-4-substituted-5-acyl-1,2,3-triazoles. However, application of Wu's conditions^31^ (phenylacetylene, CuI, Et_3_N and benzoyl chloride or acetyl chloride) with our fluorinated azides^23,32,33^ (CF_3_N_3_ or C_2_F_5_N_3_) did not afford the desired 5-acylated triazoles. Therefore, we turned to the use of (phenylethynyl)copper (1a), which was shown to be reactive with azido(per)fluoroalkanes in intercepted click iodination or allylation.^28^ A three-component reaction of 1a, azidopentafluoroethane (2a) and methacryloyl chloride was used for the optimization of the synthesis of triazole 3a (Table 1). Initially, a mixture of 3a and triazole side-product 3a-H formed (entry 1). Increasing the amount of acyl chloride and using anhydrous conditions improved the yield of 3a and suppressed the formation of 3a-H (entries 2–4). To further increase the yield of 3a, screening for an additional base was conducted (entries 5–11), identifying DIPEA as the most efficient one (entry 12). Finally, the use of 2 equiv. of acyl chloride and 3 equiv. of DIPEA provided 3a in optimized yield (entry 14).

**Table 1:** Optimization of reaction conditions leading to triazole 3a^a^

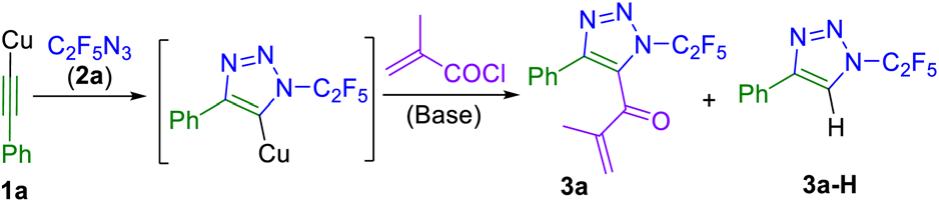				
Entry	RCOCl (equiv.)	Base (equiv.)	Ratio 3a/3a-H^b^	Yield 3a (%)^c^
1^d^	1	—	75 : 25	n.d.
2	1.2	—	99 : 1	51
3	2	—	100 : 0	53
4	5	—	100 : 0	33
5	1.2	Pyridine (1)	61 : 39	n.d.
6	1.2	NaOMe (2)	22 : 78	n.d.
7	1.2	Et_3_N (2)	80 : 20	n.d.
8	1.2	NaNH_2_ (3)	38 : 62	n.d.
9	1.2	KF (1)	100 : 0	45
10	1.2	DBU (2)	0 : 100	0
11	1.2	*i*-Pr_2_NH (2)	22 : 78	n.d.
12	1.2	DIPEA (2)	100 : 0	63
13	2	DIPEA (2)	100 : 0	75
14	2	DIPEA (3)	100 : 0	80
15	2	DIPEA (5)	100 : 0	46

a Reaction conditions: 1a (1 mmol), 2a (1.5 equiv.), THF (4 mL), 0–25 °C, then RCOCl, base, 3 Å MS (240 mg), rt, 18 h.

b ^19^F NMR ratio.

c Isolated yield.

d Without 3 Å molecular sieves. n.d. not determined.

**Scheme 3:**
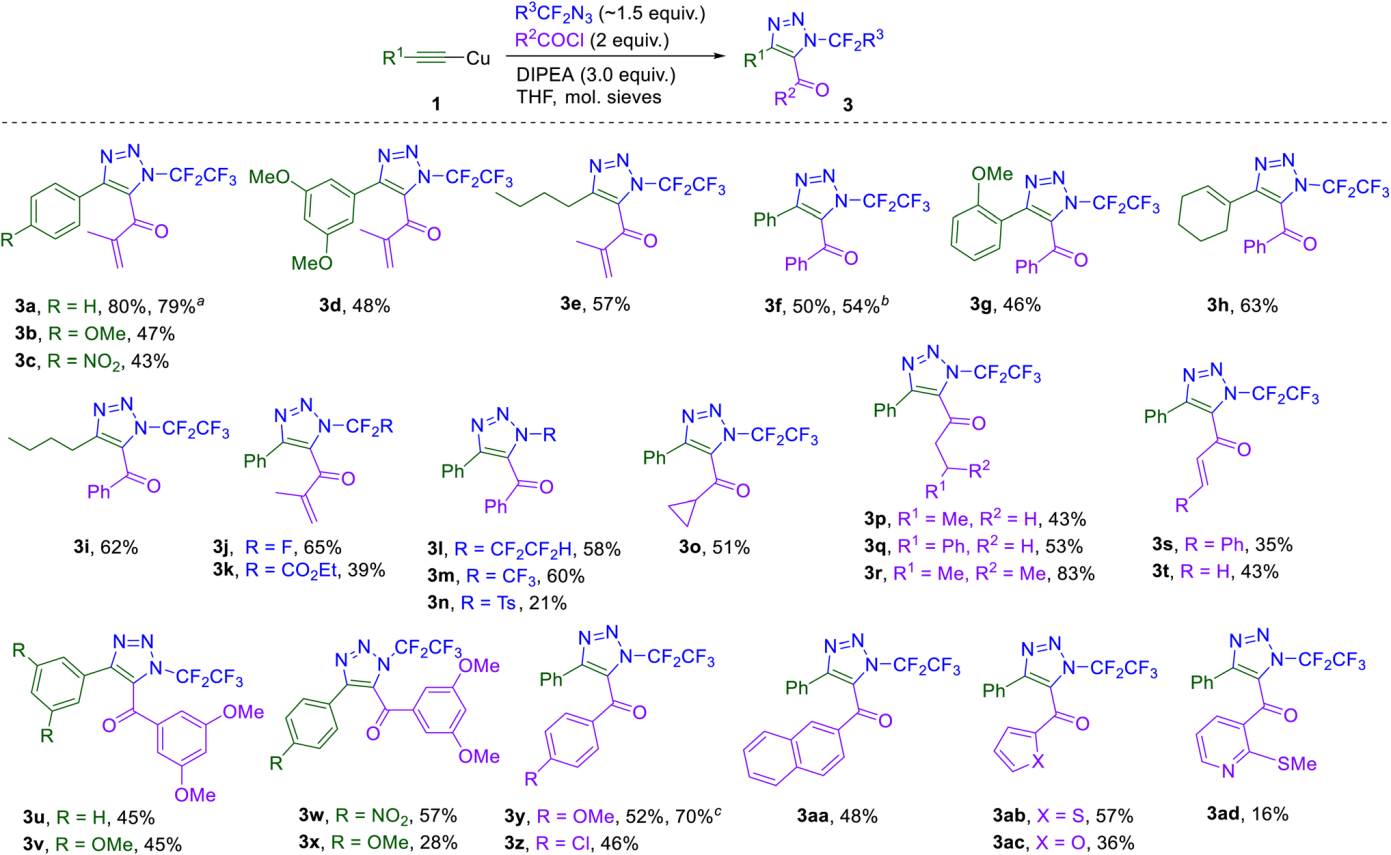
Substrate scope of the intercepted click reaction leading to 5-acyl triazoles 3. Reaction conditions: 1 (1.0 mmol), 2 (1.5 mmol), DIPEA (3.0 mmol), acyl halide (2.0 mmol), THF (4 mL), 3 Å MS (240 mg), rt, 18 h. ^*a*^10.7 mmol scale. ^*b*^4.16 mmol scale. ^*c*^5.0 mmol scale.

Having established the optimal reaction conditions for the intercepted click reaction and acylation sequence, the scope of the protocol was investigated on diverse acetylenic substrates, fluorinated azides, and acid chlorides (Scheme 3). 5-Methacryloyl triazoles (3b–e) with strongly electron-poor or electron-rich aryl rings in position 4 were prepared in moderate yields. To demonstrate the scalability of reaction, triazole 3a was prepared on 1.76 g (10.7 mmol) scale in high yield. 5-Benzoyl-substituted triazoles (3f–i) were also prepared in good yields. Varying the azide reagent revealed that highly fluorinated azidoalkanes afforded better product yields than tosyl azide or ethyl azidodifluoroacetate. Modification of triazoles in position 4 with aryl, alkyl and alkenyl groups and modification in position 5 with aryl, heteroaryl, alkyl, cycloalkyl and alkenyl groups afforded products mostly in satisfactory yields. Scale-up of 3f and 3y to 4–5 mmol was also successful.

**Scheme 4:**
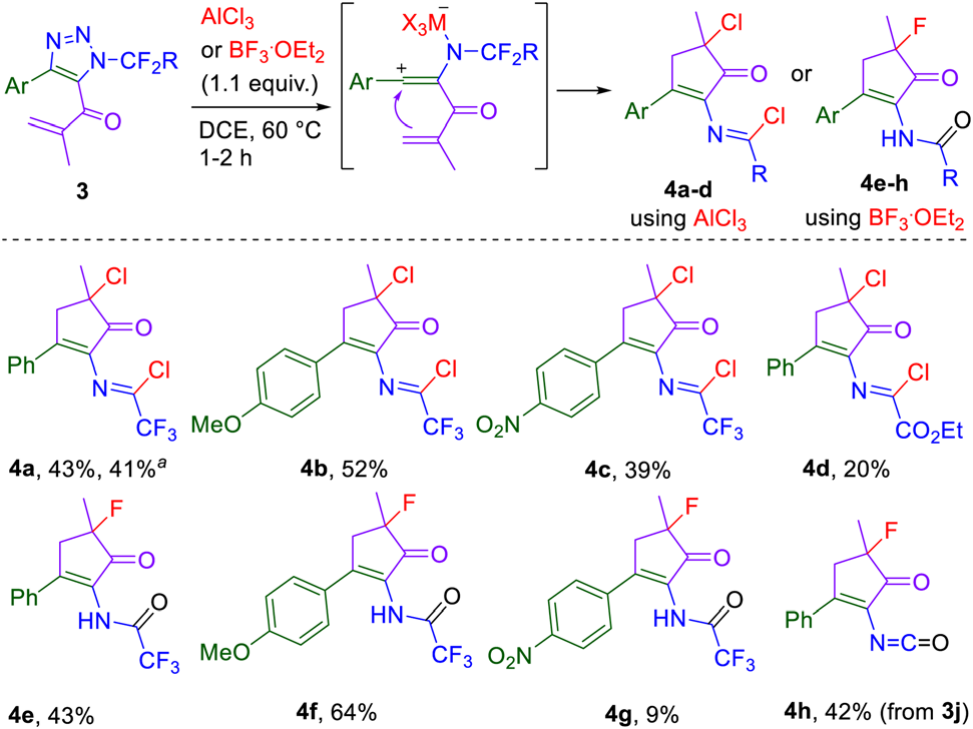
AlCl_3_-mediated denitrogenation/cyclization of triazoles 3 leading to cyclopentenone imidoyl chlorides 4a–d and BF_3_·OEt_2_-mediated denitrogenation/cyclization of triazoles 3 leading to cyclopentenone amides 4e–g and cyclopentenone isocyanate 4h. Reaction conditions: 3 (1.0 mmol), AlCl_3_ or BF_3_^.^OEt_2_ (1.1 mmol), DCE (0.1M), 60 °C, 1–2 h. ^*a*^2.2 mmol scale.

Next, AlCl_3_ was chosen as a suitably strong and easily available Lewis acid to investigate the denitrogenative transformation of 5-acryloyl substituted triazoles. Cyclization of the formed vinyl cation intermediate onto the alkene moiety and chloride capture of the resulting carbocation led to the formation of cyclopentenone imidoyl chlorides (4a–d) in moderate yields (Scheme 4). The cation-stabilizing *p*-methoxyphenyl group in position 4 of triazole 3 improved the product yields. This observation, together with the necessity of triazole denitrogenation in the initial step of the reaction, speaks against an alternative reaction mechanism involving Nazarov cyclization, typically starting from a divinyl ketone. BF_3_·OEt_2_ was used for the preparation of fluorinated cyclopentenone amides 4e–g from triazoles 3 (Scheme 4). In the case of *N*-CF_3_ triazole 3j, the final product is not a cyclopentenone amide but cyclopentenone isocyanate 4h due to the facile HF elimination of the NHC(O)F intermediate.

Triazole 3r was a special case, as the vinyl cation intermediate induced a 1,5-hydride shift to form a tertiary carbocation and an α,β-unsaturated ketone. Cyclization and proton elimination afforded cyclopentenone 4i (Scheme S1 in the ESI†).

**Scheme 5:**
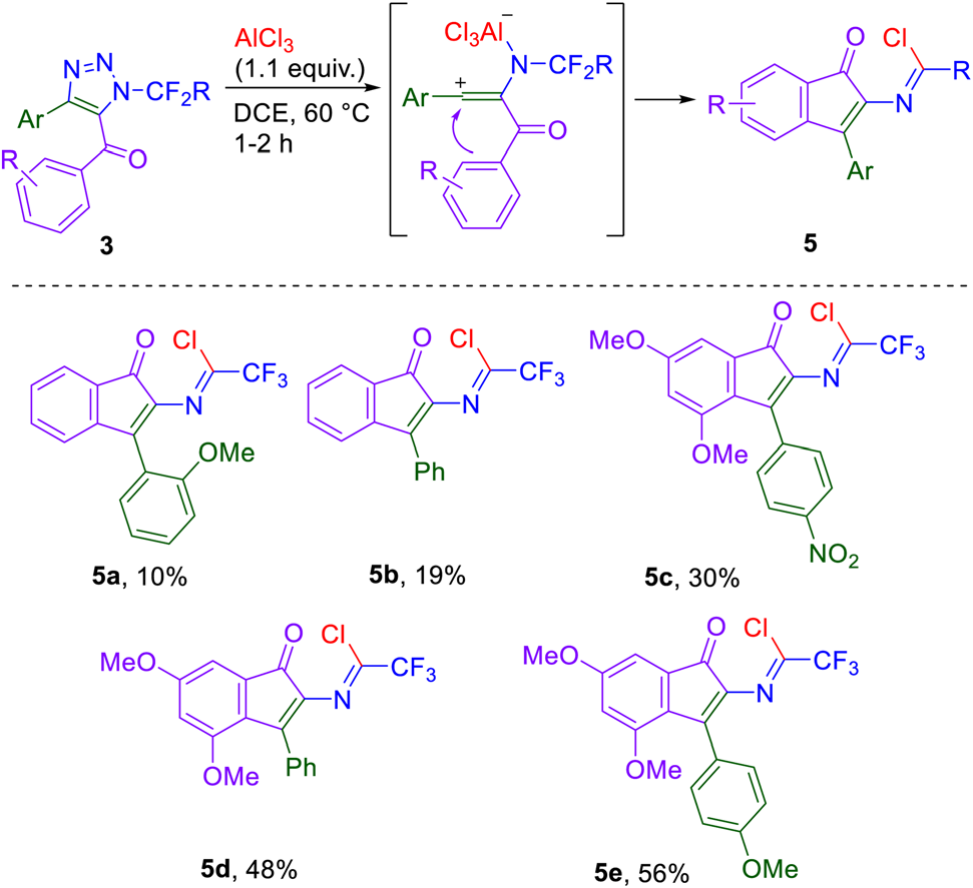
AlCl_3_-mediated denitrogenation/cyclization of triazoles 3 leading to indenone imidoyl chlorides 5. Reaction conditions: 3 (1.0 mmol), AlCl_3_ (1.1 mmol), DCE (0.1M), 60 °C, 1–2 h.

Subjecting 5-benzoyl triazoles 3 to a reaction with AlCl_3_ led to the cyclization on the aryl ring of the benzoyl moiety, forming indenone imidoyl chlorides 5 (Scheme 5). Again, the presence of vinyl cation-stabilizing groups in position 4 of the starting triazole and electron-rich groups on the aryl ring of the substituted benzoyl moiety in position 5 of the triazole both increased product yields. Using 5-benzoyl triazoles instead of dimethoxybenzoyl triazoles afforded only low to moderate indenone yields.

**Scheme 6:**
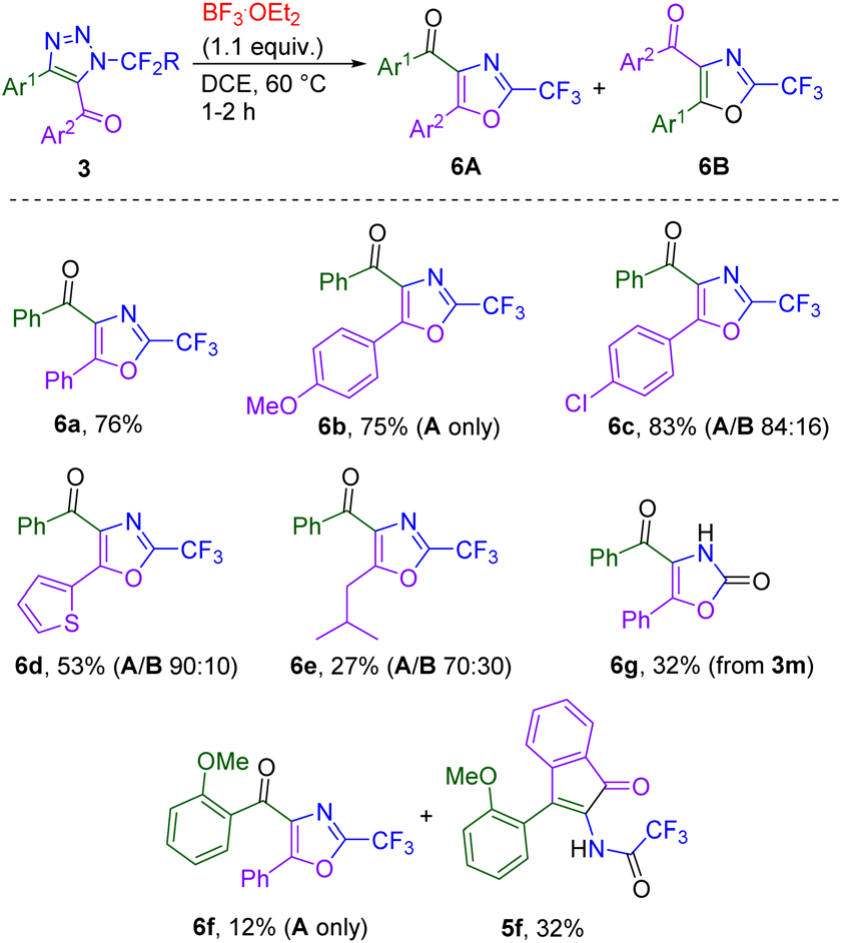
BF_3_·OEt_2_-mediated denitrogenation/cyclization of triazoles 3 leading to 4-acyl oxazoles 6. Reaction conditions: 3 (1.0 mmol), BF_3_·OEt_2_ (1.1 mmol), DCE (0.1M), 60 °C, 1–2 h.

The formation of 4-acyl oxazoles 6a–g was observed upon boron trifluoride-mediated transformation of 5-benzoyl triazoles (Scheme 6). Two isomers of products 6 can be formed: electron-rich or -neutral aryl groups in position 5 of the starting triazole cyclized selectively to form isomer A and triazoles with deactivated aryl groups in position 5 gave a mixture of isomers A and B with good to high selectivity for isomer A. Heating the mixture of isomers in a microwave did not lead to their interconversion, ruling out the possibility of Cornforth rearrangement in 2-trifluoromethyloxazoles. Oxazole 6f was accompanied by indenone 5f side-product. Oxazolone 6g was prepared selectively from 3m, as 2-fluorooxazoles are hydrolytically unstable.

**Scheme 7:**
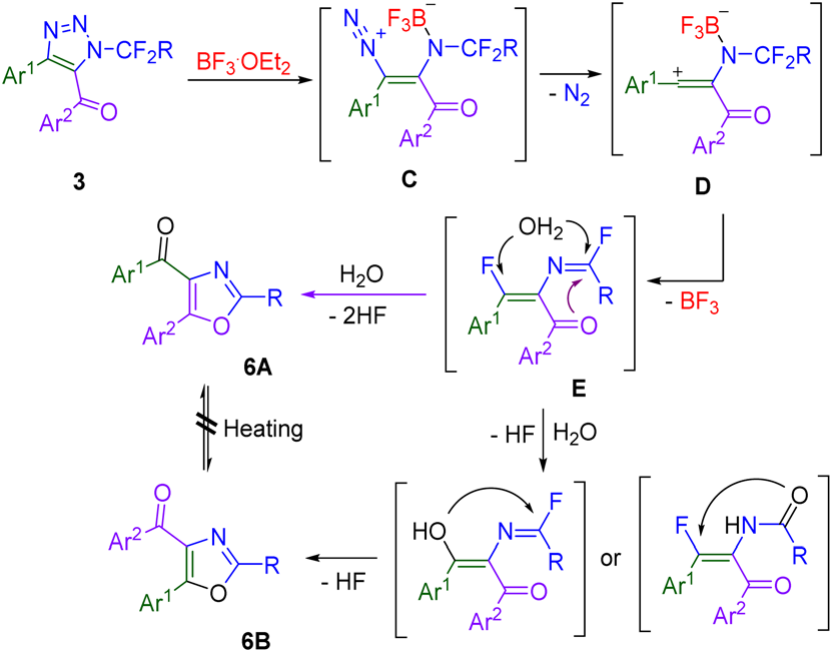
Mechanism of BF_3_-mediated transformation of triazoles 3 to oxazoles 6.

To rationalize the formation of products 6A and 6B, we considered the following reaction mechanism (Scheme 7). As demonstrated in our earlier report,^27^ the coordination of BF_3_ to nitrogen atoms of the triazole ring (particularly the coordination to N1) led to opening of the triazole ring and the elimination of the nitrogen molecule from the diazonium intermediate C. The resulting vinyl cation D underwent elimination of tetrafluoroborate and fluoride transfer to form imidoyl fluoride E. Cyclization of the acyl oxygen led to 6A. Alternatively, hydrolysis of the vinyl fluoride moiety of E created a competitive acyl moiety (oxygen nucleophile) for cyclization to 6B. The required one molecule of water probably comes from the moisture in the solvent. Deliberate addition of a small amount of water reduced product yields, presumably because of competitive hydrolysis of the used Lewis acid.

**Scheme 8:**
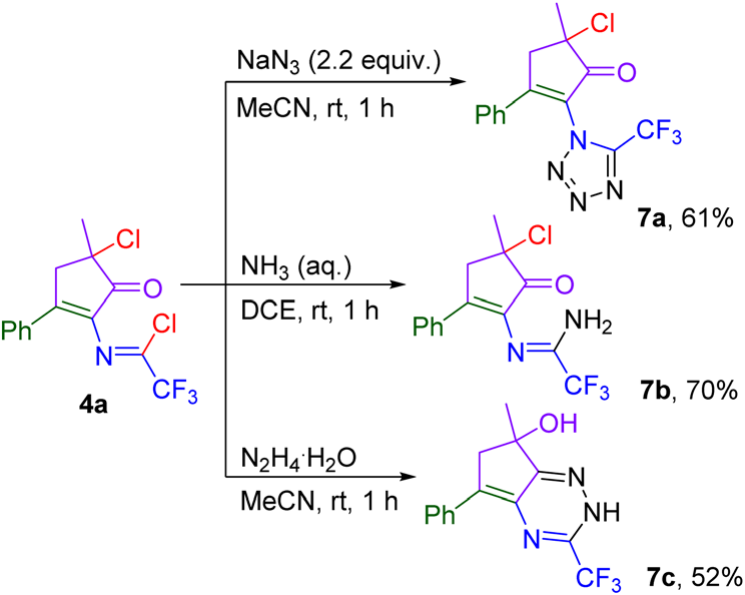
Synthetic utilization of cyclopentenone 4a.

**Scheme 9:**
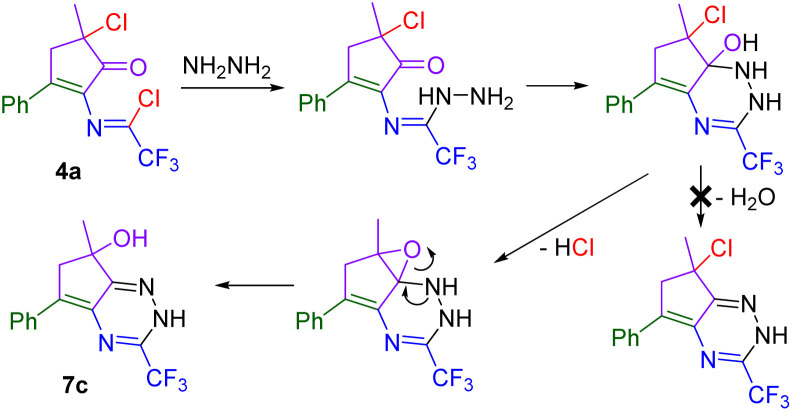
Proposed mechanism of the formation of 7c.

The synthetic utility of cyclopentenone imidoyl chlorides was demonstrated on examples of post-functionalization of 4a (Scheme 8). Trifluoromethylated tetrazole 7a was easily prepared from 4a using sodium azide. The addition of an aqueous ammonia solution afforded amidine 7b, and the addition of aqueous hydrazine led to the cyclization of the carbonyl and imidoyl chloride functional groups to give trifluoromethylated triazine 7c. We proposed that 7c is formed by cyclization of hydrazine nitrogen to the six-membered ring, followed by the hydroxyl shift and substitution of the chlorine atom for oxygen on the cyclopentene moiety (Scheme 9).

## Conclusions

In conclusion, we present the synthesis of *N*-electron-acceptor group-substituted 5-acyl-1,2,3-triazoles by an intercepted click reaction, namely a three-component cyclization of azide, copper acetylide and acyl chloride. Lewis acid-mediated triazole ring opening and nitrogen molecule elimination provided key reactive intermediates – vinyl cations, which cyclized selectively to form either cyclopentenone imidoyl chlorides, indenone imidoyl chlorides, cyclopentenone amides, or 2-trifluoromethyl oxazoles, depending on the combination of the Lewis acid used and the substitution in position 5 of the triazole ring. Post-functionalization of cyclopentenone imidoyl chloride gave access to selectively functionalized *N*-alkenyl compounds (amidines) or new nitrogen heterocycles (triazine or tetrazole). The presented methodology demonstrates a Lewis acid-mediated generation of vinyl cations from triazoles and their synthetic utilization in the formation of new C–C bonds.

## Author contributions

PB supervised the project. LJ contributed to experiments and product characterization. LJ and PB jointly conceived the project, prepared the manuscript, and contributed to discussions.

## Conflicts of interest

There are no conflicts to declare.

## Supplementary Material

RA-014-D4RA01707B-s001
