# Supplementary material for: Lewis acid-mediated transformations of 5-acyl-N-fluoroalkyl-1,2,3-triazoles to cyclopentenones, indenones, or oxazoles
Source: RSC Adv. 2024 Apr 25;14(19):13640–5. doi: 10.1039/d4ra01707b (PMC11043917; doi:10.1039/d4ra01707b)

Electronic Supplementary Material (ESI) for RSC Advances.
This journal is © The Royal Society of Chemistry 2024

Lewis Acid-Mediated Transformations of 5-Acyl-*N*-Fluoroalkyl-1,2,3-Triazoles to Cyclopentenones, Indenones, or Oxazoles

Lukáš Janecký[a,b], Petr Beier[a,*]

[a] Institute of Organic Chemistry and Biochemistry, Academy of Sciences of the Czech Republic, Flemingovo nám. 2, 166 10 Prague, Czechia

[b] Department of Organic Chemistry, Faculty of Science, Charles University, Hlavova 2030/8, 128 43 Prague 2, Czechia

*Corresponding author: beier@uochb.cas.cz

Supporting information

Contents

General information 2
Synthesis of triazoles **3** 3
General procedure: 3
Preparation and characterization of starting triazoles **3** 3
Synthesis of cyclopentenones **4** 12
General procedure A: 12
General procedure B: 12
Preparation and characterization of cyclopentenone derivatives **4** 12
Mechanistic evidence of the vinyl cation intermediate 15
Synthesis of indenones imidoyl halides **5** 15
General procedure: 15

Preparation and characterization of indenones imidoyl halides **5** 16
Synthesis of oxazoles **6** 17
General procedure: 17
Preparation and characterization of oxazoles **6** 17
Synthesis of *N*-alkenyl compounds **7** 20
Copies of NMR spectra 22

General information

All commercially available chemicals were used as received unless stated otherwise. Flash column chromatography was performed using silica gel 60 (0.040–0.063 mm). Automated flash column chromatography was performed on Teledyne ISCO CombiFlash Rf$^+$ Lumen Automated Flash Chromatography System with UV/Vis detection. ^{1}H, ^{13}C, and ^{19}F NMR spectra were measured at ambient temperature using 5 mm diameter NMR tubes. ^{13}C NMR spectra were proton decoupled. The chemical shift values (δ) are reported in ppm relative to internal Me_4Si (0 ppm for ^{1}H and ^{13}C NMR) or residual solvents and internal $CFCl_3$ (0 ppm for ^{19}F NMR). Coupling constants (J) are reported in Hertz. Structural elucidation was aided by additional acquisition of various 2D spectra (^{1}H-1H COSY, ^{1}H-^{13}C HSQC, ^{1}H-^{13}C HMBC). High resolution mass spectra (HRMS) were recorded on a Waters Micromass AutoSpec Ultima or Agilent 7890A GC coupled with Waters GCT Premier orthogonal acceleration time-of-flight detector using electron impact (EI) or chemical ionization (CI), on an LTQ Orbitrap XL using electrospray ionization (ESI), Q-Tof micro (Waters) is a quadrupole orthogonal acceleration time-of-flight tandem mass spectrometer using atmospheric-pressure chemical ionization (APCI), and on a Bruker solariX 94 ESI/MALDI-FT-ICR using dual ESI/MALDI ionization.

Synthesis of triazoles **3**

General procedure:

Under air atmosphere, a 10 mL screw-cap glass reaction tube was charged with the corresponding copper(I) acetylide (1.0 mmol, 1.0 equiv.), 3Å molecular sieves (240 mg) and a stirring bar. Then it was cooled to 0 °C, followed by the addition of a solution of azide in THF (~1.5 mmol, 4 mL), EDIPA (523 µl, 3.0 mmol, 3.0 equiv.) and the corresponding acyl chloride (2.0 mmol, 2.0 equiv.). The reaction mixture was left to warm to room temperature and stirred for 16-20 h. The crude suspension was filtered via a short plug of silica gel and washed with THF (3 x 10 mL). The filtrate was evaporated with Celite (water bath 40 °C) and purified by column chromatography (silica gel, cyclohexane/EtOAc or pentane/toluene) to obtain pure triazole **3**.

Preparation and characterization of starting triazoles **3**

*2-methyl-1-(1-(perfluoroethyl)-4-phenyl-1H-1,2,3-triazol-5-yl)prop-2-en-1-one (**3a**):*

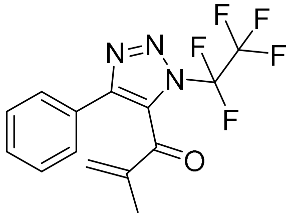

Prepared according to the general procedure. Yield: 266.0 mg, 80%. Scale up reaction: Under air atmosphere, 250 mL round bottom flask was charged with the corresponding copper(I) acetylide (10.67 mmol, 1.756 g, 1.0 equiv.), 3Å molecular sieves (2.56 g) and a stirring bar. Then it was cooled to 0 °C, followed by the addition of a solution of $CF_3CF_2N_3$ in THF (~16 mmol, 45 mL), EDIPA (5.6 ml, 32.0 mmol, 3.0 equiv.) and methacryloyl chloride (2.1 ml, 21.33 mmol, 2.0 equiv.). The reaction mixture was left to warm to room temperature and stirred for 18 h. The crude suspension was filtered via a short plug of silica gel and washed with THF (3 x 50 mL). The filtrate was evaporated with Celite (water bath 40 °C) and purified by flash column chromatography (Eluent: 10:1 cyclohexane/EtOAc) to obtain pure **3a**. Yield: 79%, 2.796 g, pale yellow oil. ^{1}H NMR (400 MHz, $CDCl_3$) δ 7.63 – 7.60 (m, 2H), 7.44 – 7.40 (m, 3H), 6.06 (q, *J* = 1.5 Hz, 1H), 5.60 (q, *J* = 0.9 Hz, 1H), 2.05 (dd, *J* = 1.5, 0.9 Hz, 3H); ^{13}C NMR (101 MHz, $CDCl_3$) δ 187.7, 146.5, 144.5, 133.0, 130.4 (t, *J* = 1.8 Hz), 129.8, 129.2, 128.0, 127.6, 117.0 (qt, *J* = 287.9, 40.0 Hz), 110.9 (tq, *J* = 272.0, 43.7 Hz), 16.6; ^{19}F NMR (376 MHz, $CDCl_3$) δ –82.7 (s, 3F), –94.9 (s, 2F); HRMS (ESI$^+$) *m/z* calcd for $C_{14}H_{11}F_5N_3O$ [M+H]$^+$: 332.08168, found 332.08156.

*1-(4-(4-methoxyphenyl)-1-(perfluoroethyl)-1H-1,2,3-triazol-5-yl)-2-methylprop-2-en-1-one (**3b**):*

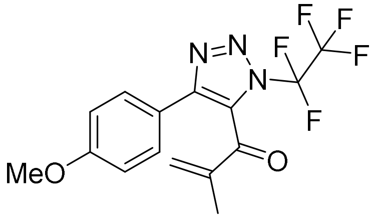

Prepared according to the general procedure. Yield: 187.0 mg, 47%, pale yellow oil; Eluent: 10:1 cyclohexane/EtOAc. ^{1}H NMR (401 MHz, $CDCl_3$) δ 7.56 – 7.52 (m, 2H), 6.96 – 6.92 (m, 2H), 6.06 (q, *J* = 1.5 Hz, 1H), 5.60 (q, *J* = 0.9 Hz, 1H), 3.83 (s, 3H), 2.04 (dd, *J* = 1.5, 0.9 Hz, 3H); ^{13}C NMR (101 MHz, $CDCl_3$) δ 188.0, 160.8, 146.5, 144.5, 132.8, 129.4 (t, *J* = 1.8 Hz), 129.0, 120.4, 117.1 (qt, *J* = 288.3, 40.2 Hz), 114.6, 110.9 (tq, *J* = 272.0, 43.6 Hz), 55.5, 16.6; ^{19}F NMR

(377 MHz, $CDCl_3$) δ –82.7 (s, 3F), –94.9 s (2F); HRMS (ESI$^+$) *m/z* calcd for $C_{15}H_{13}F_5N_3O_2$ $[M+H]^+$: 362.09224, found 362.09200.

*2-methyl-1-(4-(4-nitrophenyl)-1-(perfluoroethyl)-1H-1,2,3-triazol-5-yl)prop-2-en-1-one (**3c**):*

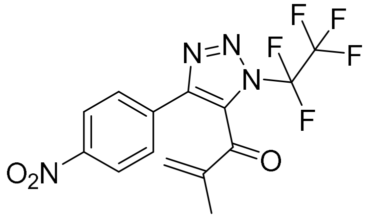

Prepared according to the general procedure. Yield: 161.0 mg, 43%, pale yellow oil; Eluent: 10:1 cyclohexane/EtOAc. ^{1}H NMR (400 MHz, $CDCl_3$) δ 8.29 – 8.25 (m, 2H), 7.83 – 7.79 (m, 2H), 6.16 (q, *J* = 1.5 Hz, 1H), 5.62 (q, *J* = 1.0 Hz, 1H), 2.08 (dd, *J* = 1.6, 0.9 Hz, 3H); ^{13}C NMR (101 MHz, $CDCl_3$) δ 187.1, 148.4, 144.7, 144.1, 134.0, 133.7, 131.9 (t, *J* = 1.7 Hz), 128.2, 124.4, 116.9 (qt, *J* = 287.9, 39.8 Hz), 110.9 (tq, *J* = 273.1, 43.8 Hz), 16.5; ^{19}F NMR (376 MHz, $CDCl_3$) δ –82.7 (s, 3F), –95.0 (s, 2F); HRMS (ESI$^+$) *m/z* calcd for $C_{14}H_9F_5N_4O_3$ $[M+Na]^+$: 399.04870, found 399.04898.

*1-(4-(3,5-dimethoxyphenyl)-1-(perfluoroethyl)-1H-1,2,3-triazol-5-yl)-2-methylprop-2-en-1-one (**3d**):*

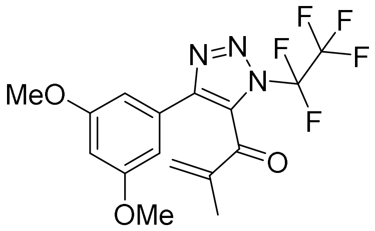

Prepared according to the general procedure. Yield: 188.0 mg, 48%, pale yellow oil; Eluent: 10:1 cyclohexane/EtOAc. ^{1}H NMR (401 MHz, $CDCl_3$) δ 6.77 (d, *J* = 2.3 Hz, 2H), 6.51 (t, *J* = 2.3 Hz, 1H), 6.11 (q, *J* = 1.5 Hz, 1H), 5.63 (q, *J* = 1.0 Hz, 1H), 3.80 (s, 6H), 2.07 (s, 3H); ^{13}C NMR (101 MHz, $CDCl_3$) δ 187.7, 161.3, 146.4, 144.5, 133.0, 130.5 (t, *J* = 1.8 Hz), 129.6, 117.0 (qt, *J* = 287.9, 40.0 Hz), 110.9 (tq, *J* = 272.0, 43.6 Hz), 105.5, 102.0, 55.5, 16.5; ^{19}F NMR (377 MHz, $CDCl_3$) δ –82.7 (s, 3F), –95.0 (s, 2F); HRMS (ESI$^+$) *m/z* calcd for $C_{16}H_{15}F_5N_3O_3$ $[M+H]^+$: 392.10281, found 392.10311.

*1-(4-butyl-1-(perfluoroethyl)-1H-1,2,3-triazol-5-yl)-2-methylprop-2-en-1-one (**3e**):*

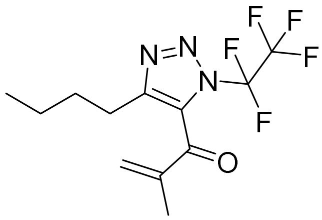

Prepared according to the general procedure. Yield: 176.5 mg, 57%, pale yellow oil; ^{1}H NMR (400 MHz, $CDCl_3$) δ 6.20 (q, *J* = 1.6 Hz, 1H), 5.65 – 5.64 (m, 1H), 2.67 – 2.62 (m, 2H), 2.06 – 2.04 (m, 3H), 1.68 – 1.60 (m, 2H), 1.37 – 1.27 (m, 2H), 0.92 – 0.87 (m, 3H); ^{13}C NMR (101 MHz, $CDCl_3$) δ 187.1, 148.2, 145.2, 132.3, 131.4 (t, *J* = 1.8 Hz), 117.1 (qt, *J* = 287.9, 40.0 Hz), 110.8 (tq, *J* = 271.3, 43.3 Hz), 31.0, 24.8, 22.4, 16.6, 13.8; ^{19}F NMR (376 MHz, $CDCl_3$) δ –82.7 (s, 3F), –94.9 (s, 2F); HRMS (ESI$^+$) *m/z* calcd for $C_{12}H_{15}F_5N_3O$ $[M+H]^+$: 312.11298, found 312.11277.

*(1-(perfluoroethyl)-4-phenyl-1H-1,2,3-triazol-5-yl)(phenyl)methanone (**3f**):*

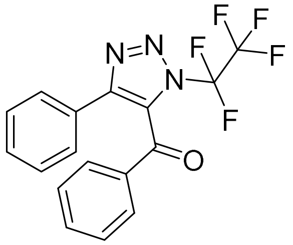

Prepared according to the general procedure. Yield: 183.0 mg, 50%. Scale up reaction: Under air atmosphere, a 100 mL screw-cap glass reaction tube was charged with the corresponding copper(**I**) acetylide (0.685 g, 4.16 mmol, 1.0 equiv.), 3Å molecular sieves (1.0 g) and a stirring bar. Then it was cooled to 0 °C, followed by the addition of a solution of $CF_3CF_2N_3$ in THF (~6.24 mmol, 18 mL), EDIPA (523 µl, 3.0 mmol, 3.0 equiv.) and benzoyl chloride (967 µl, 8.32 mmol, 2.0 equiv.). The reaction mixture was left to warm to room temperature and stirred for 18 h. The crude suspension was filtered via short plug of silica gel and washed with THF (3 x 20 mL). The filtrate was evaporated with Celite and purified by flash column chromatography (silica gel, eluent: 96:4 cyclohexane/EtOAc) to obtain pure **3f**. Yield: 54%, 831 mg, pale yellow oil; ^{1}H NMR (401 MHz, $CDCl_3$) δ 7.78 – 7.74 (m, 2H), 7.65 – 7.60 (m, 3H), 7.47 – 7.42 (m, 2H), 7.34 – 7.29 (m, 3H); ^{13}C NMR (101 MHz, $CDCl_3$) δ 185.8, 146.9, 135.6, 135.2, 130.6 (t, J = 2.0 Hz), 129.9, 129.7, 129.4, 129.1, 127.8, 127.7, 117.3 (qt, J = 288.3, 39.8 Hz), 111.0 (tq, J = 271.7, 43.5 Hz); ^{19}F NMR (471 MHz, $CDCl_3$) δ –82.6 (s, 3F), –94.5 (s, 2F); HRMS (ESI$^+$) *m/z* calcd for $C_{17}H_{11}F_5N_3O$ [M+H]$^+$: 368.08168, found 368.08163.

*(4-(2-methoxyphenyl)-1-(perfluoroethyl)-1H-1,2,3-triazol-5-yl)(phenyl)methanone (**3g**):*

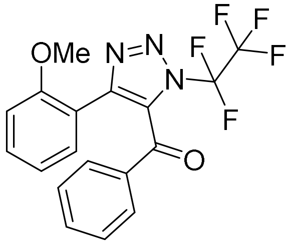

Prepared according to the general procedure. Yield: 160.0 mg, 46%, colorless oil; eluent: 95:5 cyclohexane/EtOAc. ^{1}H NMR (401 MHz, $CDCl_3$) δ 8.01 (dd, J = 7.7, 1.8 Hz, 1H), 7.74 – 7.70 (m, 2H), 7.56 – 7.51 (m, 1H), 7.40 – 7.35 (m, 2H), 7.29 (ddd, J = 8.2, 7.4, 1.8 Hz, 1H), 7.06 (td, J = 7.6, 1.1 Hz, 1H), 6.64 (dd, J = 8.3, 1.1 Hz, 1H), 3.27 (s, 3H); ^{13}C NMR (101 MHz, $CDCl_3$) δ 184.3, 155.3, 143.8, 135.7, 134.4, 131.7 (t, J = 2.2 Hz), 131.3, 130.2, 129.2, 128.8, 121.3, 117.4, 117.2 (qt, J = 287.9, 39.4 Hz), 111.1 (tq, J = 271.9, 43.0 Hz), 110.6, 53.5; ^{19}F NMR (377 MHz, $CDCl_3$) δ –82.0 (s, 3F), –93.4 (s, 2F); HRMS (ESI$^+$) *m/z* calcd for $C_{18}H_{13}F_5N_3O_2$ [M+H]$^+$: 398.09224, found 398.09217.

*(4-(cyclohex-1-en-1-yl)-1-(perfluoroethyl)-1H-1,2,3-triazol-5-yl)(phenyl)methanone (**3h**):*

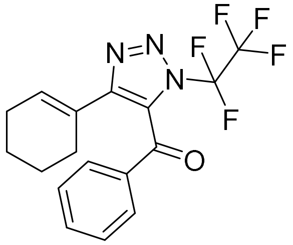

Prepared according to the general procedure. Yield: 234.9 mg, 63%, pale yellow oil; eluent: 10:1 cyclohexane/EtOAc. ^{1}H NMR (400 MHz, $CDCl_3$) δ 7.77 – 7.73 (m, 2H), 7.70 – 7.65 (m, 1H), 7.53 – 7.48 (m, 2H), 6.20 (td, J = 4.0, 2.1 Hz, 1H), 2.26 (ttd, J = 6.6, 2.7, 1.8 Hz, 2H), 1.99 (tdt, J = 6.6, 4.0, 2.7 Hz, 2H), 1.63 – 1.57 (m, 2H), 1.51 – 1.45 (m, 2H); ^{13}C NMR (101 MHz, $CDCl_3$) δ 186.0, 149.1, 135.8, 135.3, 132.4, 129.6, 129.4 (t, J = 2.0 Hz), 129.3, 126.0, 117.1 (qt, J = 287.9, 40.0 Hz), 110.9 (tq, J = 271.3, 43.3 Hz), 27.0, 25.6, 22.3, 21.6; ^{19}F NMR (376 MHz, $CDCl_3$) δ –82.6 (s, 3F), –94.4 (s, 2F); HRMS (ESI$^+$) *m/z* calcd for $C_{17}H_{15}F_5N_3O$ [M+H]$^+$: 372.11298, found 372.11319.

*(4-butyl-1-(perfluoroethyl)-1H-1,2,3-triazol-5-yl)(phenyl)methanone (**3i**):*

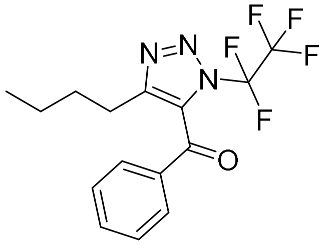

Prepared according to the general procedure. Yield: 215.0 mg, 62%, pale yellow oil; eluent: 10:1 cyclohexane/EtOAc. ^{1}H NMR (400 MHz, $CDCl_3$) δ 7.79 – 7.75 (m, 2H), 7.73 – 7.68 (m, 1H), 7.56 – 7.51 (m, 2H), 2.58 – 2.53 (m, 2H), 1.64 – 1.56 (m, 2H), 1.23 (dq, *J* = 14.5, 7.3 Hz, 2H), 0.82 – 0.77 (m, 3H); ^{13}C NMR (101 MHz, $CDCl_3$) δ 185.2, 148.9, 135.9, 135.4, 131.8 (d, *J* = 2.2 Hz), 129.7, 129.4, 117.1 (qt, *J* = 287.9, 40.0 Hz), 110.9 (tq, *J* = 271.8, 43.1 Hz), 31.0, 24.9, 22.2, 13.6; ^{19}F NMR (376 MHz, $CDCl_3$) δ –82.6 (s, 3F), –94.5 (s, 2F); HRMS (ESI$^+$) *m/z* calcd for $C_{15}H_{15}F_5N_3O$ $[M+H]^+$: 348.11298, found 348.11273.

*2-methyl-1-(4-phenyl-1-(trifluoromethyl)-1H-1,2,3-triazol-5-yl)prop-2-en-1-one (**3j**):*

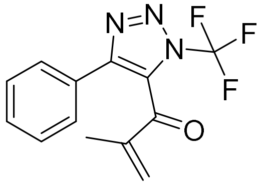

Prepared according to the general procedure. Yield: 182.0 mg, 65%, yellow oil; eluent: 10:1 cyclohexane/EtOAc. ^{1}H NMR (401 MHz, $CDCl_3$) δ 7.63 – 7.58 (m, 2H), 7.45 – 7.39 (m, 3H), 6.06 (q, *J* = 1.5 Hz, 1H), 5.62 (q, *J* = 1.0 Hz, 1H), 2.05 (t, *J* = 1.2 Hz, 3H); ^{13}C NMR (101 MHz, $CDCl_3$) δ 187.7, 146.9, 144.5, 133.0, 129.8, 129.3, 129.1, 128.1, 127.7, 117.7 (q, *J* = 270.3 Hz), 16.6; ^{19}F NMR (377 MHz, $CDCl_3$) δ –56.8 (s, 3F); HRMS (ESI$^+$) *m/z* calcd for $C_{13}H_{11}F_3N_3O$ $[M+H]^+$: 282.08487, found 282.08483.

*ethyl 2,2-difluoro-2-(5-methacryloyl-4-phenyl-1H-1,2,3-triazol-1-yl)acetate (**3k**):*

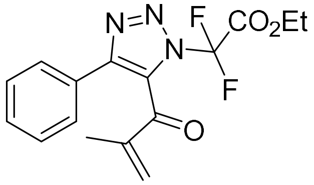

Prepared according to the general procedure. Yield: 132.0 mg, 39%, pale yellow oil; eluent: 10:1 cyclohexane/EtOAc. ^{1}H NMR (401 MHz, $CDCl_3$) δ 7.64 – 7.58 (m, 2H), 7.44 – 7.39 (m, 3H), 6.03 (q, *J* = 1.5 Hz, 1H), 5.67 (q, *J* = 1.1 Hz, 1H), 4.53 (q, *J* = 7.2 Hz, 2H), 2.06 – 2.05 (m, 3H), 1.43 (td, *J* = 7.2, 0.9 Hz, 3H); ^{13}C NMR (101 MHz, $CDCl_3$) δ 188.0, 158.2 (t, *J* = 33.9 Hz), 147.1, 144.1, 132.8, 129.6, 129.0, 128.5, 127.8, 126.2, 110.2 (t, *J* = 269.0 Hz), 65.2, 16.7, 13.9; ^{19}F NMR (377 MHz, $CDCl_3$) δ –85.7 (s, 2F); HRMS (ESI$^+$) *m/z* calcd for $C_{16}H_{16}F_2N_3O_3$ $[M+H]^+$: 336.11542, found 336.11563.

*phenyl(4-phenyl-1-(1,1,2,2-tetrafluoroethyl)-1H-1,2,3-triazol-5-yl)methanone (**3l**):*

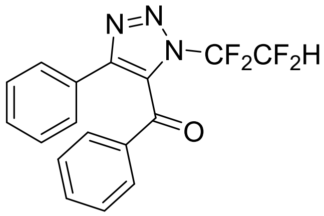

Prepared according to the general procedure. Yield: 202.9 mg, 58%, pale yellow oil; eluent: 97:3 cyclohexane/EtOAc. ^{1}H NMR (401 MHz, $CDCl_3$) δ 7.78 (dd, *J* = 8.2, 1.8 Hz, 2H), 7.65 – 7.58 (m, 3H), 7.47 – 7.41 (m, 2H), 7.32 (td, *J* = 4.3, 1.8 Hz, 3H), 6.81 (tt, *J* = 52.3, 5.0, 1.7 Hz, 1H); ^{13}C NMR (101 MHz, $CDCl_3$) δ 186.1, 147.2, 135.6, 135.2, 130.3 (d, *J* = 1.8 Hz), 130.0, 129.7, 129.3, 129.0, 128.0, 127.8, 113.0 (tt, *J* = 269.2, 29.3 Hz), 107.8 (tt, *J* = 254.6, 33.7 Hz); ^{19}F NMR (377 MHz, $CDCl_3$) δ –97.0 (td, *J* = 8.3, 4.8 Hz, 2F), –137.6 (dt, *J* = 52.2, 8.3 Hz, 2F); HRMS (ESI$^+$) *m/z* calcd for $C_{17}H_{12}F_4N_3O$ $[M+H]^+$: 350.09110, found 350.09140.

*(1-(perfluoroethyl)-4-phenyl-1H-1,2,3-triazol-5-yl)(phenyl)methanone (**3m**):*

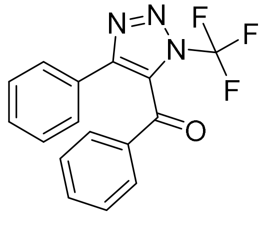

Prepared according to the general procedure. Yield: 191.5 mg, 60%, yellow oil; eluent: 2:1 pentane/toluene. ^{1}H NMR (401 MHz, $CDCl_3$) δ 7.78 – 7.74 (m, 2H), 7.64 – 7.57 (m, 3H), 7.45 – 7.40 (m, 2H), 7.33 – 7.28 (m, 3H); ^{13}C NMR (101 MHz, $CDCl_3$) δ 185.7, 147.3, 135.6, 135.1, 129.9, 129.7, 129.5, 129.4, 129.0, 127.9 (2C), 117.8 (q, *J* = 270.3 Hz); ^{19}F NMR (377 MHz, $CDCl_3$) δ –56.6 (s, 3F); HRMS (ESI$^+$) *m/z* calcd for $C_{16}H_{11}F_3N_3O$ [M+H]$^+$: 318.08487, found 318.08484.

*phenyl(4-phenyl-1-tosyl-1H-1,2,3-triazol-5-yl)methanone (**3n**):*

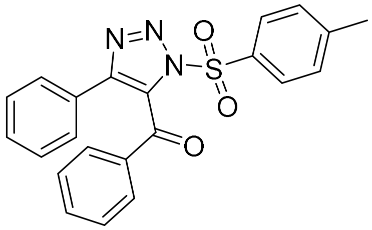

Prepared according to the general procedure. Yield: 41.7 mg, 21%, white solid; eluent: 12:88 cyclohexane/EtOAc. ^{1}H NMR (400 MHz, $CDCl_3$) δ 8.00 – 7.97 (m, 2H), 7.82 – 7.78 (dq, *J* = 8.1, 1.5 Hz, 2H), 7.65 – 7.57 (m, 3H), 7.48 – 7.42 (m, 2H), 7.41 – 7.37 (m, 2H), 7.29 – 7.25 (m, 3H), 2.45 (s, 3H); ^{13}C NMR (101 MHz, $CDCl_3$) δ 187.5, 147.7, 145.2, 136.0, 135.2, 133.0, 130.7, 130.5, 129.9, 129.4, 129.3, 129.3, 129.0, 128.2, 127.4, 22.0; HRMS (ESI$^+$) *m/z* calcd for $C_{22}H_{18}N_3O_3S$ [M+H]$^+$: 404.10634, found 404.10638.

*cyclopropyl(1-(perfluoroethyl)-4-phenyl-1H-1,2,3-triazol-5-yl)methanone (**3o**):*

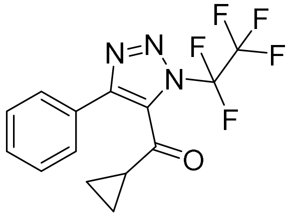

Prepared according to the general procedure. Yield: 170.0 mg, 51%, pale yellow oil; eluent: 1:2 toluene/pentane. ^{1}H NMR (401 MHz, $CDCl_3$) δ 7.75 – 7.70 (m, 2H), 7.51 – 7.46 (m, 3H), 2.09 (tt, *J* = 7.7, 4.5 Hz, 1H), 1.42 – 1.37 (m, 2H), 1.12 – 1.07 (m, 2H); ^{13}C NMR (101 MHz, $CDCl_3$) δ 193.8, 147.8, 132.6 (t, *J* = 2.0 Hz), 130.1, 129.1, 128.7, 128.1, 117.2 (qt, *J* = 287.9, 39.2 Hz), 111.0 (tq, *J* = 271.7, 42.9 Hz), 23.7, 15.2; ^{19}F NMR (377 MHz, $CDCl_3$) δ –81.9 (d, *J* = 1.8 Hz, 3F), –94.0 (d, *J* = 1.1 Hz, 2F); HRMS (ESI$^+$) *m/z* calcd for $C_{14}H_{11}F_5N_3O$ [M+H]$^+$: 332.08168, found 332.08189.

*1-(1-(perfluoroethyl)-4-phenyl-1H-1,2,3-triazol-5-yl)butan-1-one (**3p**):*

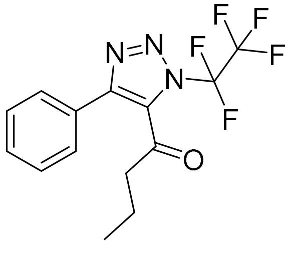

Prepared according to the general procedure. Yield: 143.0 mg, 43%, pale pink oil; eluent: 1:2 toluene/pentane. ^{1}H NMR (401 MHz, $CDCl_3$) δ 7.63 – 7.56 (m, 2H), 7.52 – 7.46 (m, 3H), 2.61 (t, *J* = 7.3 Hz, 2H), 1.70 – 1.61 (m, 2H), 0.87 (t, *J* = 7.4 Hz, 3H); ^{13}C NMR (101 MHz, $CDCl_3$) δ 194.2, 147.1, 132.6, 130.2, 129.3, 128.4, 128.1, 117.1 (qt, *J* = 287.9, 39.4 Hz), 111.0 (tq, *J* = 271.6, 43.3 Hz), 45.8, 17.0, 13.3; ^{19}F NMR (377 MHz, $CDCl_3$) δ –82.1 (s, 3F), –94.1 (s, 2F); HRMS (ESI$^+$) *m/z* calcd for $C_{14}H_{13}F_5N_3O$ [M+H]$^+$: 334.09733, found 334.09751.

*1-(1-(perfluoroethyl)-4-phenyl-1H-1,2,3-triazol-5-yl)-3-phenylpropan-1-one (**3q**):*

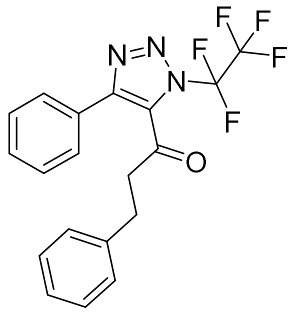

Prepared according to the general procedure. Yield: 208.0 mg, 53%, yellow oil; eluent: 1:2 toluene/pentane. ^{1}H NMR (401 MHz, $CDCl_3$) δ 7.51 – 7.46 (m, 3H), 7.45 – 7.40 (m, 2H), 7.28 – 7.21 (m, 3H), 7.08 – 7.05 (m, 2H), 2.99 – 2.97 (m, 4H); ^{13}C NMR (101 MHz, $CDCl_3$) δ 192.9, 147.5, 139.4, 132.2 (t, *J* = 2.2 Hz), 130.2, 129.3, 128.7, 128.6, 128.4, 127.9, 126.7, 117.1 (qt, *J* = 287.9, 39.4 Hz), 110.9 (tq, *J* = 272.0, 43.3 Hz), 45.4, 29.4; ^{19}F NMR (377 MHz, $CDCl_3$) δ –82.1 (s, 3F), –94.1 (s, 2F); HRMS (ESI^+) *m/z* calcd for $C_{19}H_{15}F_5N_3O$ $[M+H]^+$: 396.11298, found 396.11275.

*3-methyl-1-(1-(perfluoroethyl)-4-phenyl-1H-1,2,3-triazol-5-yl)butan-1-one (**3r**):*

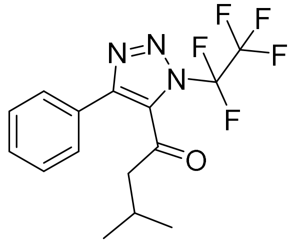

Prepared according to the general procedure. Yield: 288.0 mg, 83%, yellow oil; eluent: 1:2 toluene/pentane. ^{1}H NMR (401 MHz, $CDCl_3$) δ 7.61 – 7.58 (m, 2H), 7.51 – 7.48 (m, 3H), 2.51 (d, *J* = 6.6 Hz, 2H), 2.17 (dh, *J* = 13.3, 6.7 Hz, 1H), 0.87 (d, *J* = 6.7 Hz, 6H); ^{13}C NMR (101 MHz, $CDCl_3$) δ 193.5, 147.2, 132.7 (t, *J* = 2.0 Hz), 130.2, 129.2, 128.6, 128.1, 117.2 (qt, *J* = 287.9, 39.2 Hz), 111.0 (tq, *J* = 272.3, 42.9 Hz), 52.6, 24.2, 22.2; ^{19}F NMR (377 MHz, $CDCl_3$) δ –82.0 (s, 3F), –94.0 (s, 2F); HRMS (ESI^+) *m/z* calcd for $C_{15}H_{15}F_5N_3O$ $[M+H]^+$: 348.11298, found 348.11272.

*(E)-1-(1-(perfluoroethyl)-4-phenyl-1H-1,2,3-triazol-5-yl)-3-phenylprop-2-en-1-one (**3s**):*

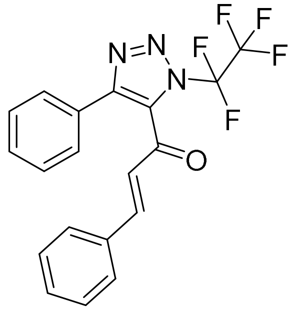

Prepared according to the general procedure. Yield: 139.0 mg, 35%, pale yellow oil; eluent: 94:6 cyclohexane/EtOAc. ^{1}H NMR (400 MHz, $CDCl_3$) δ 7.76 – 7.72 (m, 2H), 7.46 – 7.35 (m, 9H), 6.92 (d, *J* = 16.1 Hz, 1H); ^{13}C NMR (101 MHz, $CDCl_3$) δ 183.8, 149.4, 147.2, 133.3, 132.1, 131.4 (t, *J* = 1.8 Hz), 130.0, 129.3, 129.2, 129.1, 128.2, 128.0, 125.4, 117.2 (qt, *J* = 288.3, 39.4 Hz), 109.7 (tq, *J* = 272.2, 43.1 Hz); ^{19}F NMR (376 MHz, $CDCl_3$) δ –82.2 (s, 3F), –94.3 (s, 2F); HRMS (ESI^+) *m/z* calcd for $C_{19}H_{13}F_5N_3O$ $[M+H]^+$: 394.09733, found 394.09718.

*1-(1-(perfluoroethyl)-4-phenyl-1H-1,2,3-triazol-5-yl)prop-2-en-1-one (**3t**):*

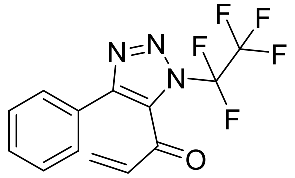

Prepared according to the general procedure. Yield: 137.0 mg, 43%, pale yellow oil; eluent: 1:2 toluene/pentane. ^{1}H NMR (400 MHz, $CDCl_3$) δ 7.67 – 7.62 (m, 2H), 7.47 – 7.43 (m, 3H), 6.57 (dd, *J* = 17.5, 10.6 Hz, 1H), 6.13 (d, *J* = 1.6 Hz, 1H), 6.10 (d, *J* = 8.6 Hz, 1H); ^{13}C NMR (101 MHz, $CDCl_3$) δ 184.8, 147.5, 135.7, 135.2, 130.4 (t, *J* = 1.8 Hz), 130.1, 129.2, 128.2, 127.8, 117.1 (qt, *J* = 287.9, 39.6 Hz), 110.96 (tq, *J* = 272.5, 43.4 Hz); ^{19}F NMR (376 MHz, $CDCl_3$) δ –82.4 (s, 3F), –94.4 (s, 2F); HRMS (ESI^+) *m/z* calcd for $C_{13}H_9F_5N_3O$ $[M+H]^+$: 318.06603, found 318.06577.

*(2-(methylthio)pyridin-3-yl)(1-(perfluoroethyl)-4-phenyl-1H-1,2,3-triazol-5-yl)methanone (**3u**):*

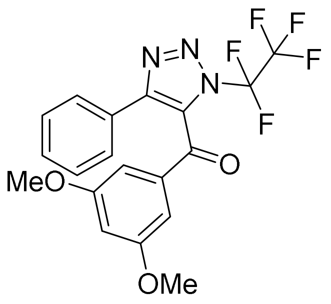

Prepared according to the general procedure. Yield: 194.0 mg, 45%, yellow oil; eluent: 95:5 cyclohexane/EtOAc. ^{1}H NMR (400 MHz, $CDCl_3$) δ 7.65 – 7.60 (m, 2H), 7.36 – 7.32 (m, 3H), 6.87 (d, *J* = 2.3 Hz, 2H), 6.68 (t, *J* = 2.3 Hz, 1H), 3.74 (s, 6H); ^{13}C NMR (101 MHz, $CDCl_3$) δ 185.5, 161.4, 146.8, 136.9, 130.5 (t, *J* = 1.8 Hz), 129.8, 129.1, 127.9, 127.7, 117.1 (qt, *J* = 288.1, 40.0 Hz), 111.0 (tq, *J* = 272.8, 43.3 Hz), 108.2, 107.5, 55.8; ^{19}F NMR (376 MHz, $CDCl_3$) δ –82.6 (s, 3F), –94.5 (s, 2F); HRMS (ESI$^+$) *m/z* calcd for $C_{19}H_{15}F_5N_3O_3$ $[M+H]^+$: 428.10281, found 428.10242.

*(3,5-dimethoxyphenyl)(4-(3,5-dimethoxyphenyl)-1-(perfluoroethyl)-1H-1,2,3-triazol-5-yl)methanone (**3v**):*

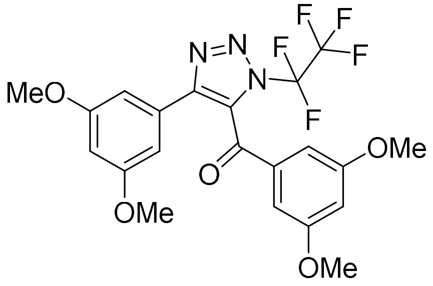

Prepared according to the general procedure. Yield: 217.0 mg, 45%, pale yellow oil; eluent: 8:2 cyclohexane/EtOAc. ^{1}H NMR (401 MHz, $CDCl_3$) δ 6.87 (d, *J* = 2.3 Hz, 2H), 6.76 (d, *J* = 2.3 Hz, 2H), 6.68 (t, *J* = 2.3 Hz, 1H), 6.41 (t, *J* = 2.3 Hz, 1H), 3.74 (s, 6H), 3.67 (s, 6H); ^{13}C NMR (101 MHz, $CDCl_3$) δ 185.5, 161.4, 161.2, 146.6, 137.0, 130.7, 129.4, 117.0 (qt, *J* = 287.9, 40.0 Hz), 110.9 (tq, *J* = 272.4, 43.5 Hz), 108.0, 107.4, 105.6, 102.3, 55.7, 55.4; ^{19}F NMR (377 MHz, $CDCl_3$) δ –82.6 (s, 3F), –94.5 (s, 2F); HRMS (ESI$^+$) *m/z* calcd for $C_{21}H_{19}F_5N_3O_5$ $[M+H]^+$: 488.12394, found 488.12404.

*(3,5-dimethoxyphenyl)(4-(4-nitrophenyl)-1-(perfluoroethyl)-1H-1,2,3-triazol-5-yl)methanone (**3w**):*

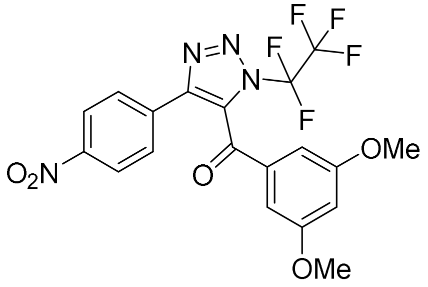

Prepared according to the general procedure. Yield: 269.5 mg, 57%, yellow oil; eluent: 92:8 cyclohexane/EtOAc. ^{1}H NMR (400 MHz, $CDCl_3$) δ 8.24 – 8.20 (m, 2H), 7.86 – 7.83 (m, 2H), 6.87 (d, *J* = 2.3 Hz, 2H), 6.74 (t, *J* = 2.3 Hz, 1H), 3.79 (s, 6H); ^{13}C NMR (101 MHz, $CDCl_3$) δ 184.9, 161.6, 148.3, 144.3, 136.6, 133.9, 132.1 (t, *J* = 1.8 Hz), 128.3, 124.4, 116.9 (qt, *J* = 288.3, 39.6 Hz), 110.9 (tq, *J* = 273.4, 43.8 Hz), 108.2, 107.5, 55.8; ^{19}F NMR (376 MHz, $CDCl_3$) δ –82.6 (s, 3F), –94.6 (s, 2F); HRMS (ESI$^+$) *m/z* calcd for $C_{19}H_{14}F_5N_4O_5$ $[M+H]^+$: 473.08789, found 473.08777.

*(3,5-dimethoxyphenyl)(4-(4-methoxyphenyl)-1-(perfluoroethyl)-1H-1,2,3-triazol-5-yl)methanone (**3x**):*

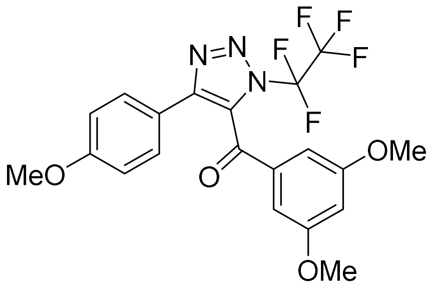

Prepared according to the general procedure. Yield: 128.0 mg, 28%, colorless oil; eluent: 10:1 cyclohexane/EtOAc. ^{1}H NMR (400 MHz, $CDCl_3$) δ 7.57 – 7.53 (m, 2H), 6.88 – 6.85 (m, 3H), 6.84 (d, *J* = 2.2 Hz, 1H), 6.68 (t, *J* = 2.3 Hz, 1H), 3.78 (s, 3H), 3.74 (s, 6H); ^{13}C NMR (101 MHz, $CDCl_3$) δ 185.7, 161.4, 160.7, 146.7, 137.0, 129.6 (t, *J* = 1.8 Hz), 129.2, 120.3, 176.3 (qt, *J* = 287.9, 40.0 Hz), 114.6, 111.0 (tq, *J* = 272.1, 43.5 Hz), 108.1, 107.5, 55.8, 55.4; ^{19}F NMR (376 MHz, $CDCl_3$) δ –82.6 (s, 3F), –94.5 (s, 2F); HRMS (ESI$^+$) *m/z* calcd for $C_{20}H_{17}F_5N_3O_4$ [M+H]$^+$: 458.11337, found 458.11320.

*(4-methoxyphenyl)(1-(perfluoroethyl)-4-phenyl-1H-1,2,3-triazol-5-yl)methanone (**3y**):*

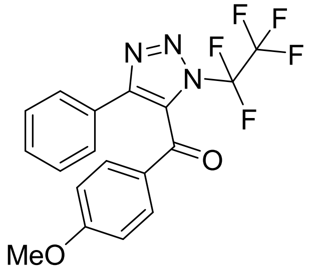

Prepared according to the general procedure. Yield: 206.0 mg, 52%. Scale up reaction: Under air atmosphere, a 100 mL round bottom flask was charged with the corresponding copper(I) acetylide (5.0 mmol, 0.823 g, 1.0 equiv.), 3Å molecular sieves (1.2 g) and a stirring bar. Then it was cooled to 0 °C, followed by the addition of a solution of $CF_3CF_2N_3$ in THF (~7.5 mmol, 20 mL), EDIPA (2.6 ml, 15.0 mmol, 3.0 equiv.) and 4-methoxybenzoyl chloride (1.36 ml, 10.00 mmol, 2.0 equiv.). The reaction mixture was left to warm to room temperature and stirred for 17 h. The crude suspension was filtered via a short plug of silica gel and washed with THF (3 x 20 mL). The filtrate was evaporated with Celite (water bath 40 °C) and purified by flash column chromatography (silica gel, eluent: 92:8 cyclohexane/EtOAc) to obtain triazole. Yield: 70%, 1.388 g, pale yellow oil; ^{1}H NMR (401 MHz, $CDCl_3$) δ 7.76 – 7.70 (m, 2H), 7.67 – 7.62 (m, 2H), 7.34 – 7.30 (m, 3H), 6.98 – 6.83 (m, 2H), 3.85 (s, 3H); ^{13}C NMR (101 MHz, $CDCl_3$) δ 184.0, 165.6, 146.4, 132.5, 130.8, 129.6, 129.1, 128.3, 128.0, 127.6, 117.1 (qt, *J* = 288.3, 40.0 Hz), 114.7, 111.0 (tq, *J* = 272.7, 43.3 Hz), 55.8; ^{19}F NMR (377 MHz, $CDCl_3$) δ –82.6 (s, 3F), –94.7 (s, 2F); HRMS (ESI$^+$) *m/z* calcd for $C_{18}H_{13}F_5N_3O_2$ [M+H]$^+$: 398.09224, found 398.09231.

*(4-chlorophenyl)(1-(perfluoroethyl)-4-phenyl-1H-1,2,3-triazol-5-yl)methanone (**3z**):*

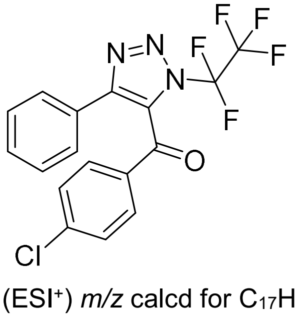

Prepared according to the general procedure. Yield: 185.0 mg, 46%, pale yellow oil; eluent: 96:4 cyclohexane/EtOAc. ^{1}H NMR (400 MHz, $CDCl_3$) δ 7.71 – 7.67 (m, 2H), 7.61 – 7.58 (m, 2H), 7.42 – 7.39 (m, 2H), 7.35 – 7.31 (m, 3H); ^{13}C NMR (101 MHz, $CDCl_3$) δ 184.6, 147.0, 142.5, 133.5, 131.1, 130.0 (t, *J* = 1.8 Hz), 129.9, 129.9, 129.2, 127.8, 127.6, 117.0 (qt, *J* = 288.3, 40.0 Hz), 111.0 (tq, *J* = 272.7, 43.5 Hz); ^{19}F NMR (377 MHz, $CDCl_3$) δ –82.6 (s, 3F), –94.5 (s, 2F); HRMS (ESI$^+$) *m/z* calcd for $C_{17}H_{10}F_5ClN_3O$ [M+H]$^+$: 402.04271, found 402.04263.

*naphthalen-2-yl(1-(perfluoroethyl)-4-phenyl-1H-1,2,3-triazol-5-yl)methanone (**3aa**):*

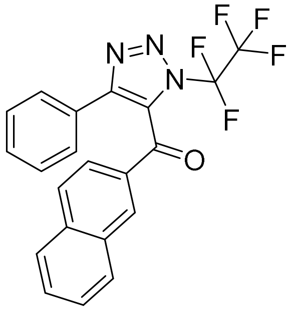

Prepared according to the general procedure. Yield: 199.0 mg, 48%, white solid; eluent: 10:1 cyclohexane/EtOAc. ^{1}H NMR (400 MHz, $CDCl_3$) δ 8.15 – 8.12 (m, 1H), 7.98 (dd, *J* = 8.7, 1.8 Hz, 1H), 7.94 – 7.91 (m, 1H), 7.89 – 7.81 (m, 2H), 7.71 – 7.67 (m, 2H), 7.64 (ddd, *J* = 8.3, 7.0, 1.3 Hz, 1H), 7.54 (ddd, *J* = 8.2, 6.9, 1.2 Hz, 1H), 7.32 – 7.26 (m, 3H); ^{13}C NMR (101 MHz, $CDCl_3$) δ 185.7, 146.9, 136.7, 133.4, 132.7, 132.4, 130.7, 130.1, 130.1, 129.7, 129.6, 129.1, 128.1, 127.9, 127.6, 127.6, 123.7, 117.1 (qt, *J* = 287.9, 40.0 Hz), 111.0 (tq, *J* = 272.3, 43.3 Hz); ^{19}F NMR (376 MHz, $CDCl_3$) δ –82.6 (s, 3F), –94.5 (s, 2F); HRMS (ESI$^+$) *m/z* calcd for $C_{21}H_{13}F_5N_3O$ $[M+H]^+$: 418.09733, found 418.09748.

*(1-(perfluoroethyl)-4-phenyl-1H-1,2,3-triazol-5-yl)(thiophen-2-yl)methanone (**3ab**):*

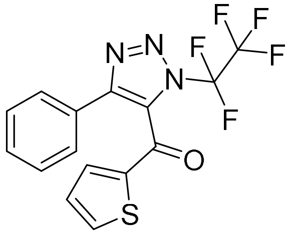

Prepared according to the general procedure. Yield: 212.0 mg, 57%, pale yellow oil; eluent: 97:3 cyclohexane/EtOAc. ^{1}H NMR (401 MHz, $CDCl_3$) δ 7.81 (dd, *J* = 4.9, 1.1 Hz, 1H), 7.70 – 7.65 (m, 2H), 7.37 – 7.32 (m, 4H), 7.03 (dd, *J* = 4.9, 3.9 Hz, 1H); ^{13}C NMR (101 MHz, $CDCl_3$) δ 177.1, 146.8, 142.2, 138.2, 136.7, 130.3 (t, *J* = 1.8 Hz), 129.8, 129.2, 129.1, 127.8 (2C), 117.1 (qt, *J* = 287.9, 39.6 Hz), 111.0 (tq, *J* = 273.2, 43.3 Hz); ^{19}F NMR (377 MHz, $CDCl_3$) δ –82.6 (s, 3F), –94.8 (s, 2F); HRMS (ESI$^+$) *m/z* calcd for $C_{15}H_9F_5N_3OS$ $[M+H]^+$: 374.03810, found 374.03851.

*furan-2-yl(1-(perfluoroethyl)-4-phenyl-1H-1,2,3-triazol-5-yl)methanone (**3ac**):*

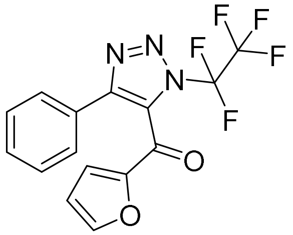

Prepared according to the general procedure. Yield: 129.0 mg, 36%, white solid; eluent: 96:4 to 92:8 cyclohexane/EtOAc. ^{1}H NMR (401 MHz, $CDCl_3$) δ 7.67 – 7.63 (m, 2H), 7.62 – 7.59 (m, 1H), 7.38 – 7.35 (m, 3H), 7.12 (d, *J* = 3.8 Hz, 1H), 6.53 (dd, *J* = 3.7, 1.7 Hz, 1H); ^{13}C NMR (101 MHz, $CDCl_3$) δ 171.7, 151.3, 149.6, 147.5, 129.8 (2C), 129.1, 128.0, 127.8, 122.8, 117.1 (qt, *J* = 288.3, 40.0 Hz), 113.7, 111.0 (tq, *J* = 272.9, 43.5 Hz); ^{19}F NMR (377 MHz, $CDCl_3$) δ –82.6 (s, 3F), –95.0 (s, 2F); HRMS (ESI$^+$) *m/z* calcd for $C_{15}H_9F_5N_3O_2$ $[M+H]^+$: 358.06094, found 358.06066.

*(2-(methylthio)pyridin-3-yl)(1-(perfluoroethyl)-4-phenyl-1H-1,2,3-triazol-5-yl)methanone (**3ad**):*

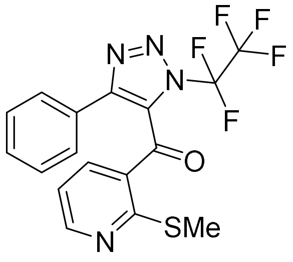

Prepared according to the general procedure. Yield: 67.0 mg, 16%, yellow oil; eluent: 10:1 cyclohexane/EtOAc. ^{1}H NMR (400 MHz, $CDCl_3$) δ 8.56 (dd, *J* = 4.7, 1.8 Hz, 1H), 7.57 – 7.53 (m, 2H), 7.48 (dd, *J* = 7.9, 1.8 Hz, 1H), 7.33 – 7.30 (m, 3H), 6.89 (dd, *J* = 7.9, 4.7 Hz, 1H), 2.61 (s, 3H); ^{13}C NMR (101 MHz, $CDCl_3$) δ 184.0, 164.8, 153.6, 147.2, 140.2, 130.2 (t, *J* = 1.8 Hz), 129.9, 129.2, 127.7, 127.6, 127.5, 118.0, 115.6 (qt, *J* = 287.8, 39.6 Hz), 111.0 (tq, *J* = 272.7, 43.6

Hz), 14.0; ^{19}F NMR (376 MHz, $CDCl_3$) δ –82.5 (s, 3F), –94.6 (s, 2F); HRMS (ESI$^+$) *m/z* calcd for $C_{17}H_{12}F_5N_4OS$ [M+H]$^+$: 415.06465, found 415.06443.

Synthesis of cyclopentenones **4**

General procedure A:

Under air atmosphere, a 10 mL screw-cap glass reaction tube was charged with $AlCl_3$ (1.05 equiv.) and stirring bar, then 0.1M solution of the starting triazole **3** in DCE was added (0.068–0.204 mmol in 0.68–2.04 ml). The reaction mixture was heated to 60 °C and stirred for 1-2 h. The conversion was monitored by ^{19}F NMR spectroscopy or TLC. The dark reaction mixture was filtered via paper, washed with Et_2O, evaporated, and purified by column chromatography (silica gel, pentane/DCM) to obtain pure product.

General procedure B:

Under air atmosphere, a 10 mL screw-cap glass reaction tube was charged with 0.1M solution of the starting triazole **3** in DCE (0.068–0.204 mmol in 0.68–2.04 ml) and a stirring bar. Then, $BF_3{\cdot}OEt_2$ (1.05 equiv.) was added. The reaction mixture was heated to 60 °C and stirred for 1-2 h. The conversion was monitored by ^{19}F NMR spectroscopy or TLC. The dark reaction mixture was filtered via paper, washed with Et_2O, evaporated, and purified by column chromatography (silica gel, pentane/DCM) to obtain pure product.

Preparation and characterization of cyclopentenone derivatives **4**

*(Z)-N-(4-chloro-4-methyl-5-oxo-2-phenylcyclopent-1-en-1-yl)-2,2,2-trifluoroacetimidoyl chloride (**4a**):*

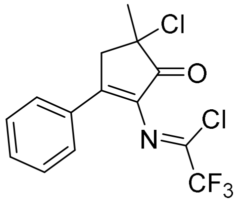

Prepared according to the general procedure A from **3a** (43.6 mg, 0.1315 mmol). Yield: 19.0 mg, 43%. Scale up reaction: Under air atmosphere, a 100 mL round bottom flask was charged with $AlCl_3$ (308.2 mg, 2.311 mmol, 1.05 equiv.) and a stirring bar, then a solution of **3a** was added (739 mg) in DCE (22 ml). The reaction mixture was heated to 60 °C and stirred for 2 h. The conversion was monitored by ^{19}F NMR spectroscopy. The dark reaction mixture was filtered via paper, washed with Et_2O, evaporated, and purified by column chromatography (silica gel, eluent: 4:1 to 3:1 pentane/DCM) to obtain pure product. Yield: 41%, 302.5 mg, yellow oil; ^{1}H NMR (400 MHz, $CDCl_3$) δ 7.64 – 7.61 (m, 2H), 7.54 – 7.46 (m, 3H), 3.65 (d, *J* = 18.2 Hz, 1H), 3.42 (d, *J* = 18.2 Hz, 1H), 1.84 (s, 3H); ^{13}C NMR (101 MHz, $CDCl_3$) δ 191.9, 150.5, 138.4 (q, *J* = 43.6 Hz), 135.2, 132.1, 132.1, 129.3, 128.8, 116.8 (q, *J* = 278.0 Hz), 63.1, 46.0, 27.0; ^{19}F NMR (376 MHz, $CDCl_3$) δ –71.0 (s, 3F); HRMS (APCI$^+$) *m/z* calcd for $C_{14}H_{11}F_3Cl_2NO$ [M+H]$^+$: 336.01643, found 336.01681.

*(Z)-N-(4-chloro-2-(4-methoxyphenyl)-4-methyl-5-oxocyclopent-1-en-1-yl)-2,2,2-trifluoroacetimidoyl chloride (**4b**):*

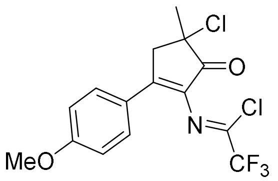

Prepared according to the general procedure A from **3b** (56.2 mg, 0.156 mmol). Yield: 29.5 mg, 52%, yellow oil; eluent: 3:1 pentane/DCM. ^{1}H NMR (400 MHz, $CDCl_3$) δ 7.64 – 7.60 (m, 2H), 7.01 – 6.97 (m, 2H), 3.88 (s, 3H), 3.62 (d, *J* = 18.1 Hz, 1H), 3.39 (d, *J* = 18.1 Hz, 1H), 1.82 (s, 3H); ^{13}C NMR (151 MHz, $CDCl_3$) δ 191.4, 162.8, 150.8, 137.8 (q, *J* = 43.1 Hz), 133.5, 131.0, 124.8, 116.9 (q, *J* = 277.5 Hz), 114.8, 63.4, 55.7, 46.0, 27.1; ^{19}F NMR (376 MHz, $CDCl_3$) δ –71.4 (s, 3F); HRMS (ESI$^+$) *m/z* calcd for $C_{15}H_{13}F_3Cl_2NO_2$ $[M+H]^+$: 366.02700, found 366.02672.

*(Z)-N-(4-chloro-4-methyl-2-(4-nitrophenyl)-5-oxocyclopent-1-en-1-yl)-2,2,2-trifluoroacetimidoyl chloride (**4c**):*

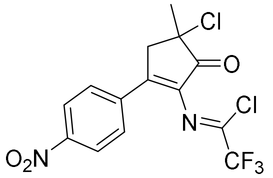

Prepared according to the general procedure A from **3c** (48.0 mg, 0.128 mmol). Yield: 19.0 mg, 39%, colorless oil; eluent: 1:1 pentane/DCM. ^{1}H NMR (400 MHz, $CDCl_3$) δ 8.35 – 8.31 (m, 2H), 7.81 – 7.77 (m, 2H), 3.66 (d, *J* = 18.2 Hz, 1H), 3.44 (d, *J* = 18.2 Hz, 1H), 1.87 (s, 3H); ^{13}C NMR (151 MHz, $CDCl_3$) δ 191.7, 149.1, 146.8, 139.8 (q, *J* = 43.7 Hz), 137.9, 137.5, 129.4, 124.3, 116.6 (q, *J* = 278.1 Hz), 62.7, 46.0, 26.6; ^{19}F NMR (376 MHz, $CDCl_3$) δ –71.5 (s, 3F); HRMS (APCI$^+$) *m/z* calcd for $C_{14}H_9F_3Cl_2N_2O_3$ $[M]^+$: 379.99368, found 379.99270.

*ethyl (Z)-2-chloro-2-((4-chloro-4-methyl-5-oxo-2-phenylcyclopent-1-en-1-yl)imino)acetate (**4d**):*

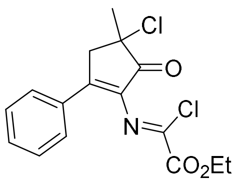

Prepared according to the general procedure A from **3k** (50.0 mg, 0.149 mmol). Yield: 10.0 mg, 20%, yellow oil; eluent: 1:1 pentane/DCM. ^{1}H NMR (400 MHz, $CDCl_3$) δ 7.70 – 7.65 (m, 2H), 7.52 – 7.47 (m, 3H), 4.50 (q, *J* = 7.1 Hz, 2H), 3.66 (d, *J* = 18.1 Hz, 1H), 3.43 (d, *J* = 18.1 Hz, 1H), 1.86 (s, 3H), 1.48 (t, *J* = 7.1 Hz, 3H); ^{13}C NMR (126 MHz, $CDCl_3$) δ 192.3, 158.6, 149.0, 141.1, 137.0, 132.5, 131.7, 129.2, 128.8, 64.5, 63.4, 46.0, 27.2, 14.2; HRMS (ESI$^+$) *m/z* calcd for $C_{16}H_{16}Cl_2NO_3$ $[M+H]^+$: 340.05018, found 340.05011.

*2,2,2-trifluoro-N-(4-fluoro-4-methyl-5-oxo-2-phenylcyclopent-1-en-1-yl)acetamide (**4e**):*

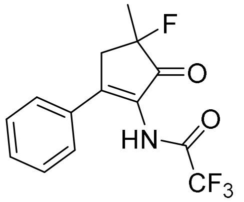

Prepared according to the general procedure B from **3a** (61.3 mg, 0.185 mmol). Yield: 24.2 mg, 43%, yellow oil; eluent: 2:1 cyclohexane/EtOAc. ^{1}H NMR (401 MHz, $CDCl_3$) δ 8.00 (bs, 1H), 7.52 – 7.45 (m, 5H), 3.43 (ddd, *J* = 21.3, 17.7, 1.0 Hz, 1H), 3.21 (ddd, *J* = 17.7, 9.8, 1.0 Hz, 1H), 1.66 (d, *J* = 22.6 Hz, 3H); ^{13}C NMR (101 MHz, $CDCl_3$) δ 197.8 (d, *J* = 19.4 Hz), 157.7 (d, *J* = 2.6 Hz), 154.3 (q, *J* = 38.5 Hz), 133.5, 132.1, 129.0, 127.8, 126.5 (d, *J* = 2.2 Hz), 115.5 (q, *J* = 288.1 Hz), 91.6 (d, *J* = 184.1 Hz), 42.5 (d, *J* = 24.9 Hz), 21.7 (d, *J* = 26.8 Hz); ^{19}F NMR (377 MHz, $CDCl_3$) δ –76.0 (s, 3F), –151.31 to –151.57 (m, 1H); HRMS (EI) *m/z* calcd for $C_{14}H_{11}F_4NO_2$ $[M]^+$: 301.0720, found 301.0718.

*2,2,2-trifluoro-N-(4-fluoro-2-(4-methoxyphenyl)-4-methyl-5-oxocyclopent-1-en-1-yl)acetamide (**4f**):*

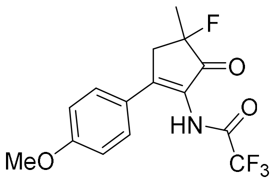

Prepared according to the general procedure B from **3b** (39.4 mg, 0.109 mmol). Yield: 23.1 mg, 64%, white solid; eluent: 3:1 cyclohexane/EtOAc. ^{1}H NMR (401 MHz, $CDCl_3$) δ 8.02 (bs, 1H), 7.52 – 7.47 (m, 2H), 7.00 – 6.94 (m, 2H), 3.87 (s, 3H), 3.40 (ddd, *J* = 21.5, 17.6, 1.0 Hz, 1H), 3.19 (ddd, *J* = 17.5, 9.9, 0.9 Hz, 1H), 1.64 (d, *J* = 22.6 Hz, 3H); ^{13}C NMR (101 MHz, $CDCl_3$) δ 197.4 (d, *J* = 19.1 Hz), 162.7, 157.4, 154.3 (q, *J* = 38.2 Hz), 130.1, 125.5, 124.7 (d, *J* = 2.2 Hz), 115.4 (q, *J* = 288.3 Hz), 114.3, 91.4 (d, *J* = 183.4 Hz), 55.5, 42.2 (d, *J* = 24.9 Hz), 21.7 (d, *J* = 27.1 Hz); ^{19}F NMR (377 MHz, $CDCl_3$) δ –76.0 (s, 3F), –150.62 to –150.88 (m, 1H); HRMS (ESI$^+$) *m/z* calcd for $C_{15}H_{14}F_4NO_3$ [M+H]$^+$: 332.09043, found 332.09024.

*2,2,2-trifluoro-N-(4-fluoro-4-methyl-2-(4-nitrophenyl)-5-oxocyclopent-1-en-1-yl)acetamide (**4g**):*

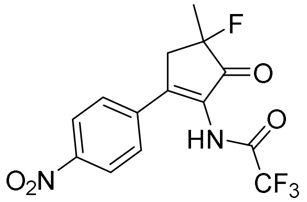

Prepared according to the general procedure B from **3c** (25.5 mg, 0.068 mmol). Yield: 2.1 mg, 9%, white solid; eluent: 2:1 cyclohexane/EtOAc. ^{1}H NMR (401 MHz, $CDCl_3$) δ 8.33 – 8.29 (m, 2H), 8.05 (bs, 1H), 7.58 – 7.55 (m, 2H), 3.44 (dd, *J* = 21.1, 17.8 Hz, 1H), 3.23 (dd, *J* = 17.7, 10.5 Hz, 1H), 1.70 (d, *J* = 22.6 Hz, 3H); ^{13}C NMR (151 MHz, $CDCl_3$) δ 197.2 (d, *J* = 19.3 Hz), 154.0 (q, *J* = 39.1 Hz), 152.2 (d, *J* = 2.2 Hz), 148.8, 140.1, 128.3, 123.8, 115.2 (q, *J* = 288.2 Hz), 91.1 (d, *J* = 184.7 Hz), 42.7 (d, *J* = 25.4 Hz), 29.7, 21.4 (d, *J* = 27.1 Hz); ^{19}F NMR (377 MHz, $CDCl_3$) δ –76.0 (s, 3F), –150.99 to –151.26 (m, 1H); HRMS (EI) *m/z* calcd for $C_{14}H_{10}F_4N_2O_4$ [M]$^+$: 346.0571, found 346.0572.

*5-fluoro-2-isocyanato-5-methyl-3-phenylcyclopent-2-en-1-one (**4h**):*

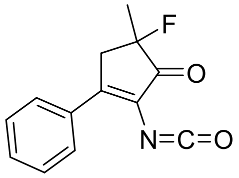

Prepared according to the general procedure B from **3j** (25.5 mg, 0.091 mmol). Yield: 8.8 mg, 42%, yellow oil; eluent: 2:1 cyclohexane/EtOAc. ^{1}H NMR (400 MHz, $CDCl_3$) δ 8.02 – 7.97 (m, 2H), 7.56 – 7.51 (m, 3H), 3.37 (dd, *J* = 21.3, 17.7 Hz, 1H), 3.18 (dd, *J* = 17.6, 9.9 Hz, 1H), 1.69 (d, *J* = 22.6 Hz, 3H); ^{13}C NMR (101 MHz, $CDCl_3$) δ 197.1 (d, *J* = 19.4 Hz), 149.6 (d, *J* = 2.2 Hz), 133.6, 132.1, 132.0, 129.1, 128.6, 126.9, 91.2 (d, *J* = 183.8 Hz), 40.8 (d, *J* = 25.3 Hz), 22.0 (d, *J* = 26.8 Hz); ^{19}F NMR (376 MHz, $CDCl_3$) δ –150.59 to –150.85 (m, 1H); HRMS (APCI$^+$) *m/z* calcd for $C_{13}H_{11}FNO_2$ [M+H]$^+$: 232.07683, found 232.07660.

Mechanistic evidence of the vinyl cation intermediate

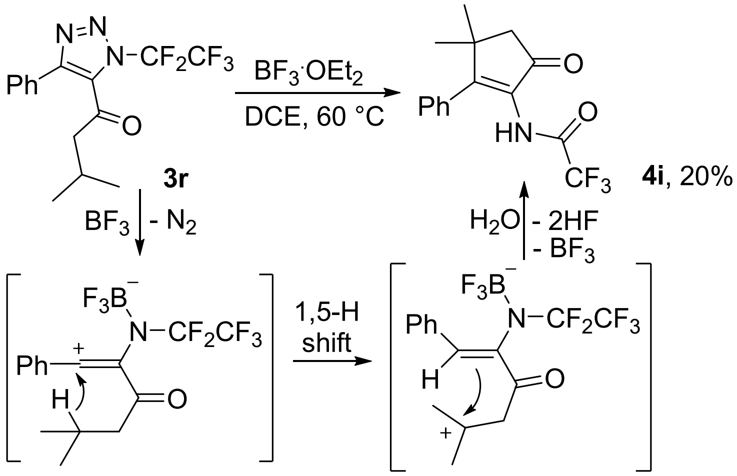

Scheme S1 Cyclization of triazole **3r** to cyclopentenone **4i**.

*N-(3,3-dimethyl-5-oxo-2-phenylcyclopent-1-en-1-yl)-2,2,2-trifluoroacetamide (**4i**):*

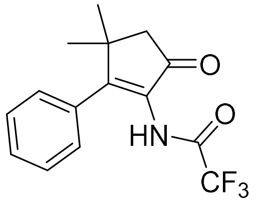

Prepared according to the general procedure B from **3r** (30.4 mg, 0.088 mmol). Yield: 5.0 mg, 20%, colorless oil; eluent: 4:1 cyclohexane/EtOAc. ^{1}H NMR (401 MHz, $CDCl_3$) δ 7.59 (bs, 1H), 7.45 – 7.38 (m, 3H), 7.24 – 7.21 (m, 2H), 2.56 (s, 2H), 1.35 (s, 6H); ^{13}C NMR (101 MHz, $CDCl_3$) δ 201.2, 171.1, 154.0 (q, J = 38.1 Hz), 133.8, 130.0, 129.3, 128.6, 126.9, 115.5 (q, J = 288.3 Hz), 49.9, 41.8, 28.0; ^{19}F NMR (377 MHz, $CDCl_3$) δ –76.1 (s, 3F); HRMS (ESI^{+}) *m/z* calcd for $C_{15}H_{15}F_3NO_2$ [M+H]$^{+}$: 298.10494, found 298.10443.

Synthesis of indenones imidoyl halides **5**

General procedure:

Under air atmosphere, a 10 mL screw-cap glass reaction tube was charged with $AlCl_3$ (1.05 equiv.) and a stirring bar, then 0.1M solution of the starting triazole **3** (0.131–0.200 mmol) in DCE (1.31–2.00 ml) was added. The reaction mixture was heated to 60 °C and stirred for 1-2 h. The conversion was monitored by ^{19}F NMR spectroscopy. The dark reaction mixture was filtered via paper, washed with Et_2O, evaporated, and purified by column chromatography (silica gel, pentane/DCM) to obtain pure product.

Preparation and characterization of indenones imidoyl halides **5**

*(Z)-2,2,2-trifluoro-N-(3-(2-methoxyphenyl)-1-oxo-1H-inden-2-yl)acetimidoyl chloride (**5a**):*

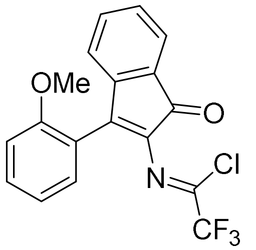

Prepared according to the general procedure from **3g** (58.0 mg, 0.146 mmol). Yield: 5.5 mg, 10%, orange solid; eluent: 1:3 pentane/DCM. ^{1}H NMR (401 MHz, $CDCl_3$) δ 7.52 (ddd, *J* = 7.1, 1.3, 0.7 Hz, 1H), 7.46 (ddd, *J* = 8.3, 7.1, 1.7 Hz, 1H), 7.39 (dd, *J* = 7.6, 1.3 Hz, 1H), 7.35 (ddd, *J* = 7.8, 7.1, 1.3 Hz, 1H), 7.26 – 7.22 (m, 1H), 7.09 (dt, *J* = 7.3, 0.9 Hz, 1H), 7.07 (td, *J* = 7.7, 1.1 Hz, 1H), 7.01 (dd, *J* = 8.4, 1.1 Hz, 1H), 3.79 (s, 3H); ^{13}C NMR (151 MHz, $CDCl_3$) δ 187.8, 157.6, 144.2, 142.9, 136.3 (q, *J* = 43.1 Hz), 135.5, 134.0, 131.9, 129.6, 129.1, 129.0, 123.6, 122.1, 120.6, 119.4, 116.8 (q, *J* = 277.0 Hz), 111.6, 55.4; ^{19}F NMR (377 MHz, $CDCl_3$) δ –71.5 (s, 3F); HRMS (ESI$^+$) *m/z* calcd for $C_{18}H_{12}F_3ClNO_2$ $[M+H]^+$: 366.05032, found 366.05005.

*(Z)-2,2,2-trifluoro-N-(1-oxo-3-phenyl-1H-inden-2-yl)acetimidoyl chloride (**5b**):*

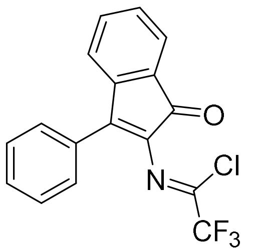

Prepared according to the general procedure from **3a** (53.5 mg, 0.146 mmol). Yield: 10.0 mg, 19%, orange solid; eluent: 3:1 pentane/DCM. ^{1}H NMR (401 MHz, $CDCl_3$) δ 7.58 – 7.55 (m, 1H), 7.53 – 7.48 (m, 5H), 7.41 (td, *J* = 7.5, 1.3 Hz, 1H), 7.34 (dt, *J* = 7.5, 1.0 Hz, 1H), 7.32 – 7.28 (m, 1H); ^{13}C NMR (101 MHz, $CDCl_3$) δ 187.5, 145.4, 143.3, 137.2 (q, *J* = 43.3 Hz), 134.2, 134.1, 130.7, 130.6, 129.8, 129.4, 129.0, 128.4, 124.1, 122.2, 116.7 (q, *J* = 277.7 Hz); ^{19}F NMR NMR (471 MHz, $CDCl_3$) δ –71.5 (s, 3F); HRMS (EI$^+$) *m/z* calcd for $C_{17}H_9F_3ClNO$ $[M]^+$: 335.0319, found 335.0322.

*(Z)-N-(4,6-dimethoxy-3-(4-nitrophenyl)-1-oxo-1H-inden-2-yl)-2,2,2-trifluoroacetimidoyl chloride (**5c**):*

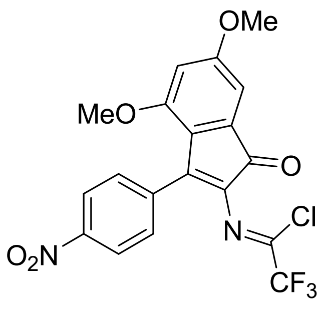

Prepared according to the general procedure from **3w** (87.0 mg, 0.184 mmol). Yield: 24.5 mg, 30%, dark solid; eluent: 3:1 cyclohexane/EtOAc. ^{1}H NMR (500 MHz, $CDCl_3$) δ 8.26 – 8.24 (m, 2H), 7.58 – 7.55 (m, 2H), 6.87 (d, *J* = 2.1 Hz, 1H), 6.47 (d, *J* = 2.1 Hz, 1H), 3.88 (s, 3H), 3.68 (s, 3H); ^{13}C NMR (126 MHz, $CDCl_3$) δ 186.8, 163.6, 155.6, 148.4, 147.4, 138.6, 136.9 (q, *J* = 43.1 Hz), 133.7, 132.6, 129.9, 122.8, 119.4, 116.7 (q, *J* = 277.5 Hz), 104.5, 104.0, 56.2, 55.7; ^{19}F NMR (377 MHz, $CDCl_3$) δ –71.4 (s, 3F); HRMS (APCI$^+$) *m/z* calcd for $C_{19}H_{12}F_3ClN_2O_5$ $[M]^+$: 440.03814, found 440.03805.

*(Z)-N-(4,6-dimethoxy-1-oxo-3-phenyl-1H-inden-2-yl)-2,2,2-trifluoroacetimidoyl chloride (**5d**):*

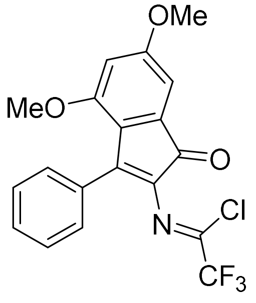

Prepared according to the general procedure from **3u** (85.4 mg, 0.200 mmol). Yield: 38.0 mg, 48%, dark solid; eluent: 1:1 pentane/DCM. ^{1}H NMR (400 MHz, $CDCl_3$) δ 7.41 – 7.38 (m, 5H), 6.85 (d, *J* = 2.2 Hz, 1H), 6.46 (d, *J* = 2.1 Hz, 1H), 3.86 (s, 3H), 3.67 (s, 3H); ^{13}C NMR (101 MHz, $CDCl_3$) δ 187.2, 163.2, 155.7, 150.9, 135.7 (q, *J* = 42.9 Hz), 133.4, 132.8, 131.9, 130.2, 129.0, 127.5, 120.1, 116.9 (q, *J* = 277.1 Hz), 104.1, 104.0, 56.1, 55.7; ^{19}F NMR (376 MHz, $CDCl_3$) δ –71.3 (s, 3F); HRMS (ESI$^+$) *m/z* calcd for $C_{19}H_{14}F_3ClNO_3$ [M+H]$^+$: 396.06088, found 396.06064.

*(Z)-N-(4,6-dimethoxy-3-(4-methoxyphenyl)-1-oxo-1H-inden-2-yl)-2,2,2-trifluoroacetimidoyl chloride (**5e**):*

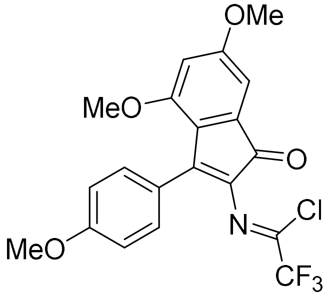

Prepared according to the general procedure from **3x** (60.0 mg, 0.131 mmol). Yield: 31.5 mg, 56%, dark solid; eluent: 3:1 cyclohexane/EtOAc. ^{1}H NMR (500 MHz, $CDCl_3$) δ 7.41 – 7.38 (m, 2H), 6.93 – 6.90 (m, 2H), 6.84 (d, *J* = 2.1 Hz, 1H), 6.46 (d, *J* = 2.1 Hz, 1H), 3.87 (s, 3H), 3.86 (s, 3H), 3.70 (s, 3H); ^{13}C NMR (126 MHz, $CDCl_3$) δ 186.8, 163.3, 161.4, 155.7, 151.9, 135.2 (q, *J* = 42.7 Hz), 134.0, 131.9, 131.3, 124.2, 119.8, 117.0 (q, *J* = 277.0 Hz), 112.9, 104.0, 103.7, 56.1, 55.7, 55.5; ^{19}F NMR (377 MHz, $CDCl_3$) δ –71.1 (s, 3F); HRMS (ESI$^+$) *m/z* calcd for $C_{20}H_{16}F_3ClNO_4$ [M+H]$^+$: 426.07145, found 426.07180.

Synthesis of oxazoles **6**

General procedure:

Under air atmosphere, a 10 mL screw-cap glass reaction tube was charged with 0.1M solution of the starting triazole **3** (0.084–0.132 mmol) in DCE (0.84–1.32 ml) and a stirring bar. Then, $BF_3{\cdot}OEt_2$ (1.05 equiv.) was added. The reaction mixture was heated to 60 °C and stirred for 1-2 h. The conversion was monitored by ^{19}F NMR spectroscopy. The dark reaction mixture was filtered via paper, washed with Et_2O, evaporated with Celite, and purified by flash column chromatography (silica gel, cyclohexane/EtOAc) to obtain pure product.

Preparation and characterization of oxazoles **6**

*phenyl(5-phenyl-2-(trifluoromethyl)oxazol-4-yl)methanone (**6a**):*

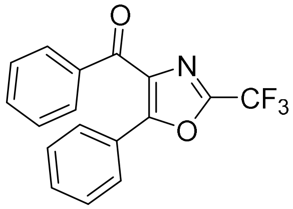

Prepared according to the general procedure from **3a** (42.3 mg, 0.115 mmol). Yield: 39.0 mg, 76%, white solid; eluent: 4:1 cyclohexane/EtOAc. ^{1}H NMR (400 MHz, $CDCl_3$) δ 8.11 – 8.07 (m, 2H), 8.05 – 8.01 (m, 2H), 7.64 – 7.59 (m, 1H), 7.53 – 7.47 (m, 5H); ^{13}C NMR (101 MHz, $CDCl_3$) δ 187.5, 157.0, 147.9 (q, *J* = 44.7 Hz), 136.7, 134.0, 133.8, 131.6, 130.6, 129.0, 128.6, 128.4, 125.9, 116.5 (q, *J* = 271.1 Hz); ^{19}F NMR (376 MHz, $CDCl_3$) δ –66.1 (s, 3F); HRMS (EI) *m/z* calcd for $C_{17}H_{10}F_3NO_2$ $[M]^+$: 316.0580, found 316.0576.

*(5-(4-methoxyphenyl)-2-(trifluoromethyl)oxazol-4-yl)(phenyl)methanone (**6b**):*

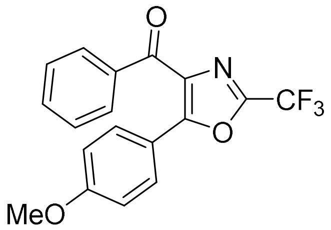

Prepared according to the general procedure from **3y** (33.5 mg, 0.084 mmol). Yield: 22.0 mg, 75%, pale orange solid; eluent: 4:1 cyclohexane/EtOAc. ^{1}H NMR (400 MHz, $CDCl_3$) δ 8.10 – 8.04 (m, 4H), 7.63 – 7.58 (m, 1H), 7.52 – 7.47 (m, 2H), 7.01 – 6.97 (m, 2H), 3.88 (s, 3H); ^{13}C NMR (101 MHz, $CDCl_3$) δ 187.4, 162.2, 157.4, 147.0 (q, *J* = 44.8 Hz), 136.9, 133.4, 132.6, 130.4, 130.2, 128.4, 118.2, 116.4 (q, *J* = 271.2 Hz), 114.2, 55.5; ^{19}F NMR (376 MHz, $CDCl_3$) δ –66.1 (s, 3F); HRMS (EI) *m/z* calcd for $C_{18}H_{12}F_3NO_3$ $[M]^+$: 347.0764, found 347.0763.

(5-(4-chlorophenyl)-2-(trifluoromethyl)oxazol-4-yl)(phenyl)methanone (mixture of izomers **A**/**B** 84:16) ***(6c):***

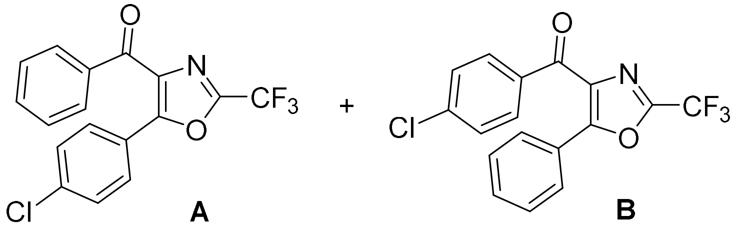

Prepared according to the general procedure from **3z** (33.9 mg, 0.084 mmol). Yield: 24.5 mg, 83%, colorless oil; eluent: 4:1 cyclohexane/EtOAc. ^{1}H NMR (401 MHz, $CDCl_3$) δ 8.11 – 8.02 (m, 4H), 7.65 – 7.60 (m, 1H), 7.53 – 7.47 (m, 4H); ^{13}C NMR (101 MHz, $CDCl_3$) δ 187.3, 156.1, 148.0 (q, *J* = 44.7 Hz), 137.9, 136.6, 133.9, 132.0, 130.6, 129.7, 129.3, 128.6, 124.3, 116.4 (q, *J* = 271.4 Hz); ^{19}F NMR (377 MHz, $CDCl_3$) δ –66.0 (s, 3F); HRMS (EI) *m/z* calcd for $C_{17}H_9F_3ClNO_2$ $[M]^+$: 351.0268, found 351.0263.

phenyl(5-(thiophen-2-yl)-2-(trifluoromethyl)oxazol-4-yl)methanone (mixture of izomers A/B 90:10) ***(6d):***

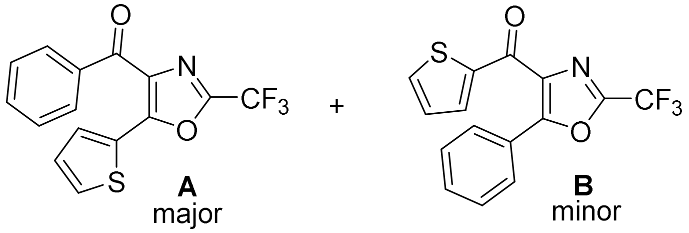

Prepared according to the general procedure from **3ab** (44.4 mg, 0.119 mmol). Yield: 20.2 mg, 53%, orange solid; eluent: 3:1 cyclohexane/EtOAc. ^{1}H NMR (401 MHz, $CDCl_3$) δ 8.24 – 8.21 (m, 2H), 8.19 (dd, *J* = 3.9, 1.3 Hz, 1H), 7.66 – 7.60 (m, 2H), 7.55 – 7.50 (m, 2H), 7.22 (dd, *J* = 5.0, 3.9 Hz, 1H); ^{13}C NMR (101 MHz, $CDCl_3$) δ 186.5, 154.0, 146.7 (q, *J* = 44.7 Hz), 136.8, 133.5, 132.03, 131.97, 131.9, 130.6, 128.5,

128.2, 127.4, 116.4 (q, J = 271.1 Hz); ^{19}F NMR (377 MHz, $CDCl_3$) δ –66.0 (s, 3F); HRMS (ESI$^+$) *m/z* calcd for $C_{15}H_9F_3NO_2S$ [M+H]$^+$: 324.03006, found 324.02985.

(5-isobutyl-2-(trifluoromethyl)oxazol-4-yl)(phenyl)methanone (mixture of izomers A/B 70:30) ***(6e):***

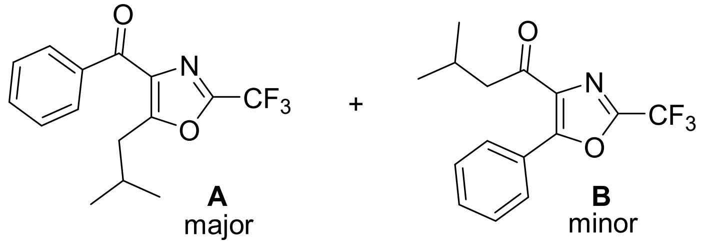

Prepared according to the general procedure from **3r** (30.4 mg, 0.088 mmol). Yield: 7.0 mg, 27%, colorless oil; (**6e** major A): eluent: 4:1 cyclohexane/EtOAc. ^{1}H NMR (401 MHz, $CDCl_3$) δ 8.23 – 8.18 (m, 2H), 7.63 – 7.59 (m, 1H), 7.53 – 7.48 (m, 2H), 3.07 (d, J = 7.2 Hz, 2H), 2.20 (hept, J = 6.7 Hz, 1H), 1.01 (d, J = 6.7 Hz, 6H); ^{13}C NMR (126 MHz, $CDCl_3$) δ 186.9, 162.8, 147.7 (q, J = 44.6 Hz), 136.7, 133.5, 130.4, 128.7, 128.5, 116.5 (q, J = 271.1 Hz), 35.2, 28.3, 22.5, ^{19}F NMR (376 MHz, $CDCl_3$) δ –66.3 (s, 3F); (**6e** minor B): eluent: 4:1 cyclohexane/EtOAc. ^{1}H NMR (401 MHz, $CDCl_3$) δ 8.23 – 8.18 (m, 2H), 7.53 – 7.48 (m, 3H), 2.97 (d, J = 7.0 Hz, 2H), 2.31 (hept, J = 6.7 Hz, 1H), 1.01 (d, J = 6.7 Hz, 6H); ^{13}C NMR (126 MHz, $CDCl_3$) δ 195.6, 155.5, 147.7 (q, J = 44.7 Hz), 135.3, 134.4, 131.8, 128.8, 125.8, 116.4 (q, J = 270.7 Hz), 49.7, 24.7, 22.8, ^{19}F NMR (376 MHz, $CDCl_3$) δ –66.3 (s, 3F); HRMS (ESI+) *m/z* calcd for $C_{15}H_{15}F_3NO_2$ [M+H]$^+$: 298.10494, found 298.10461.

(2-methoxyphenyl)(5-phenyl-2-(trifluoromethyl)oxazol-4-yl)methanone (***6f***)*:*

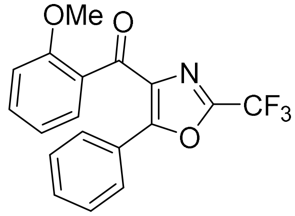

Prepared according to the general procedure with corresponding triazole **3g** (39.0 mg, 0.098 mmol). Yield: 4.0 mg, 12%, colorless oil; eluent: 3:1 cyclohexane/EtOAc. ^{1}H NMR (401 MHz, $CDCl_3$) δ 8.08 – 8.05 (m, 2H), 7.58 (dd, J = 7.6, 1.6 Hz, 1H), 7.52 – 7.44 (m, 4H), 7.05 (td, J = 7.5, 0.9 Hz, 1H), 6.93 (dd, J = 8.4, 1.0 Hz, 1H), 3.71 (s, 3H); ^{13}C NMR (101 MHz, $CDCl_3$) δ 188.7, 158.7, 155.7, 147.8 (q, J = 44.4 Hz), 135.5, 133.9, 131.5, 130.8, 128.8, 128.4, 128.2, 126.1, 120.9, 116.5 (q, J = 271.1 Hz), 111.9, 55.8; ^{19}F NMR (377 MHz, $CDCl_3$) δ –66.1 (s, 3F); HRMS (EI) *m/z* calcd for $C_{18}H_{12}F_3NO_3$ [M]$^+$: 347.0764, found 347.0768.

2,2,2-trifluoro-N-(3-(2-methoxyphenyl)-1-oxo-1H-inden-2-yl)acetamide (***5f***)*:*

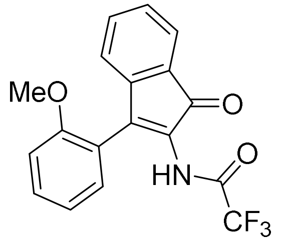

Prepared as the mixture with **6f** according to the general procedure B from **3g** (39.0 mg, 0.098 mmol). Yield: 9.1 mg, 32%, orange solid; eluent: 3:1 cyclohexane/EtOAc. ^{1}H NMR (401 MHz, $CDCl_3$) δ 8.15 (bs, 1H), 7.55 – 7.48 (m, 3H), 7.35 (td, J = 7.6, 1.3 Hz, 1H), 7.23 (ddd, J = 7.8, 7.0, 1.0 Hz, 1H), 7.16 – 7.11 (m, 2H), 7.03 (dd, J = 8.4, 1.2 Hz, 1H), 3.86 (s, 3H); ^{13}C NMR (126 MHz, $CDCl_3$) δ 190.9, 157.1, 153.6 (q, J = 37.7 Hz), 144.7, 141.3, 134.3, 131.9, 128.9, 128.9, 128.7, 126.5, 123.7, 122.3, 120.7, 120.6, 115.8 (q, J = 288.4 Hz), 111.3, 55.6; ^{19}F NMR (377 MHz, $CDCl_3$) δ –75.7 (s, 3F); HRMS (EI) *m/z* calcd for $C_{18}H_{12}F_3NO_3$ [M]$^+$: 347.0764, found 347.0754.

*4-benzoyl-5-phenyloxazol-2(3H)-one (**6g**):*

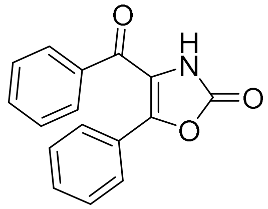

Prepared according to the general procedure with corresponding triazole **3m** (42.0 mg, 0.132 mmol). Yield: 11.3 mg, 32%, orange oil; eluent: 4:1 cyclohexane/EtOAc. ^{1}H NMR (400 MHz, $CDCl_3$) δ 8.61 (bs, 1H), 7.69 – 7.60 (m, 2H), 7.51 – 7.43 (m, 1H), 7.35 – 7.23 (m, 5H), 7.23 – 7.14 (m, 2H); ^{13}C NMR (101 MHz, $CDCl_3$) δ 185.1, 153.0, 146.6, 135.9, 133.5, 130.6, 129.4, 128.9, 128.6, 128.4, 126.4, 120.9; HRMS (ESI$^+$) *m/z* calcd for $C_{16}H_{12}NO_3$ $[M+H]^+$: 266.08117, found 266.08123.

Synthesis of *N*-alkenyl compounds **7**

*5-chloro-5-methyl-3-phenyl-2-(5-(trifluoromethyl)-1H-tetrazol-1-yl)cyclopent-2-en-1-one (**7a**):*

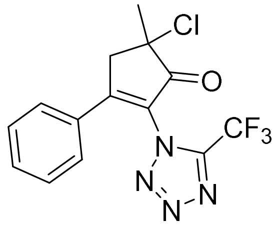

Under air atmosphere, a 10 mL screw-cap glass reaction tube was charged with cyclopentenone imidoyl halide **4a** (32.4 mg, 0.096 mmol), MeCN (1.4 mL) and sodium azide (13.8 mg, 0.212 mmol, 2.2 equiv.) was added. The reaction mixture was stirred for 1 h at 25 °C. Then the reaction suspension was filtered via paper, washed with Et_2O and extracted with Et_2O, water and brine. The organic layer was dried over anhydrous $MgSO_4$, filtered via paper, evaporated with Celite and purified by flash column chromatography (silica gel, cyclohexane/EtOAc) to obtain pure product. Yield: 20.0 mg, 61%, colorless oil; eluent: 1:2 pentane/DCM. ^{1}H NMR (401 MHz, $CDCl_3$) δ 7.58 – 7.54 (m, 1H), 7.44 – 7.39 (m, 2H), 7.16 – 7.12 (m, 2H), 3.33 (d, J = 18.6 Hz, 1H), 3.26 (d, J = 18.5 Hz, 1H), 1.74 (s, 3H); ^{13}C NMR (101 MHz, $CDCl_3$) δ 196.6, 168.2, 147.2 (q, J = 42.7 Hz), 134.1, 129.9, 129.7, 128.1, 125.5, 117.6 (q, J = 272.7 Hz), 61.7, 43.1, 21.0; ^{19}F NMR (377 MHz, $CDCl_3$) δ –62.2 (s, 3F); HRMS (ESI$^+$) *m/z* calcd for $C_{14}H_{10}F_3ClN_4ONa$ $[M+Na]^+$: 365.03874, found 365.03866.

*(Z)-N'-(4-chloro-4-methyl-5-oxo-2-phenylcyclopent-1-en-1-yl)-2,2,2-trifluoroacetimidamide (**7b**):*

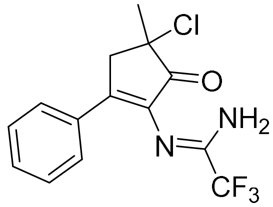

Under air atmosphere, a 10 mL screw-cap glass reaction tube was charged with cyclopentenone imidoyl halide **4a** (35.6 mg, 0.106 mmol), DCE (1.5 mL) and excess of NH_4OH (29% solution in water, 147 µl) was added. The reaction mixture was stirred for 1 h at 25 °C. Then the reaction mixture was filtered via paper, washed with Et_2O and extracted with Et_2O, water and brine. The organic layer was dried over anhydrous $MgSO_4$, filtered via paper, evaporated with Celite and purified by flash column chromatography (silica gel, cyclohexane/EtOAc) to obtain pure product. Yield: 23.5 mg, 70%, white solid; eluent: 3:1 cyclohexane/EtOAc. ^{1}H NMR (401 MHz, $CDCl_3$) δ 8.00 – 7.97 (m, 2H), 7.48 – 7.44 (m, 3H), 5.80 – 5.75 (bs, 2H), 3.60 (d, J = 18.5 Hz, 1H), 3.39 (d, J = 18.3 Hz, 1H), 1.80 (s, 3H); ^{13}C NMR (101 MHz, $CDCl_3$) δ 198.9, 153.1, 145.1 (q, J = 35.8 Hz), 137.2, 133.4, 131.3, 129.0, 128.7, 118.2 (q, J = 278.8 Hz),

63.3, 46.0, 26.9; ^{19}F NMR (377 MHz, $CDCl_3$) δ –73.2 (s, 3F); HRMS (EI) *m/z* calcd for $C_{14}H_{12}F_3ClN_2O$ $[M]^+$: 316.0585, found 316.0583.

*7-methyl-5-phenyl-3-(trifluoromethyl)-6,7-dihydro-2H-cyclopenta[e][1,2,4]triazin-7-ol (**7c**):*

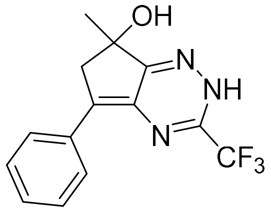

Under air atmosphere, a 10 mL screw-cap glass reaction tube was charged with cyclopentenone imidoyl halide **4a** (53.0 mg, 0.158 mmol), MeCN (1.6 mL) and hydrazine hydrate (19.7 mg, 0.394 mmol, 2.5 equiv.) was added. The reaction mixture was stirred for 1 h at 25 °C. Then the reaction suspension was filtered via paper, washed with Et_2O and extracted with Et_2O, water and brine. The organic layer was dried over anhydrous $MgSO_4$, filtered via paper, evaporated with Celite and purified by flash column chromatography (silica gel, cyclohexane/EtOAc) to obtain pure product. Yield: 24.0 mg, 52%, white solid; eluent: 3:1 cyclohexane/EtOAc. ^{1}H NMR (400 MHz, Acetone-*d*$_6$) δ 9.20 (bs, 1H), 8.61 (bs, 1H), 7.31 – 7.22 (m, 5H), 2.89 (dq, *J* = 16.8, 1.2 Hz, 1H), 2.76 (dq, *J* = 16.9, 2.0 Hz, 1H), 1.98 (dd, *J* = 2.0, 1.3 Hz, 3H); ^{13}C NMR (101 MHz, Acetone-*d*$_6$) δ 162.7, 147.8, 141.1, 138.7 (q, *J* = 35.9 Hz), 133.9, 129.2, 128.7, 126.3, 121.7 (q, *J* = 270.0 Hz), 53.0, 48.3, 13.4; ^{19}F NMR (376 MHz, Acetone-*d*$_6$) δ –69.4 (s, 3F); HRMS (ESI^+) *m/z* calcd for $C_{14}H_{13}F_3N_3O$ $[M+H]^+$: 296.10052, found 296.10057.

Copies of NMR spectra

Figure S1. ^{1}H NMR spectrum of **3a** ($CDCl_3$, 401 MHz)

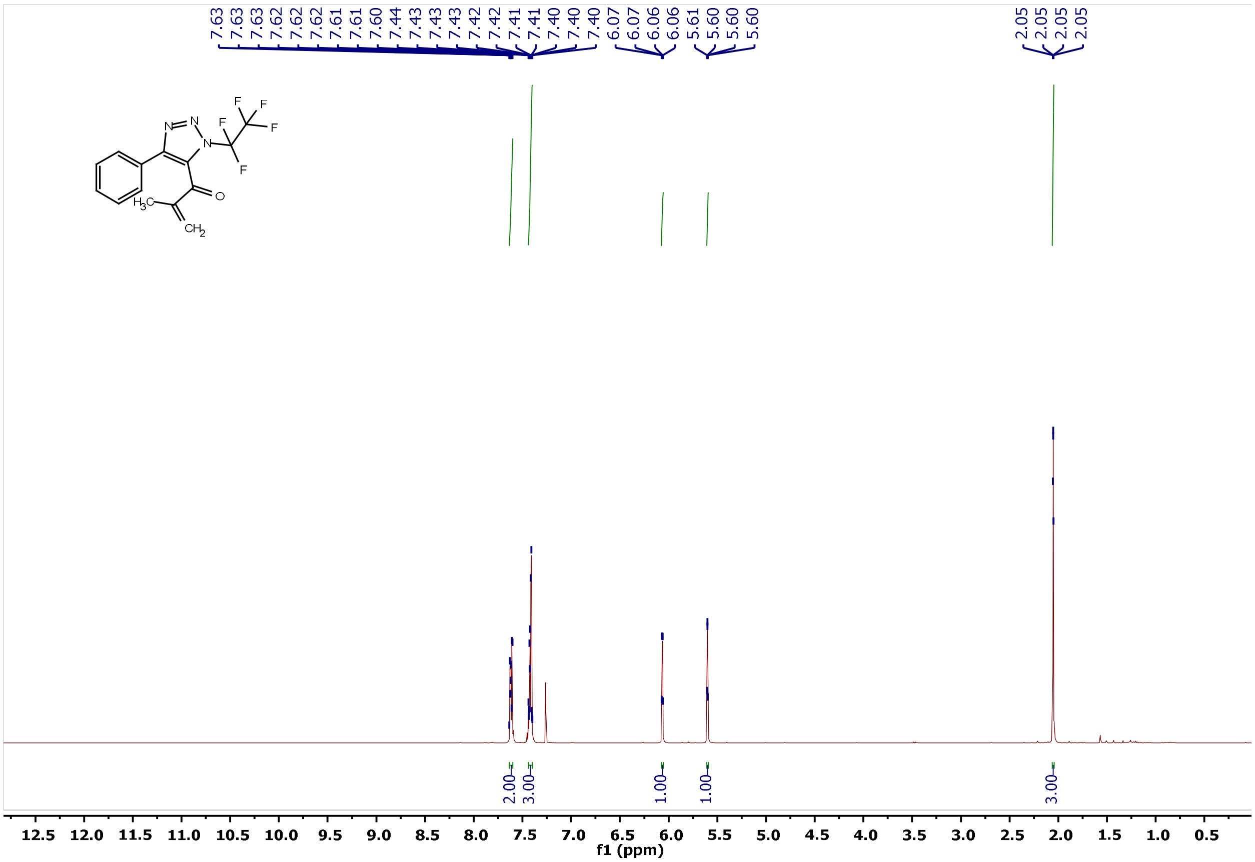

Figure S2. ^{13}C NMR spectrum of **3a** ($CDCl_3$, 101 MHz)

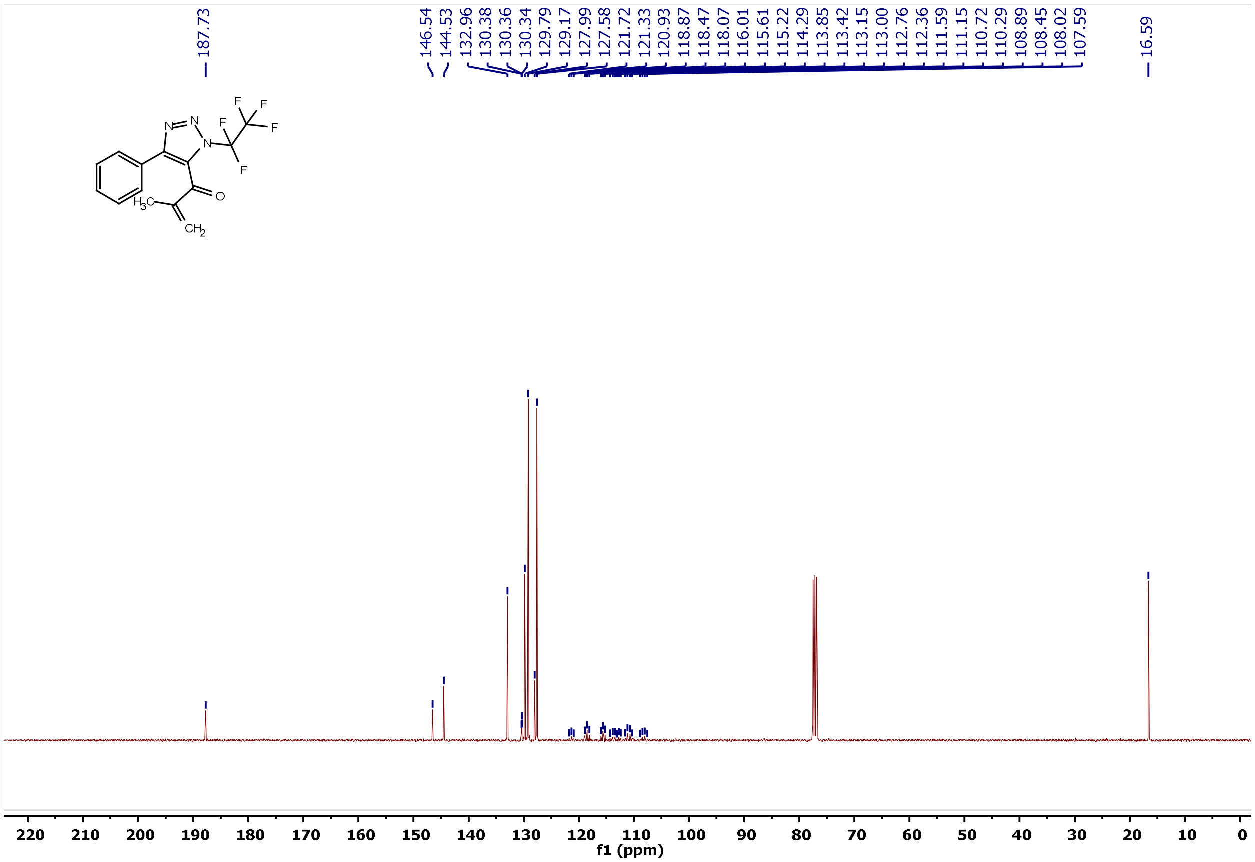

Figure S3. ^{19}F NMR spectrum of **3a** ($CDCl_3$, 377 MHz)

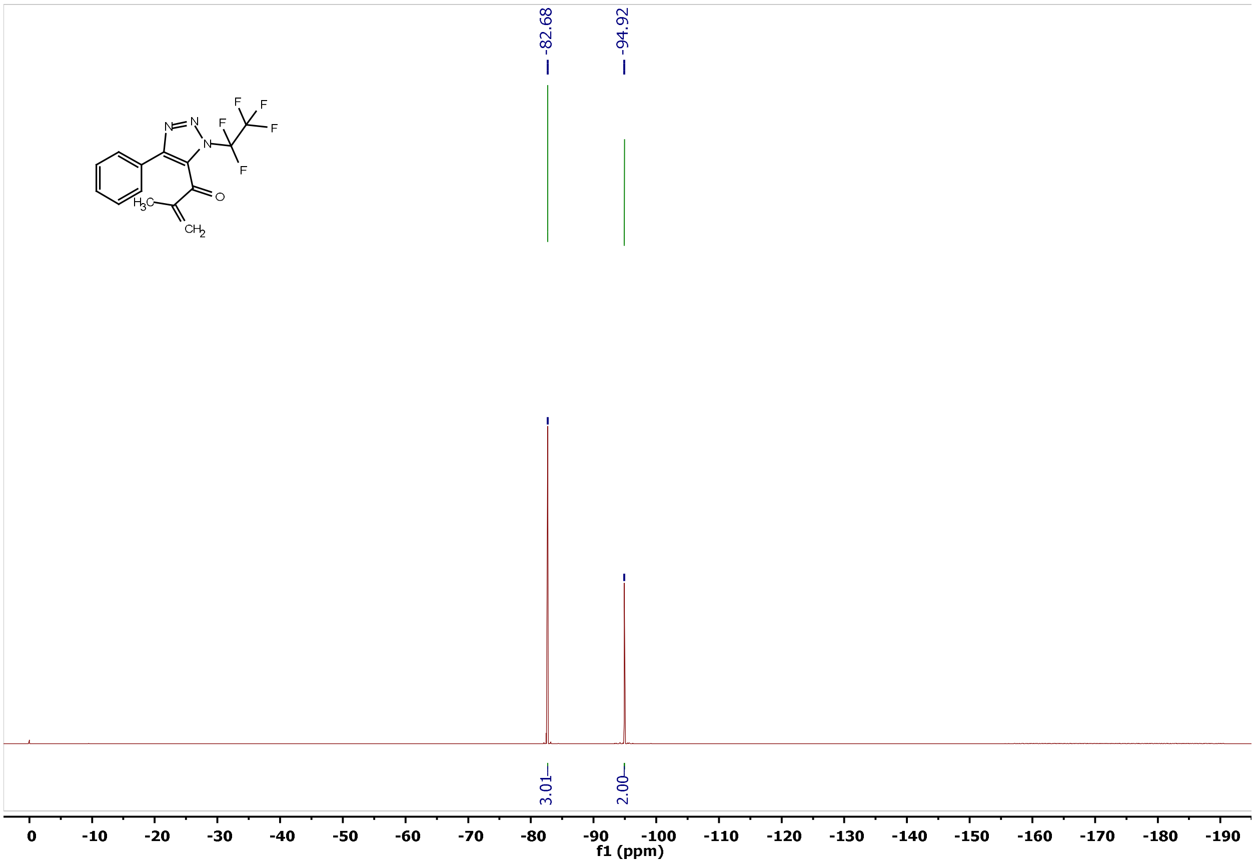

Figure S4. ^{1}H NMR spectrum of **3b** ($CDCl_3$, 401 MHz)

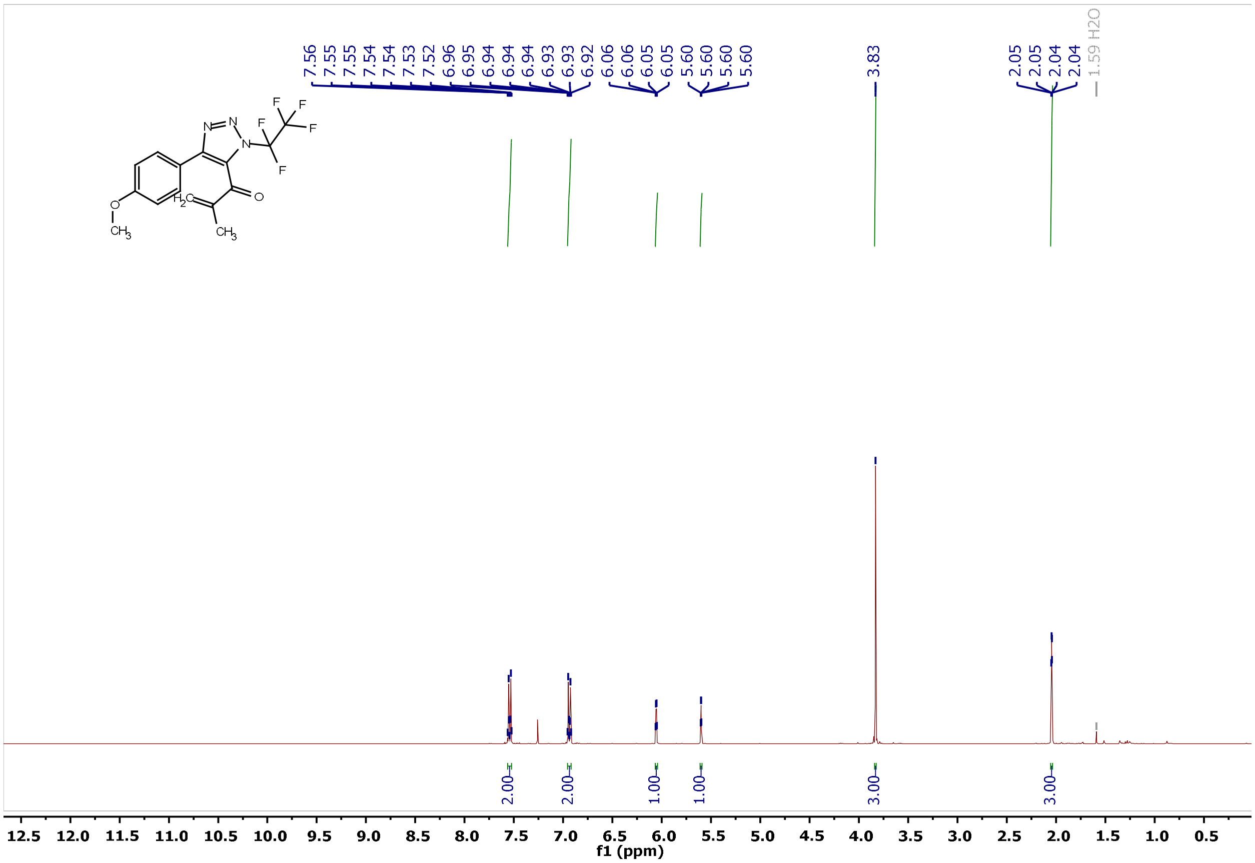

Figure S5. ^{13}C NMR spectrum of **3b** ($CDCl_3$, 101 MHz)

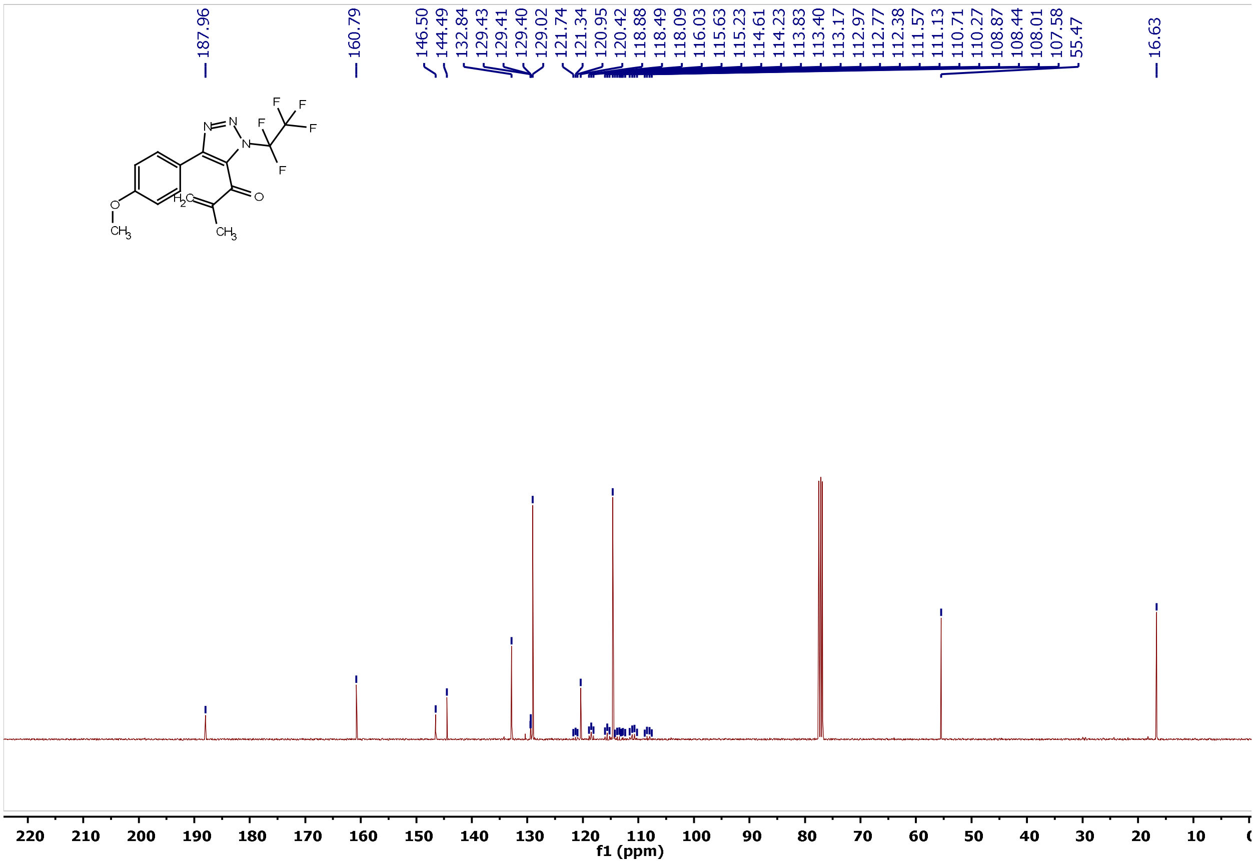

Figure S6. ^{19}F NMR spectrum of **3b** ($CDCl_3$, 377 MHz)

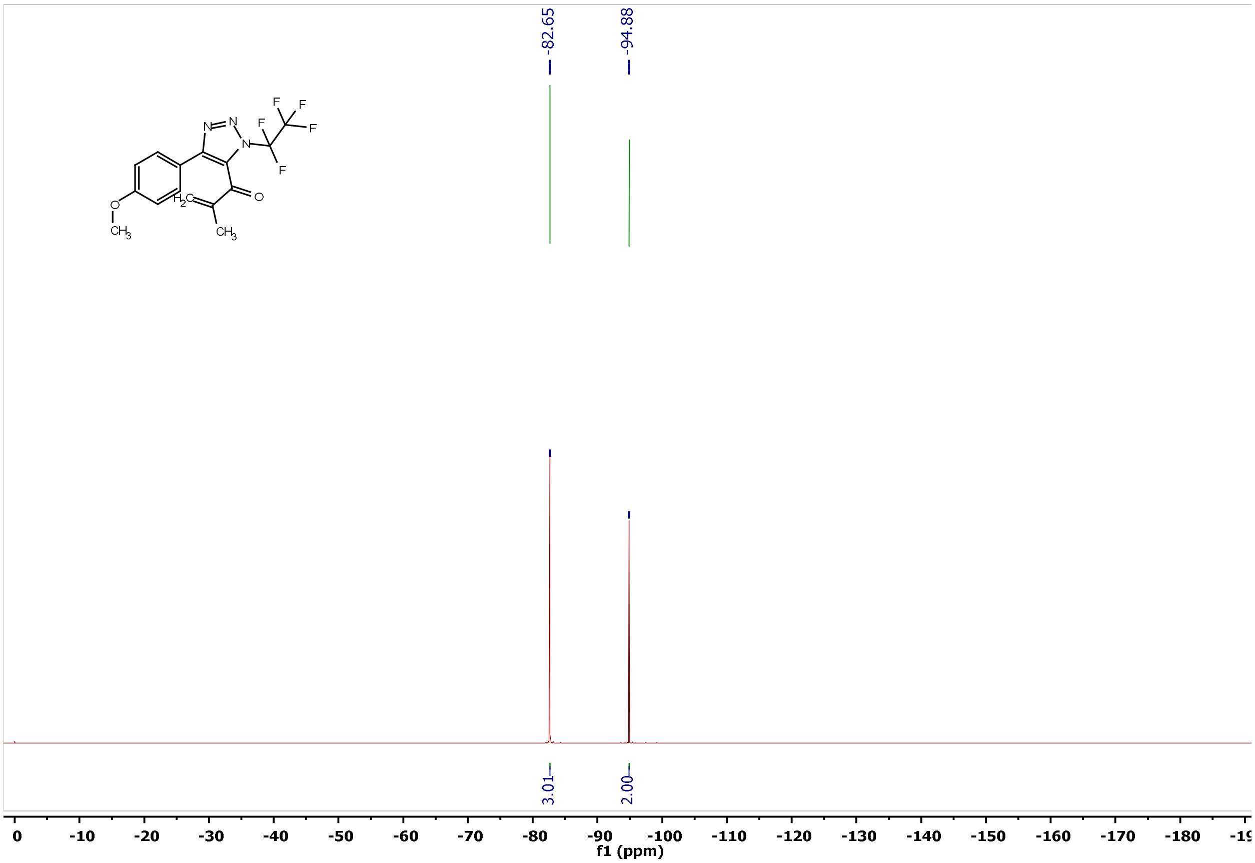

Figure S7. ^{1}H NMR spectrum of **3c** ($CDCl_3$, 401 MHz)

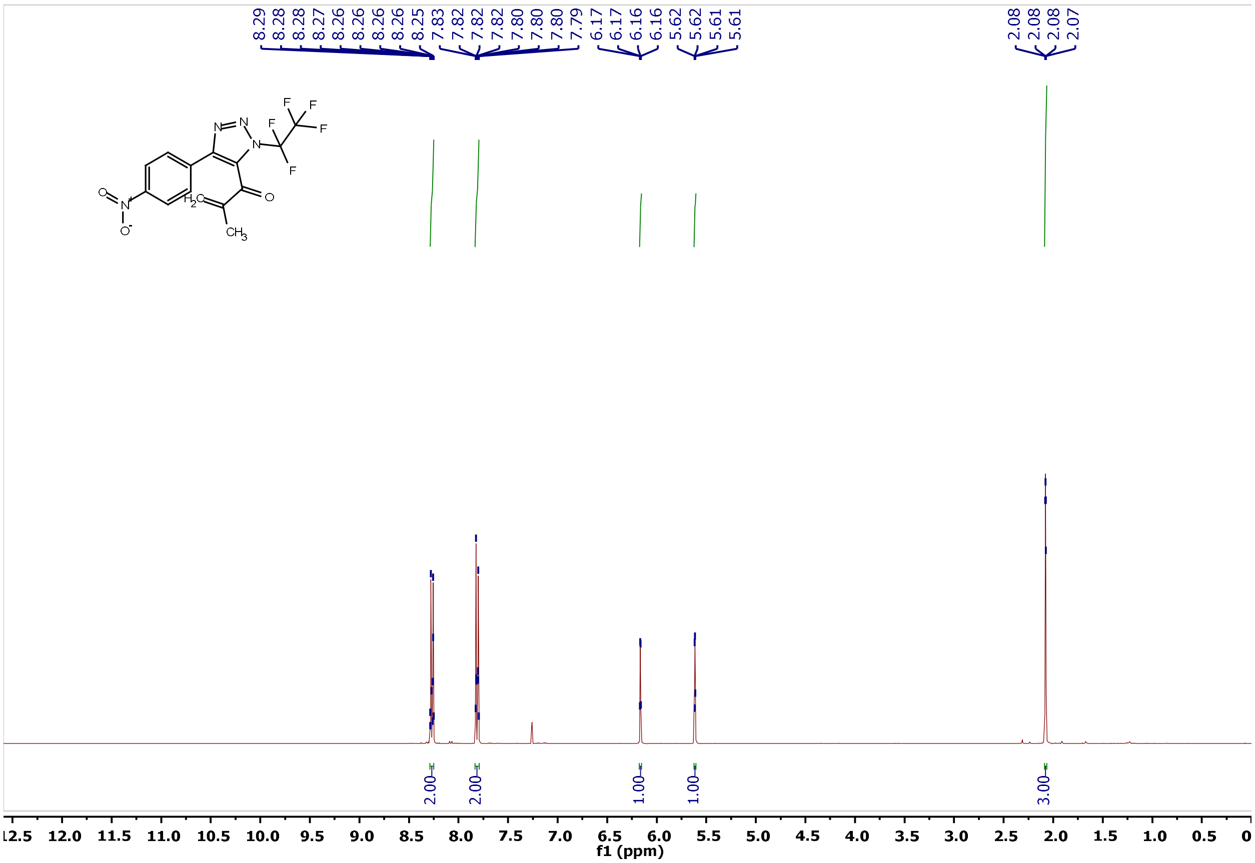

Figure S8. ^{13}C NMR spectrum of **3c** ($CDCl_3$, 101 MHz)

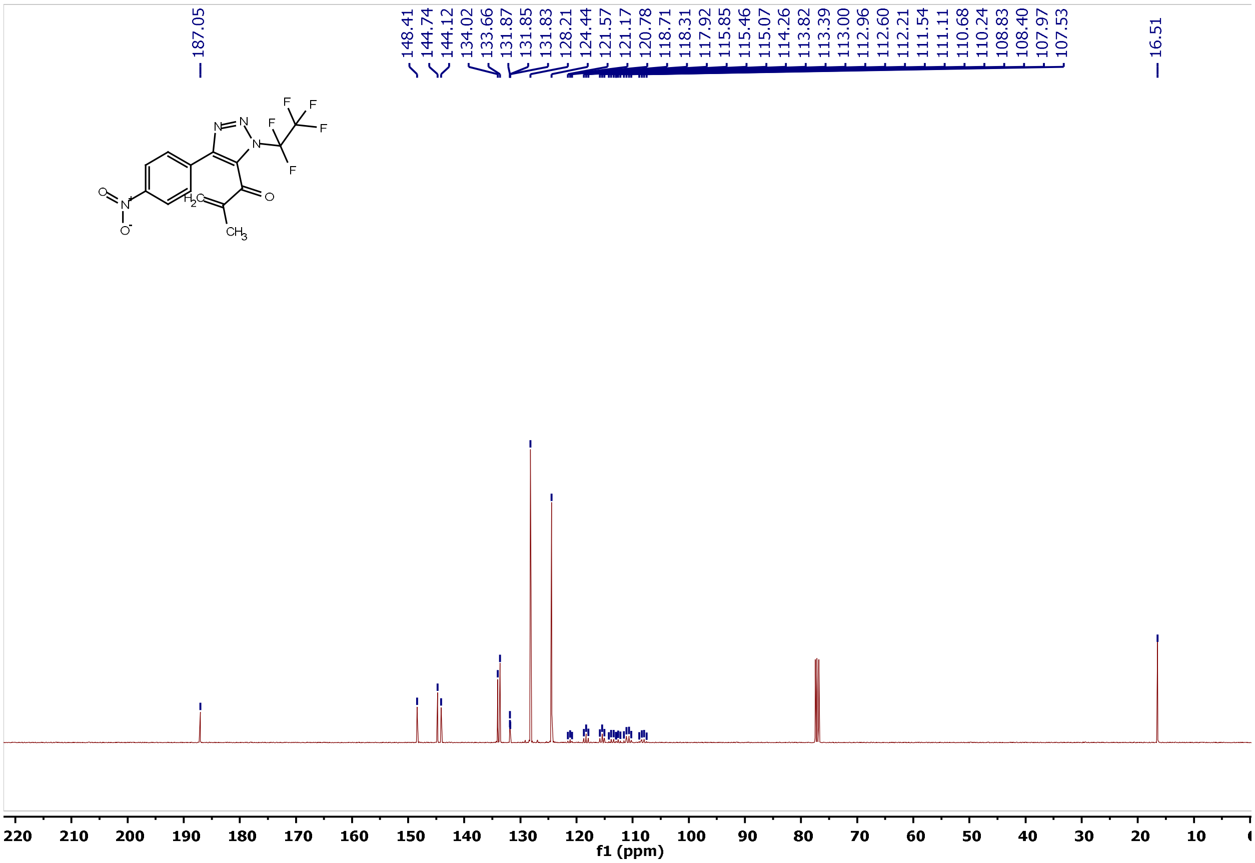

Figure S9. ^{19}F NMR spectrum of **3c** ($CDCl_3$, 377 MHz)

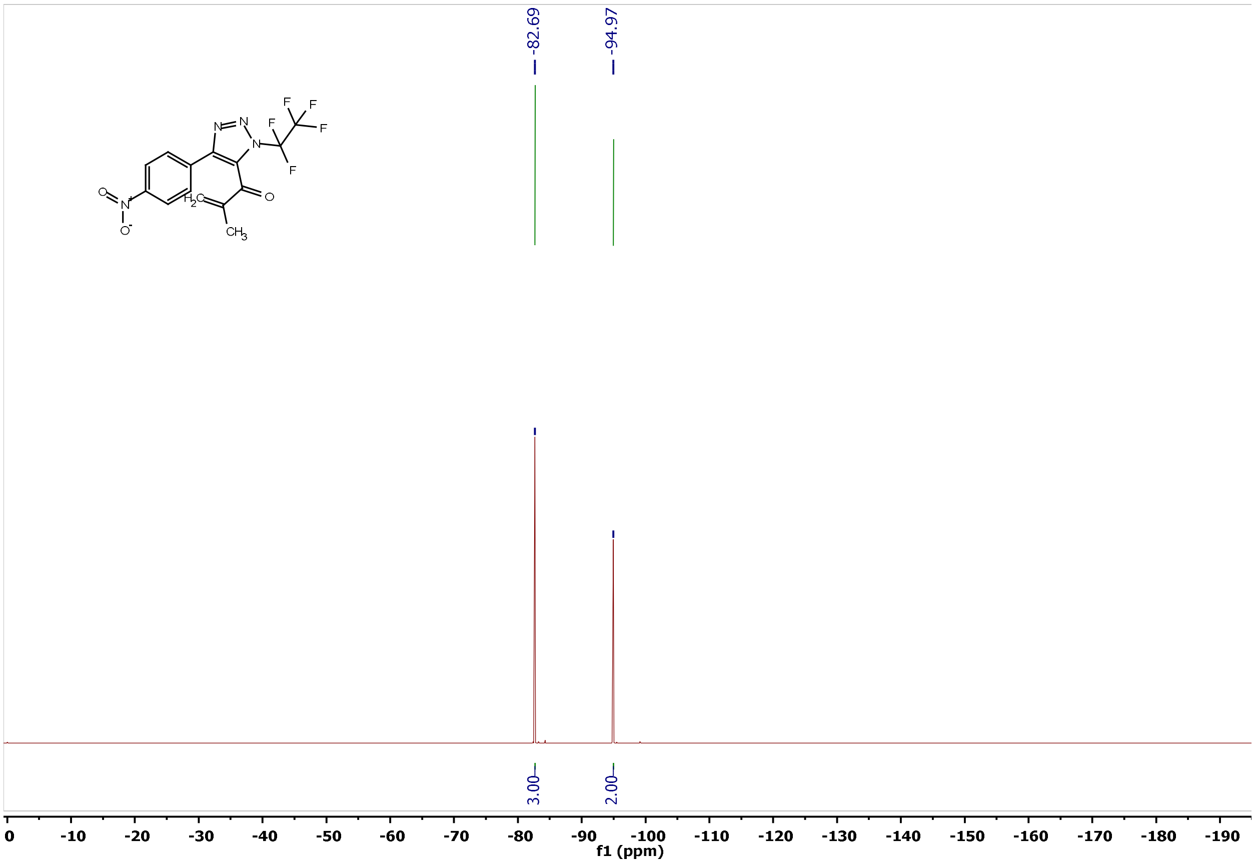

Figure S10. ^{1}H NMR spectrum of **3d** ($CDCl_3$, 401 MHz)

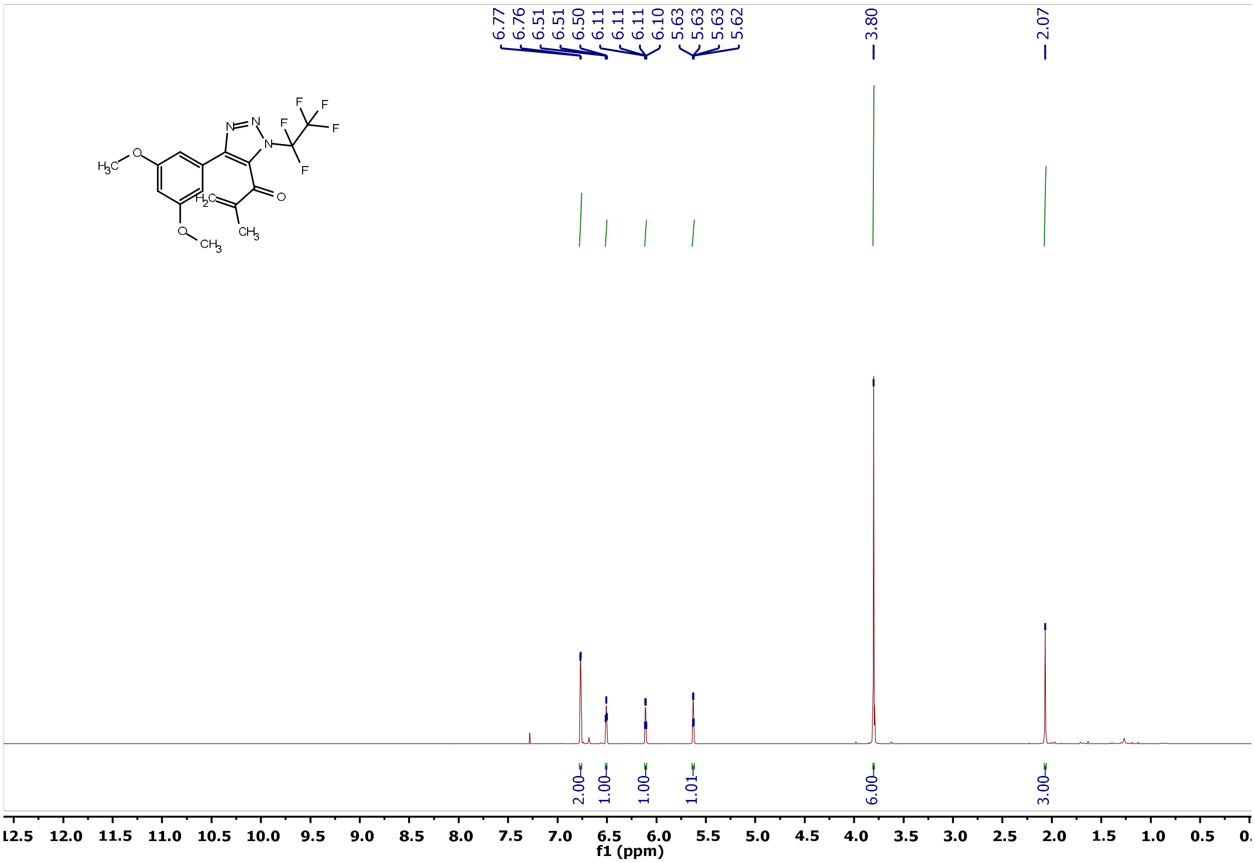

Figure S11. ^{13}C NMR spectrum of **3d** ($CDCl_3$, 101 MHz)

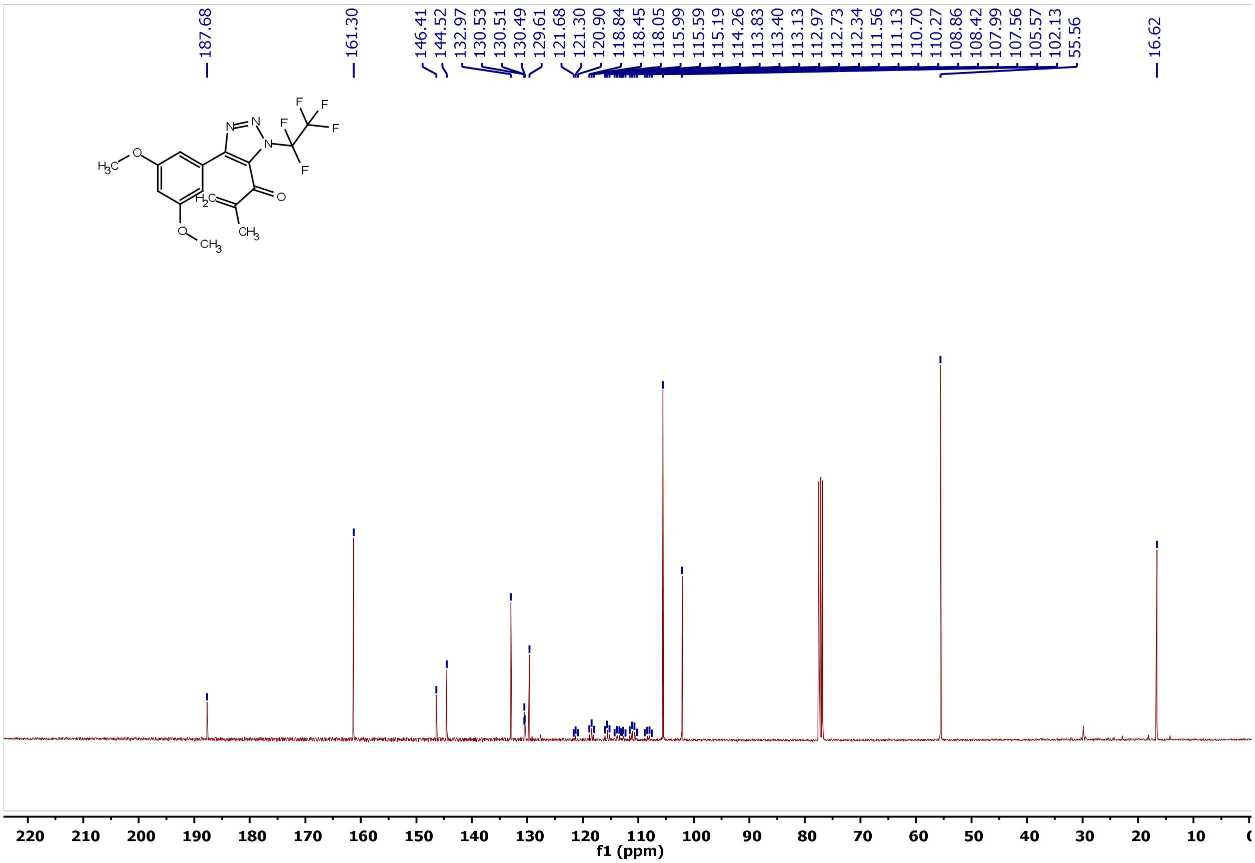

Figure S12. ^{19}F NMR spectrum of **3d** ($CDCl_3$, 377 MHz)

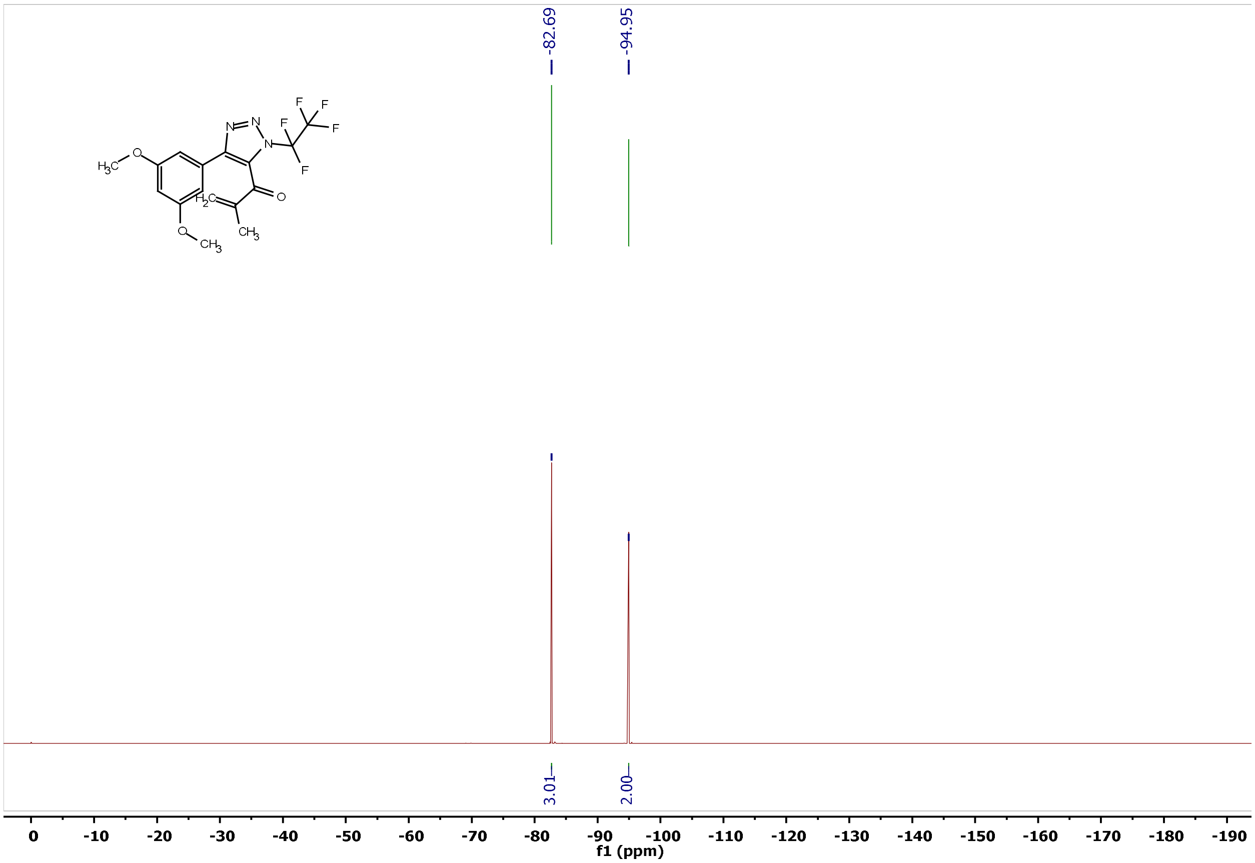

Figure S13. ^{1}H NMR spectrum of **3e** ($CDCl_3$, 401 MHz)

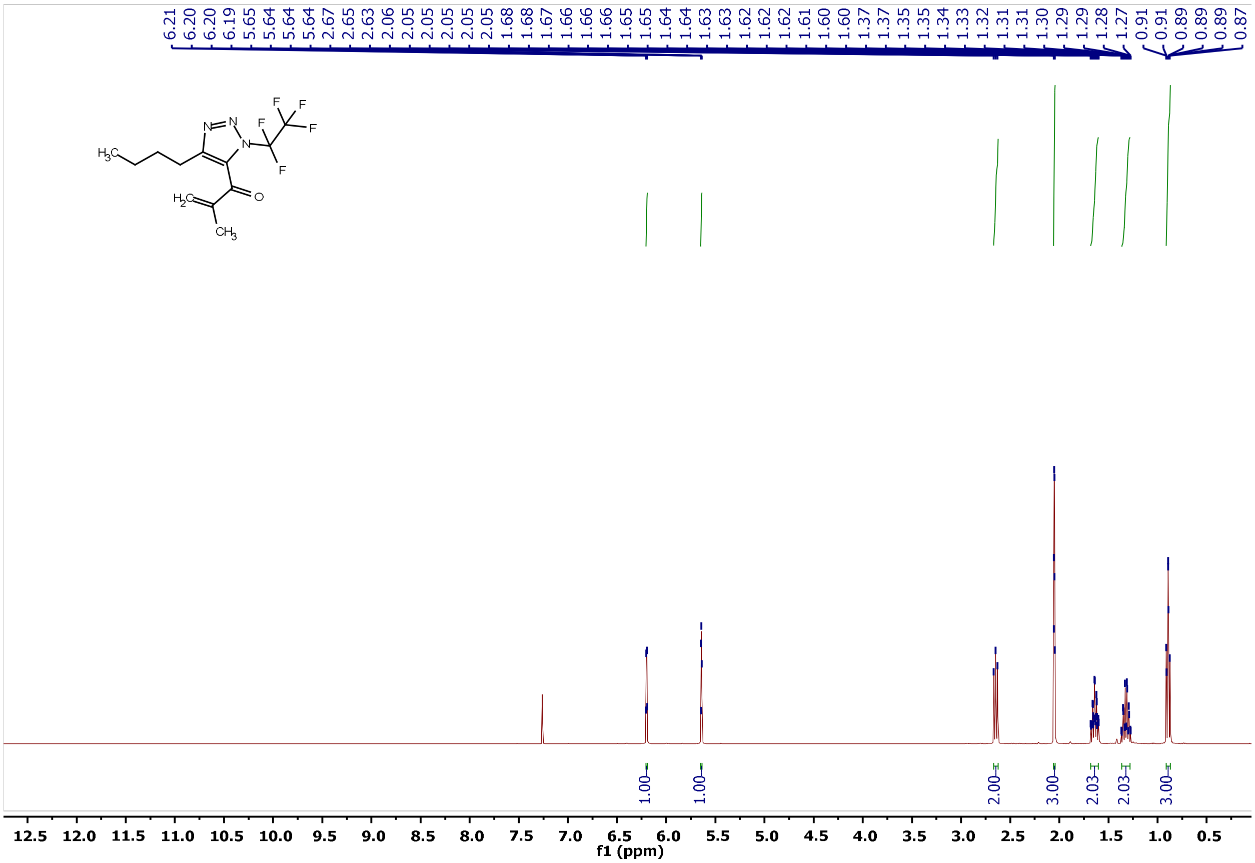

Figure S14. ^{13}C NMR spectrum of **3e** ($CDCl_3$, 101 MHz)

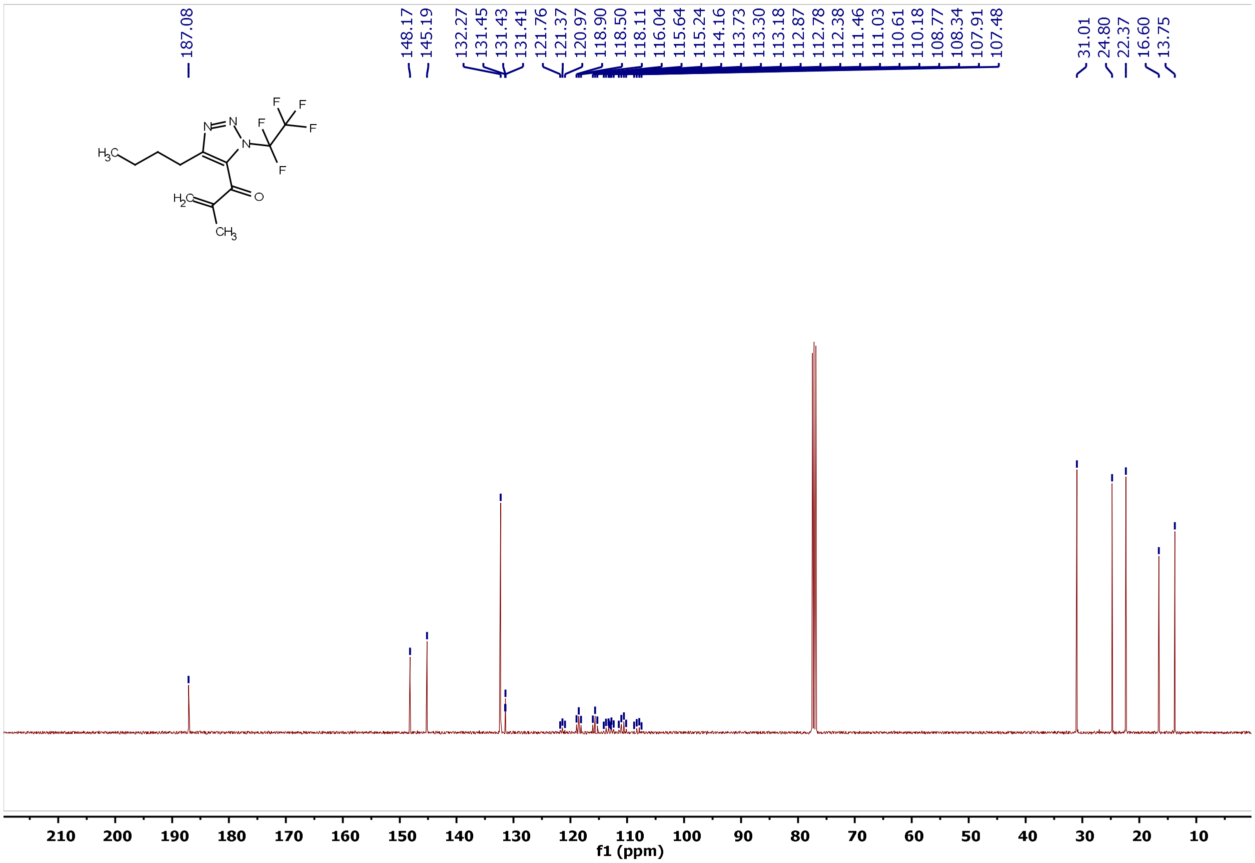

Figure S15. ^{19}F NMR spectrum of **3e** ($CDCl_3$, 377 MHz)

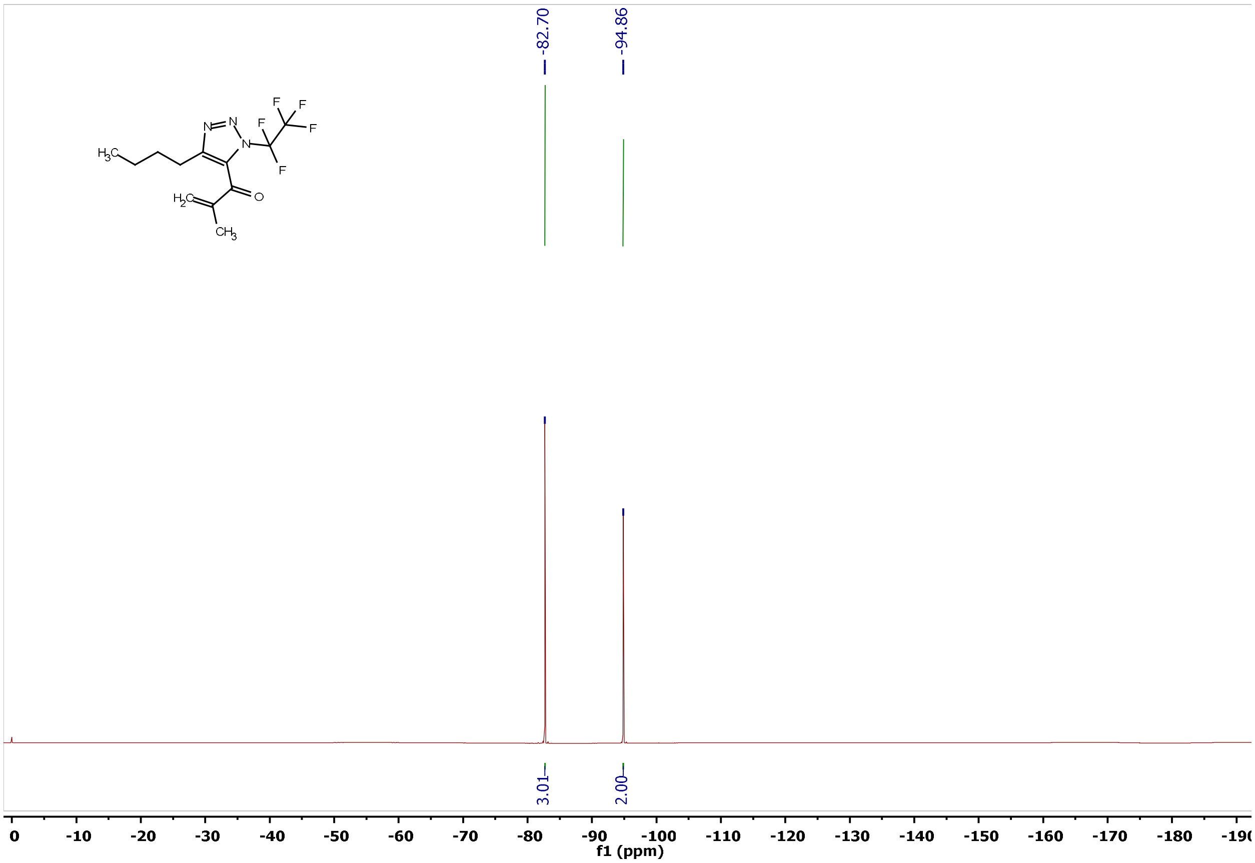

Figure S16. ^{1}H NMR spectrum of **3f** ($CDCl_3$, 401 MHz)

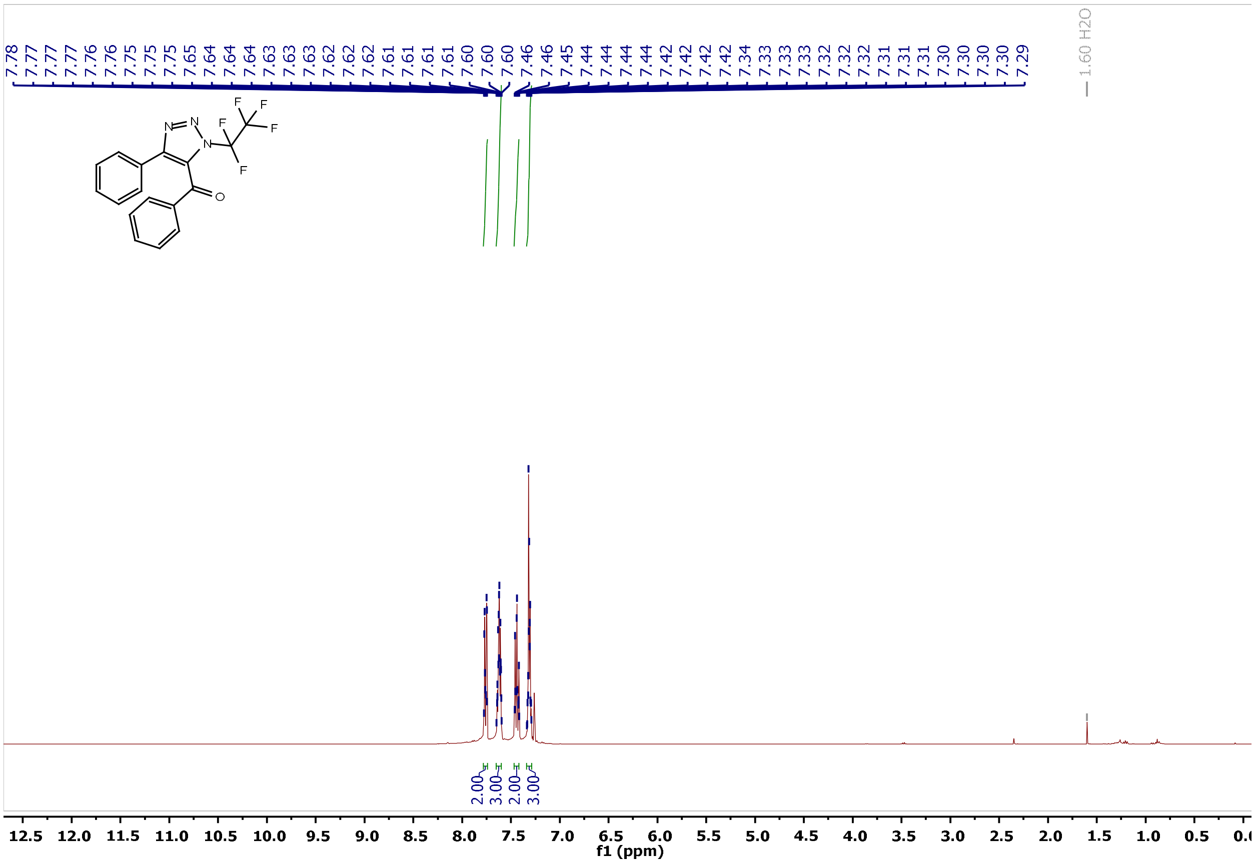

Figure S17. ^{13}C NMR spectrum of **3f** ($CDCl_3$, 101 MHz)

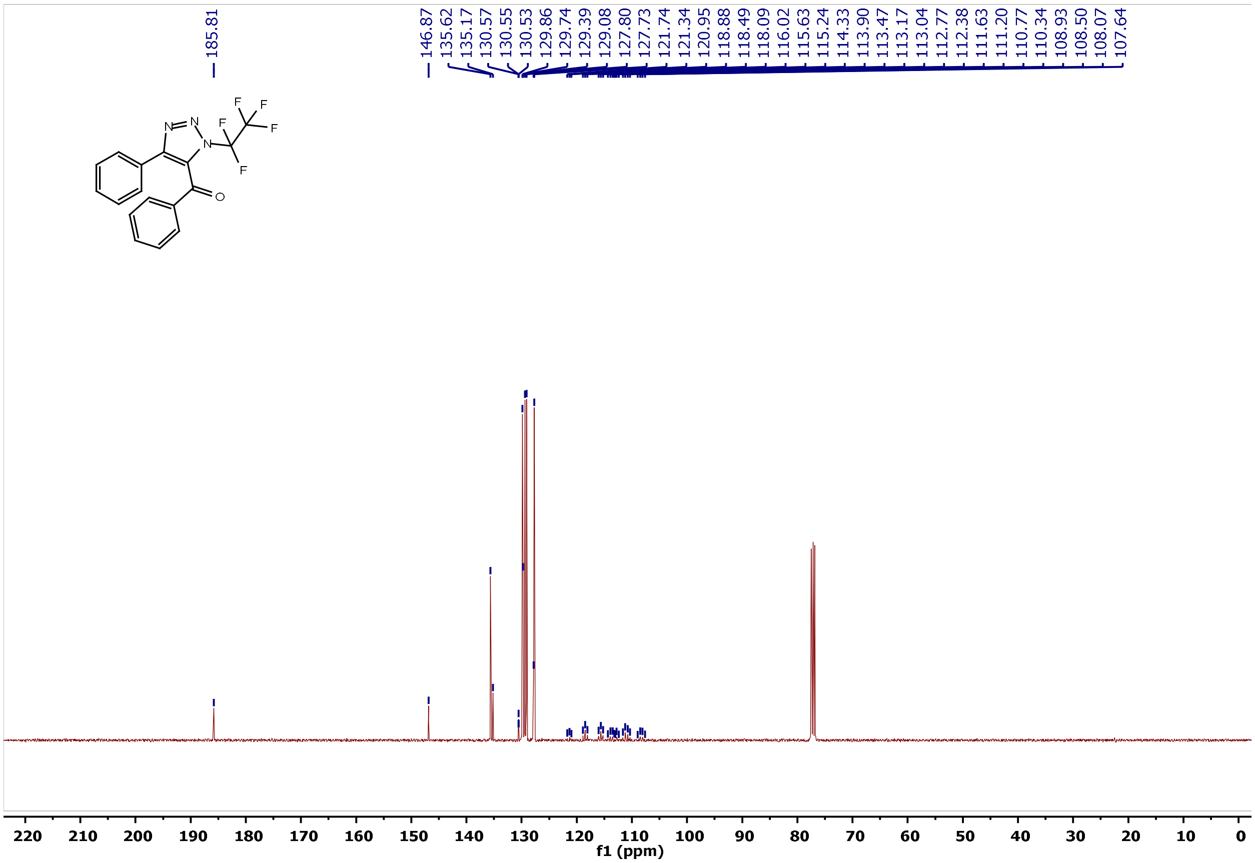

Figure S18. ^{19}F NMR spectrum of **3f** ($CDCl_3$, 471 MHz)

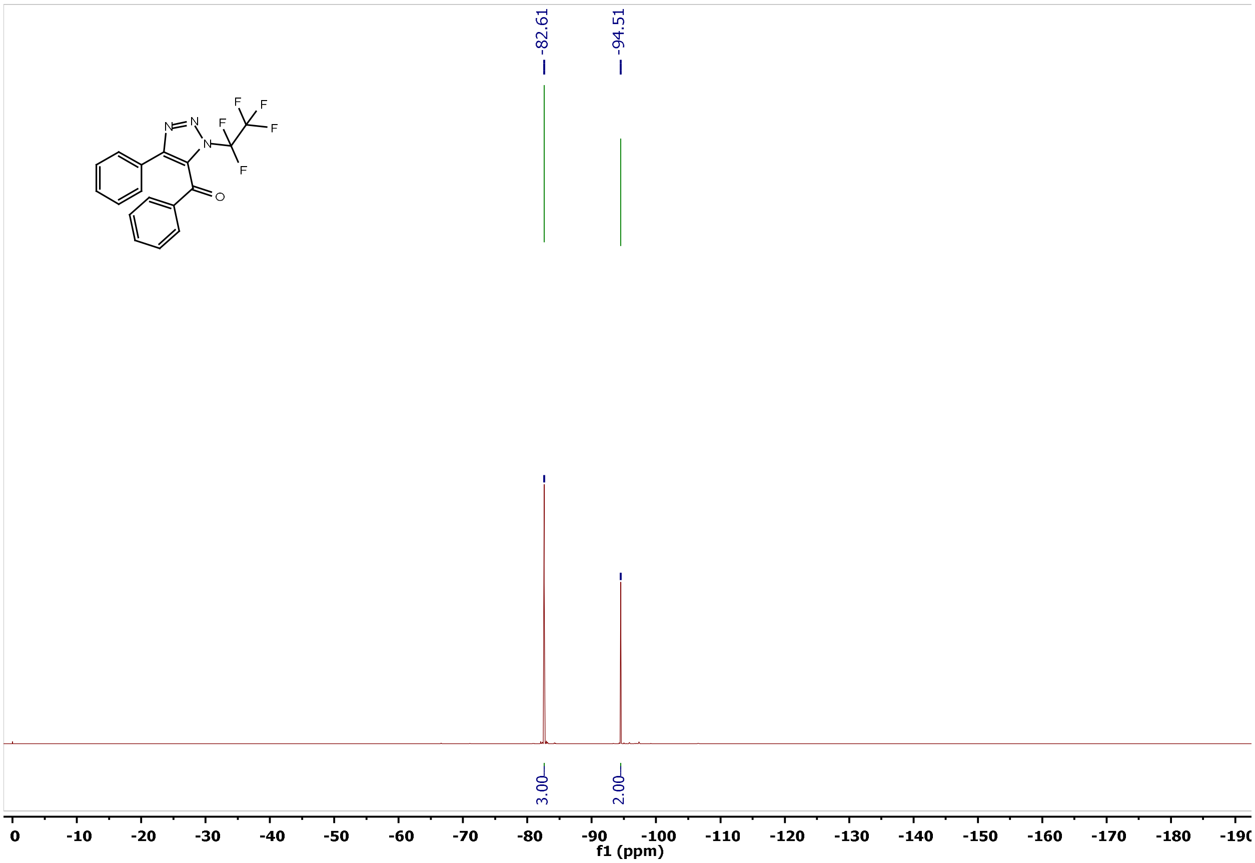

Figure S19. ^{1}H NMR spectrum of **3g** ($CDCl_3$, 401 MHz)

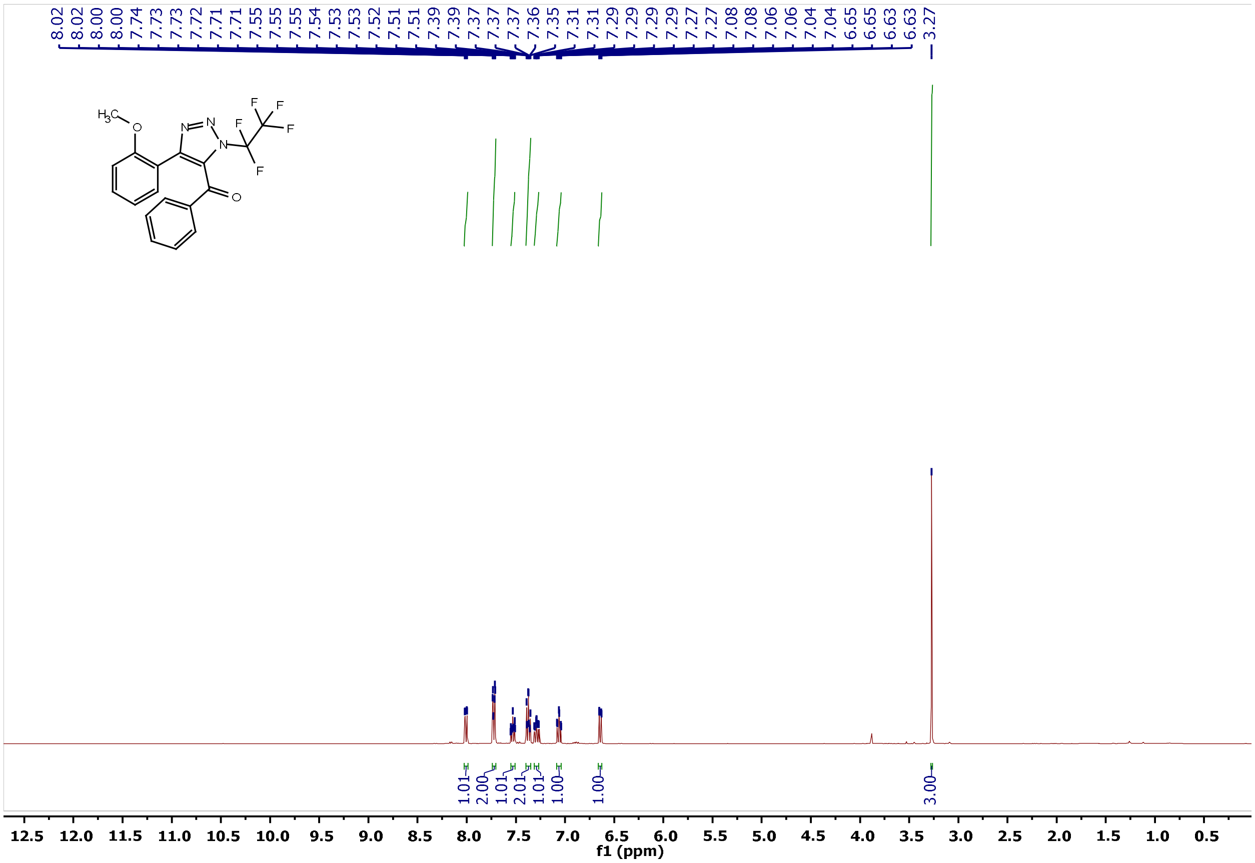

Figure S20. ^{13}C NMR spectrum of **3g** ($CDCl_3$, 101 MHz)

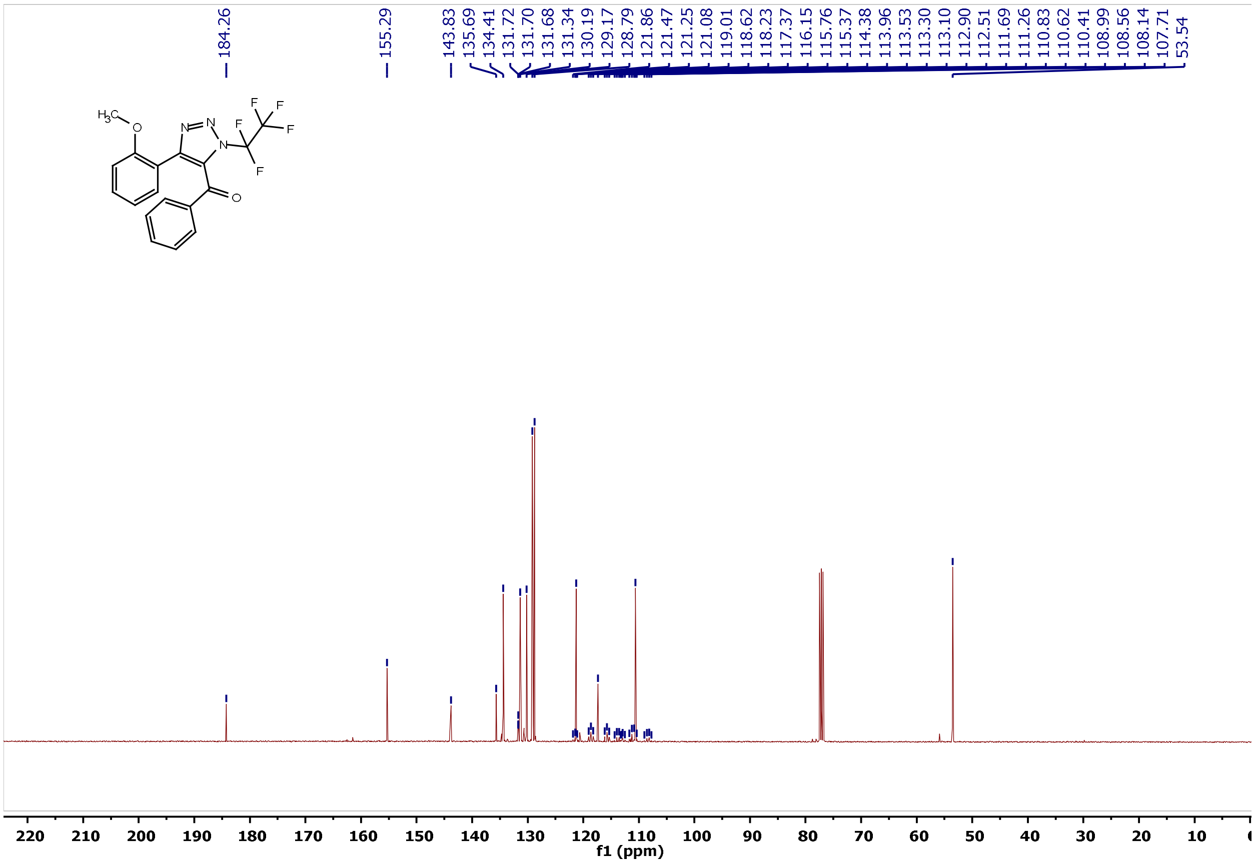

Figure S21. ^{19}F NMR spectrum of **3g** ($CDCl_3$, 377 MHz)

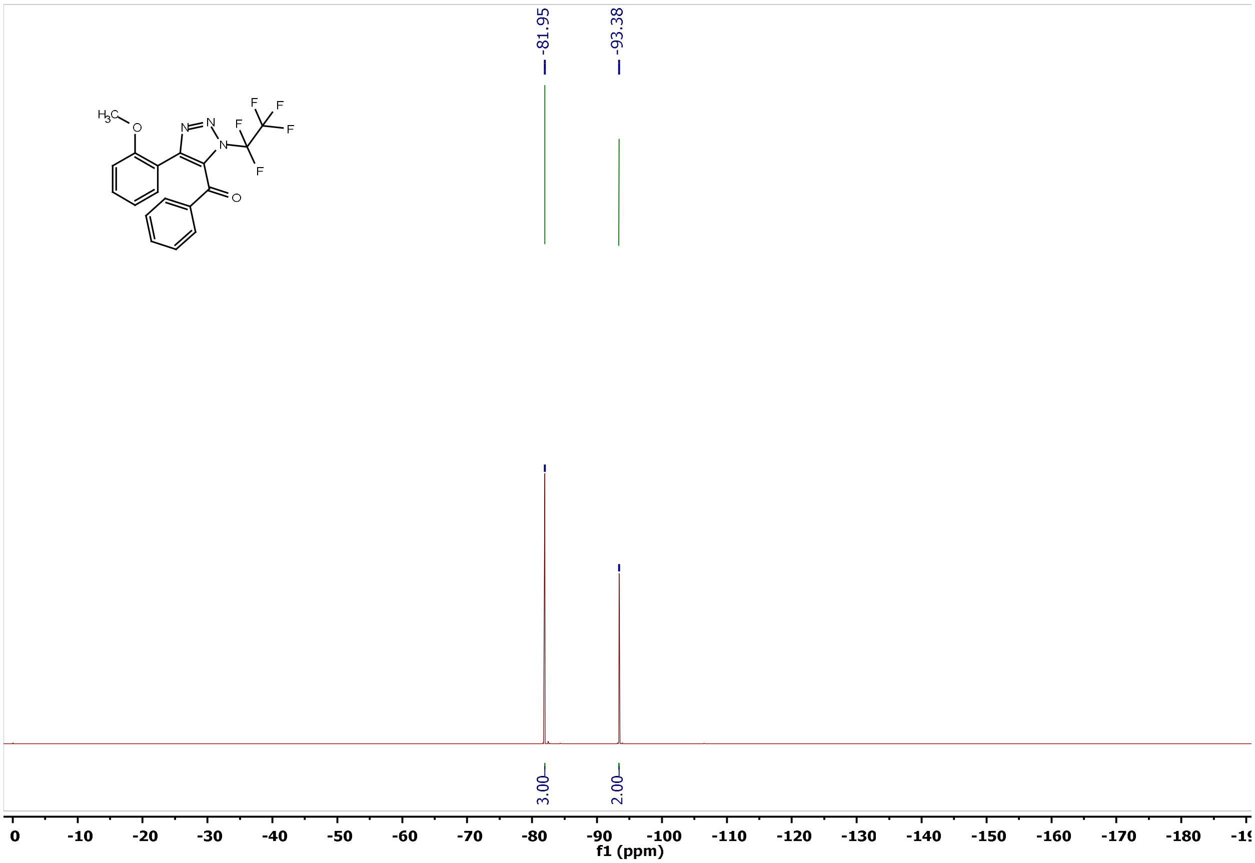

Figure S22. ^1H NMR spectrum of **3h** ($CDCl_3$, 401 MHz)

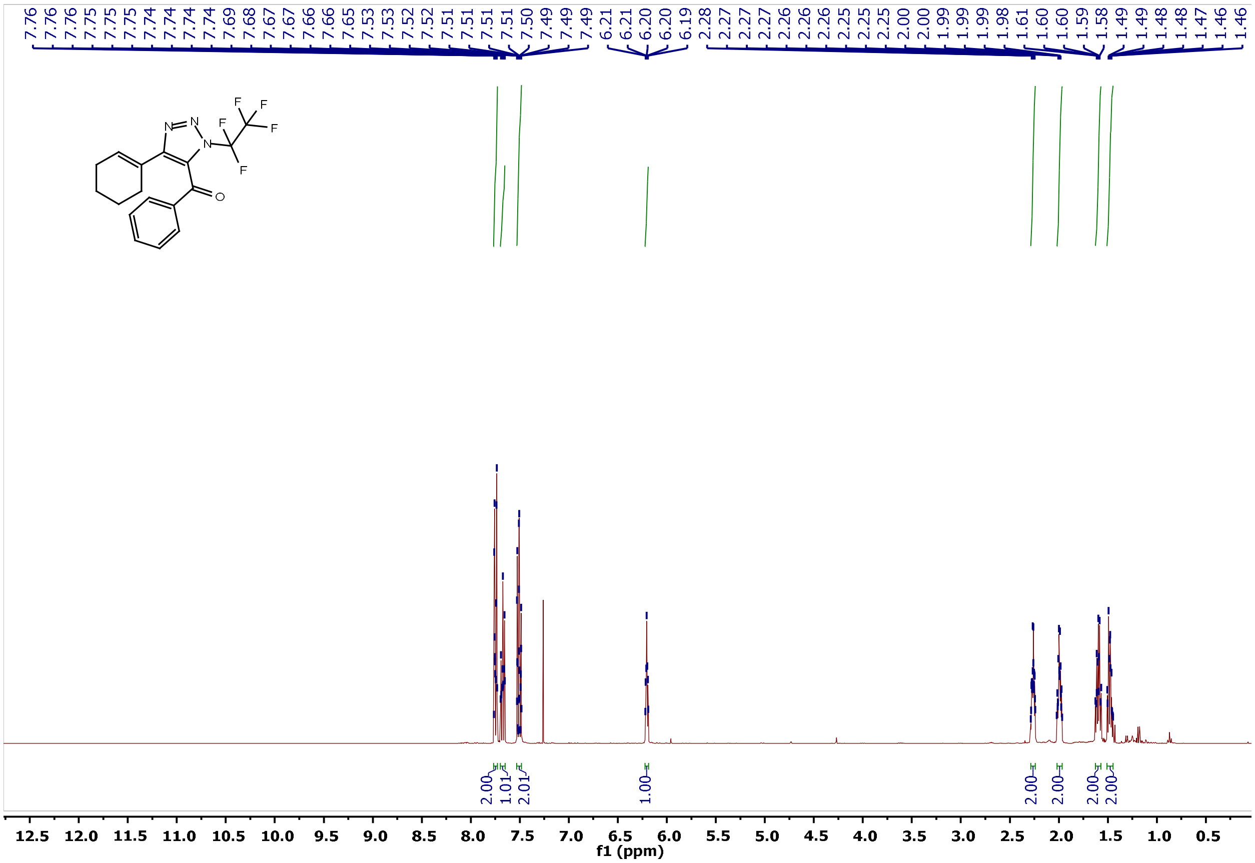

Figure S23. ^{13}C NMR spectrum of **3h** ($CDCl_3$, 101 MHz)

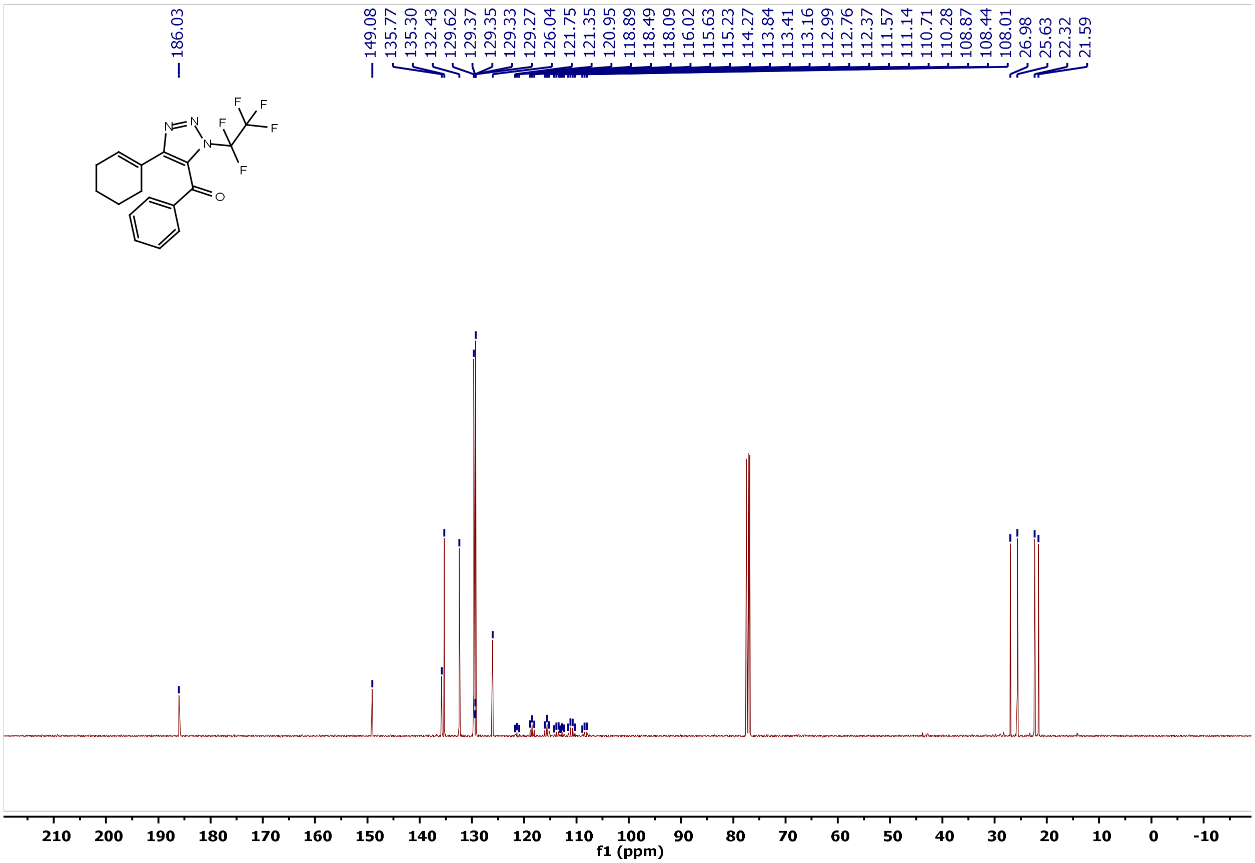

Figure S24. ^{19}F NMR spectrum of **3h** ($CDCl_3$, 377 MHz)

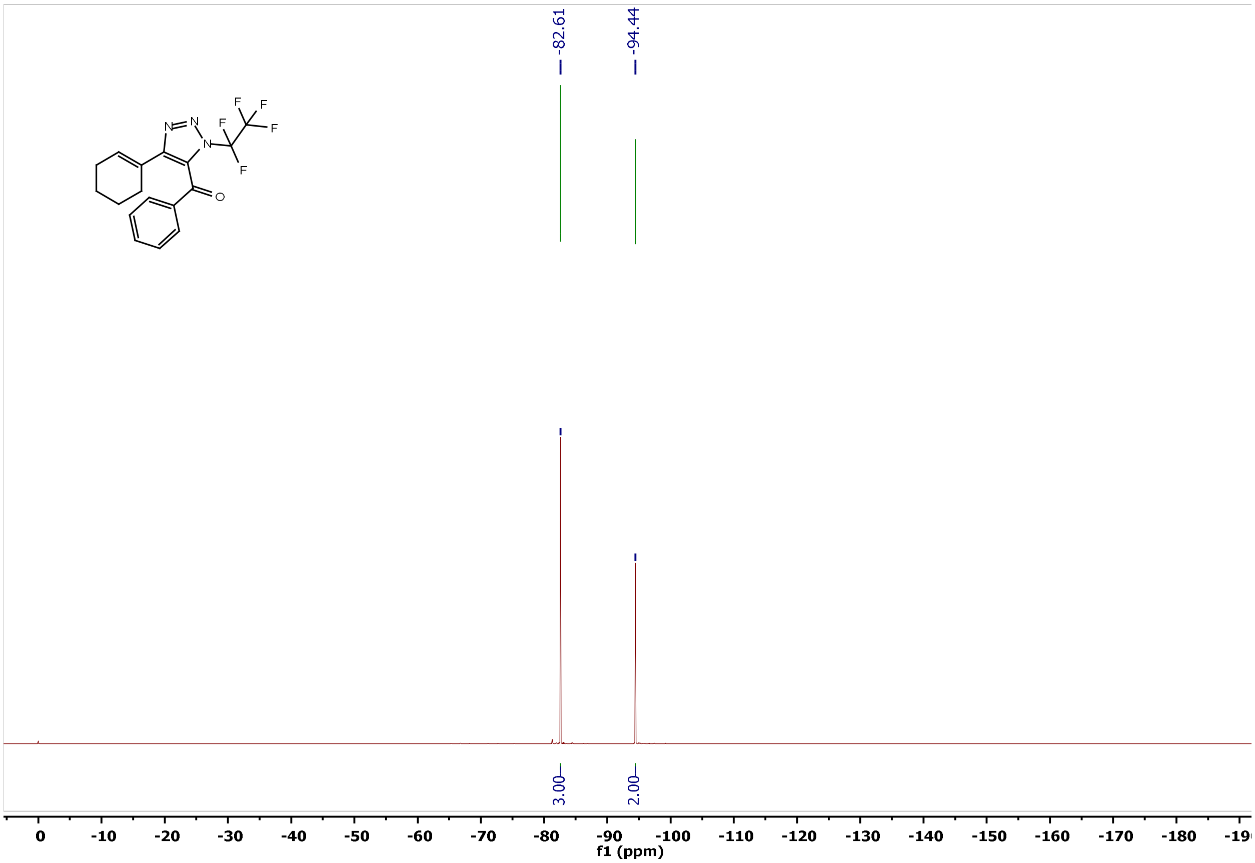

Figure S25. ^{1}H NMR spectrum of **3i** ($CDCl_3$, 401 MHz)

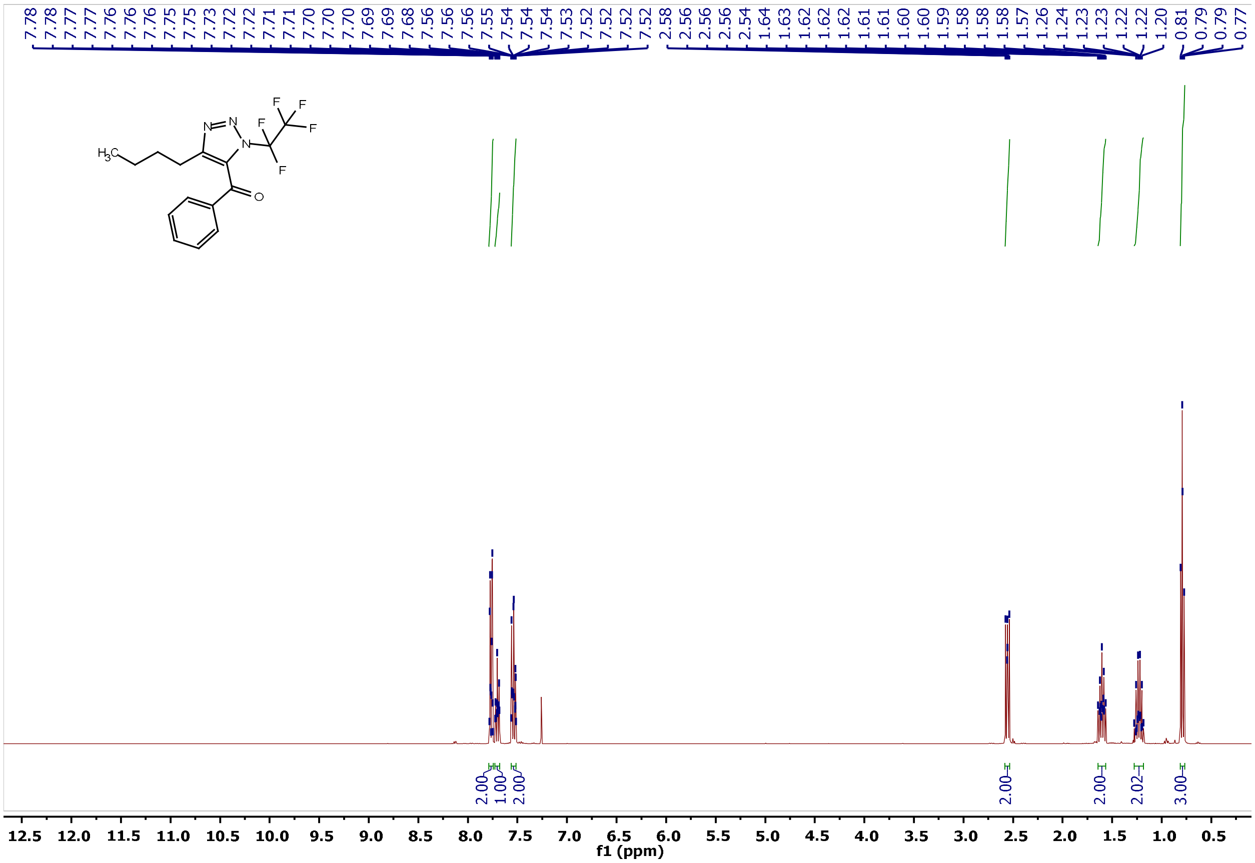

Figure S26. ^{13}C NMR spectrum of **3i** ($CDCl_3$, 101 MHz)

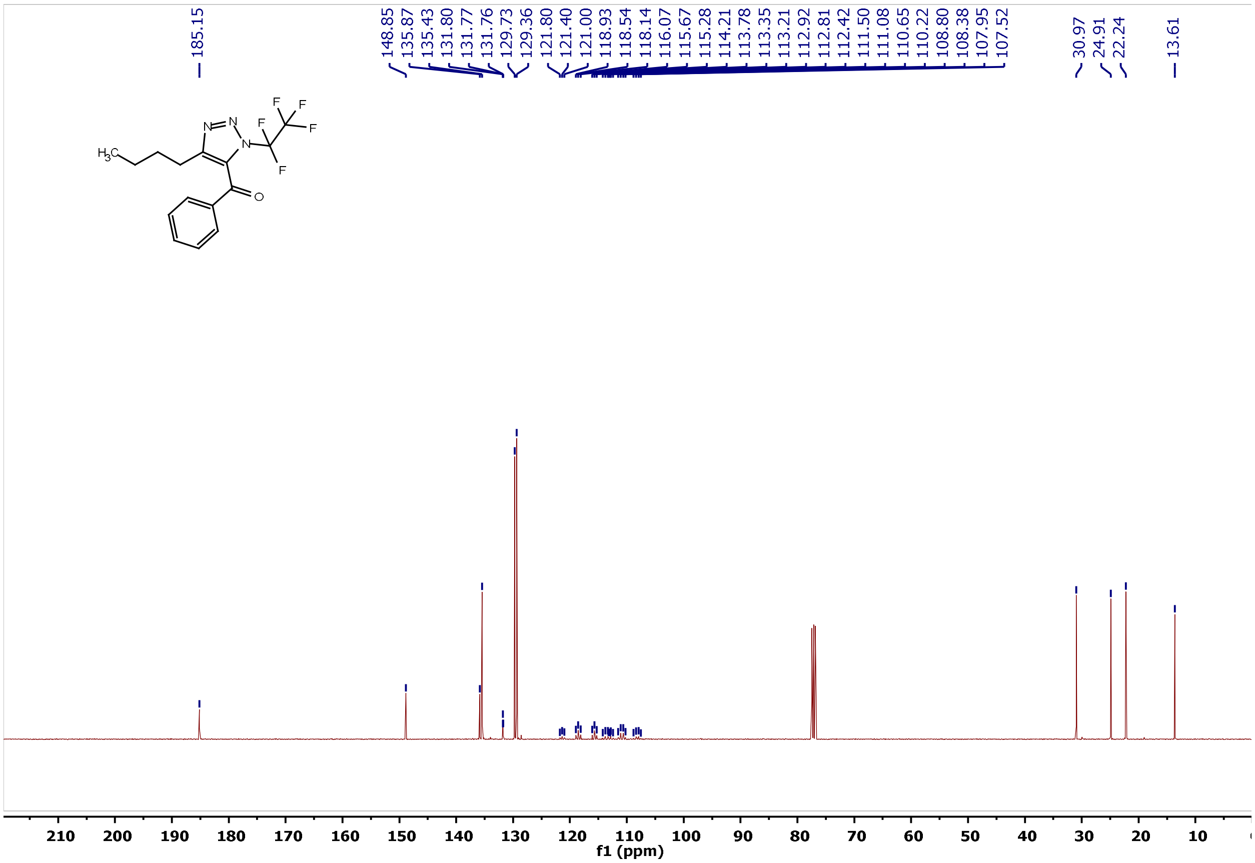

Figure S27. ^{19}F NMR spectrum of **3i** ($CDCl_3$, 377 MHz)

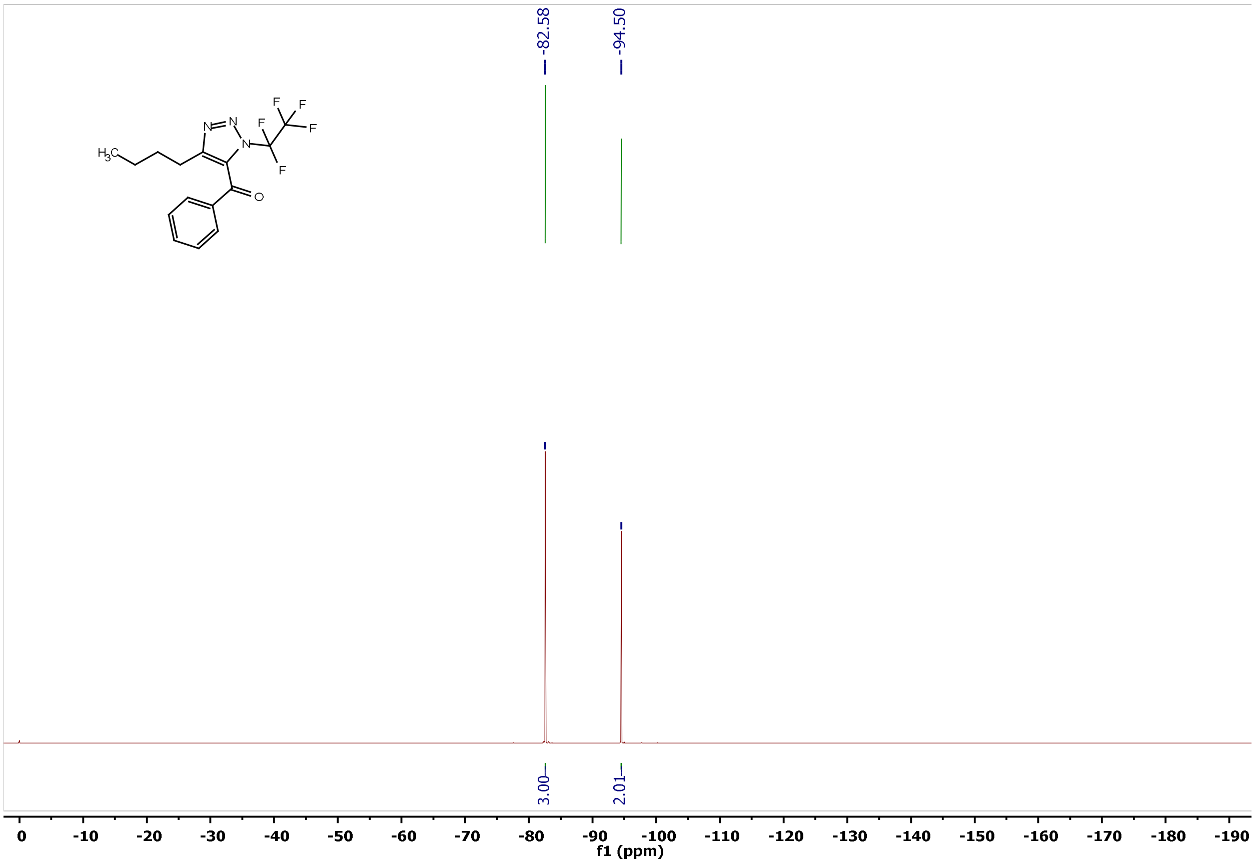

Figure S28. ^{1}H NMR spectrum of **3j** ($CDCl_3$, 401 MHz)

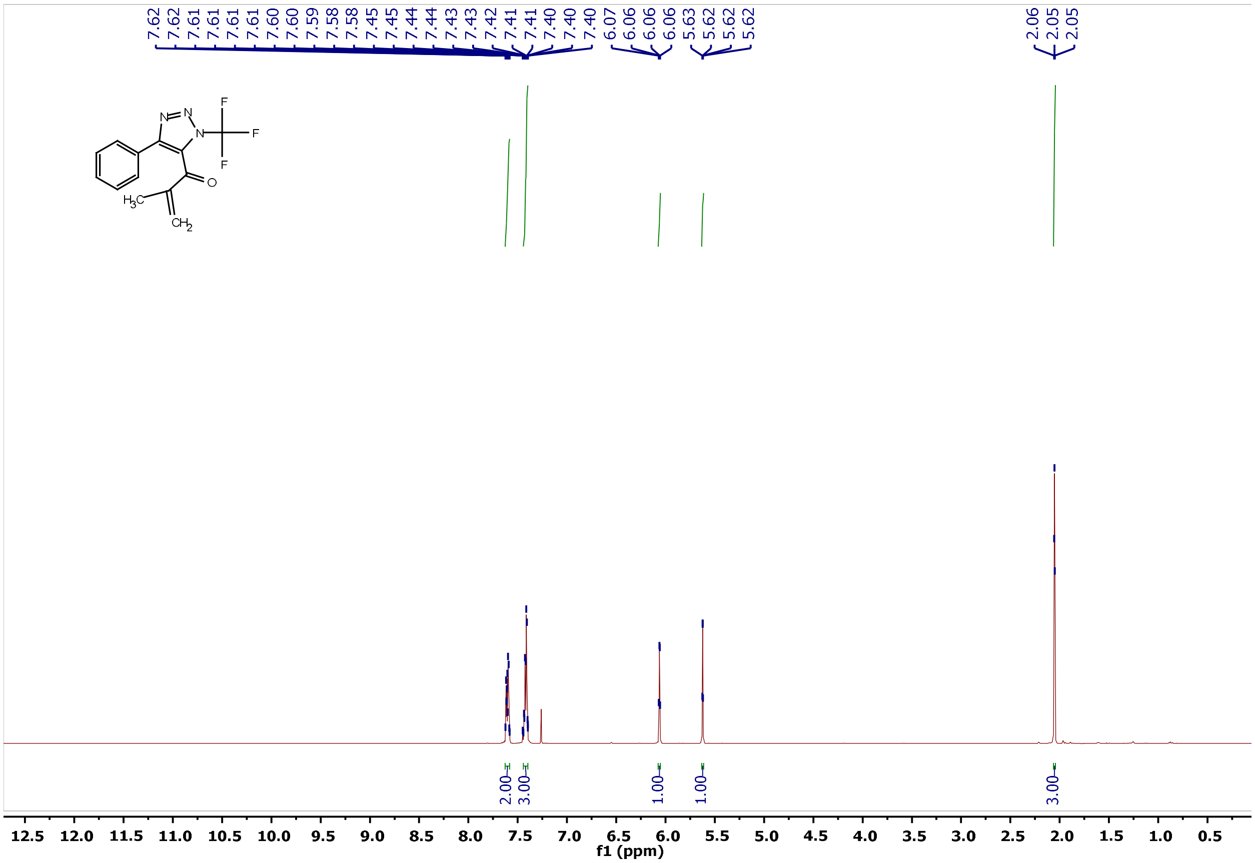

Figure S29. ^{13}C NMR spectrum of **3j** ($CDCl_3$, 101 MHz)

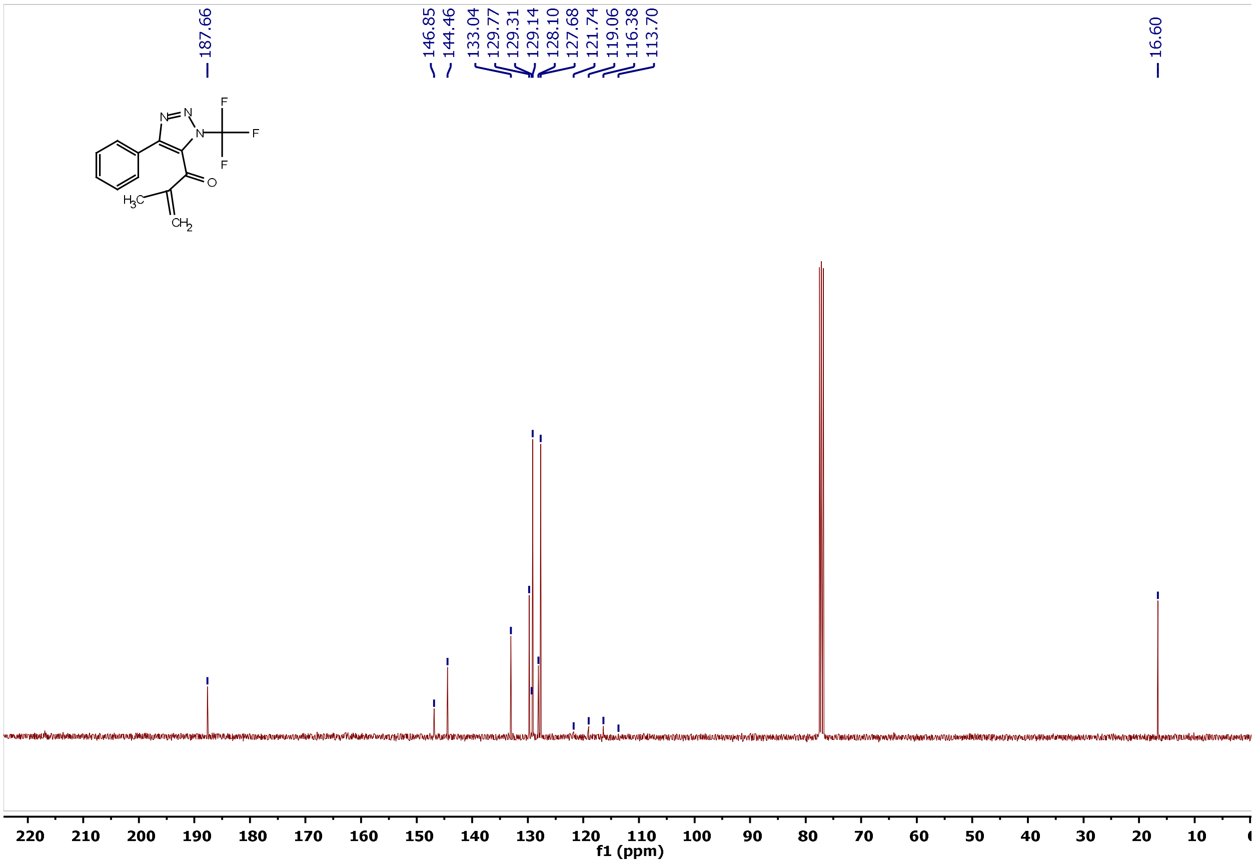

Figure S30. ^{19}F NMR spectrum of **3j** ($CDCl_3$, 377 MHz)

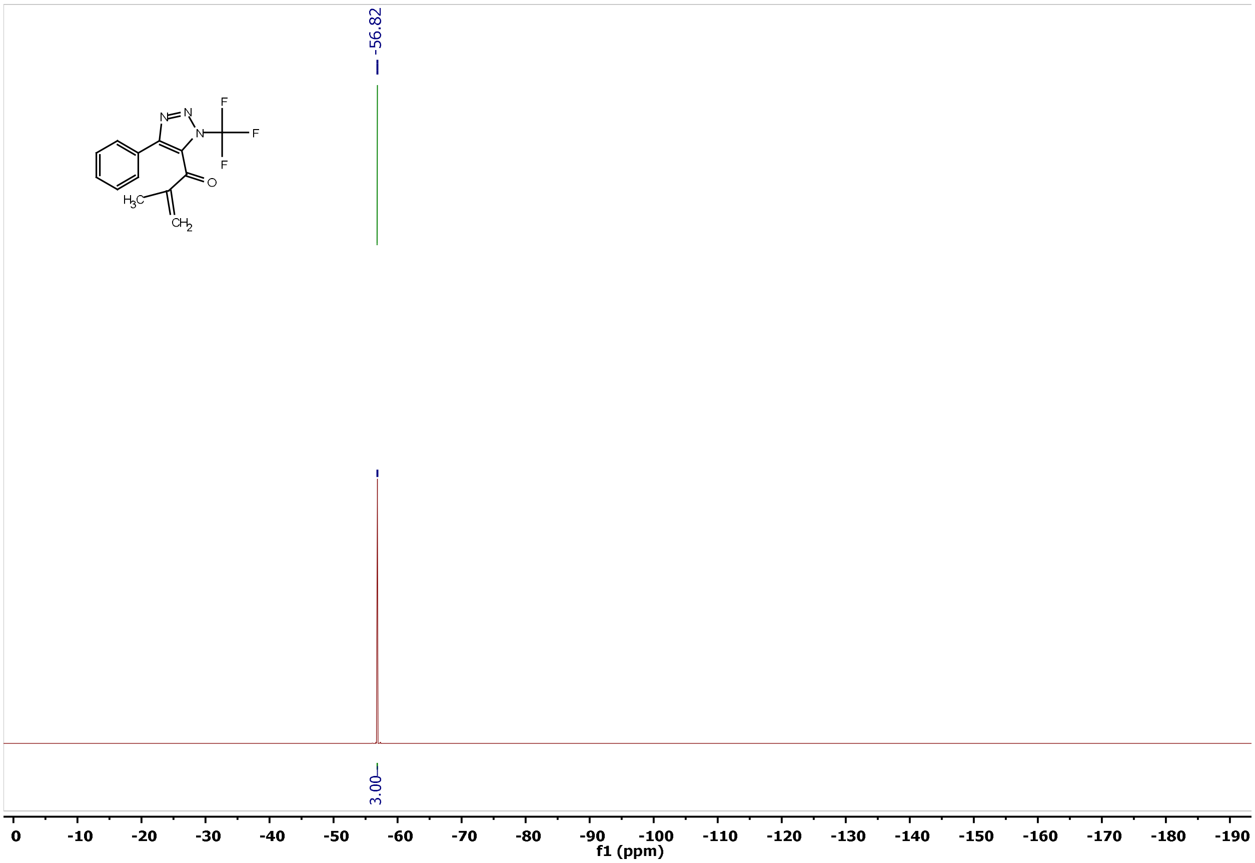

Figure S31. ^{1}H NMR spectrum of **3k** ($CDCl_3$, 401 MHz)

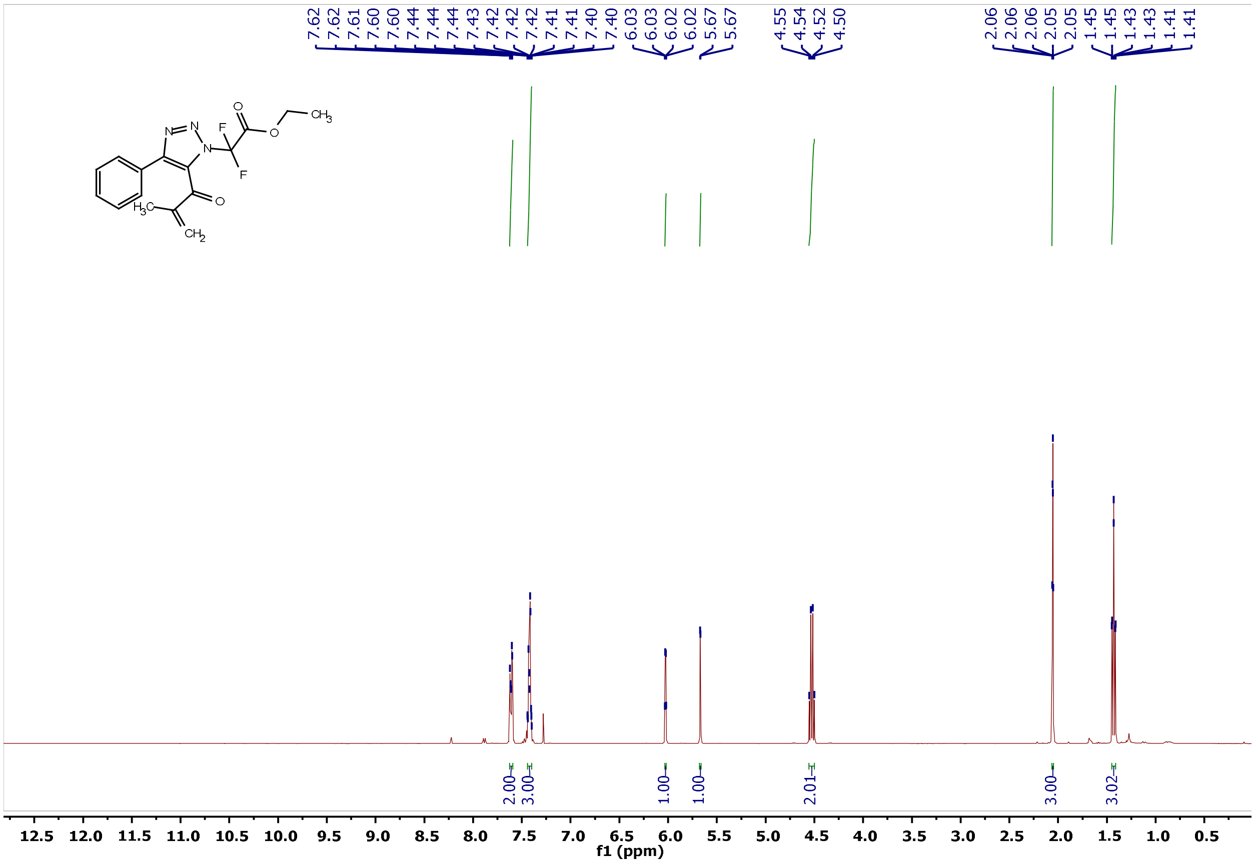

Figure S32. ^{13}C NMR spectrum of **3k** ($CDCl_3$, 101 MHz)

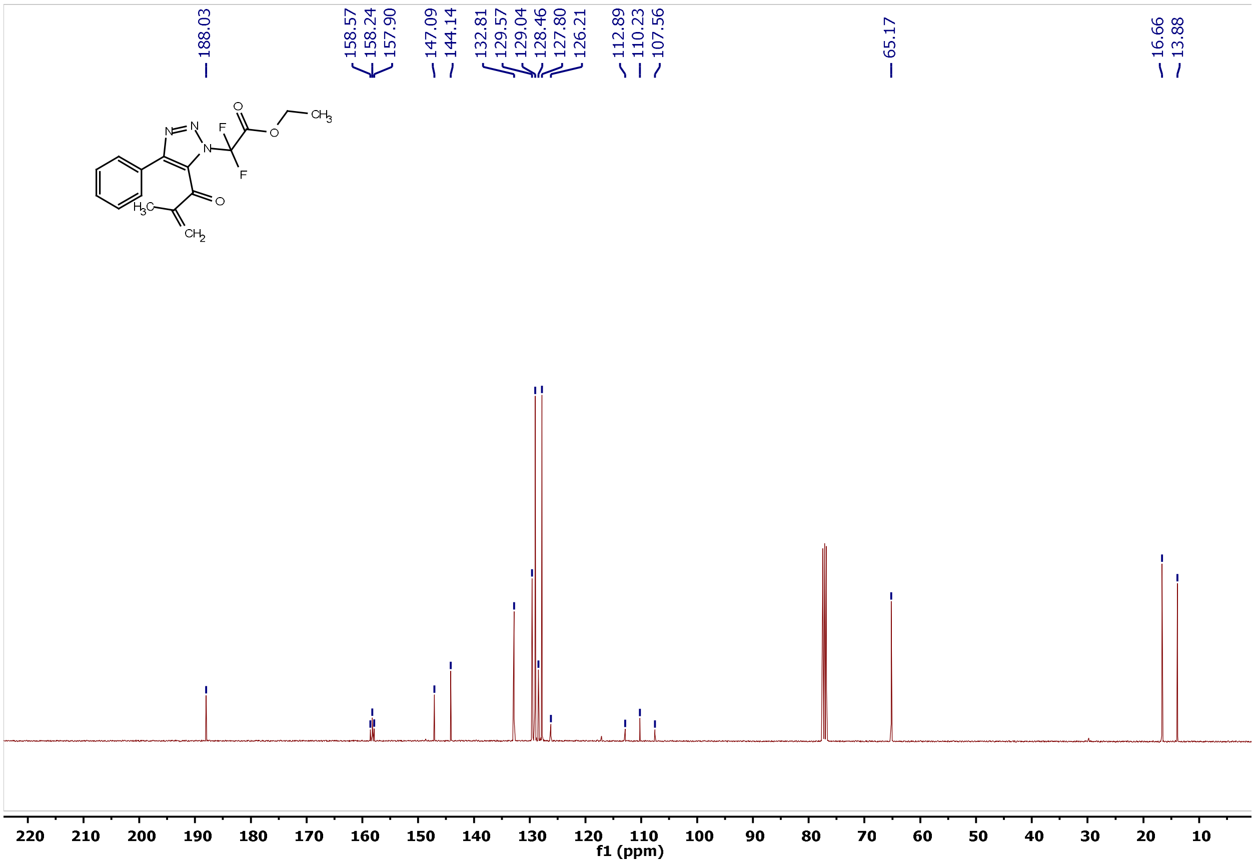

Figure S33. ^{19}F NMR spectrum of **3k** ($CDCl_3$, 377 MHz)

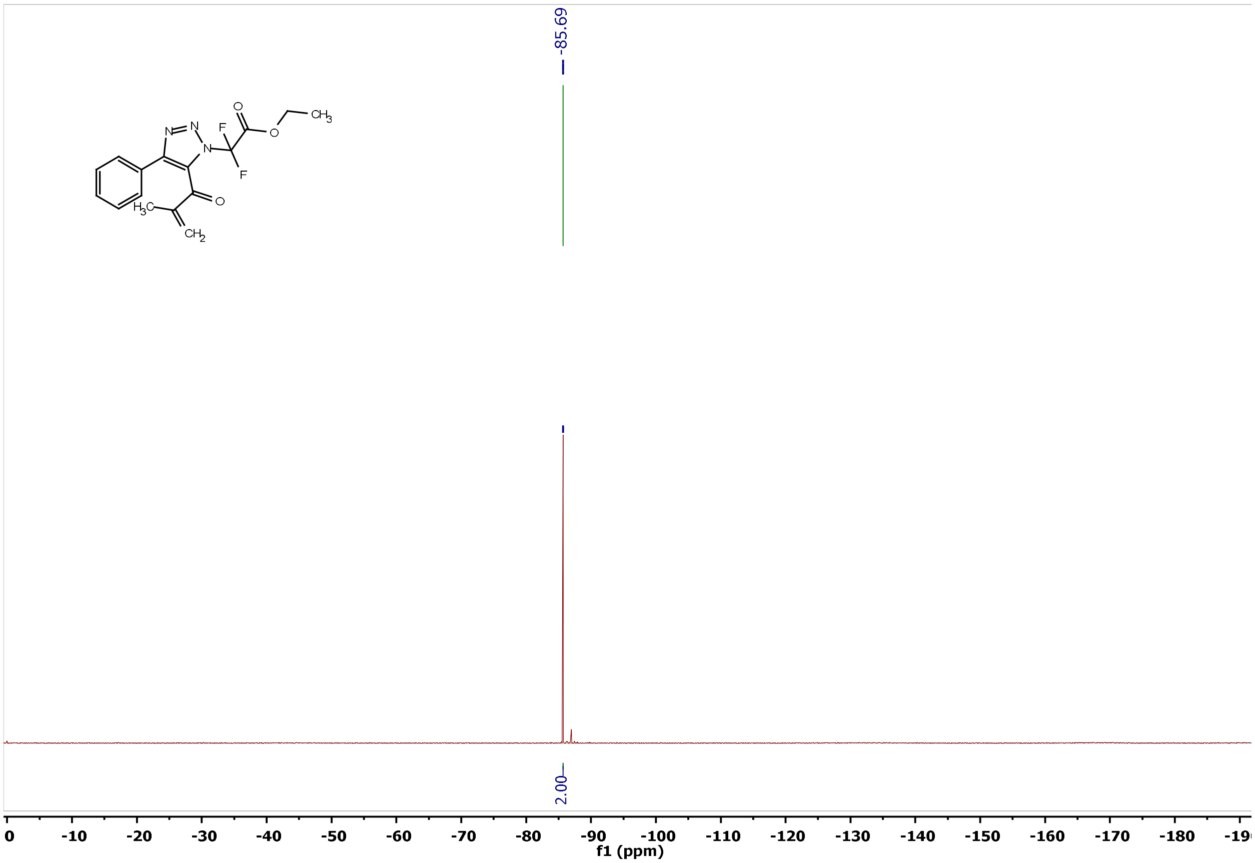

Figure S34. ^1H NMR spectrum of **3l** ($CDCl_3$, 401 MHz)

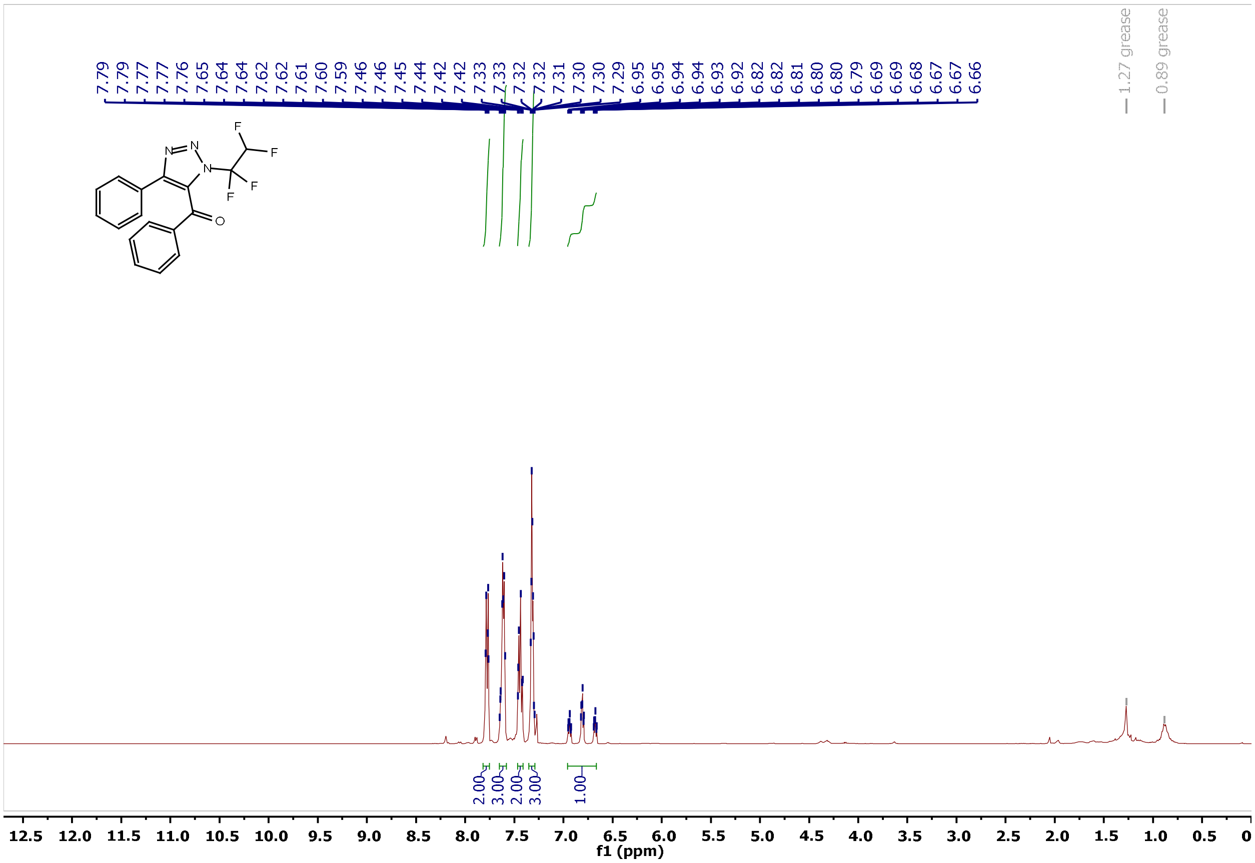

Figure S35. ^{13}C NMR spectrum of **3l** ($CDCl_3$, 101 MHz)

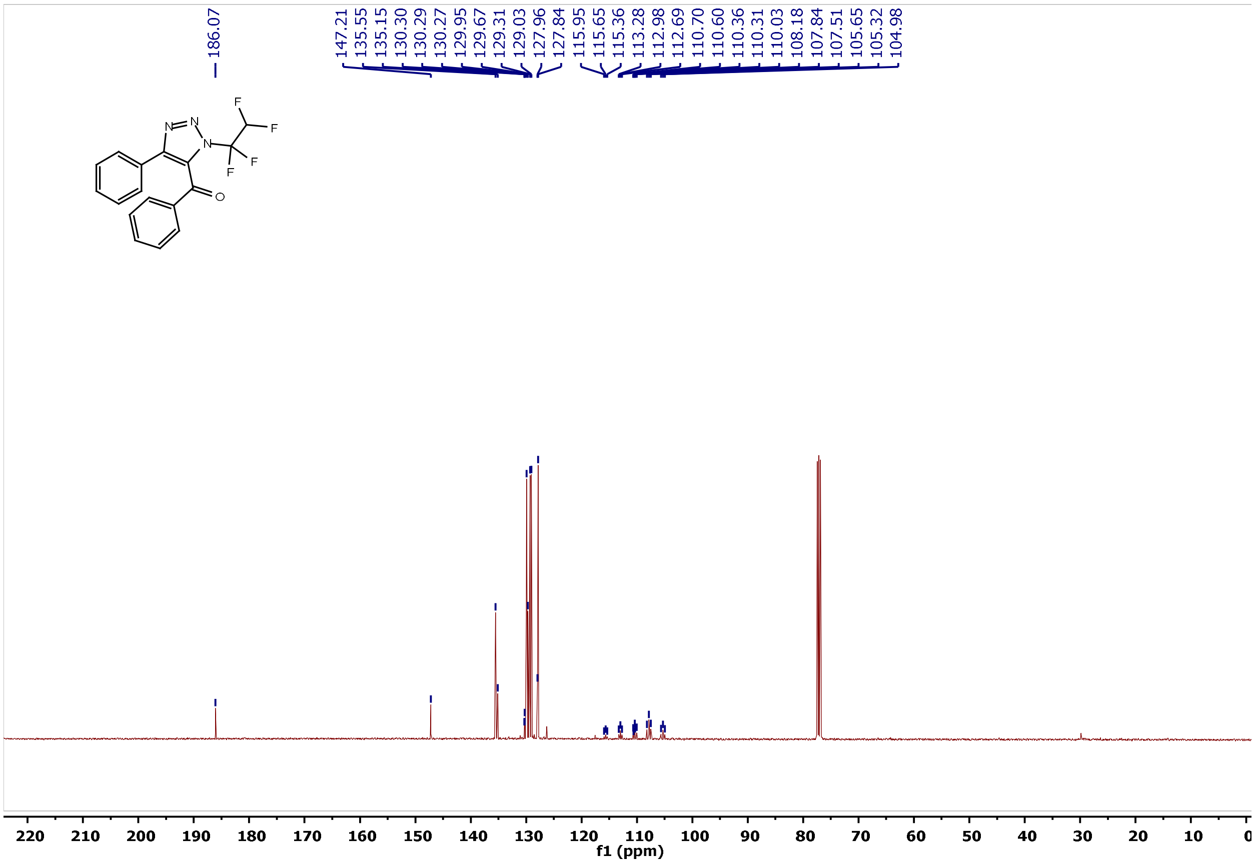

Figure S36. ^{19}F NMR spectrum of **3l** ($CDCl_3$, 377 MHz)

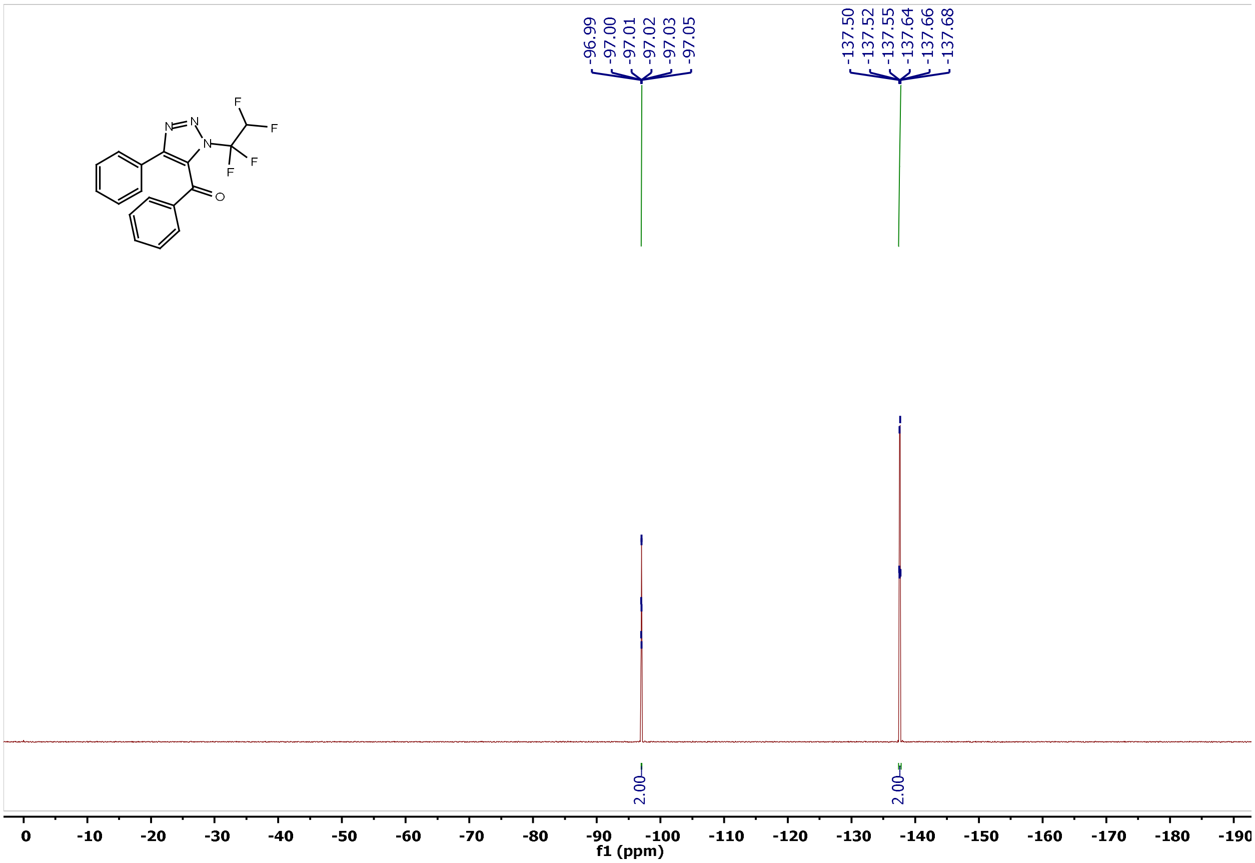

Figure S37. ^{1}H NMR spectrum of **3m** ($CDCl_3$, 401 MHz)

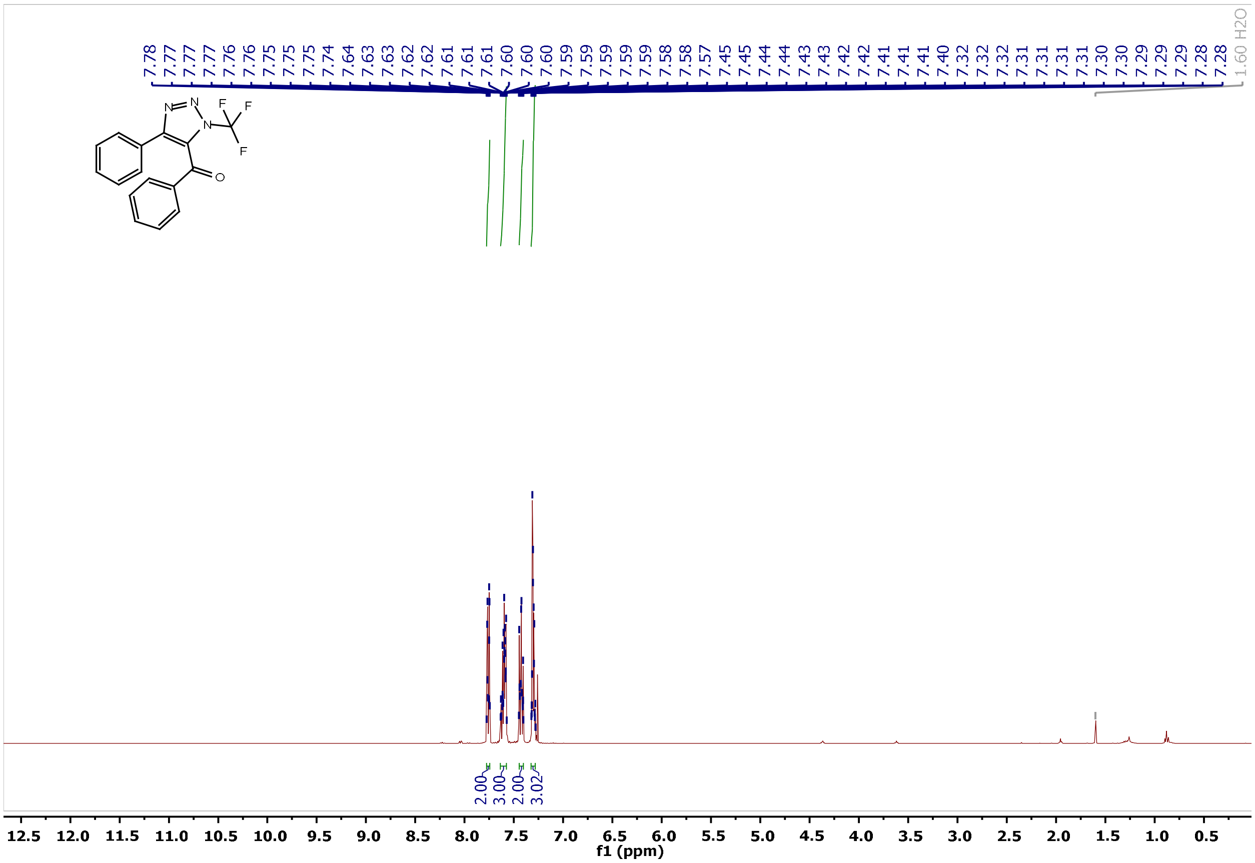

Figure S38. ^{13}C NMR spectrum of **3m** ($CDCl_3$, 101 MHz)

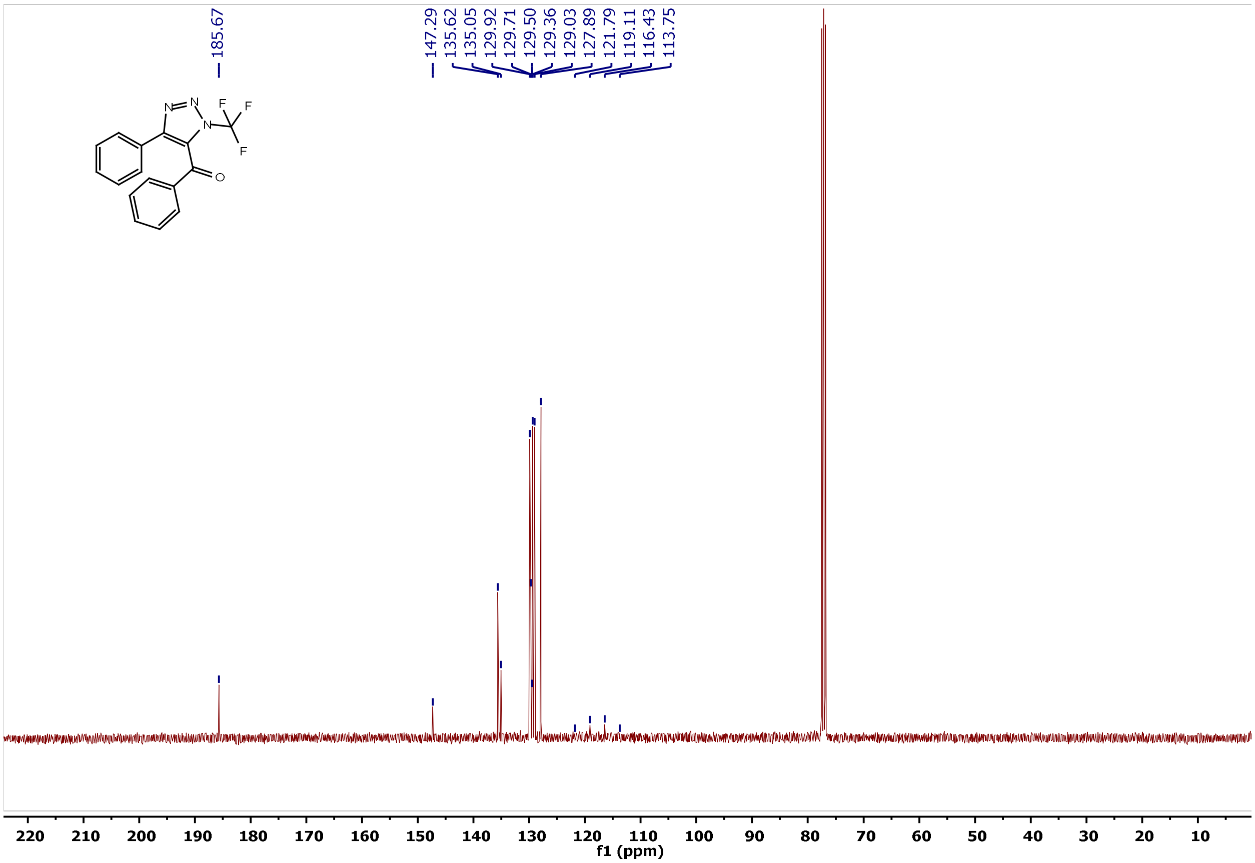

Figure S39. ^{19}F NMR spectrum of **3m** ($CDCl_3$, 377 MHz)

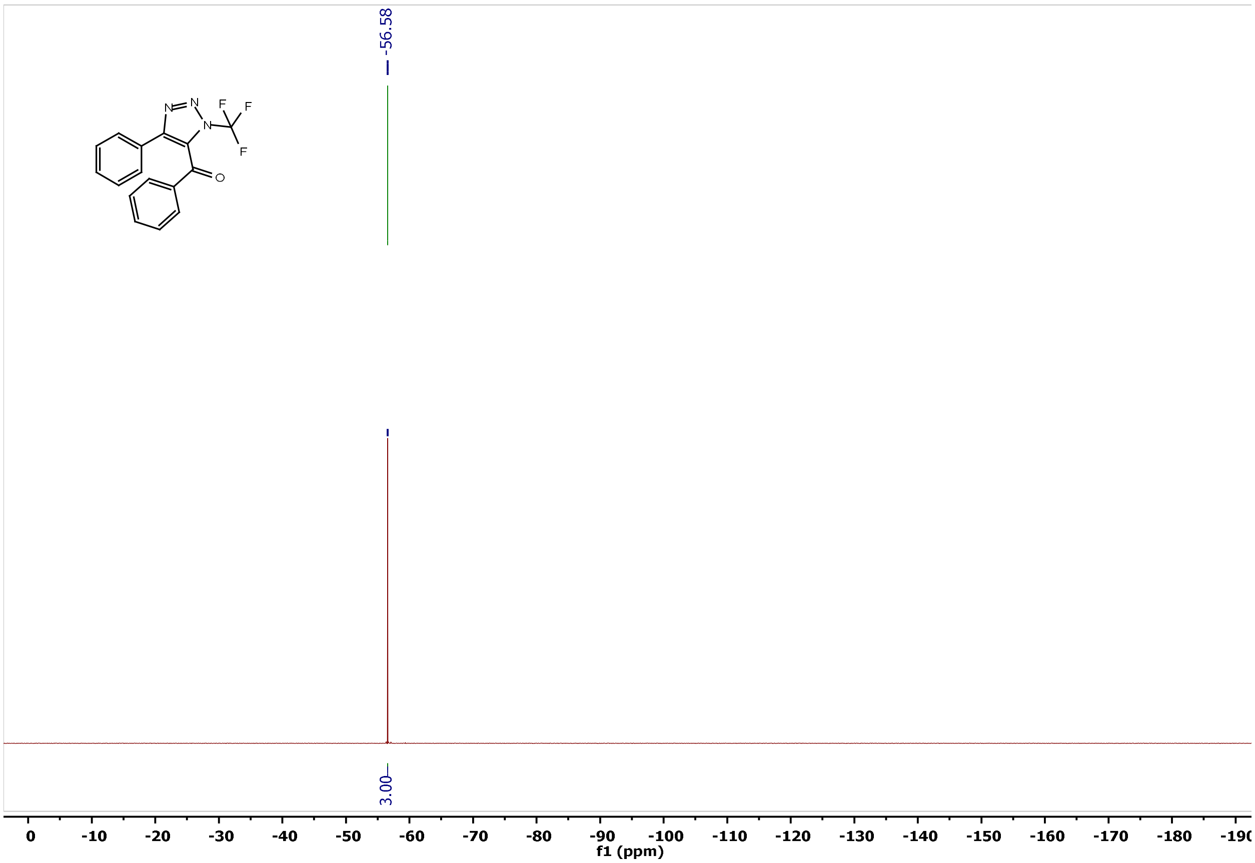

Figure S40. ^{1}H NMR spectrum of **3n** ($CDCl_3$, 401 MHz)

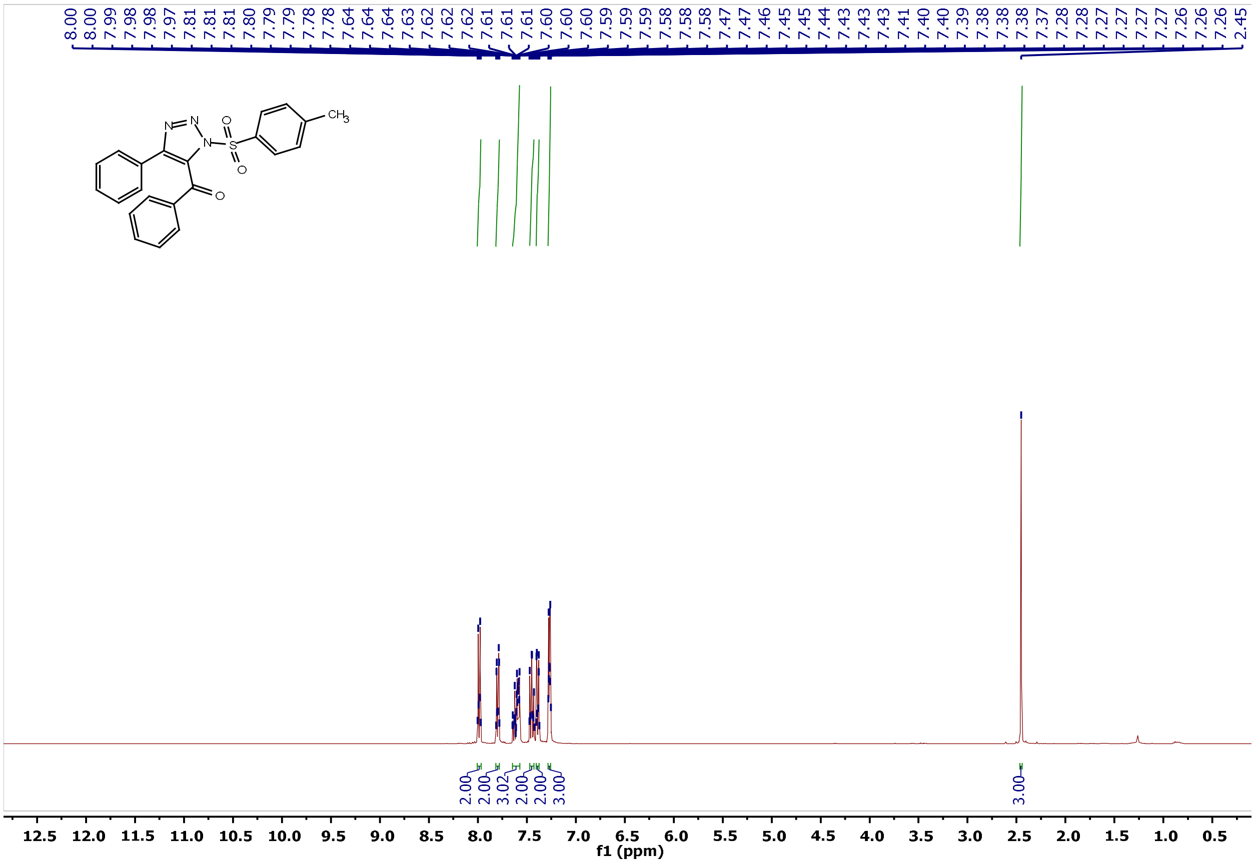

Figure S41. ^{13}C NMR spectrum of **3n** ($CDCl_3$, 101 MHz)

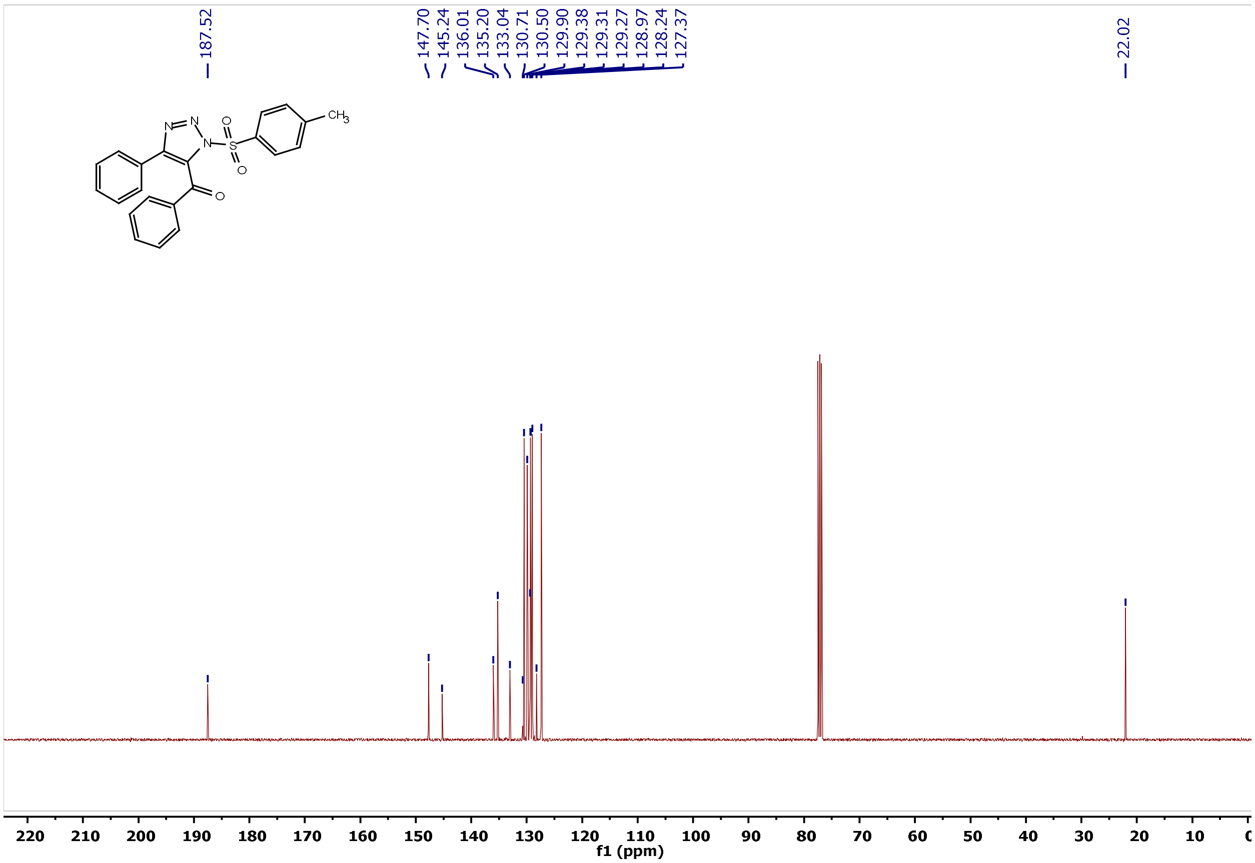

Figure S42. ^{1}H NMR spectrum of **3o** ($CDCl_3$, 401 MHz)

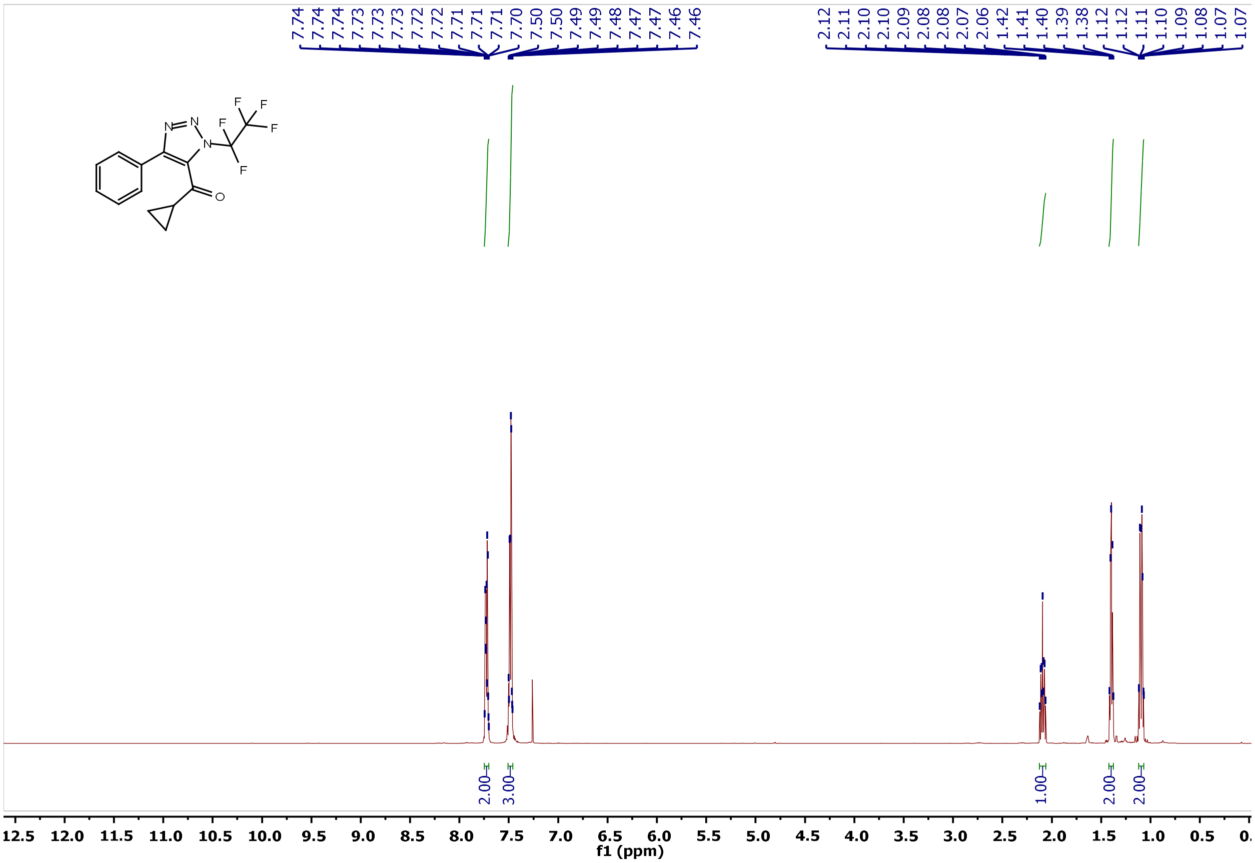

Figure S43. ^{13}C NMR spectrum of **3o** ($CDCl_3$, 101 MHz)

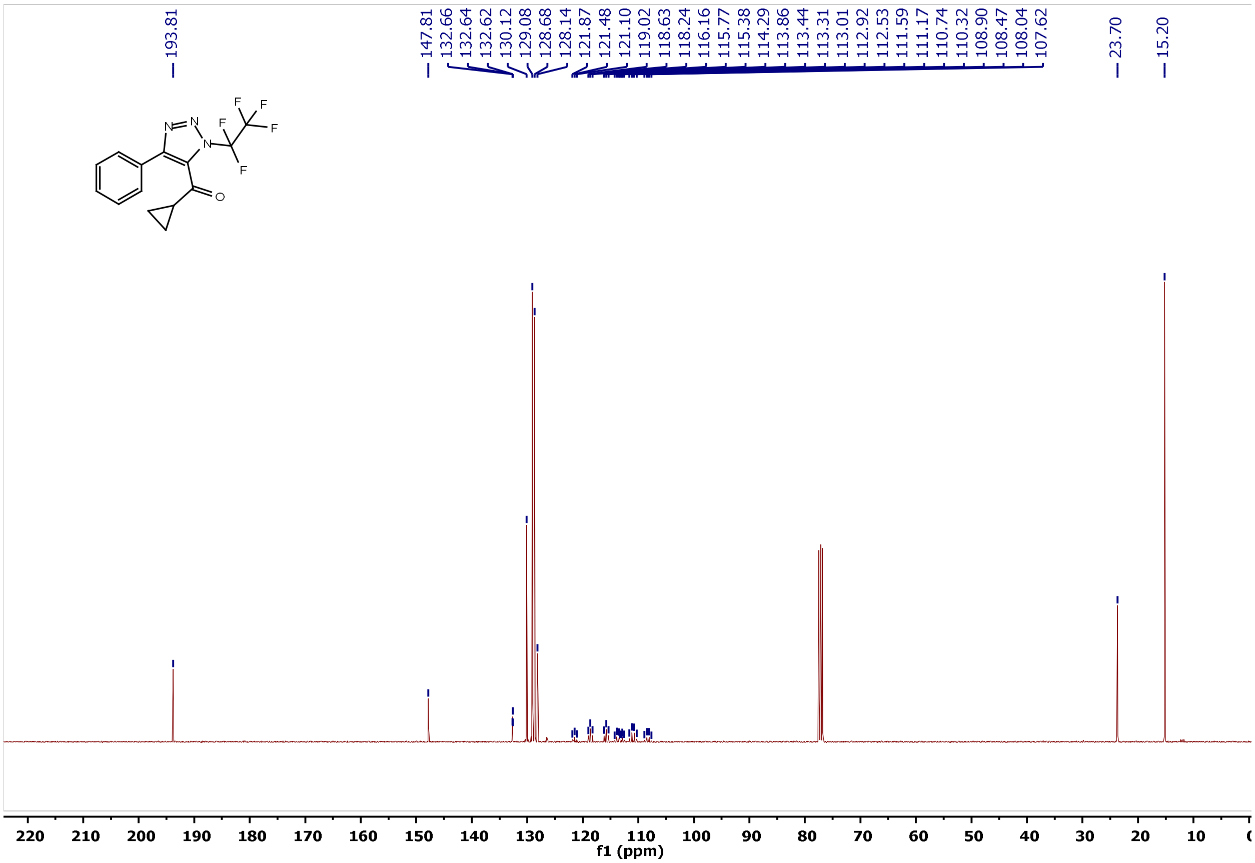

Figure S44. ^{19}F NMR spectrum of **3o** ($CDCl_3$, 377 MHz)

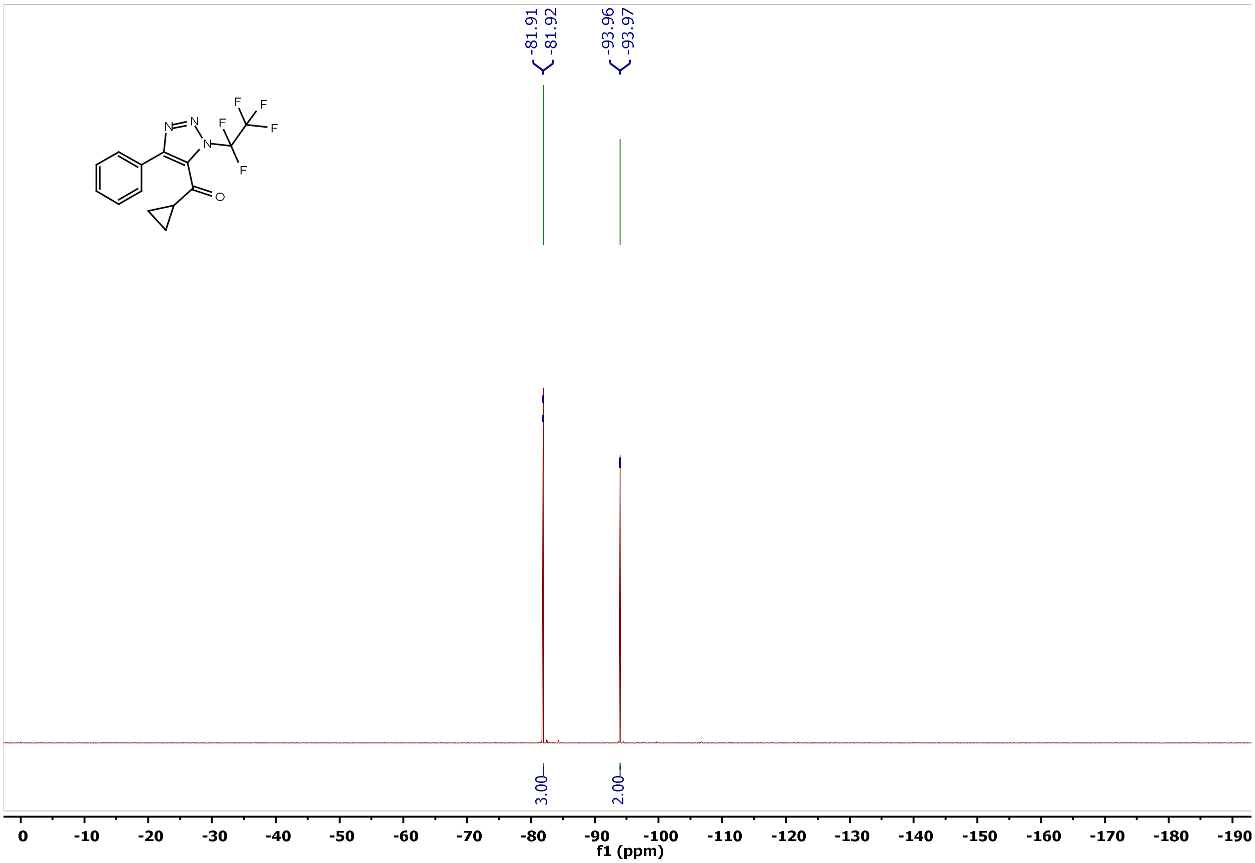

Figure S45. ^{1}H NMR spectrum of **3p** ($CDCl_3$, 401 MHz)

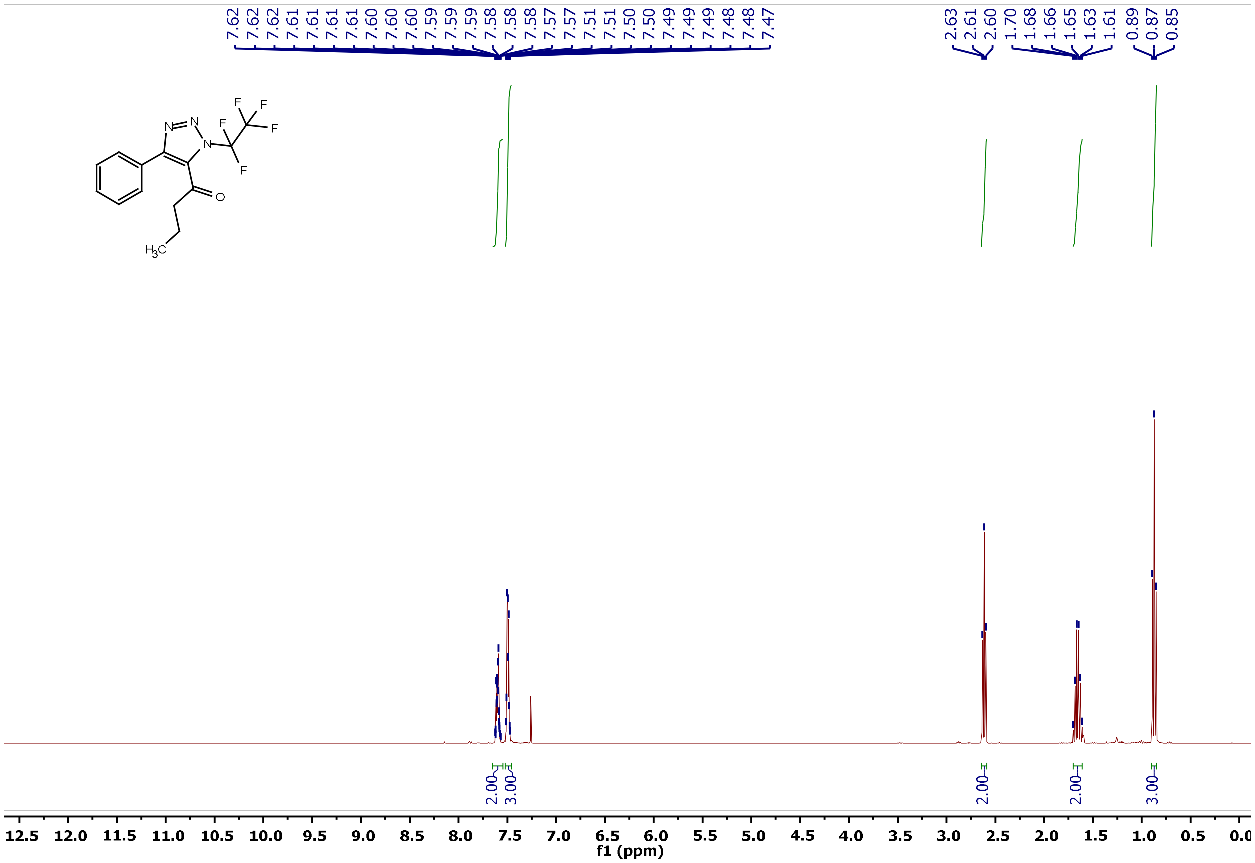

Figure S46. ^{13}C NMR spectrum of **3p** ($CDCl_3$, 101 MHz)

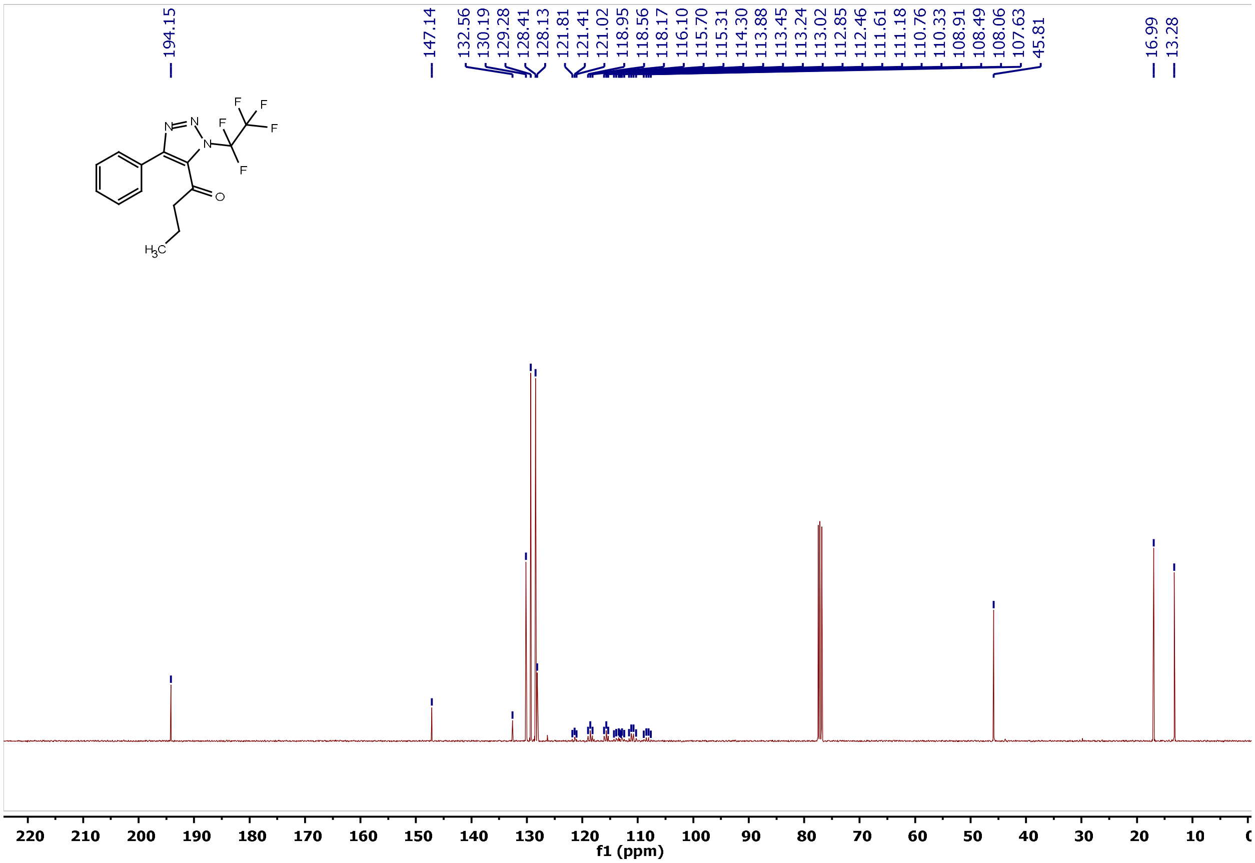

Figure S47. ^{19}F NMR spectrum of **3p** ($CDCl_3$, 377 MHz)

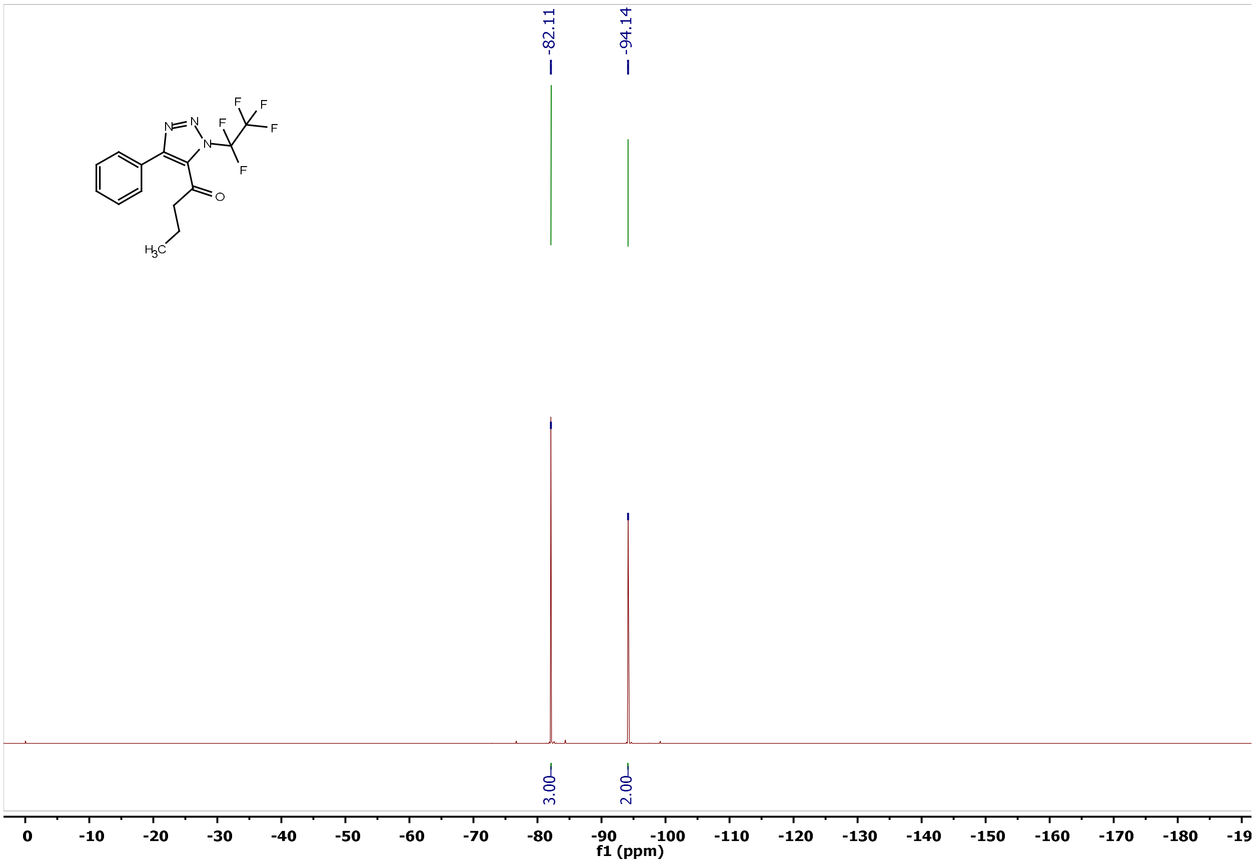

Figure S48. ^{1}H NMR spectrum of **3q** ($CDCl_3$, 401 MHz)

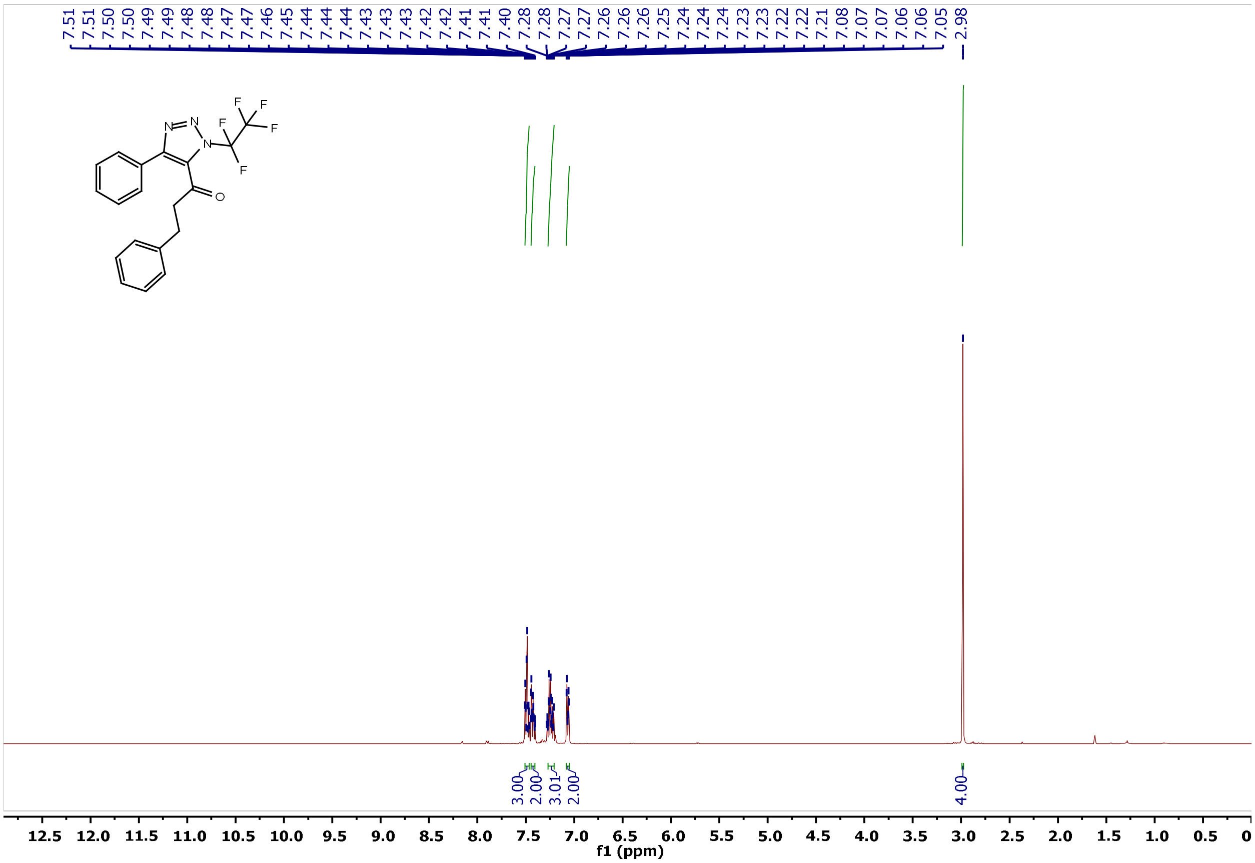

Figure S49. ^{13}C NMR spectrum of **3q** ($CDCl_3$, 101 MHz)

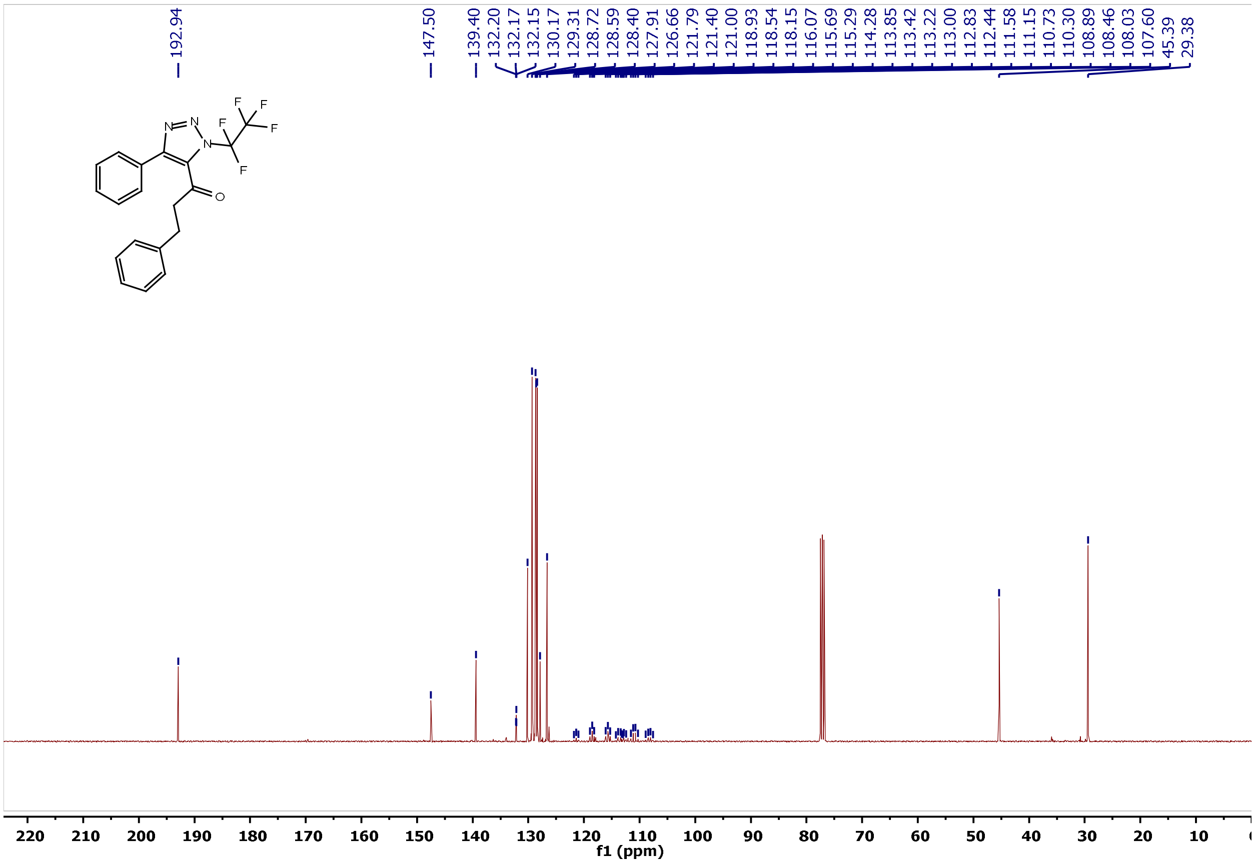

Figure S50. ^{19}F NMR spectrum of **3q** ($CDCl_3$, 377 MHz)

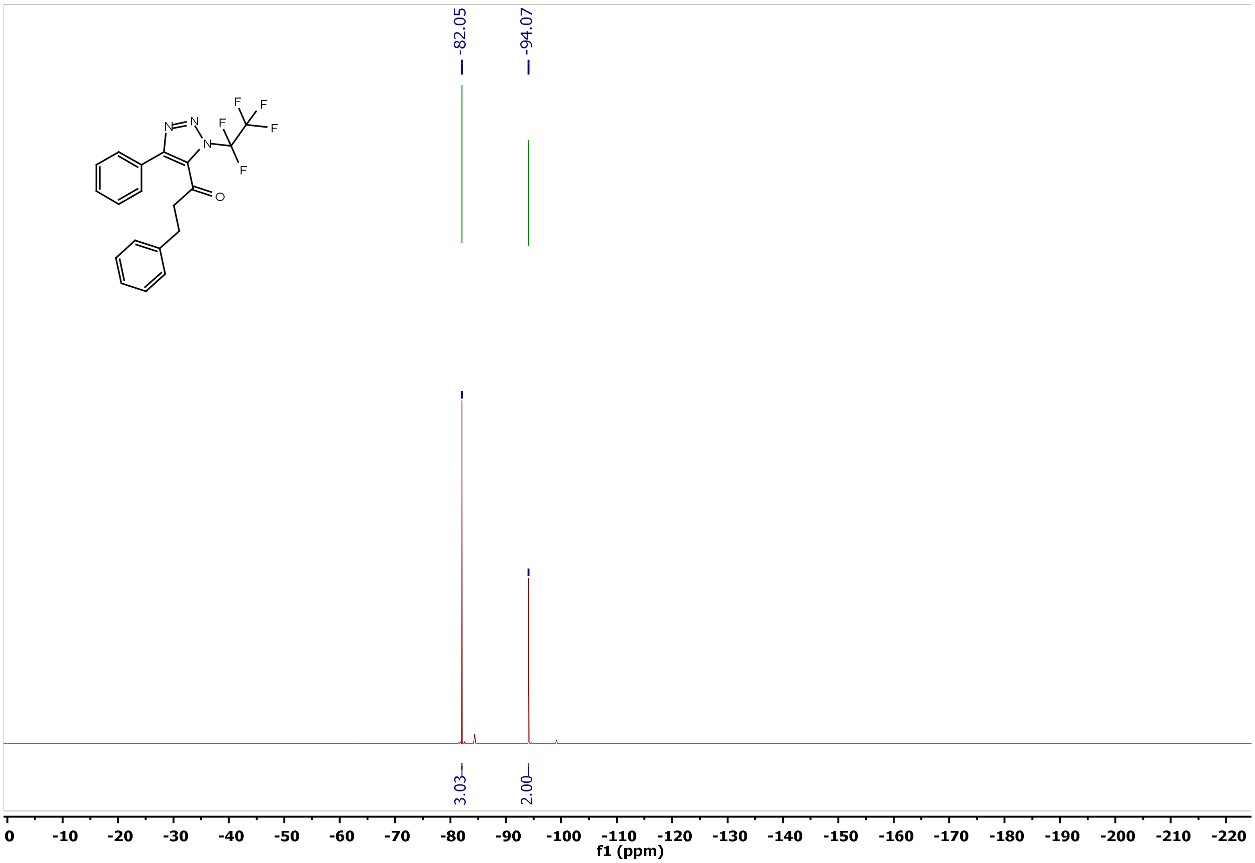

Figure S51. ^{1}H NMR spectrum of **3r** ($CDCl_3$, 401 MHz)

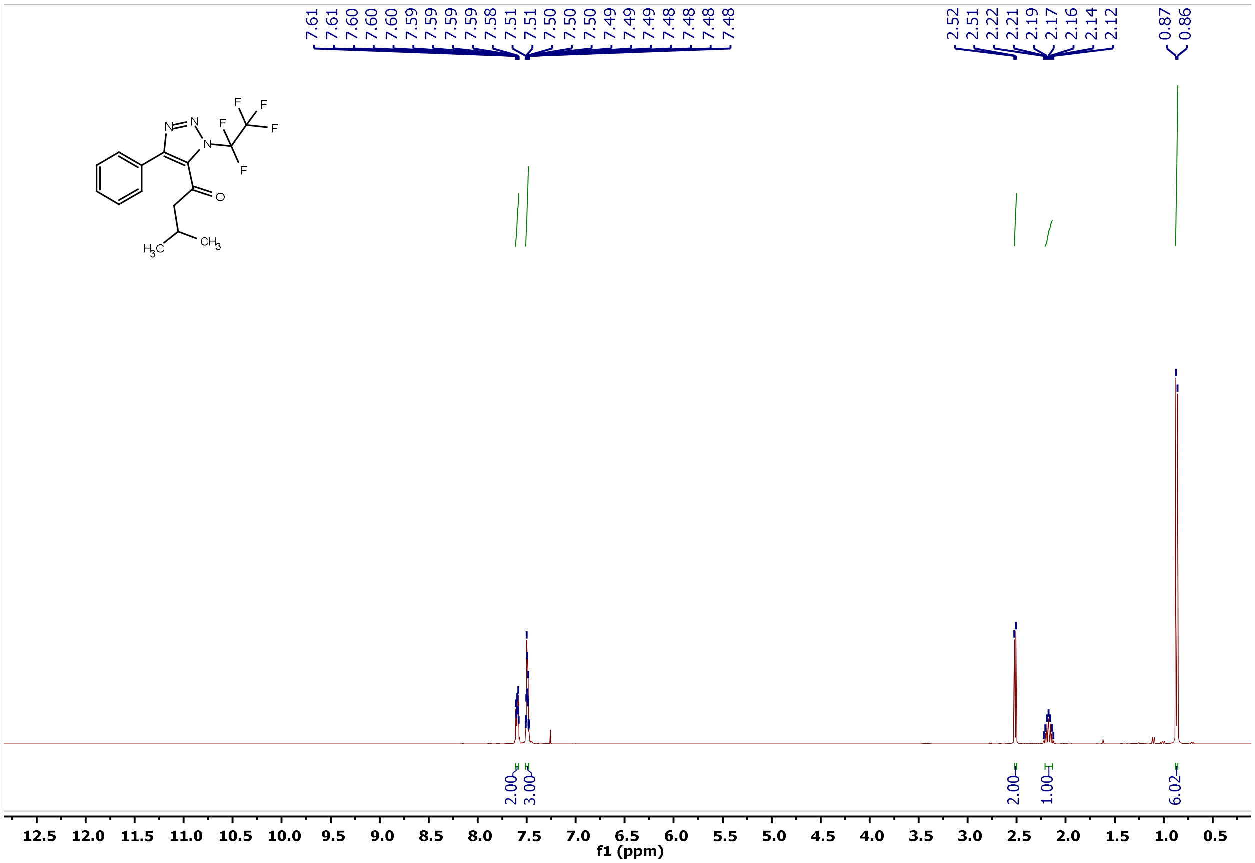

Figure S52. ^{13}C NMR spectrum of **3r** ($CDCl_3$, 101 MHz)

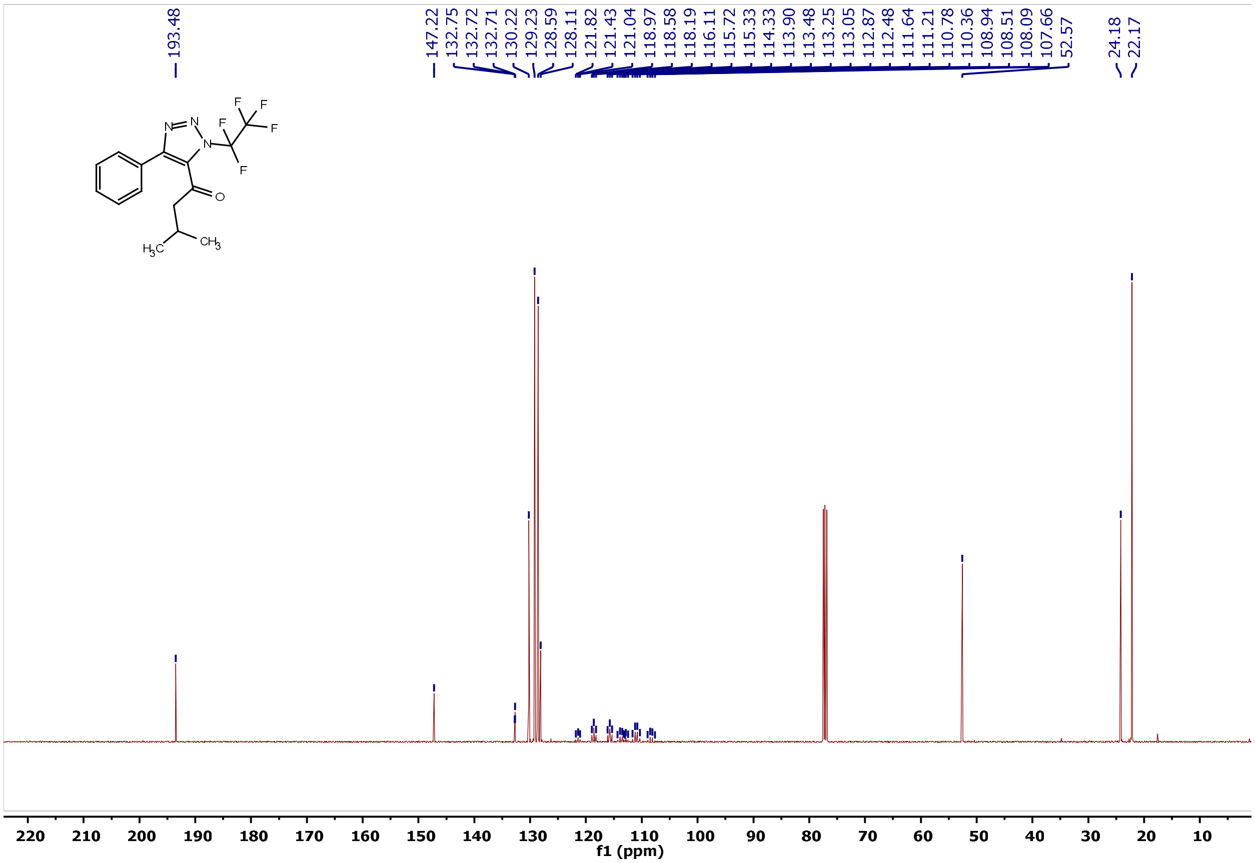

Figure S53. ^{19}F NMR spectrum of **3r** ($CDCl_3$, 377 MHz)

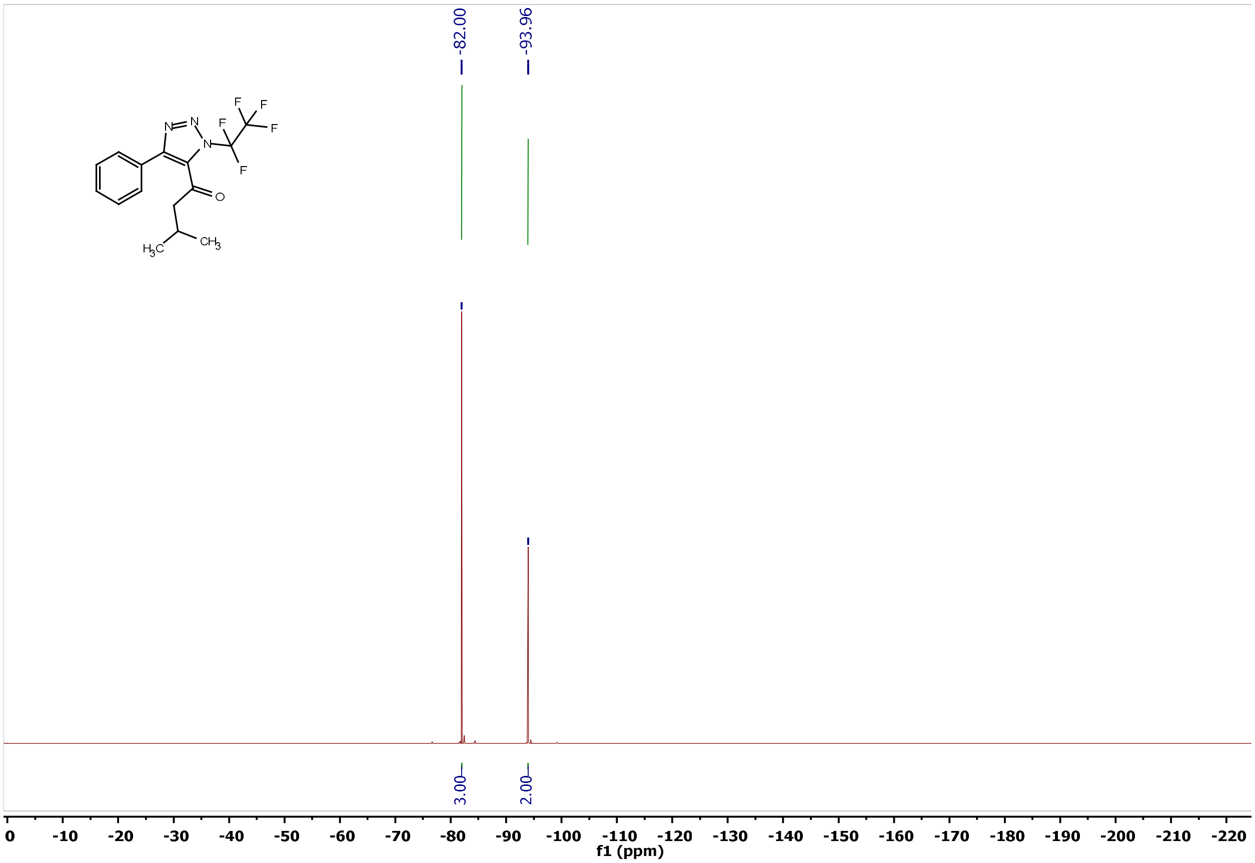

Figure S54. ^{1}H NMR spectrum of **3s** ($CDCl_3$, 401 MHz)

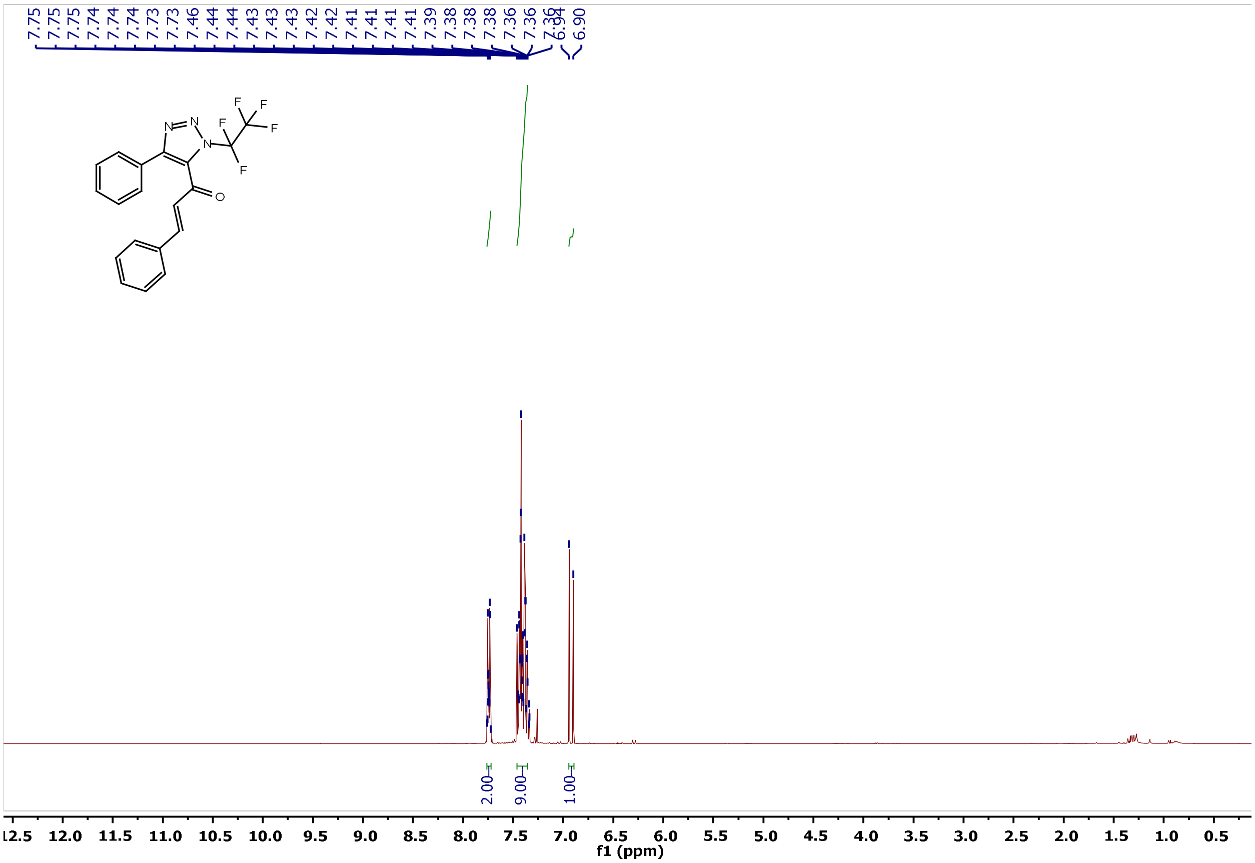

Figure S55. ^{13}C NMR spectrum of **3s** ($CDCl_3$, 101 MHz)

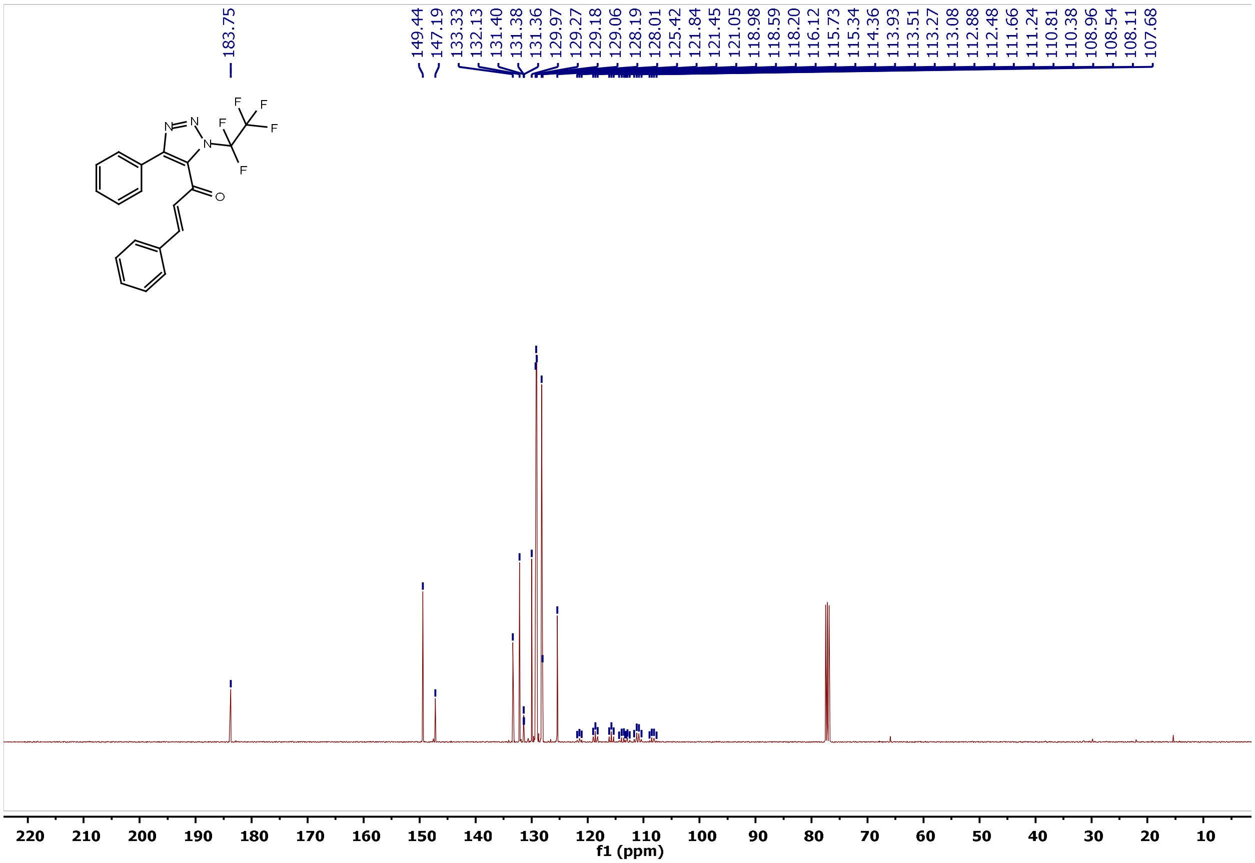

Figure S56. ^{19}F NMR spectrum of **3s** ($CDCl_3$, 377 MHz)

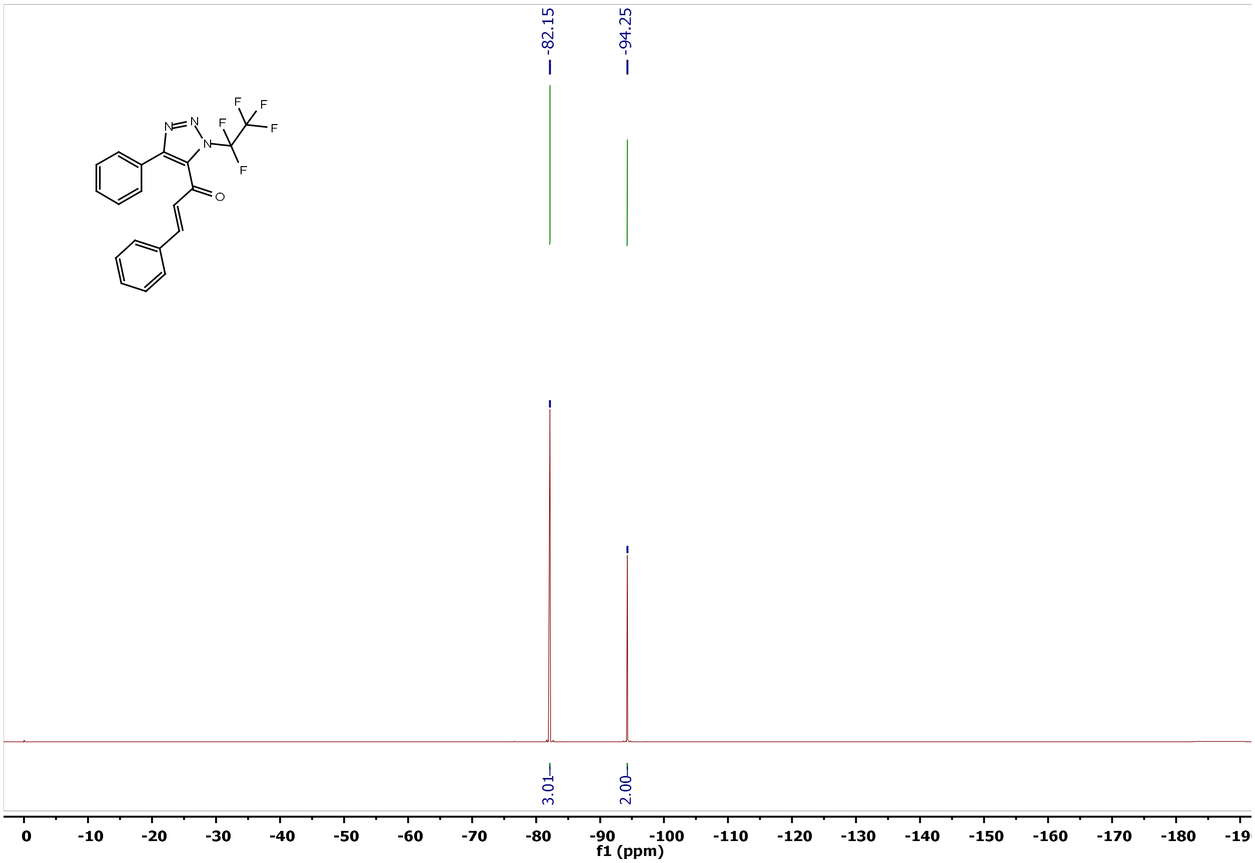

Figure S57. ^{1}H NMR spectrum of **3t** ($CDCl_3$, 401 MHz)

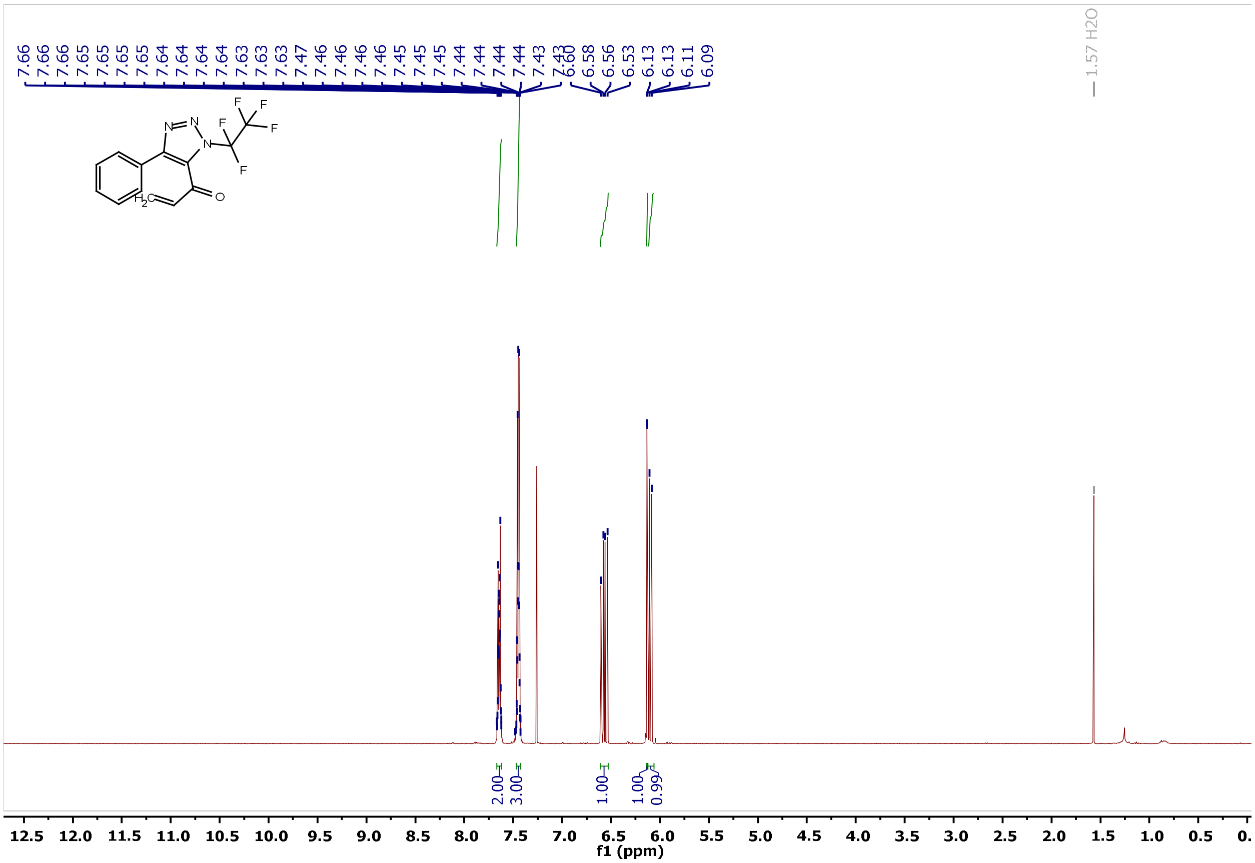

Figure S58. ^{13}C NMR spectrum of **3t** ($CDCl_3$, 101 MHz)

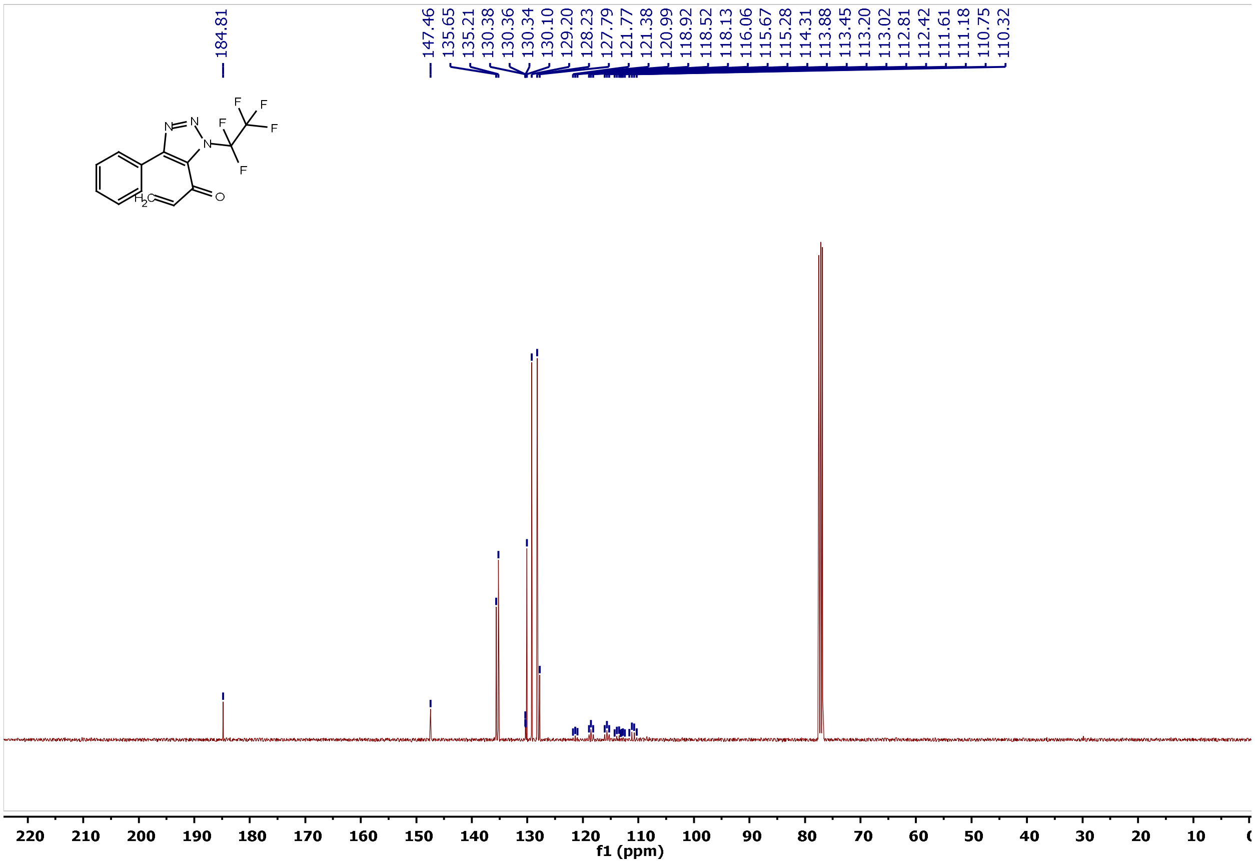

Figure S59. ^{19}F NMR spectrum of **3t** ($CDCl_3$, 377 MHz)

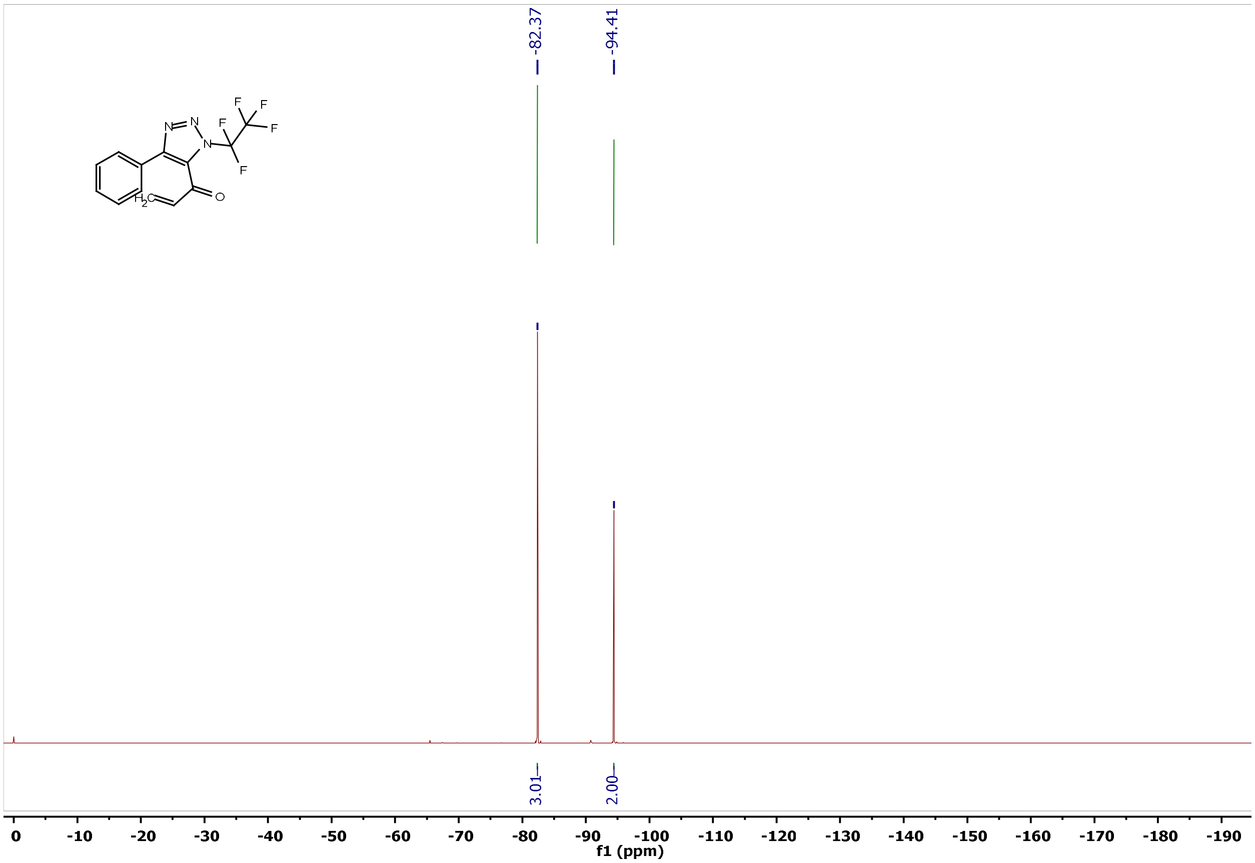

Figure S60. ^1H NMR spectrum of **3u** ($CDCl_3$, 401 MHz)

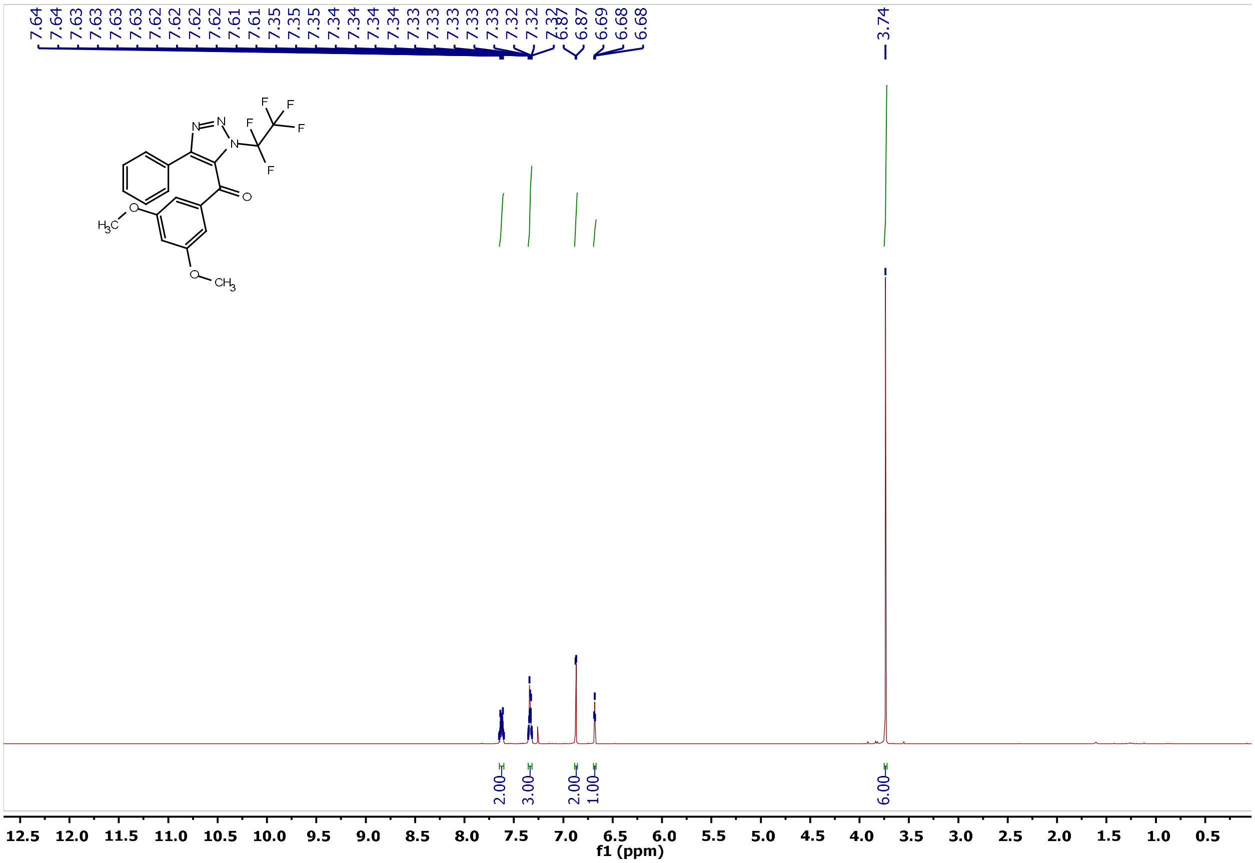

Figure S61. ^{13}C NMR spectrum of **3u** ($CDCl_3$, 101 MHz)

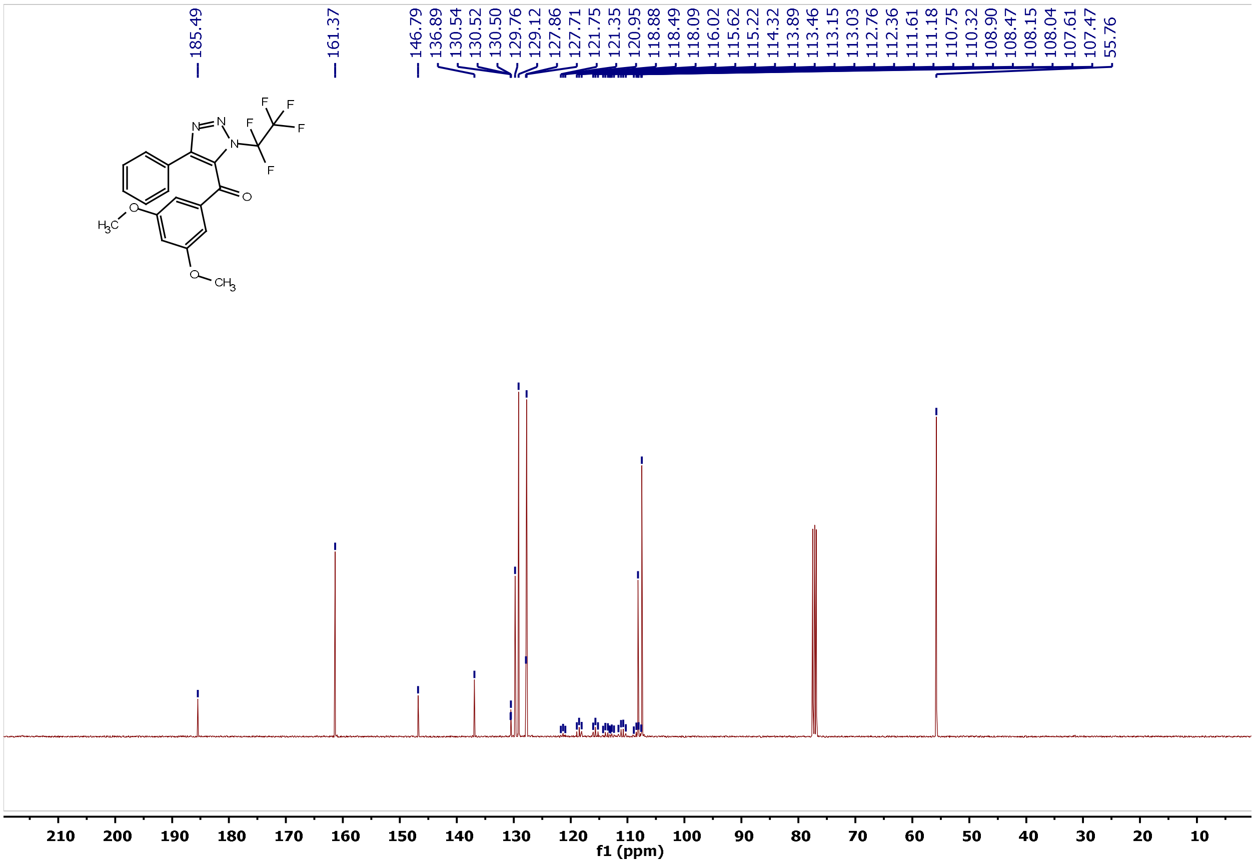

Figure S62. ^{19}F NMR spectrum of **3u** ($CDCl_3$, 377 MHz)

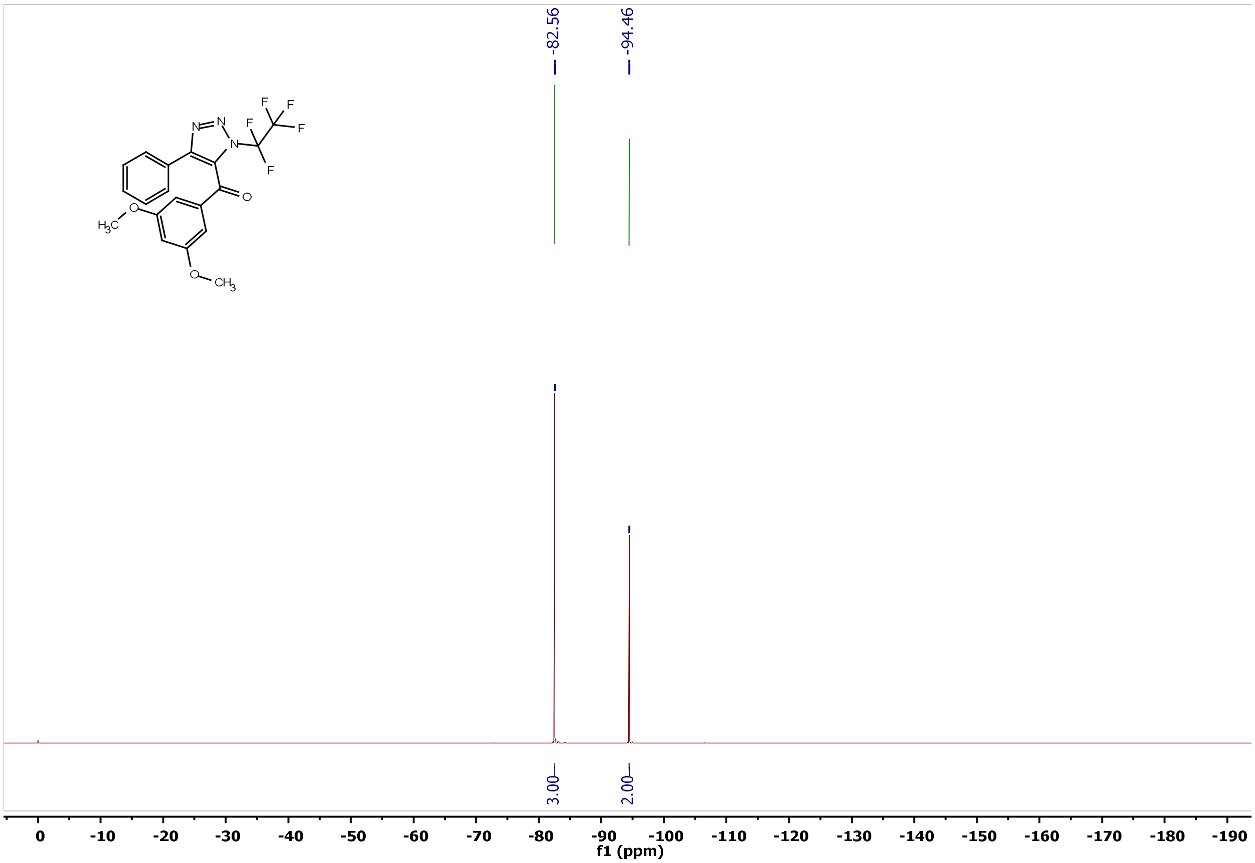

Figure S63. ^{1}H NMR spectrum of **3v** ($CDCl_3$, 401 MHz)

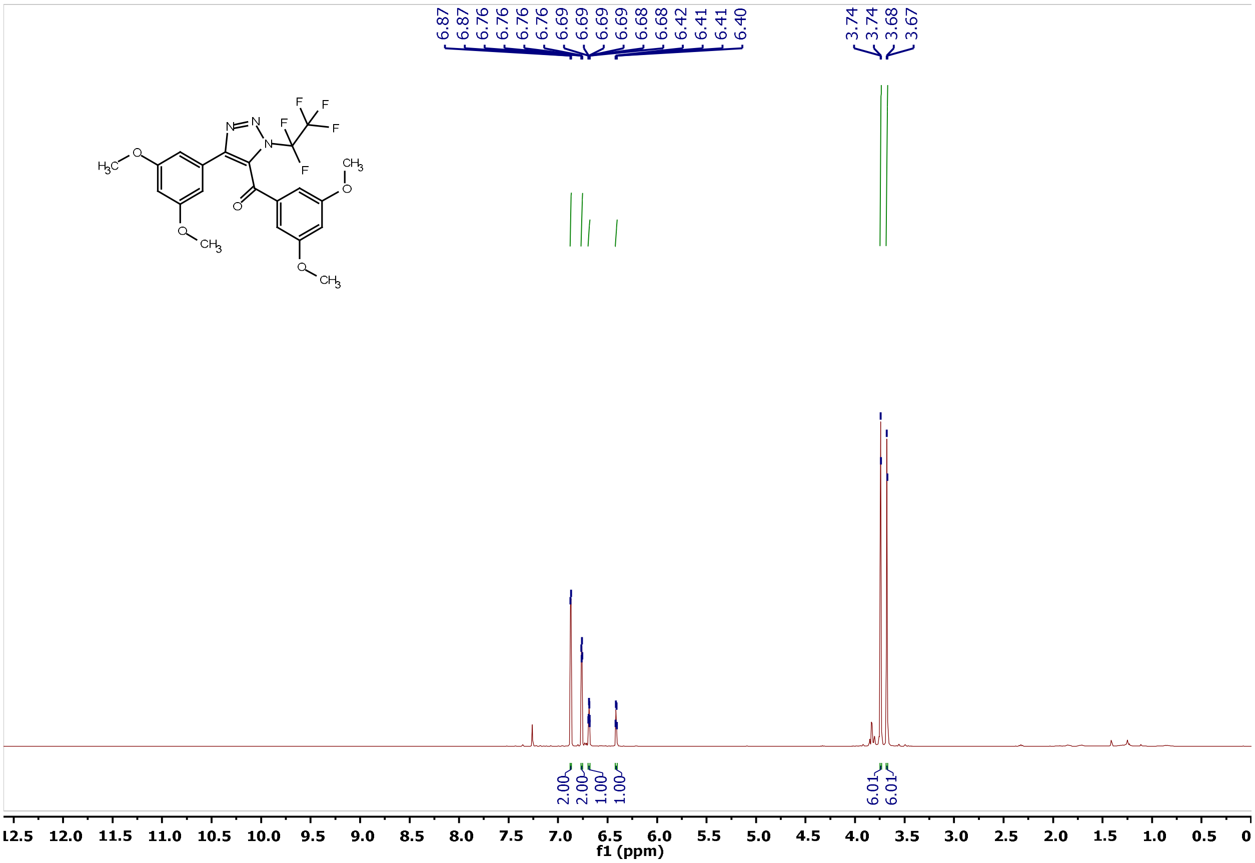

Figure S64. ^{13}C NMR spectrum of **3v** ($CDCl_3$, 101 MHz)

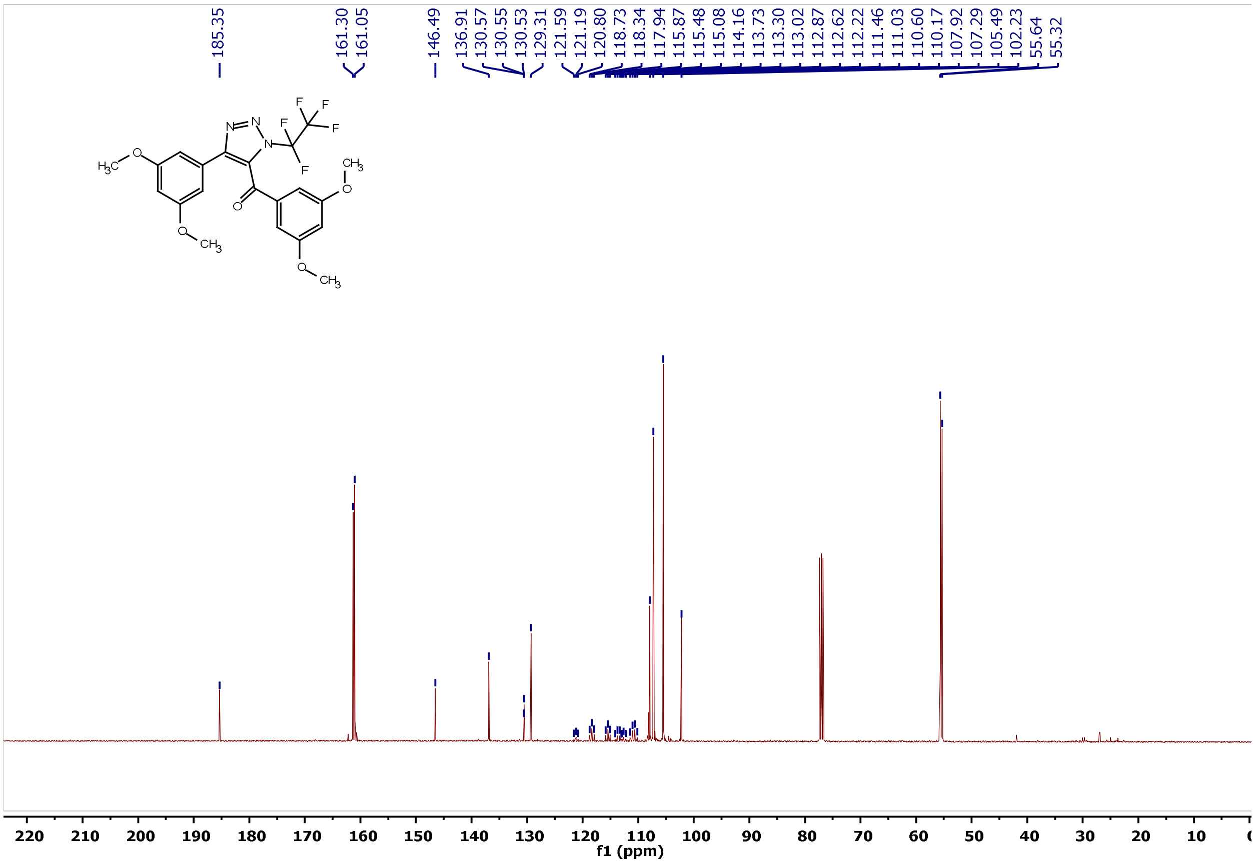

Figure S65. ^{19}F NMR spectrum of **3v** ($CDCl_3$, 377 MHz)

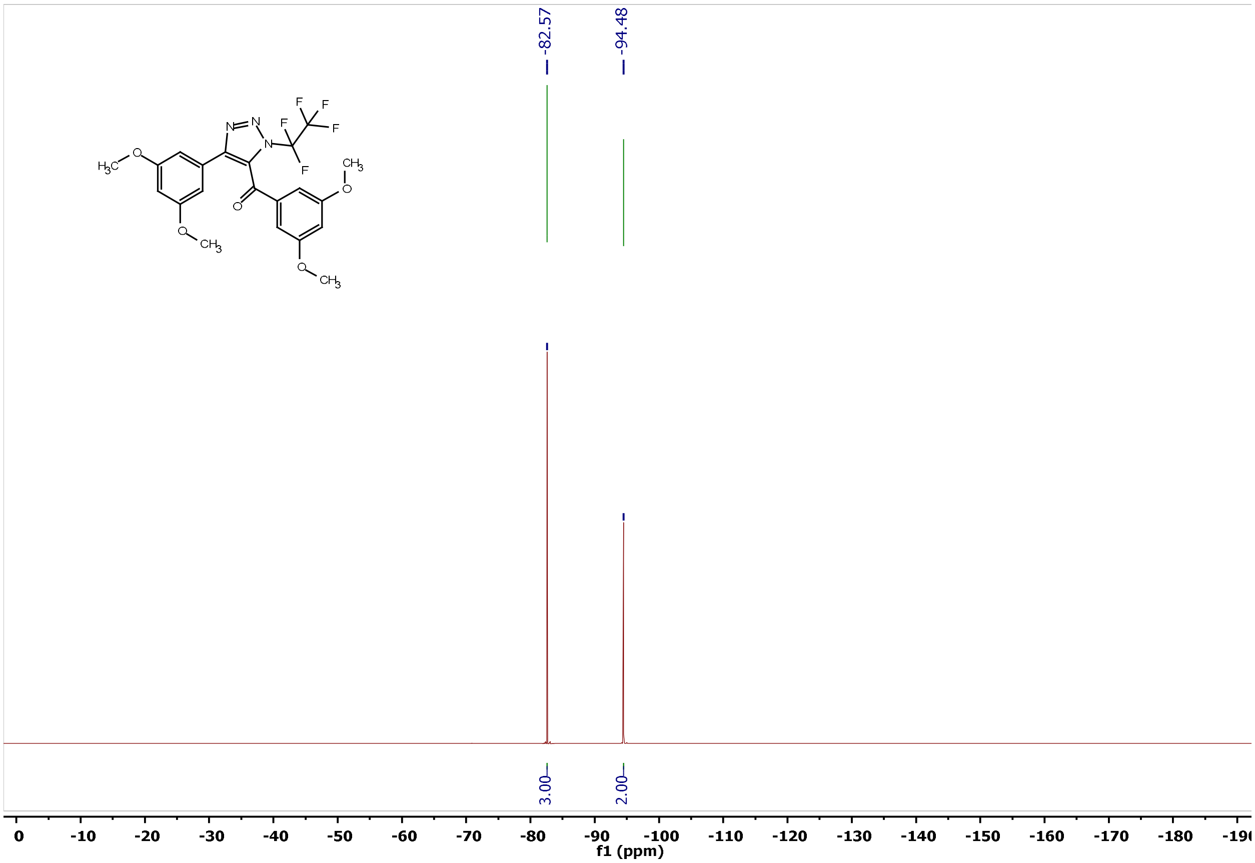

Figure S66. ^1H NMR spectrum of **3w** ($CDCl_3$, 401 MHz)

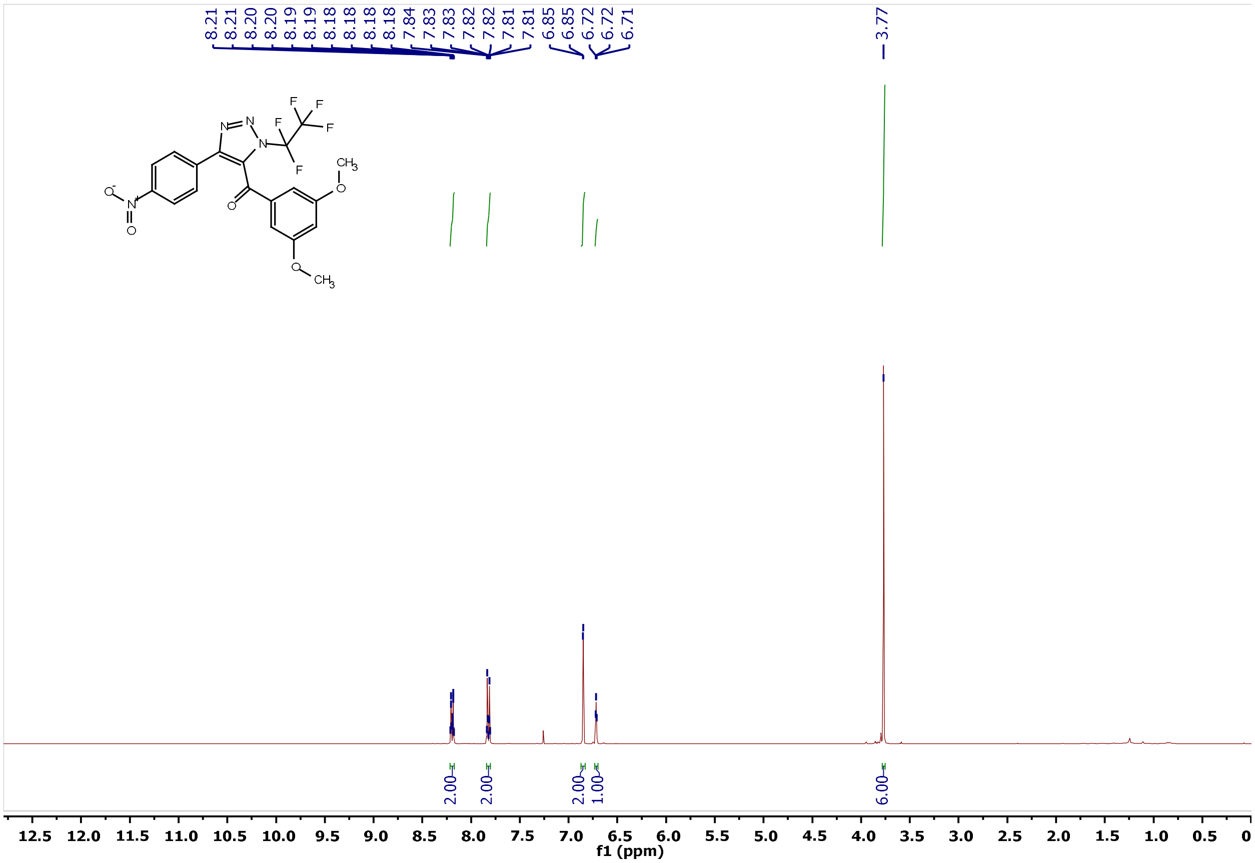

Figure S67. ^{13}C NMR spectrum of **3w** ($CDCl_3$, 101 MHz)

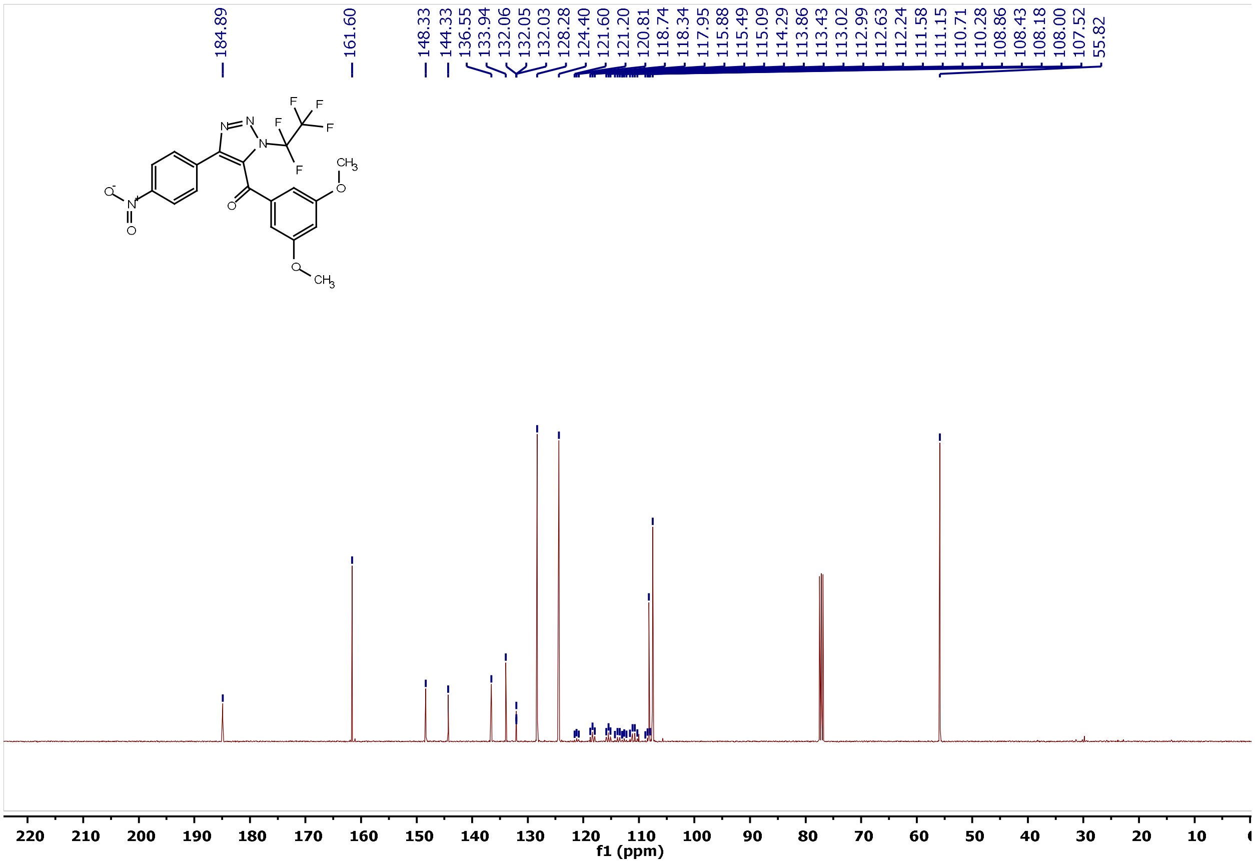

Figure S68. ^{19}F NMR spectrum of **3w** ($CDCl_3$, 377 MHz)

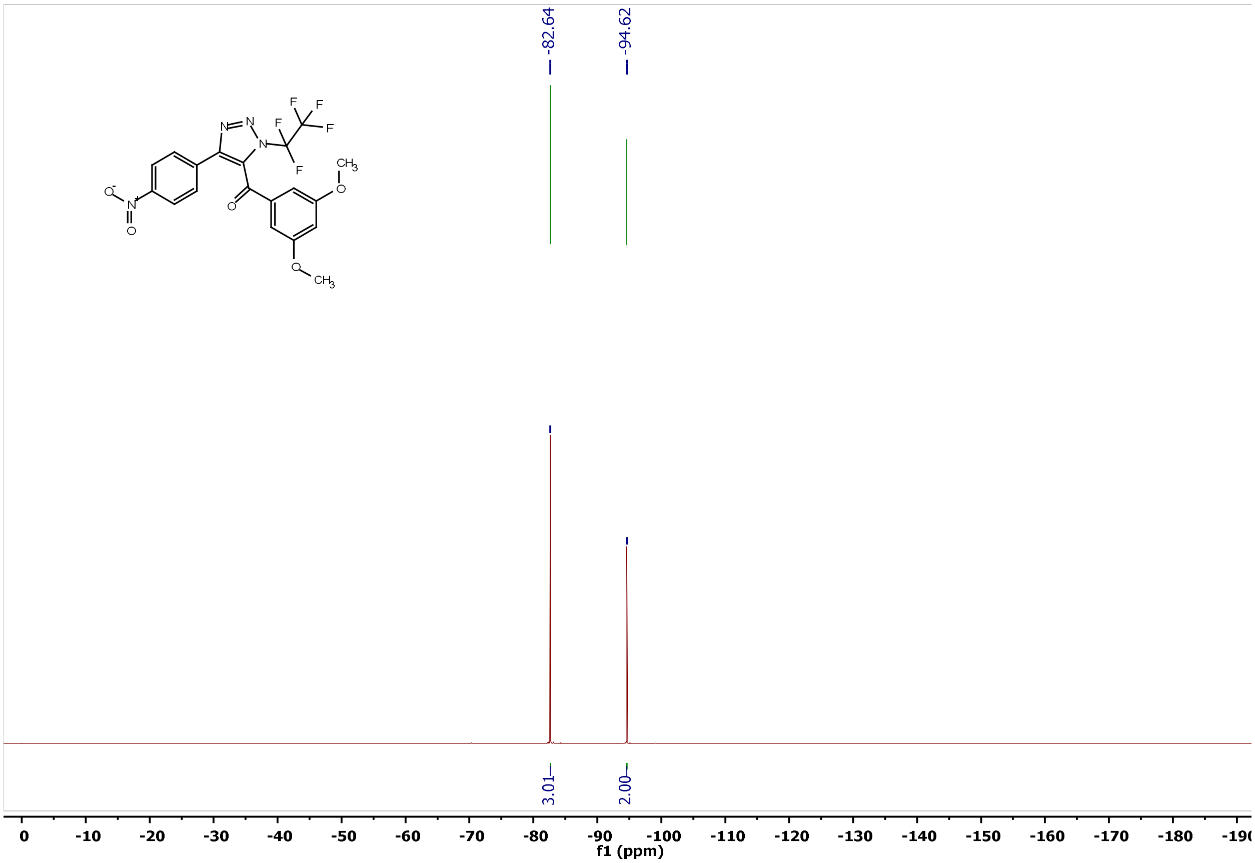

Figure S69. ^{1}H NMR spectrum of **3x** ($CDCl_3$, 401 MHz)

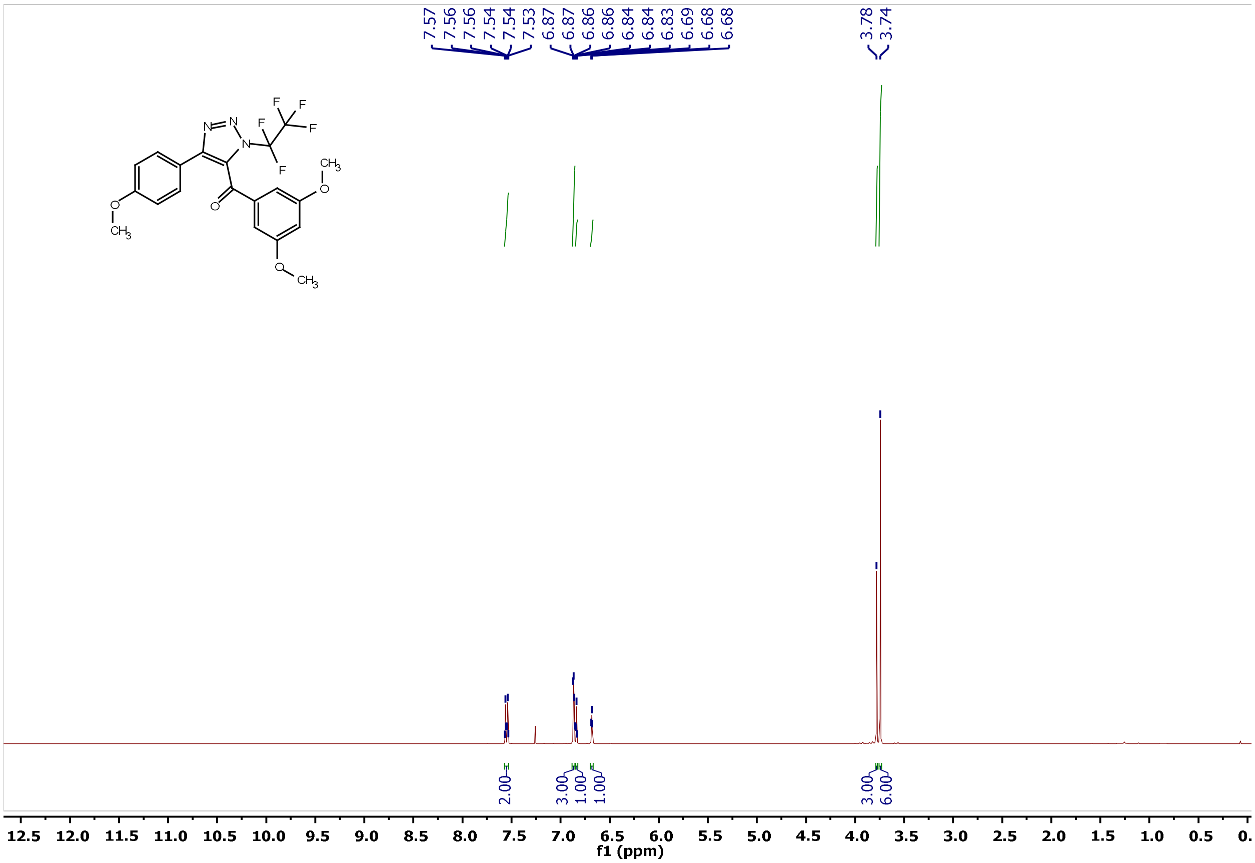

Figure S70. ^{13}C NMR spectrum of **3x** ($CDCl_3$, 101 MHz)

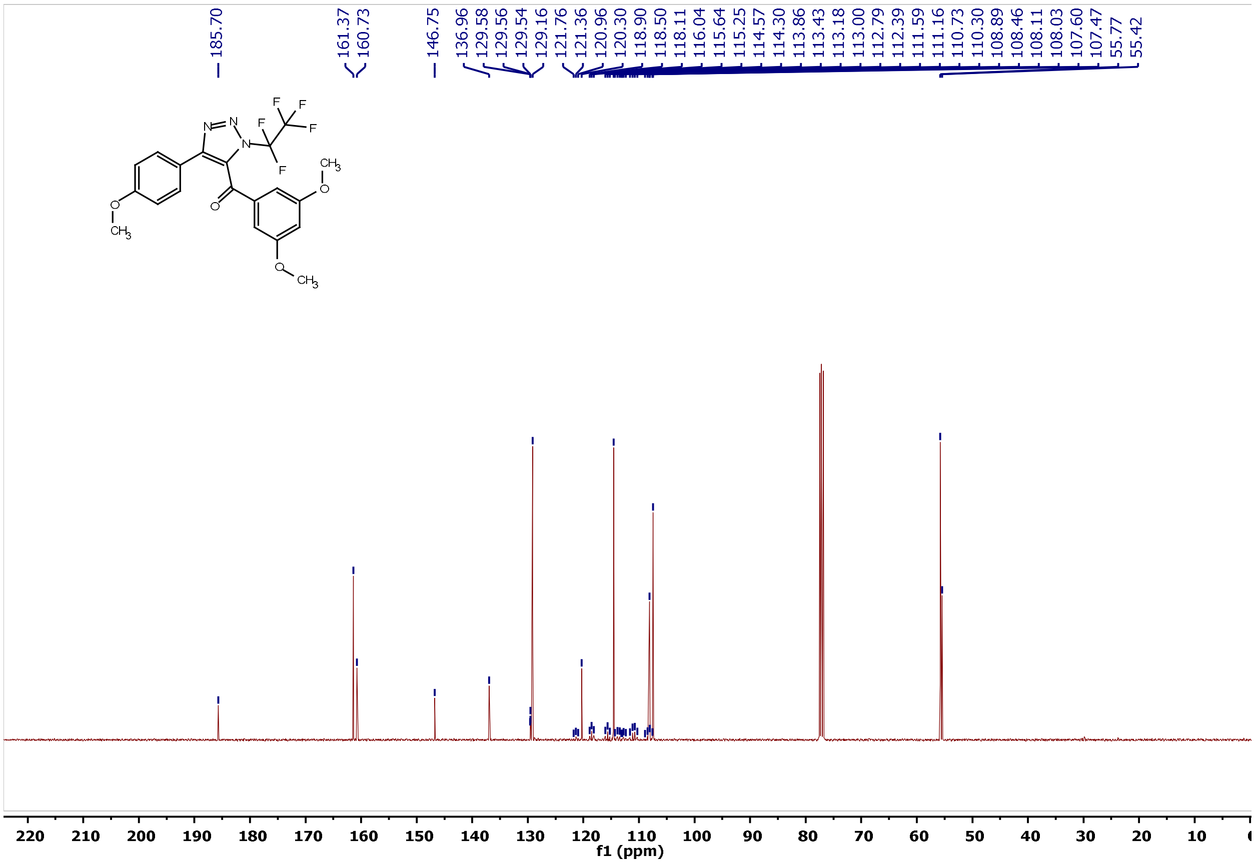

Figure S71. ^{19}F NMR spectrum of **3x** ($CDCl_3$, 377 MHz)

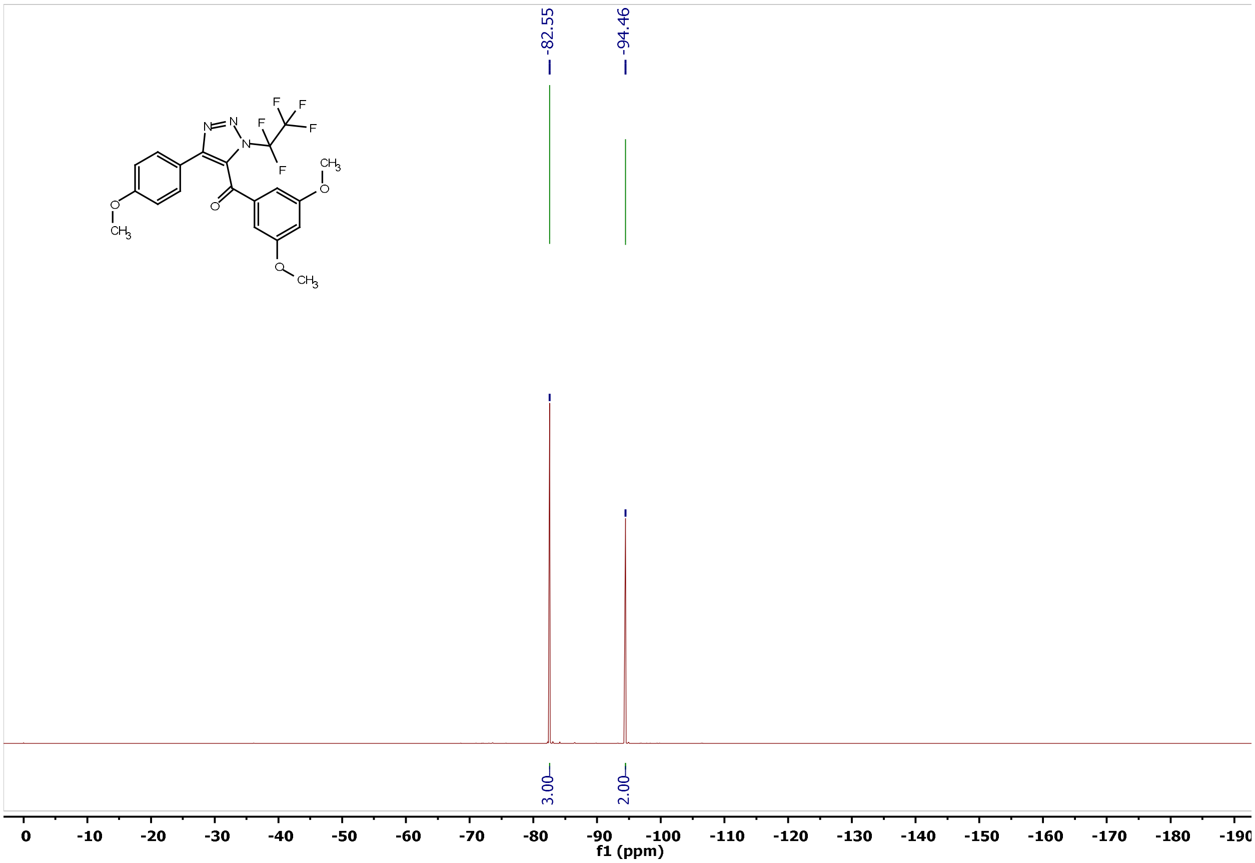

Figure S72. ^{1}H NMR spectrum of **3y** ($CDCl_3$, 401 MHz)

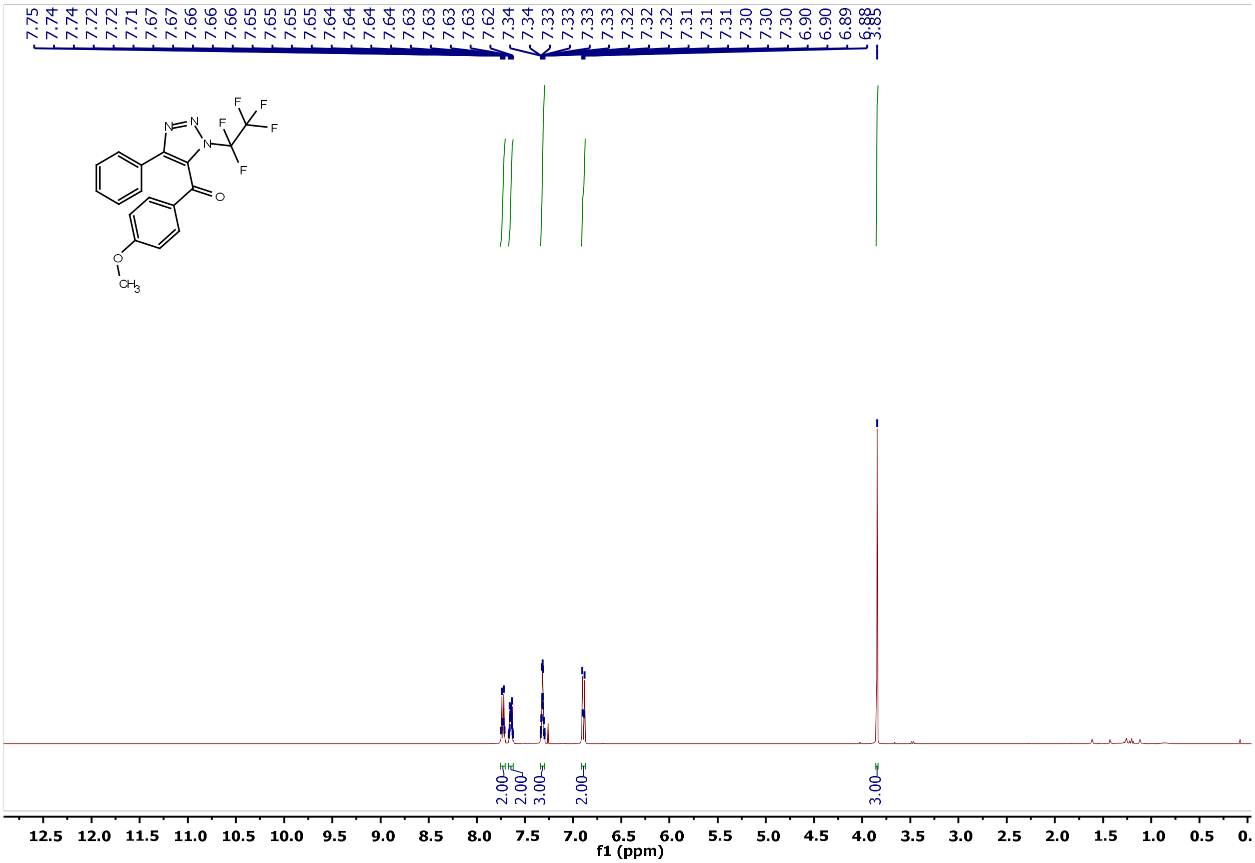

Figure S73. ^{13}C NMR spectrum of **3y** ($CDCl_3$, 101 MHz)

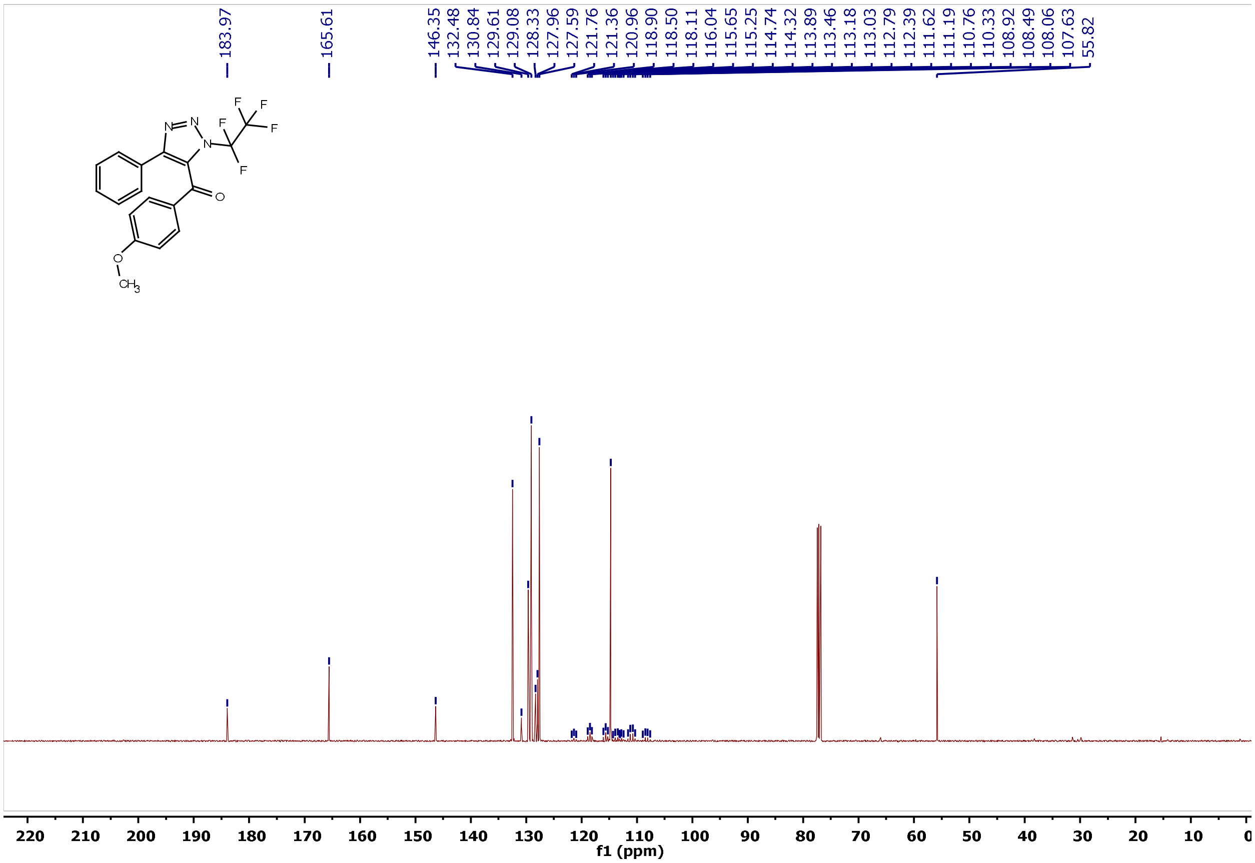

Figure S74. ^{19}F NMR spectrum of **3y** ($CDCl_3$, 377 MHz)

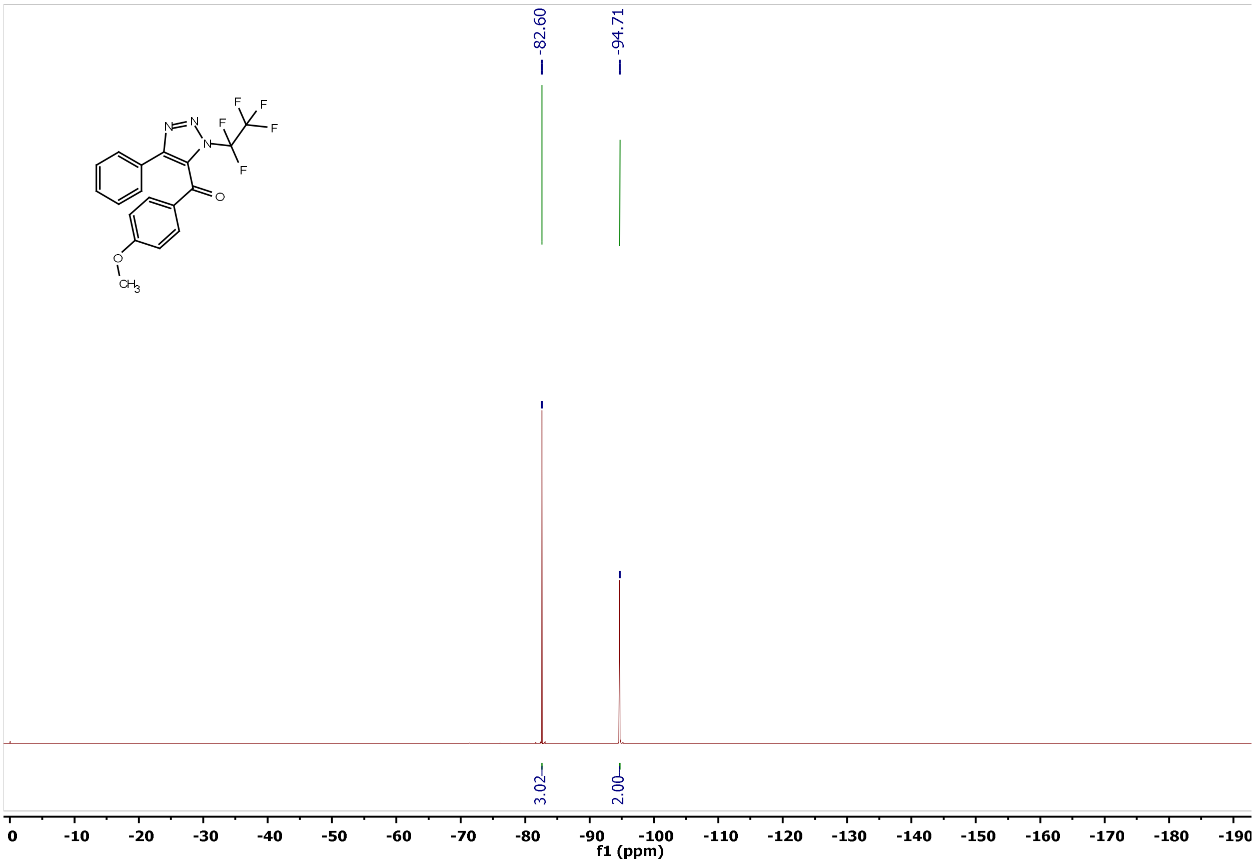

Figure S75. ^{1}H NMR spectrum of **3z** ($CDCl_3$, 401 MHz)

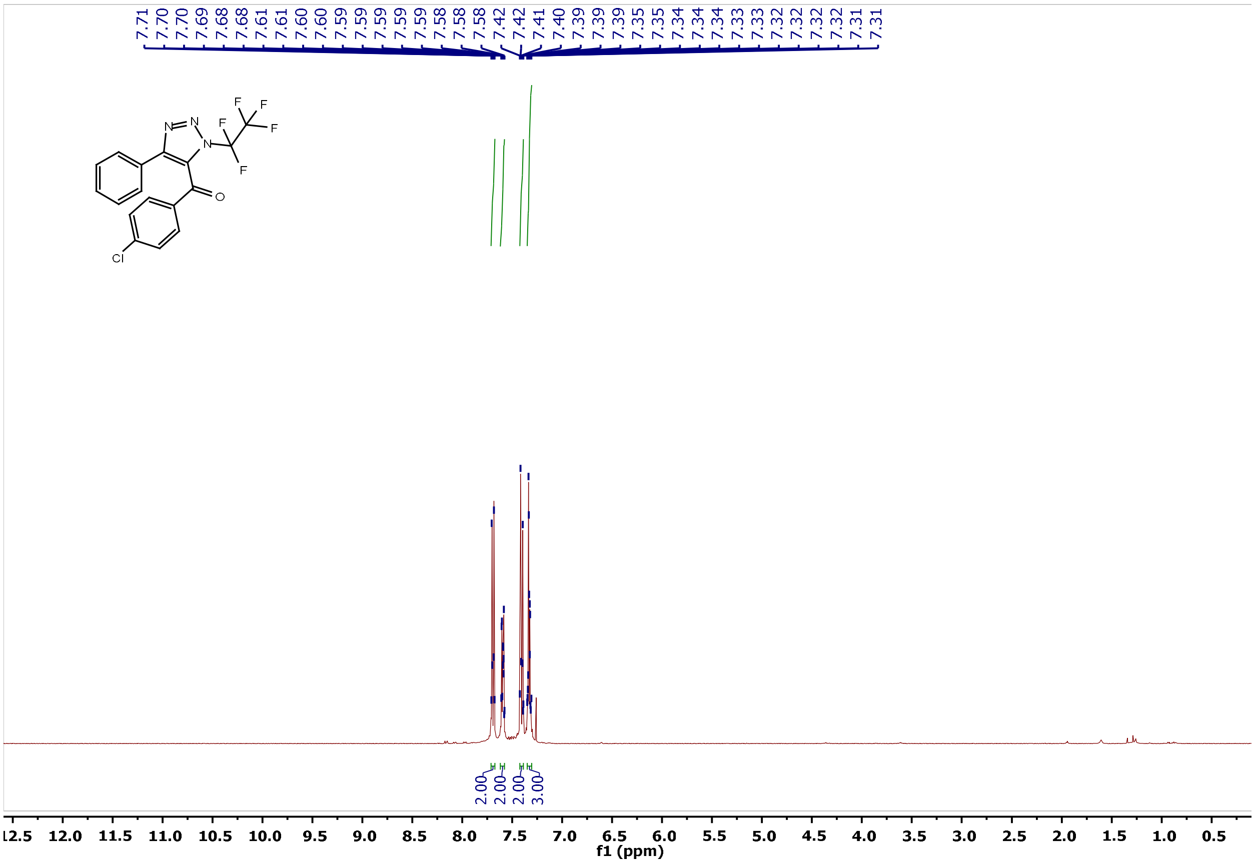

Figure S76. ^{13}C NMR spectrum of **3z** ($CDCl_3$, 101 MHz)

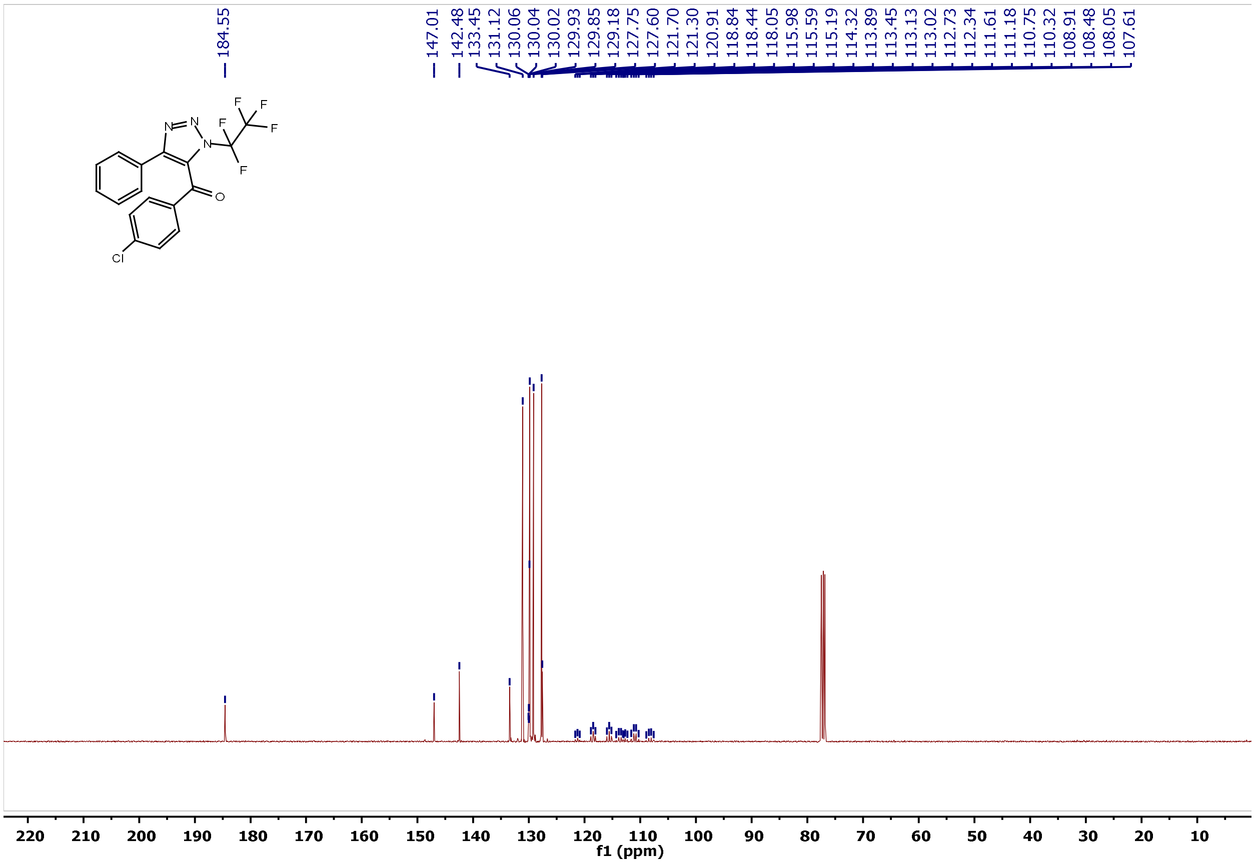

Figure S77. ^{19}F NMR spectrum of **3z** ($CDCl_3$, 377 MHz)

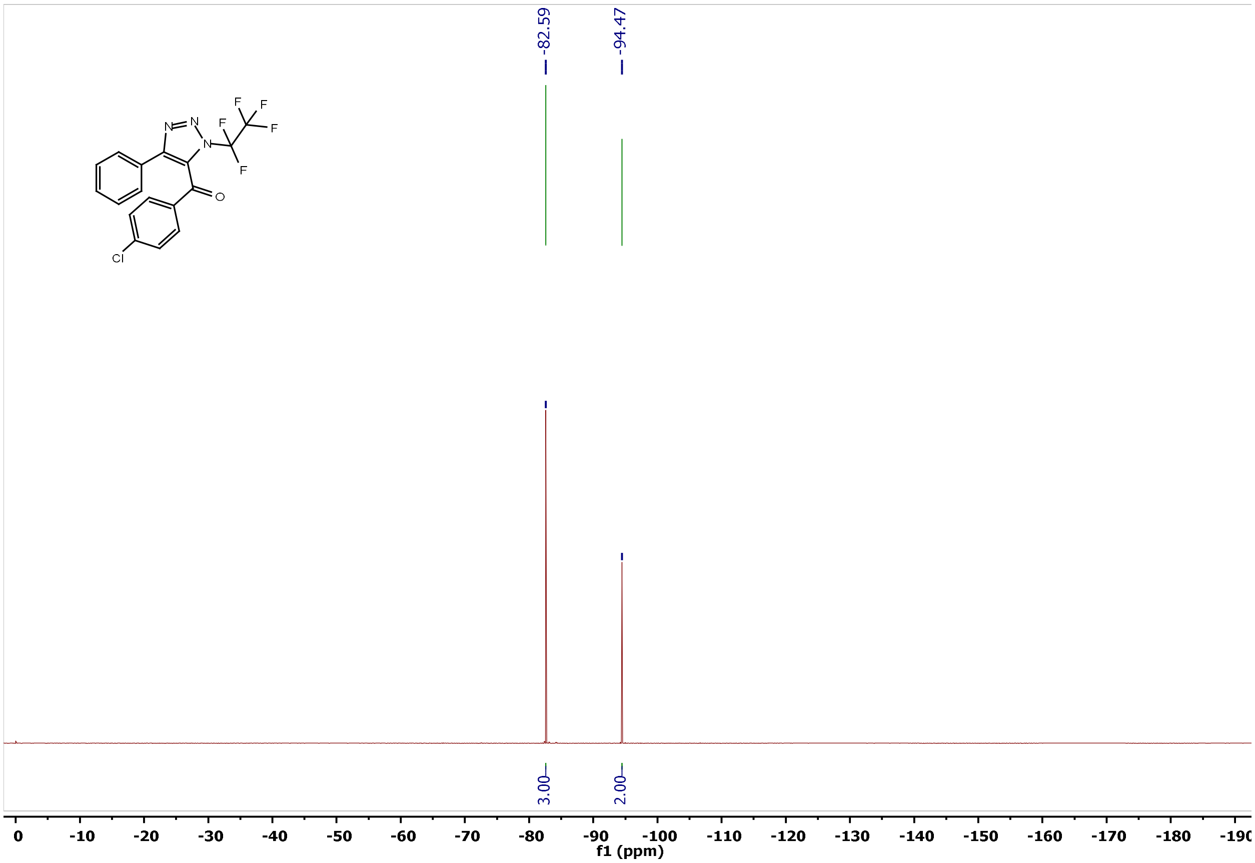

Figure S78. ^{1}H NMR spectrum of **3aa** ($CDCl_3$, 401 MHz)

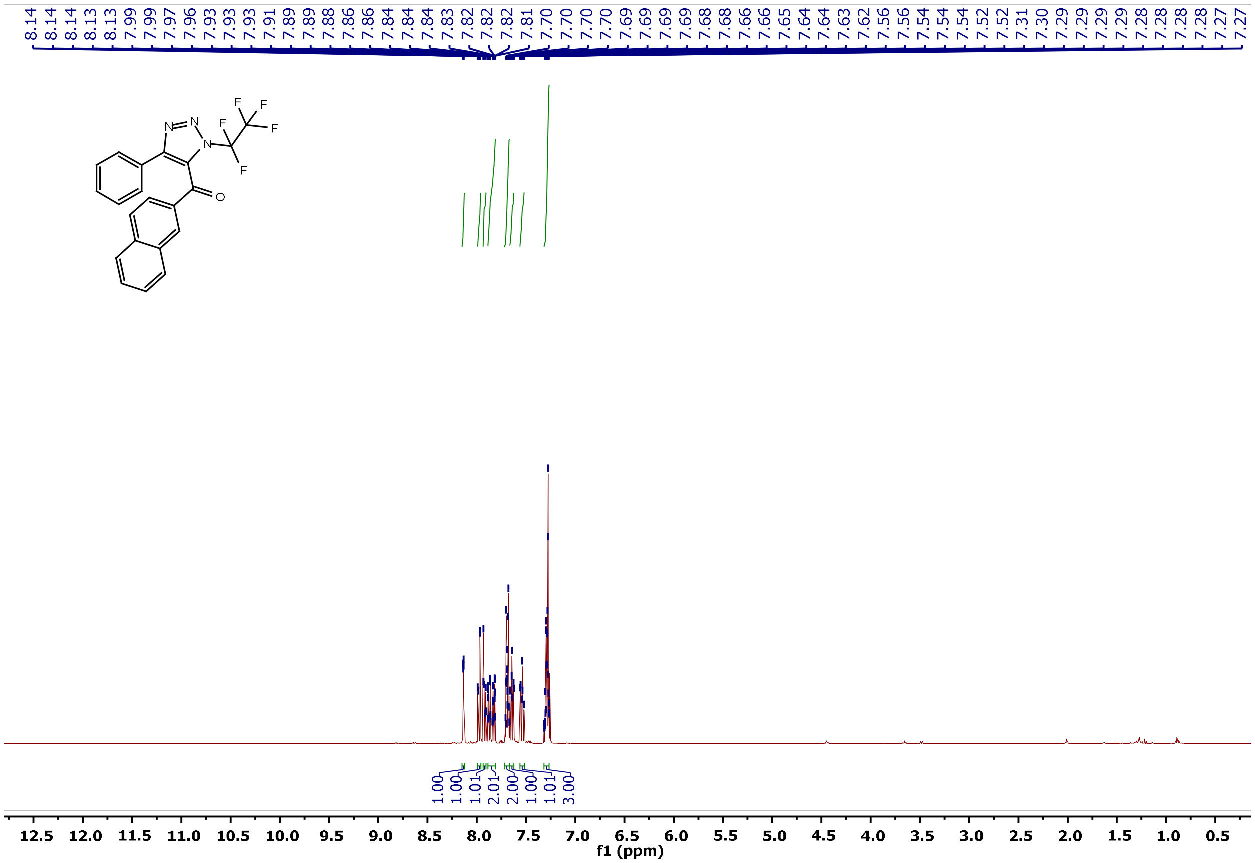

Figure S79. ^{13}C NMR spectrum of **3aa** ($CDCl_3$, 101 MHz)

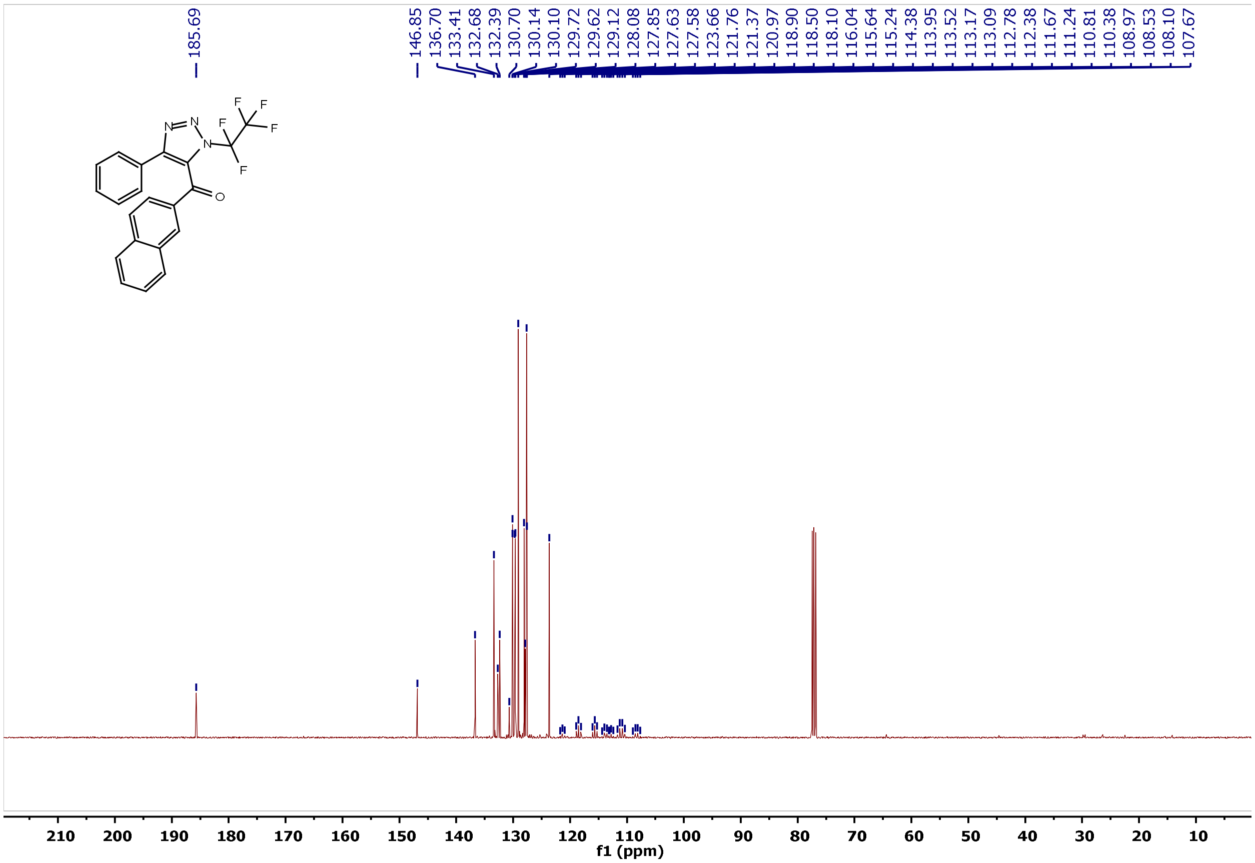

Figure S80. ^{19}F NMR spectrum of **3aa** ($CDCl_3$, 377 MHz)

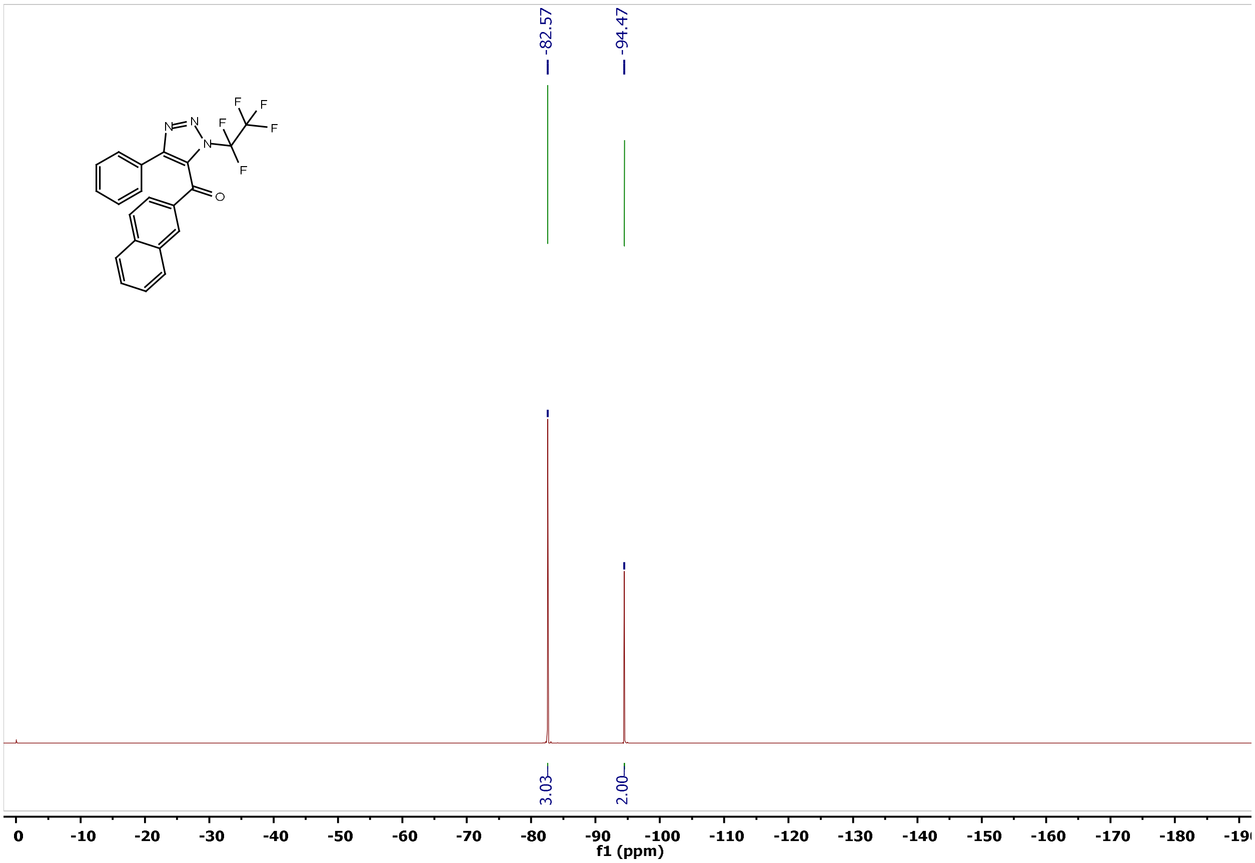

Figure S81. ^{1}H NMR spectrum of **3ab** ($CDCl_3$, 401 MHz)

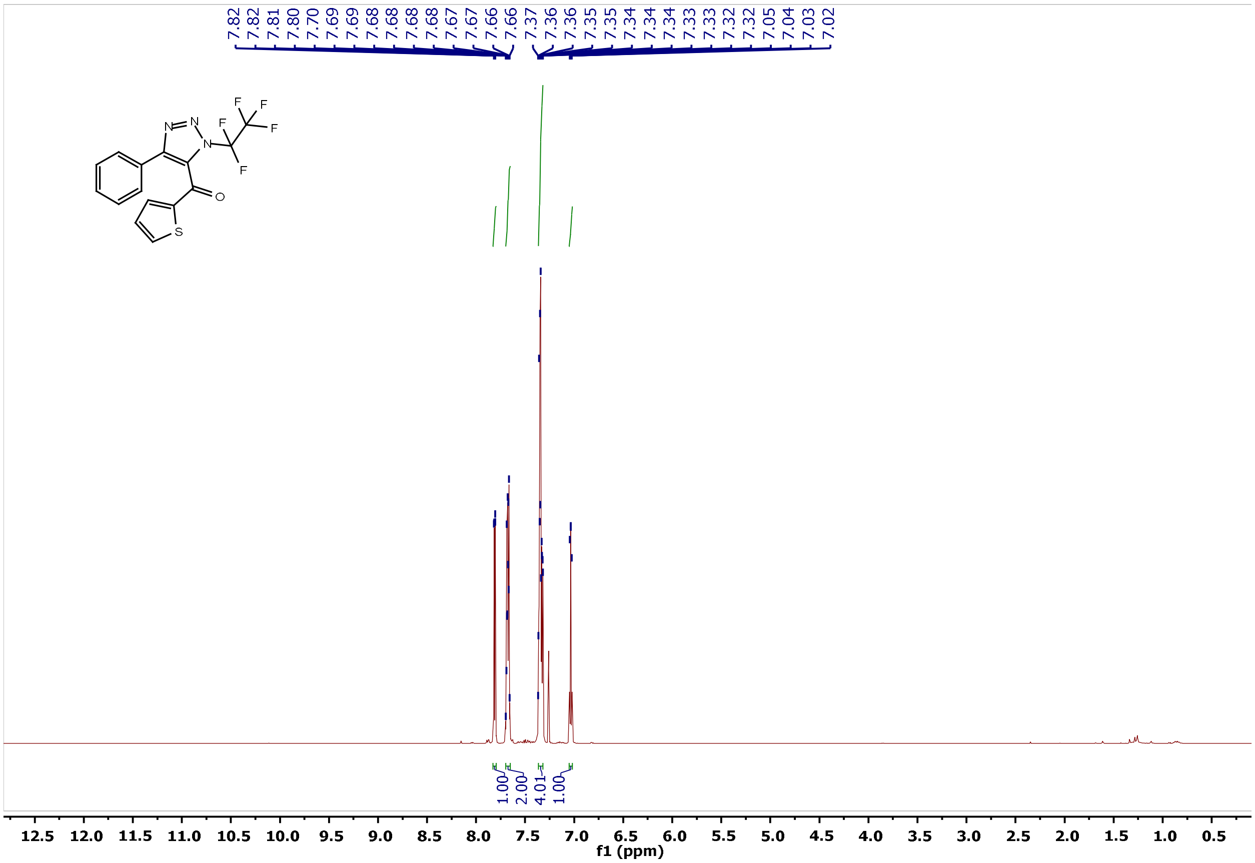

Figure S82. ^{13}C NMR spectrum of **3ab** ($CDCl_3$, 101 MHz)

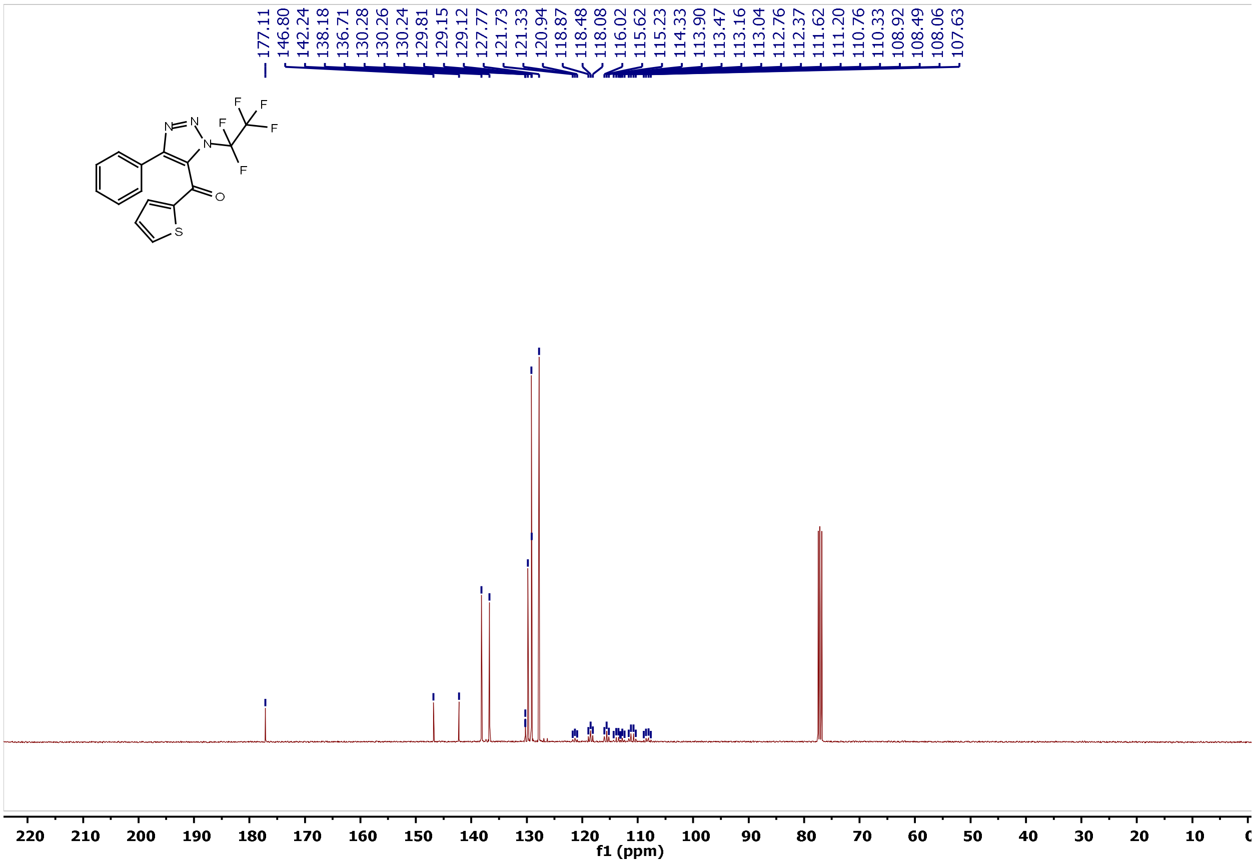

Figure S83. ^{19}F NMR spectrum of **3ab** ($CDCl_3$, 377 MHz)

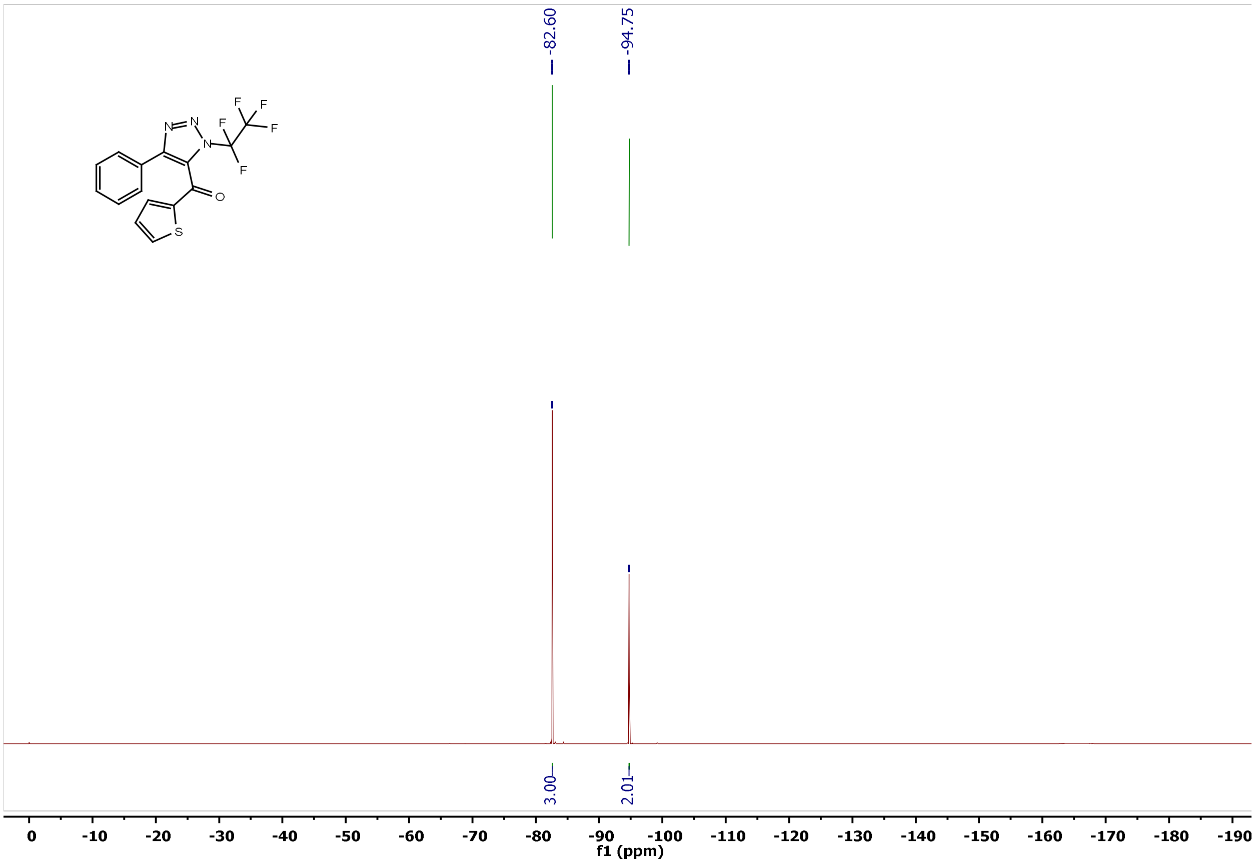

Figure S84. ¹H NMR spectrum of **3ac** ($CDCl_3$, 401 MHz)

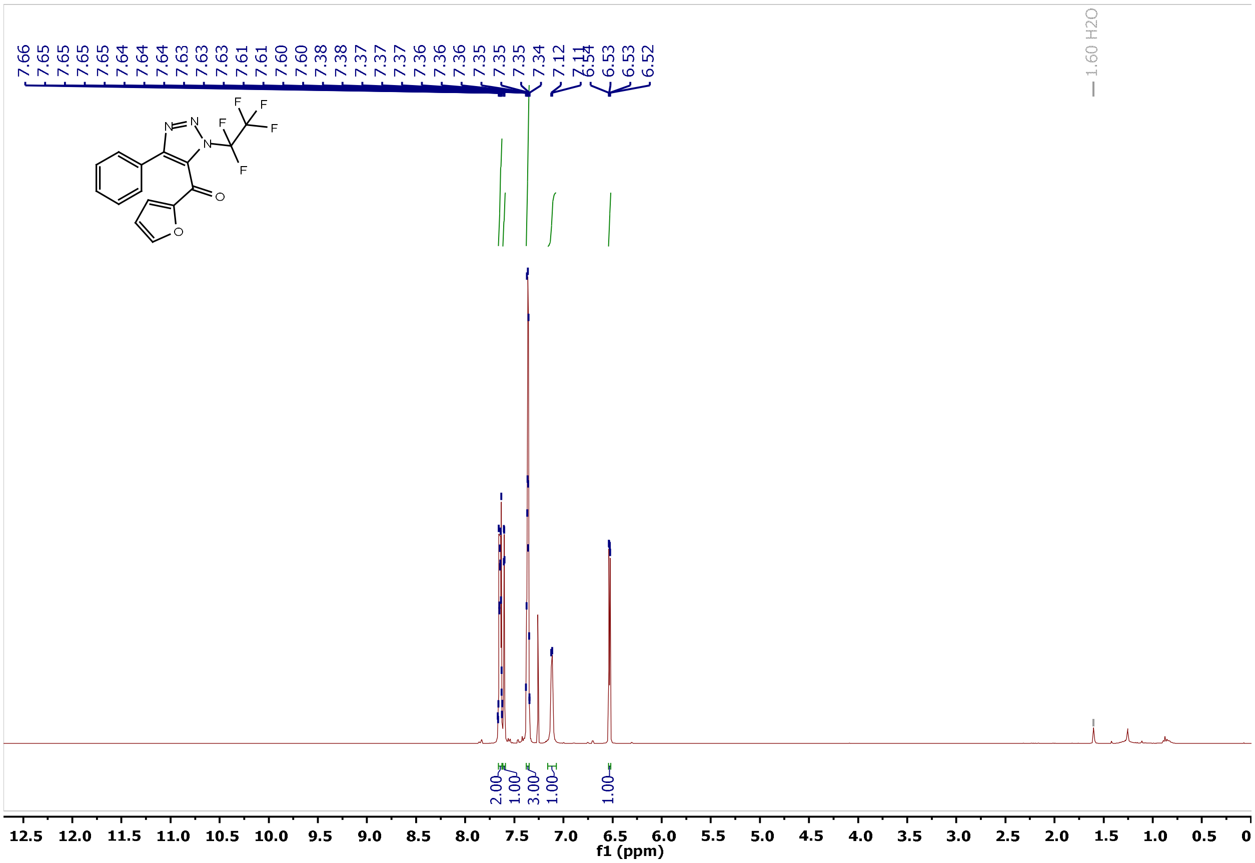

Figure S85. ^{13}C NMR spectrum of **3ac** ($CDCl_3$, 101 MHz)

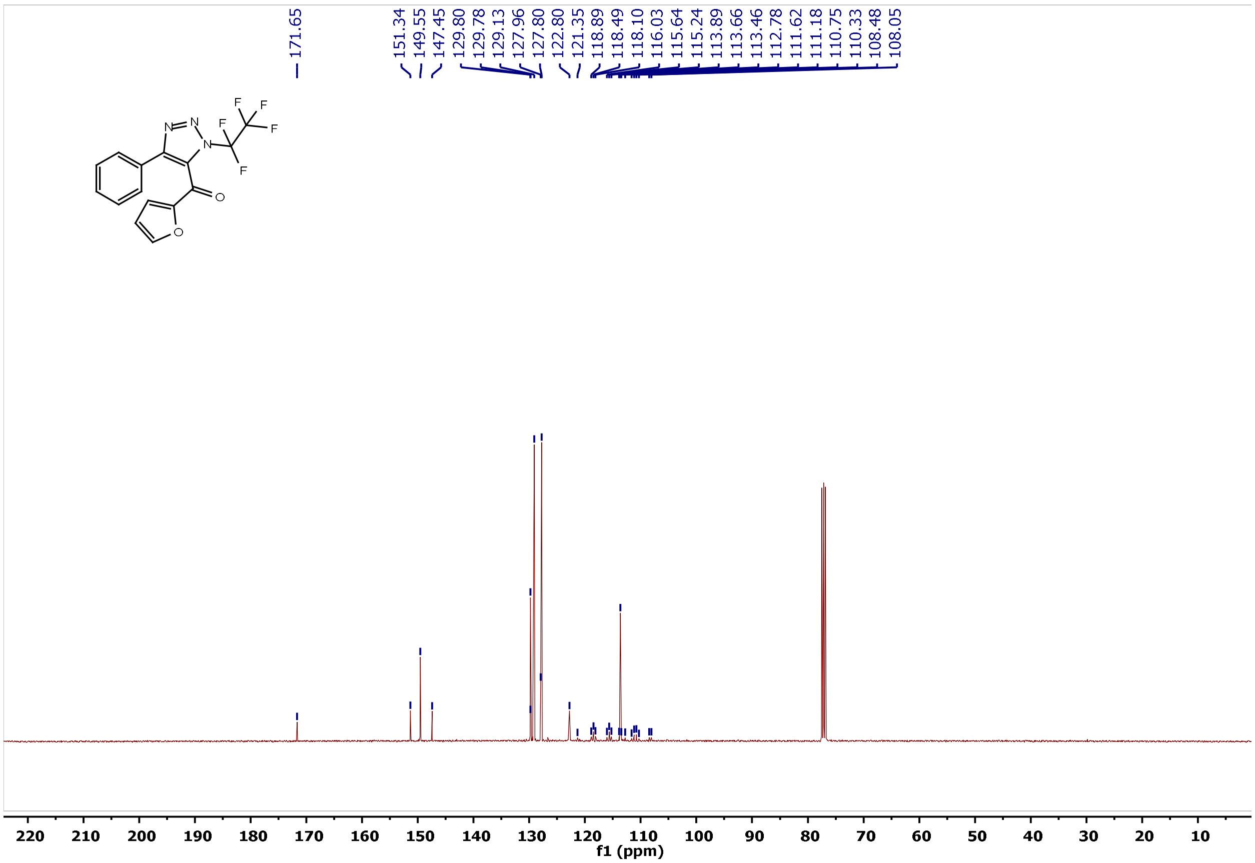

Figure S86. ^{19}F NMR spectrum of **3ac** ($CDCl_3$, 377 MHz)

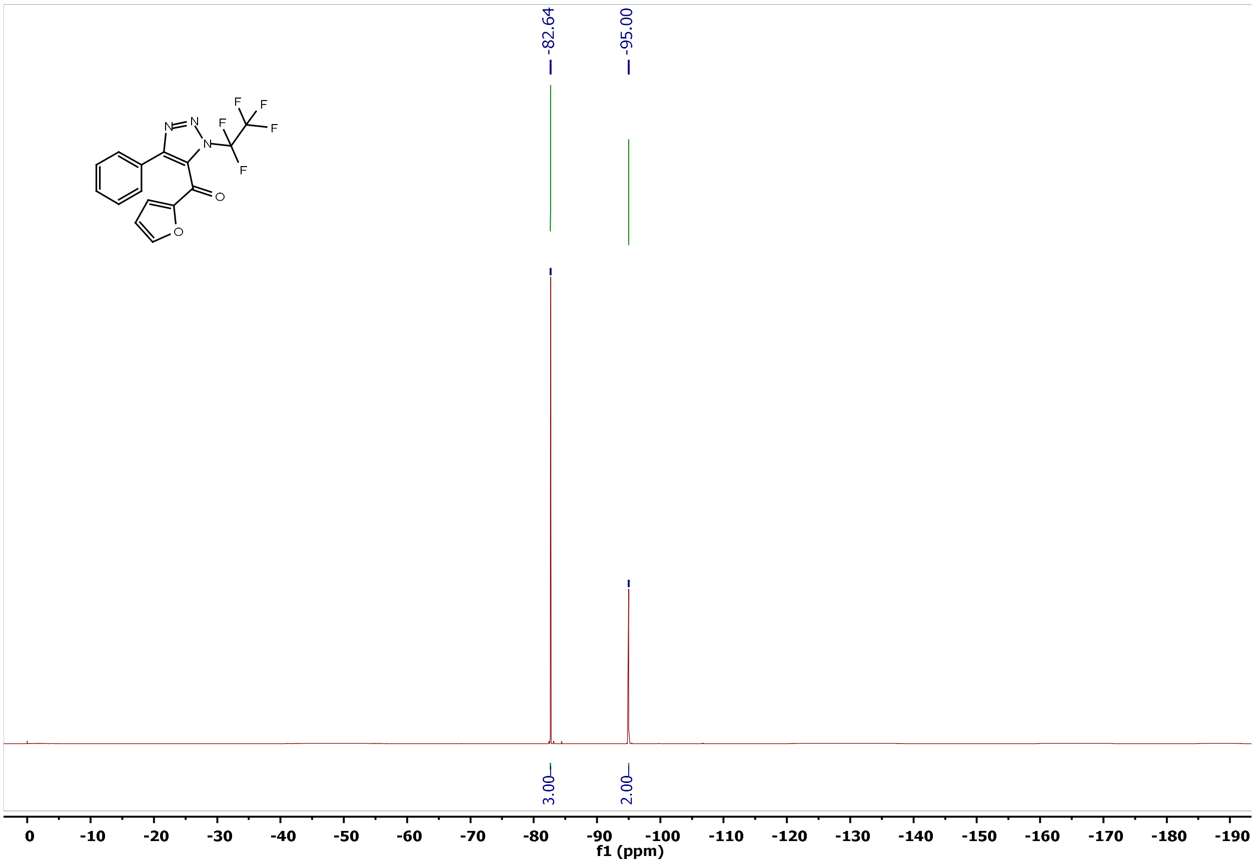

Figure S87. ^{1}H NMR spectrum of **3ad** ($CDCl_3$, 401 MHz)

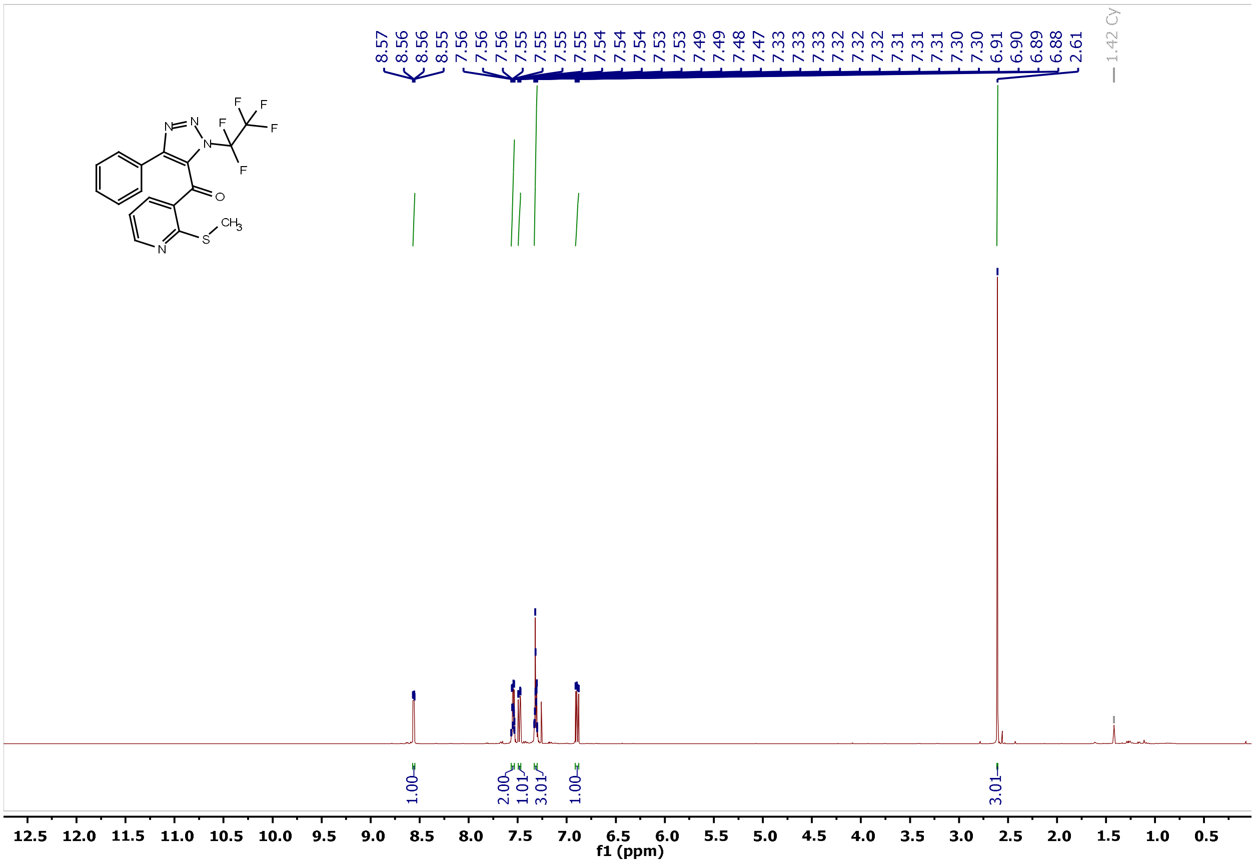

Figure S88. ^{13}C NMR spectrum of **3ad** ($CDCl_3$, 101 MHz)

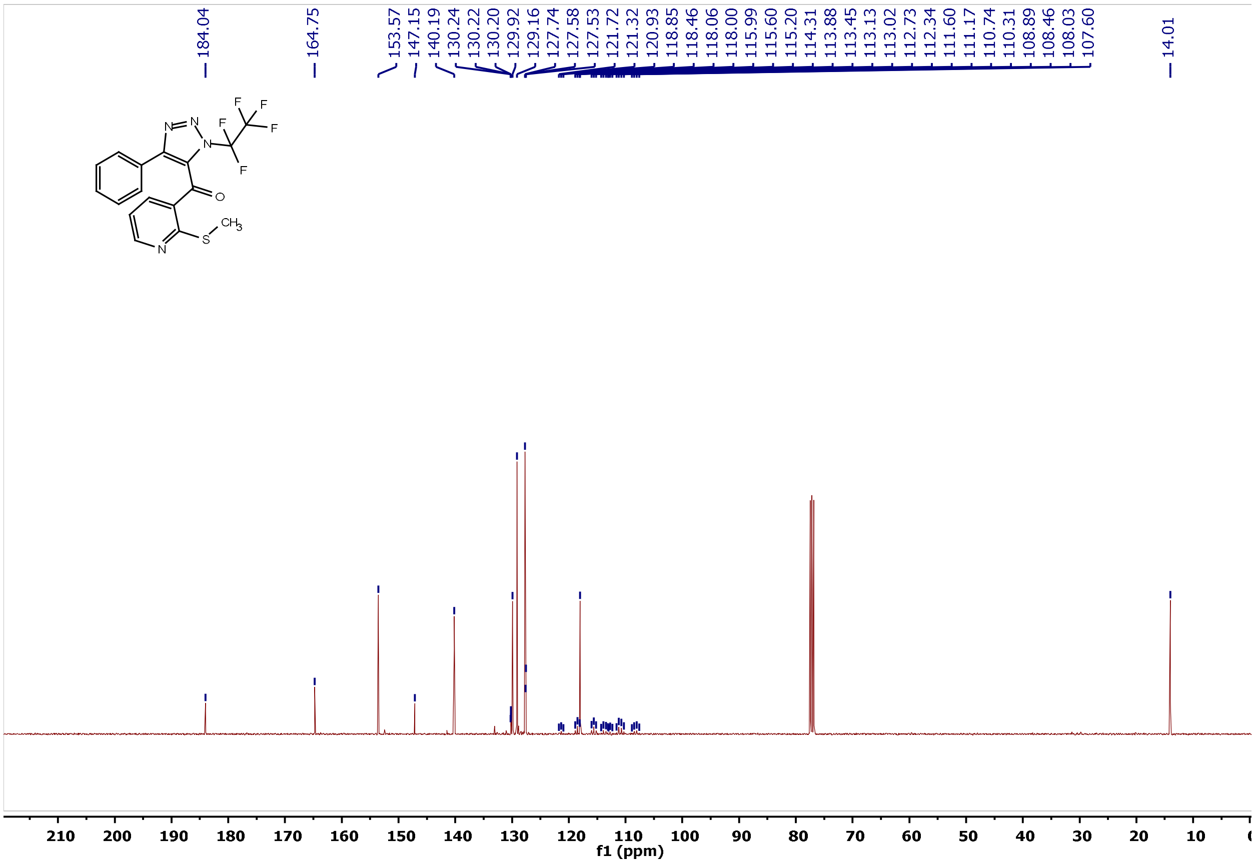

Figure S89. ^{19}F NMR spectrum of **3ad** ($CDCl_3$, 377 MHz)

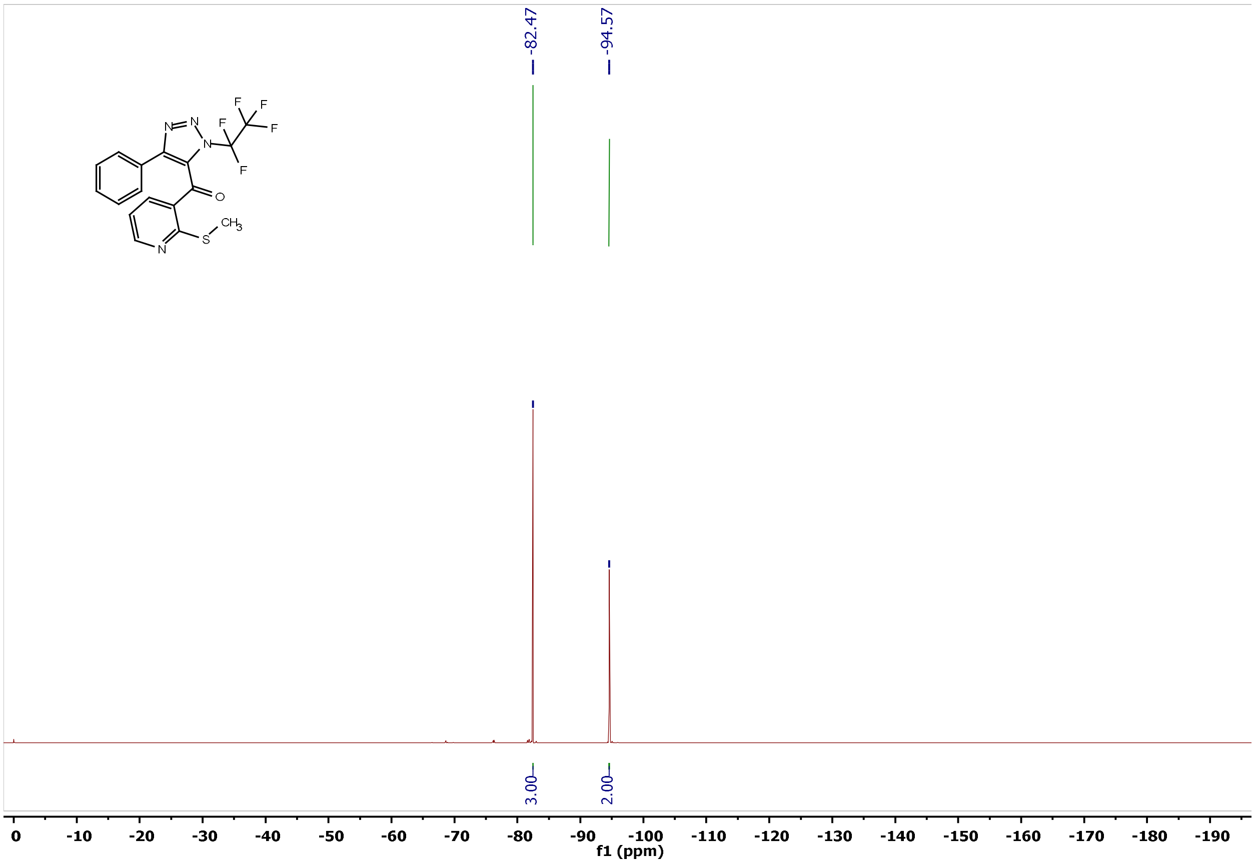

Figure S90. ^{1}H NMR spectrum of **4a** ($CDCl_3$, 401 MHz)

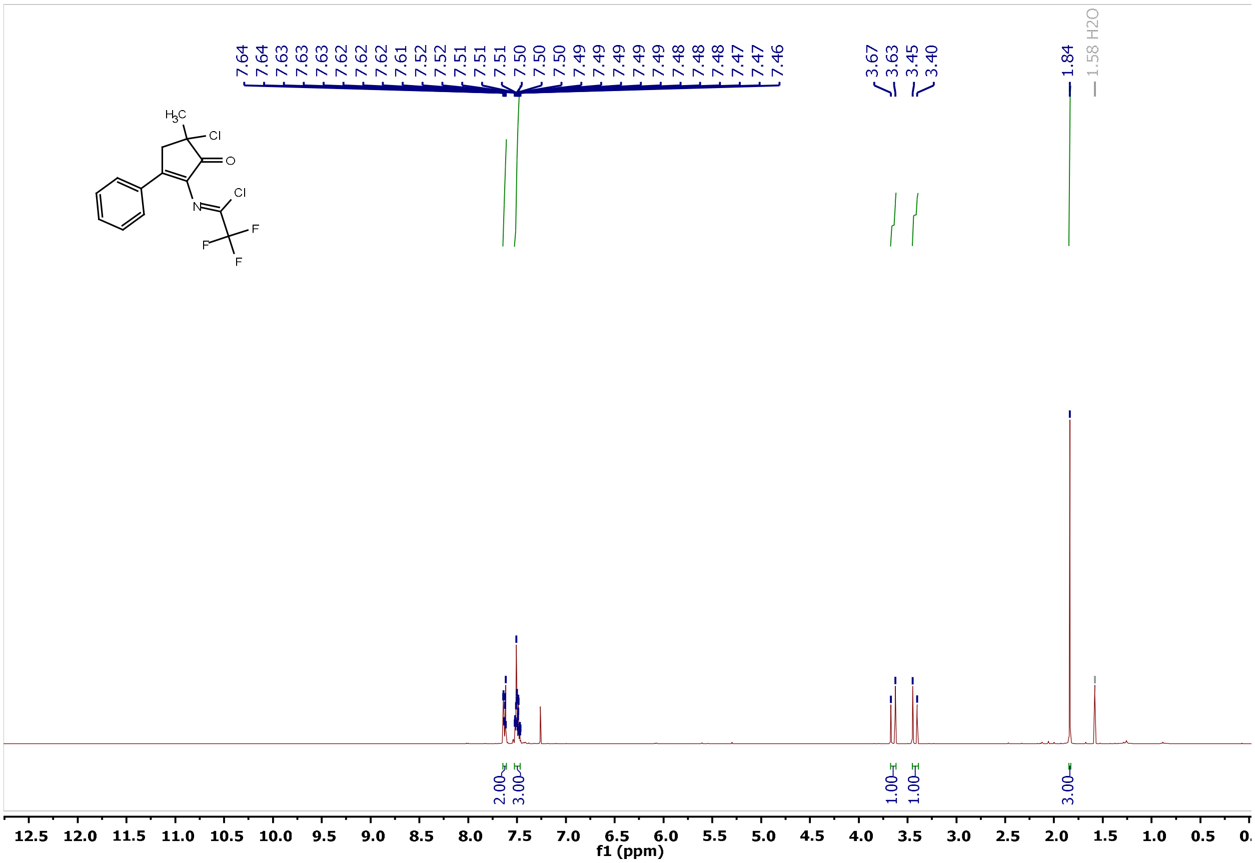

Figure S91. ^{13}C NMR spectrum of **4a** ($CDCl_3$, 101 MHz)

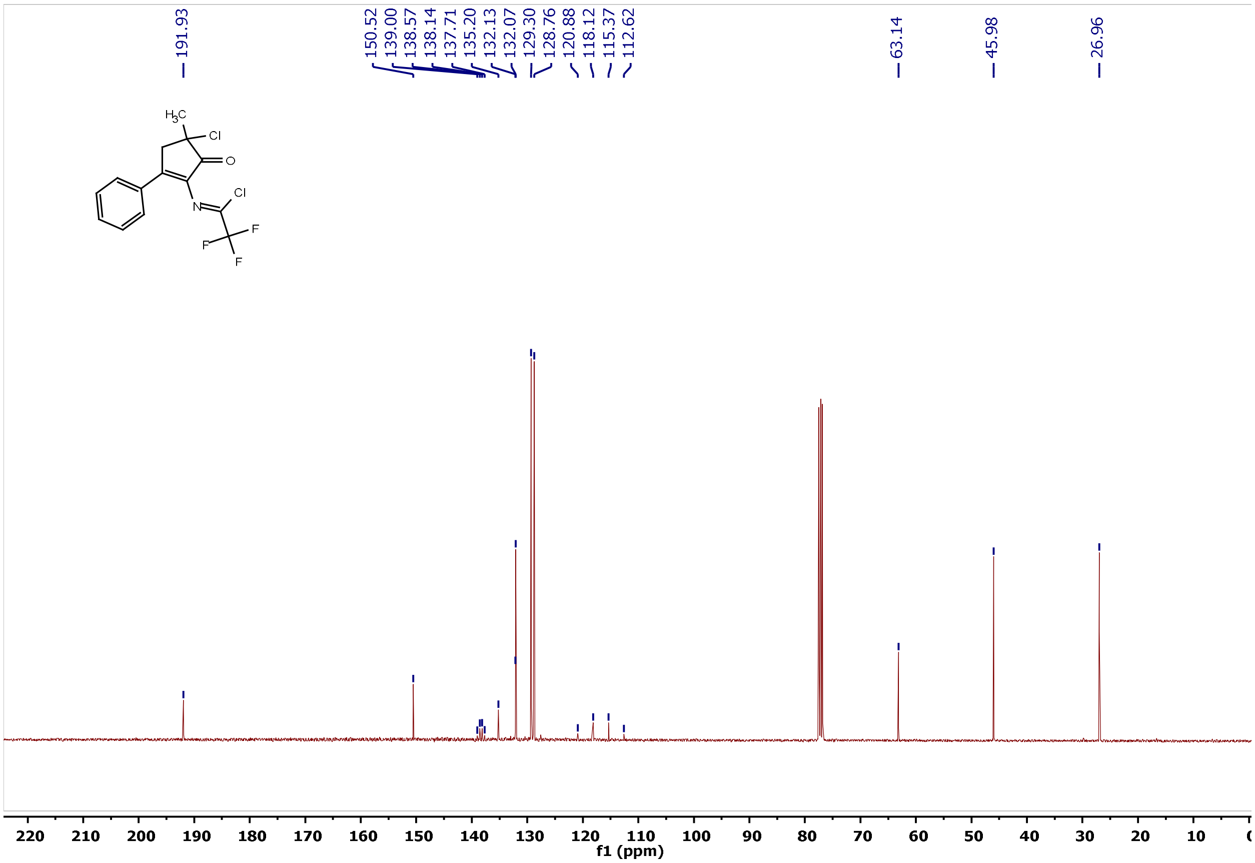

Figure S92. ^{19}F NMR spectrum of **4a** ($CDCl_3$, 377 MHz)

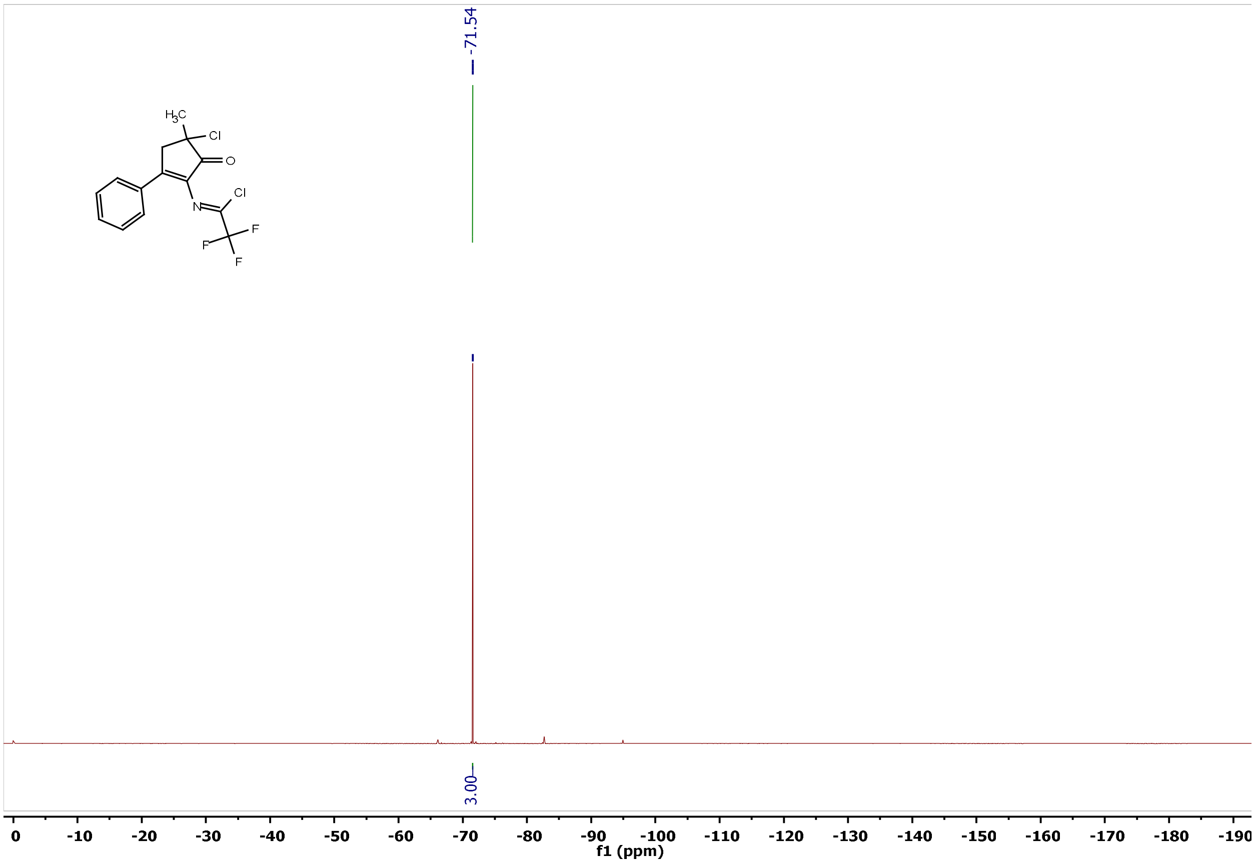

Figure S93. ^{1}H NMR spectrum of **4b** ($CDCl_3$, 401 MHz)

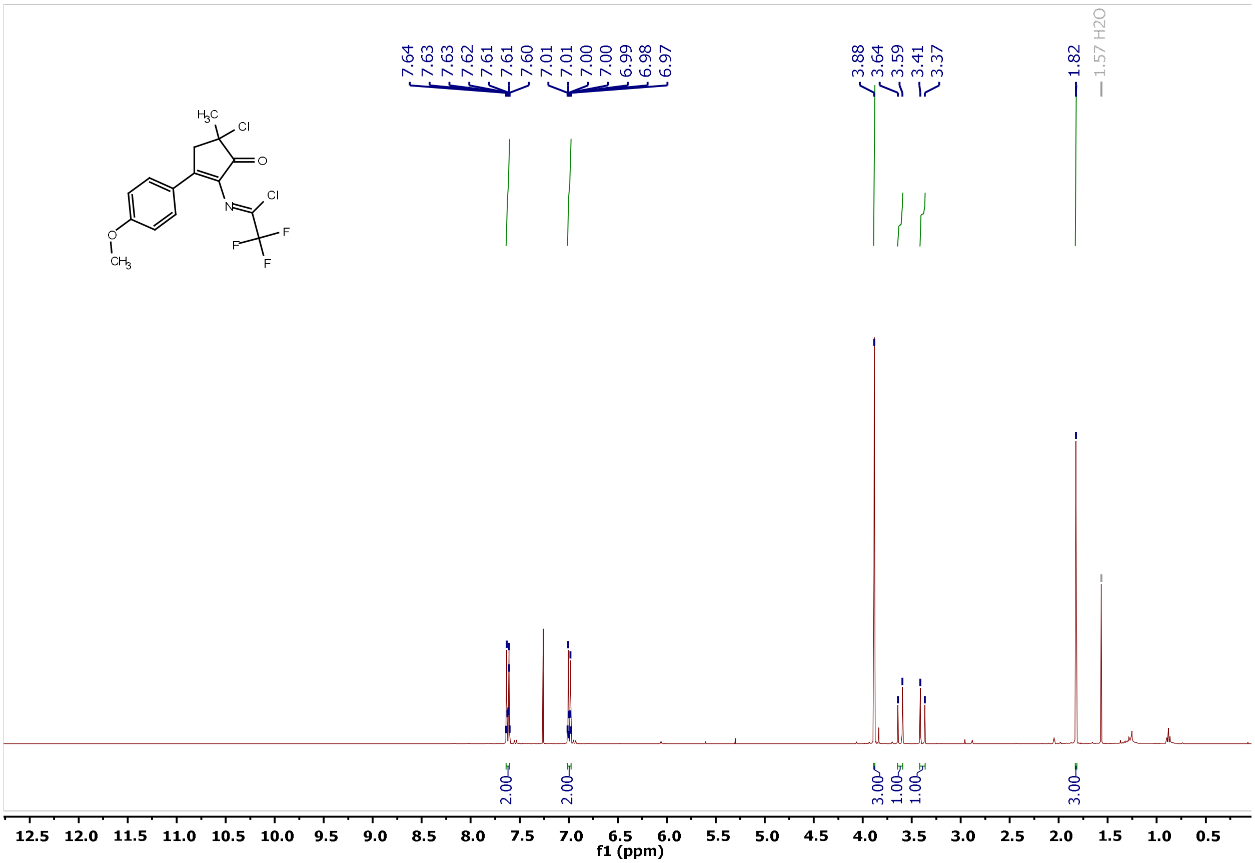

Figure S94. ^{13}C NMR spectrum of **4b** ($CDCl_3$, 151 MHz)

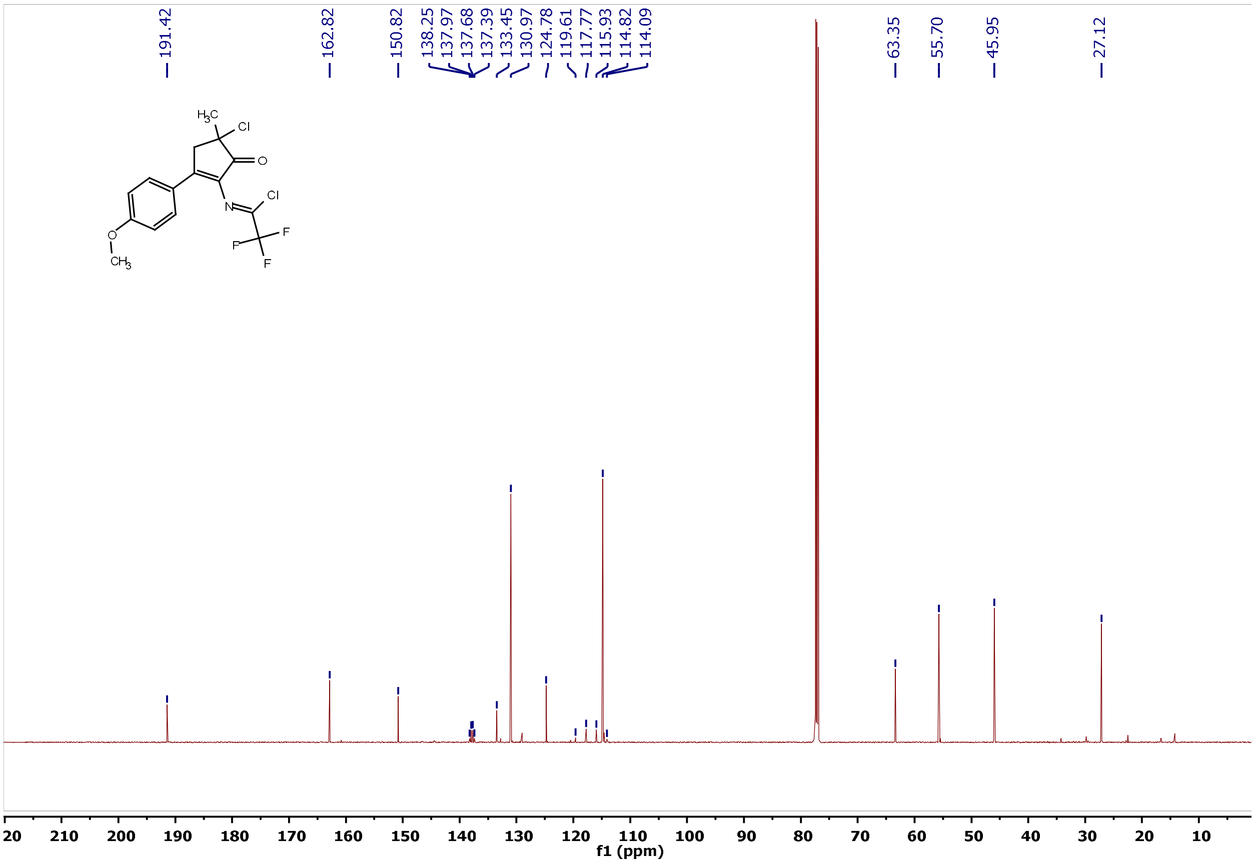

Figure S95. ^{19}F NMR spectrum of **4b** ($CDCl_3$, 377 MHz)

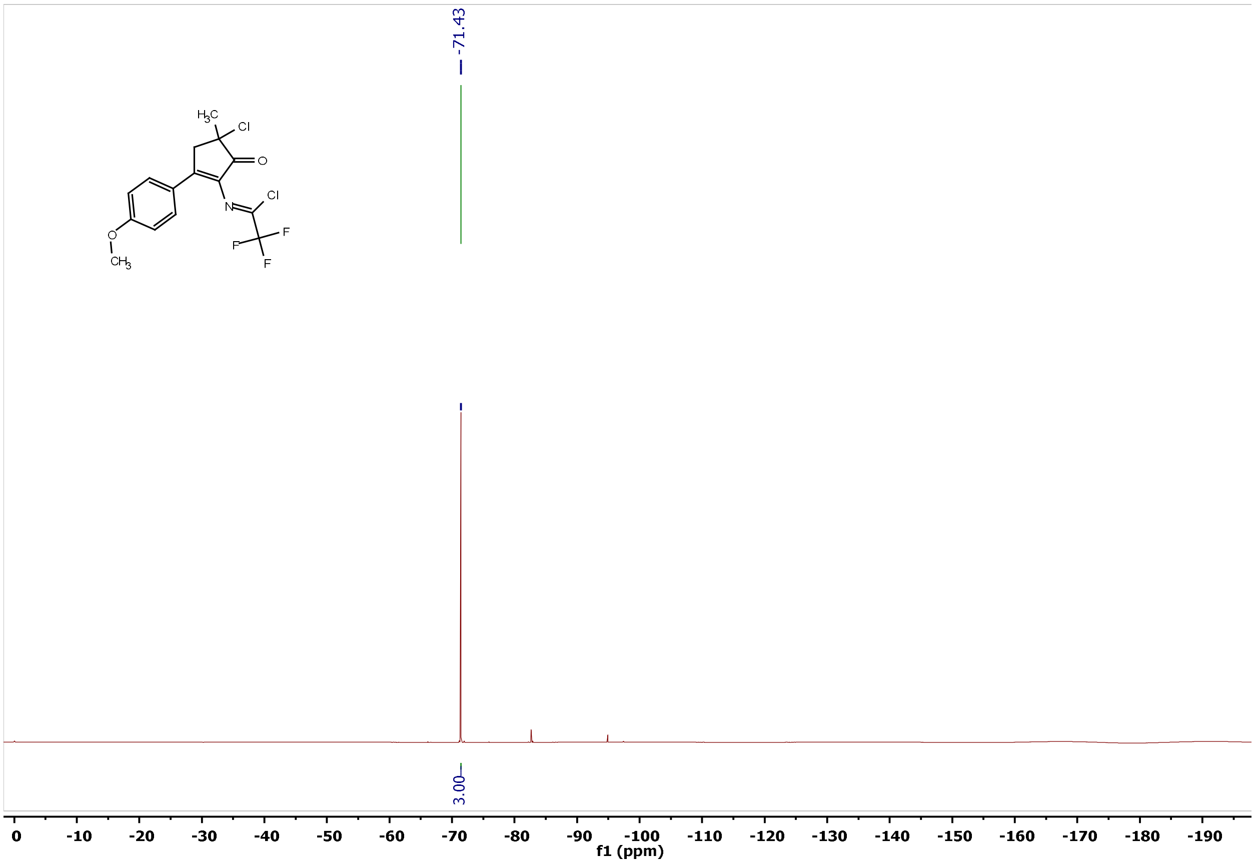

Figure S96. ^{1}H NMR spectrum of **4c** ($CDCl_3$, 401 MHz)

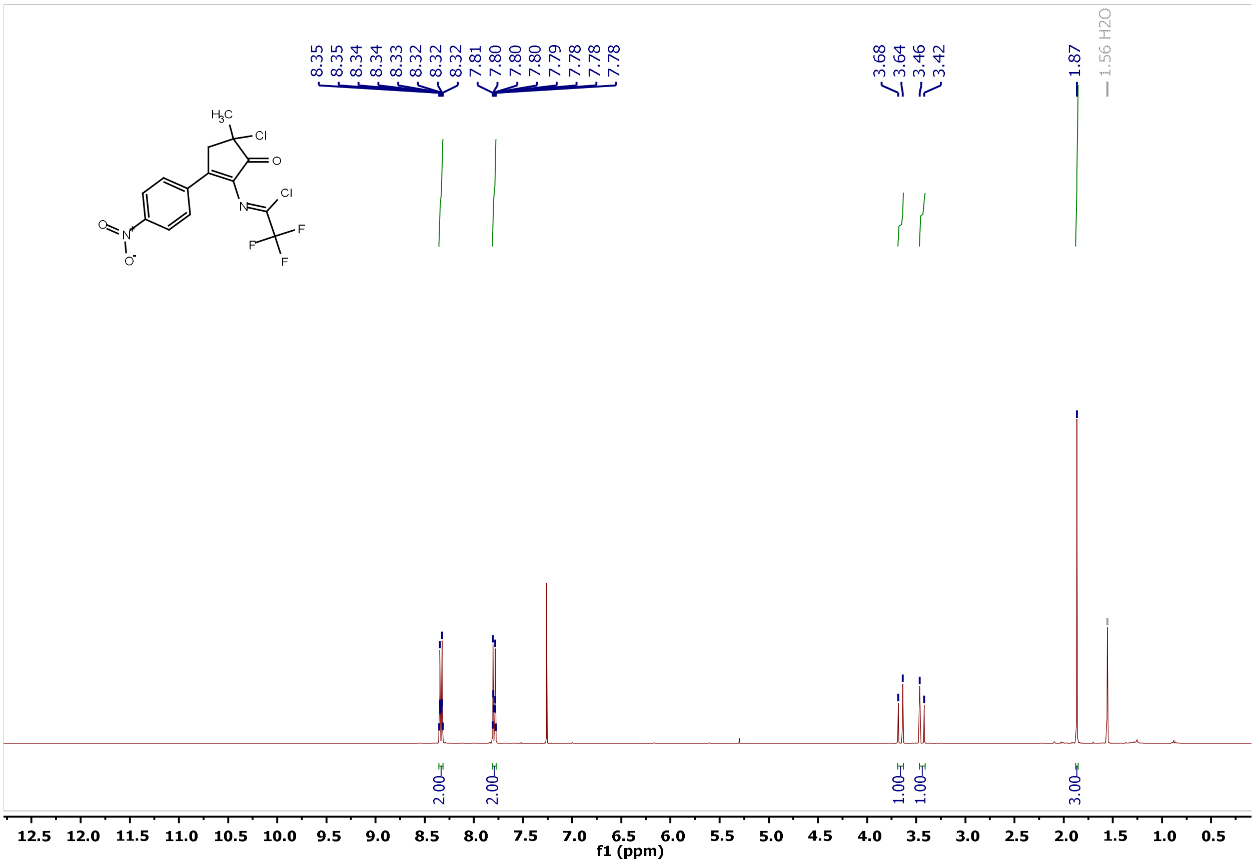

Figure S97. ^{13}C NMR spectrum of **4c** ($CDCl_3$, 151 MHz)

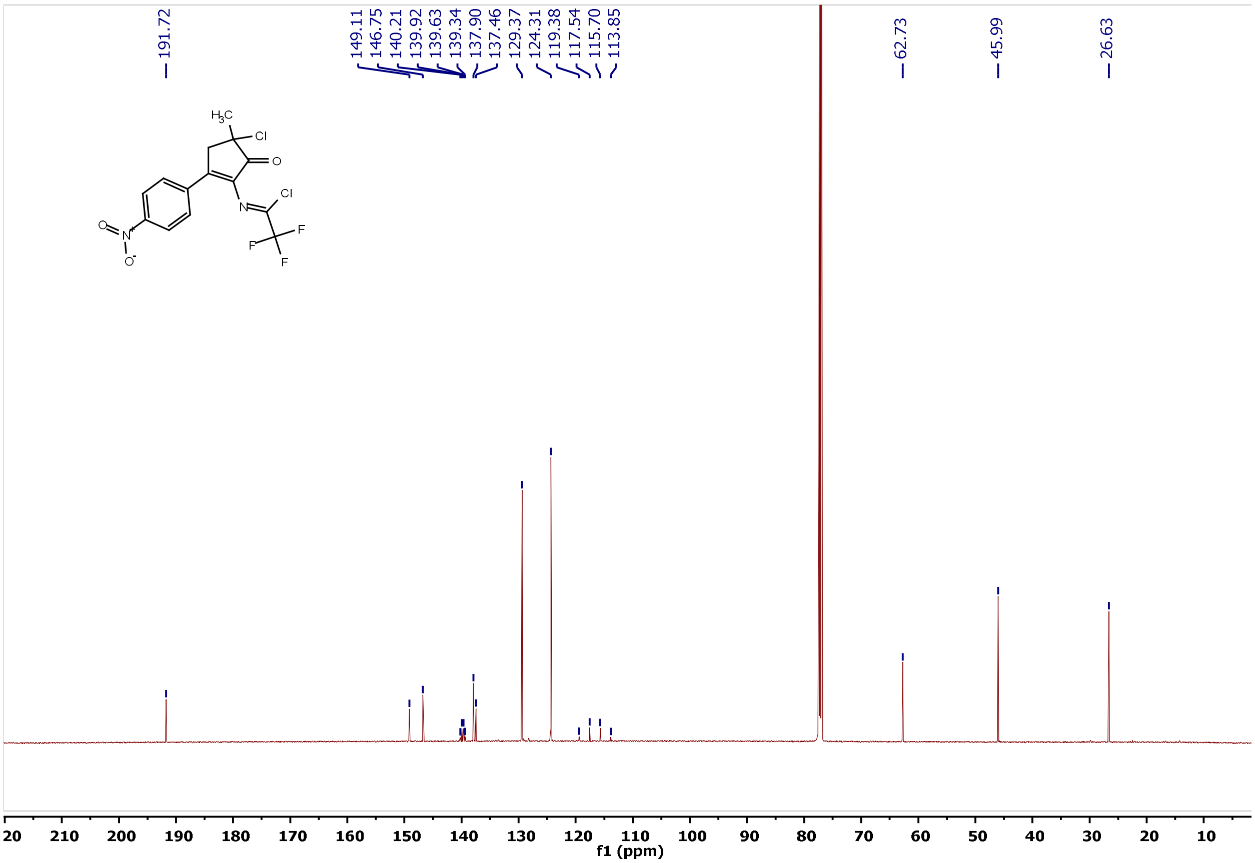

Figure S98. ^{19}F NMR spectrum of **4c** ($CDCl_3$, 377 MHz)

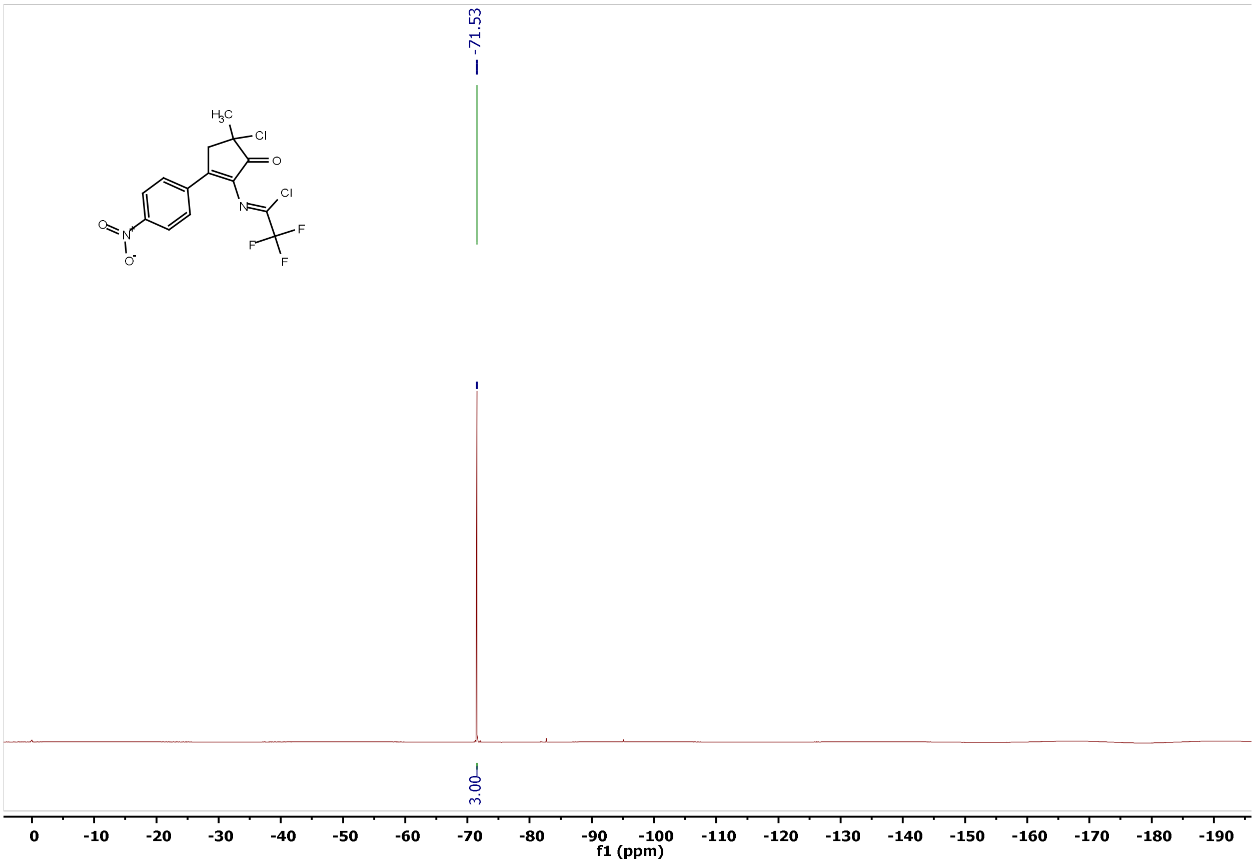

Figure S99. ^{1}H NMR spectrum of **4d** ($CDCl_3$, 401 MHz)

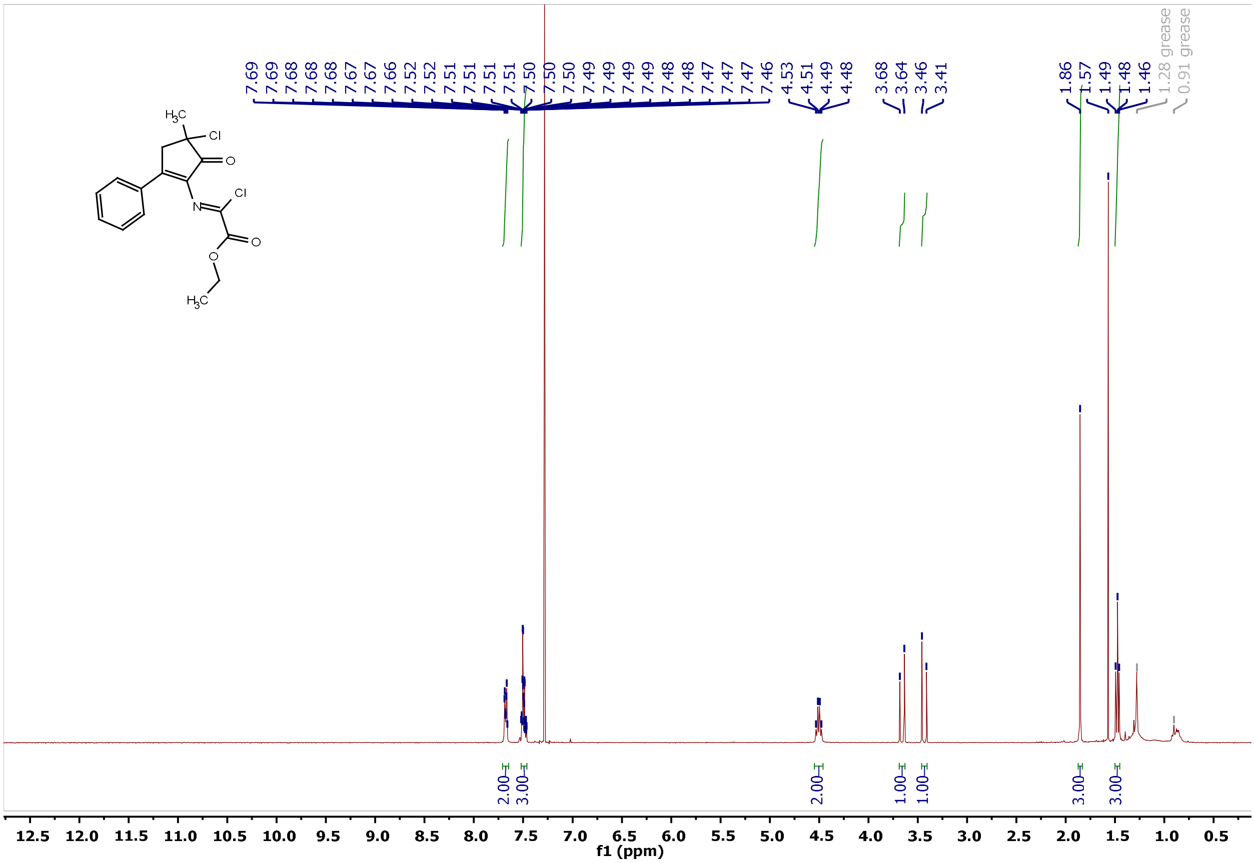

Figure S100. ^{13}C NMR spectrum of **4d** ($CDCl_3$, 126 MHz)

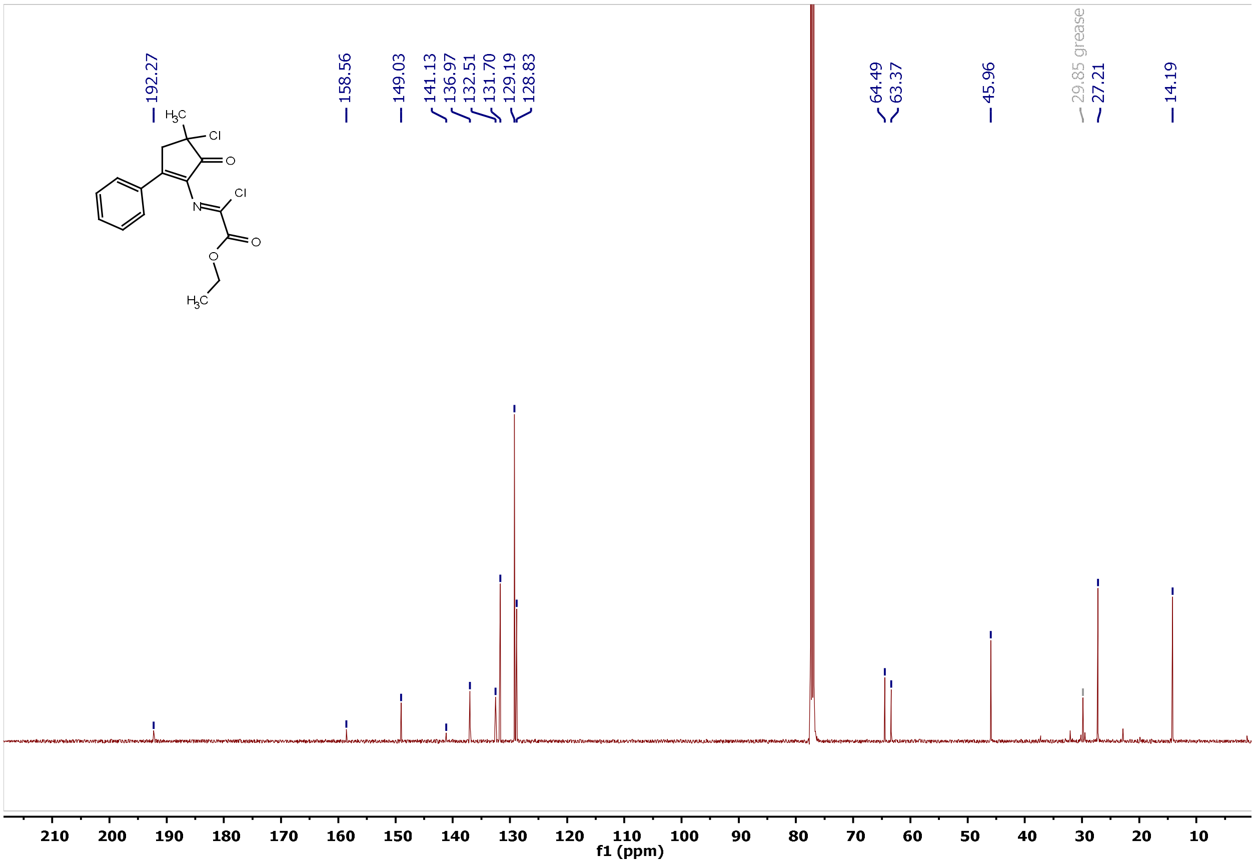

Figure S101. ^{1}H NMR spectrum of **4e** ($CDCl_3$, 401 MHz)

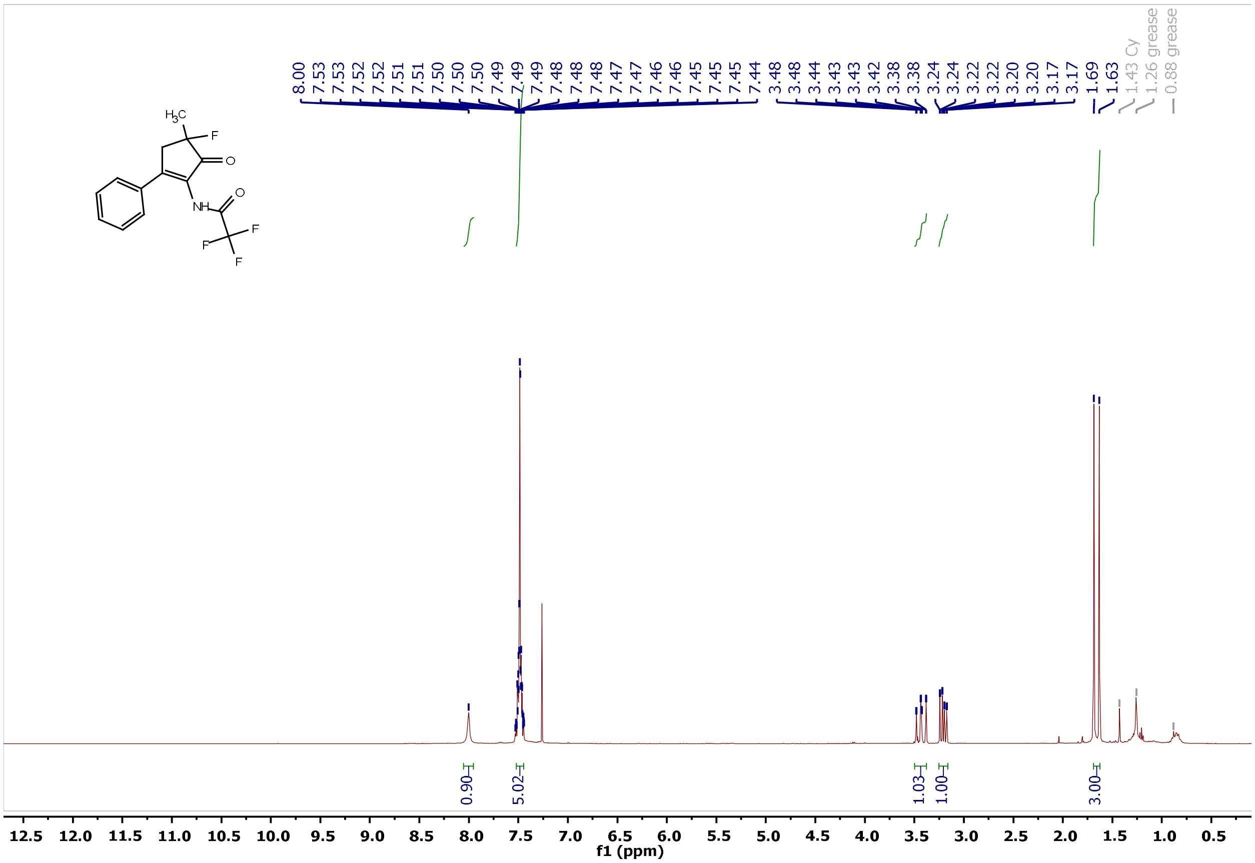

Figure S102. ^{13}C NMR spectrum of **4e** ($CDCl_3$, 101 MHz)

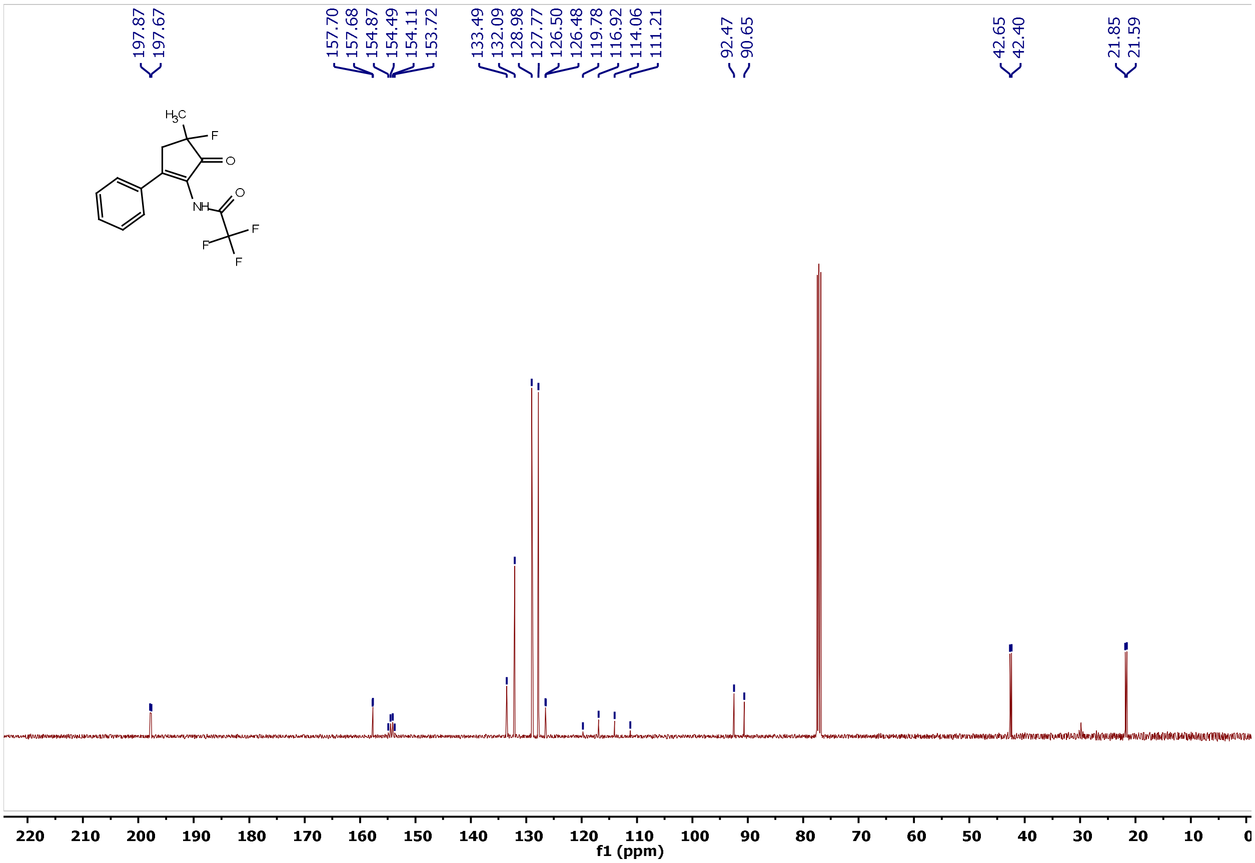

Figure S103. ^{19}F NMR spectrum of **4e** ($CDCl_3$, 377 MHz)

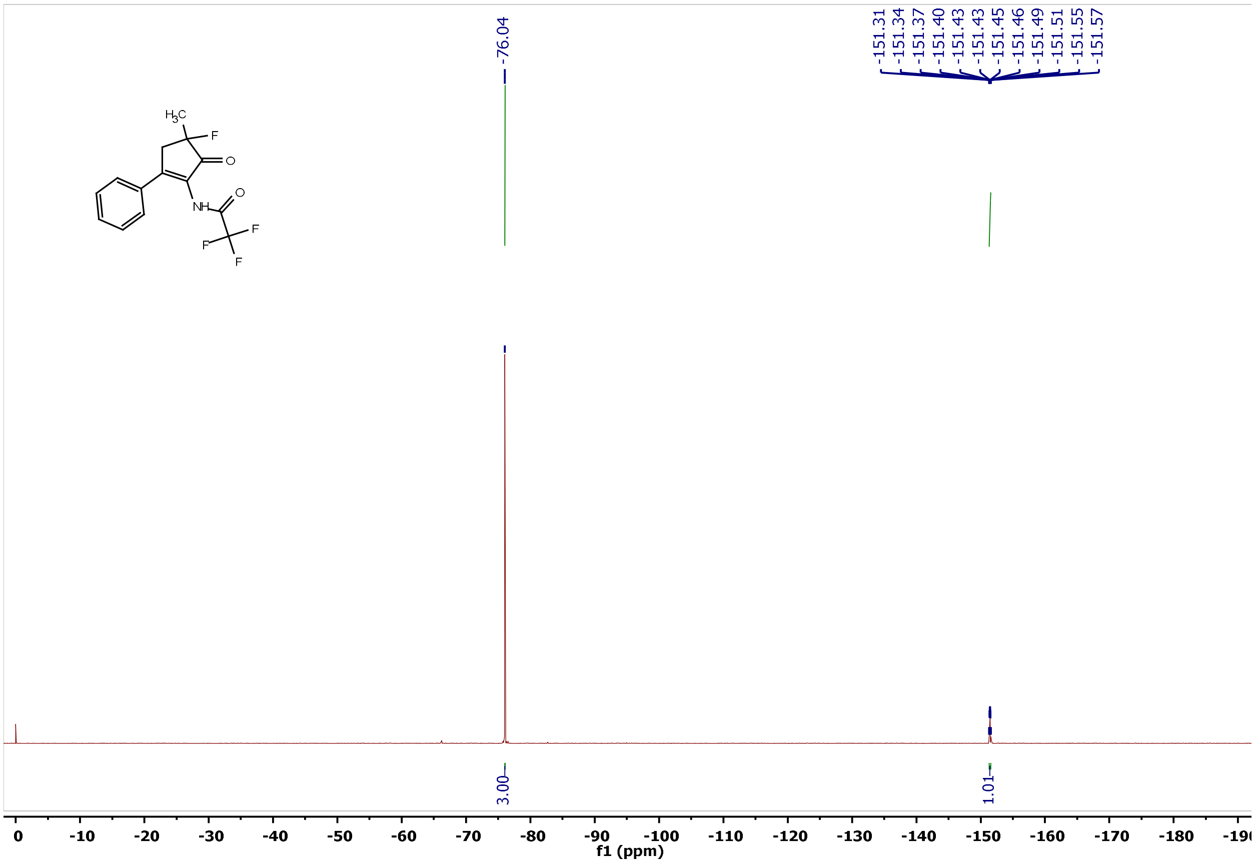

Figure S104. ^{1}H NMR spectrum of **4f** ($CDCl_3$, 401 MHz)

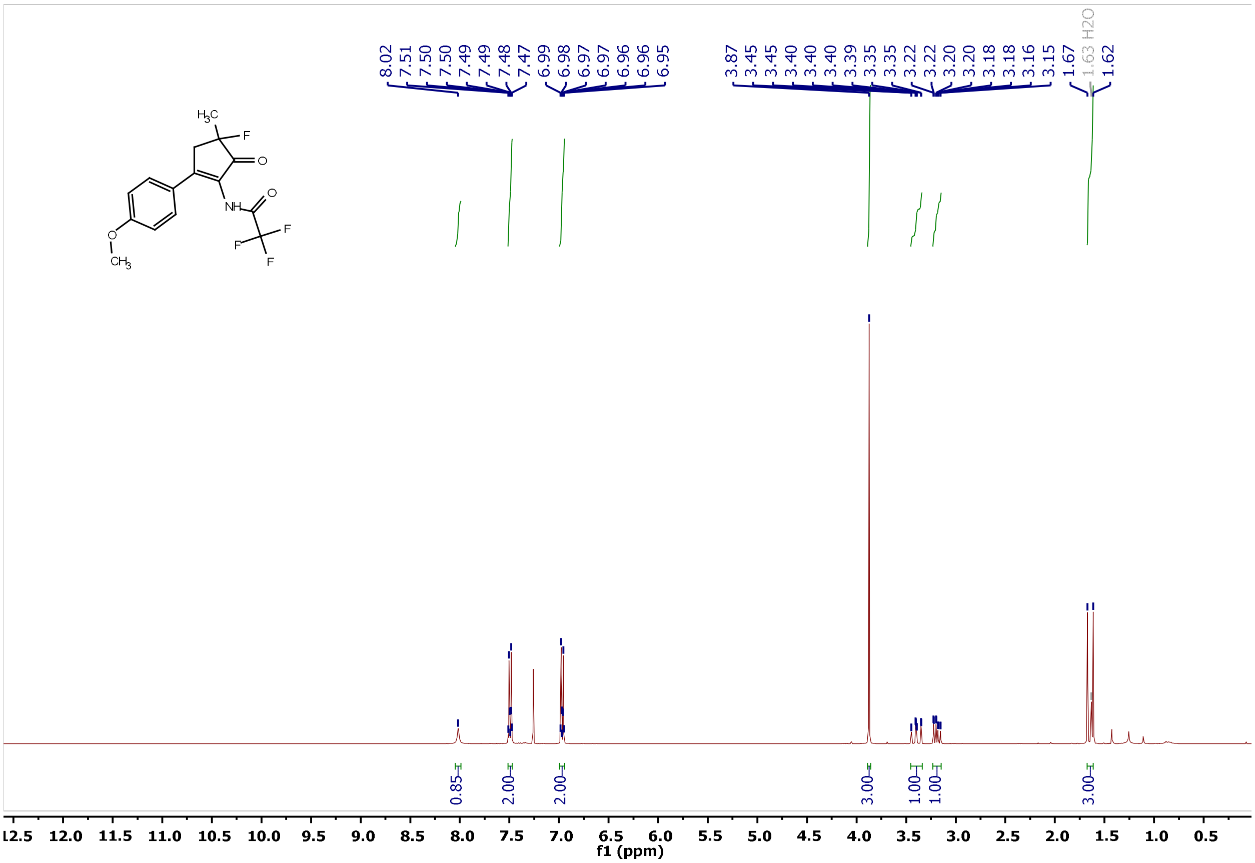

Figure S105. ^{13}C NMR spectrum of **4f** ($CDCl_3$, 101 MHz)

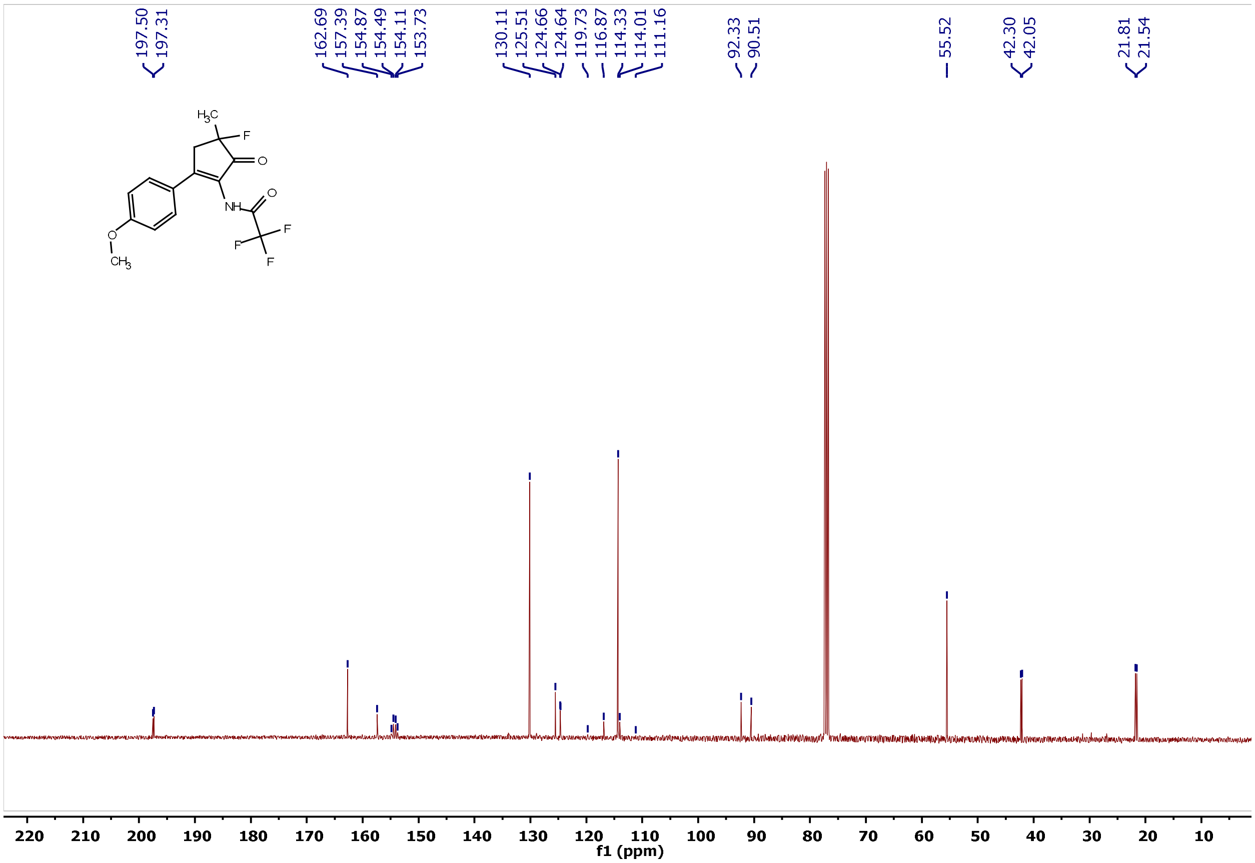

Figure S106. ^{19}F NMR spectrum of **4f** ($CDCl_3$, 377 MHz)

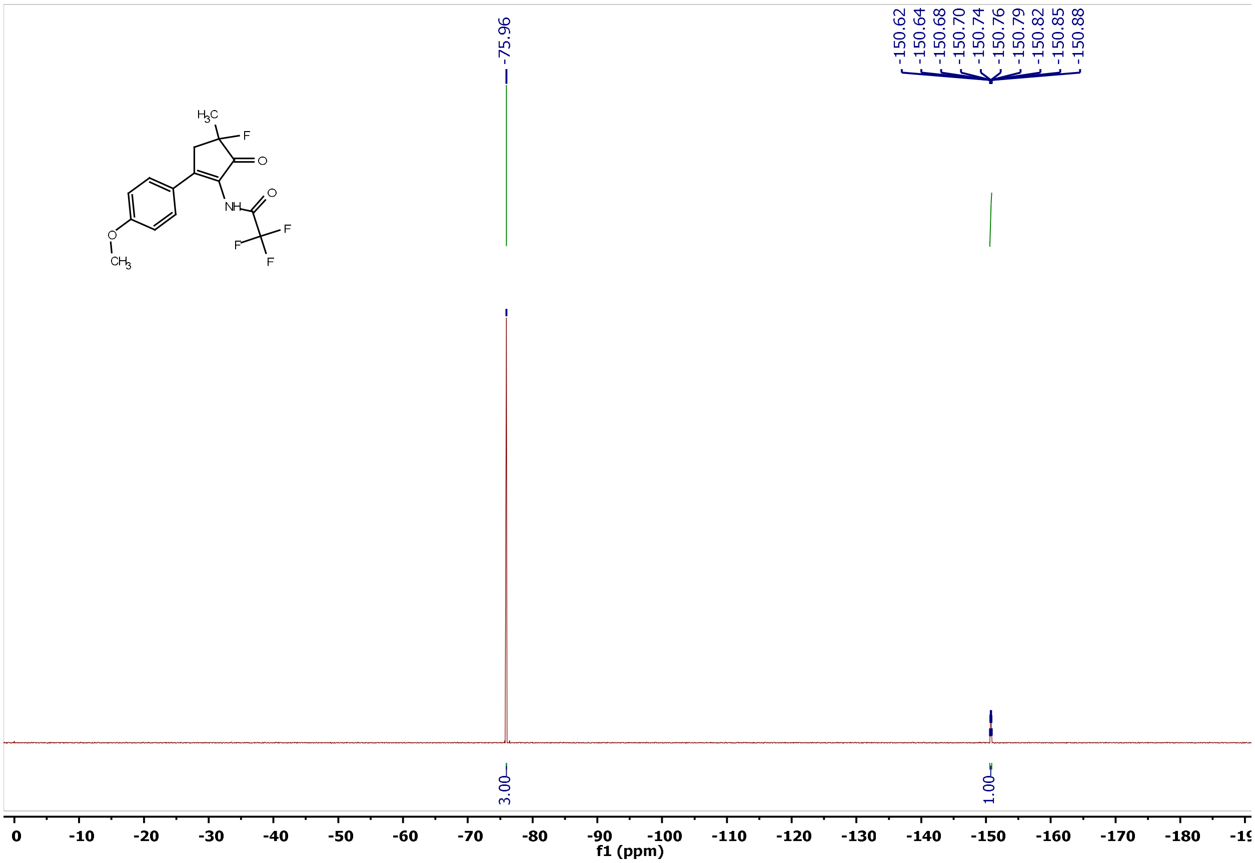

Figure S107. ^{1}H NMR spectrum of **4g** ($CDCl_3$, 401 MHz)

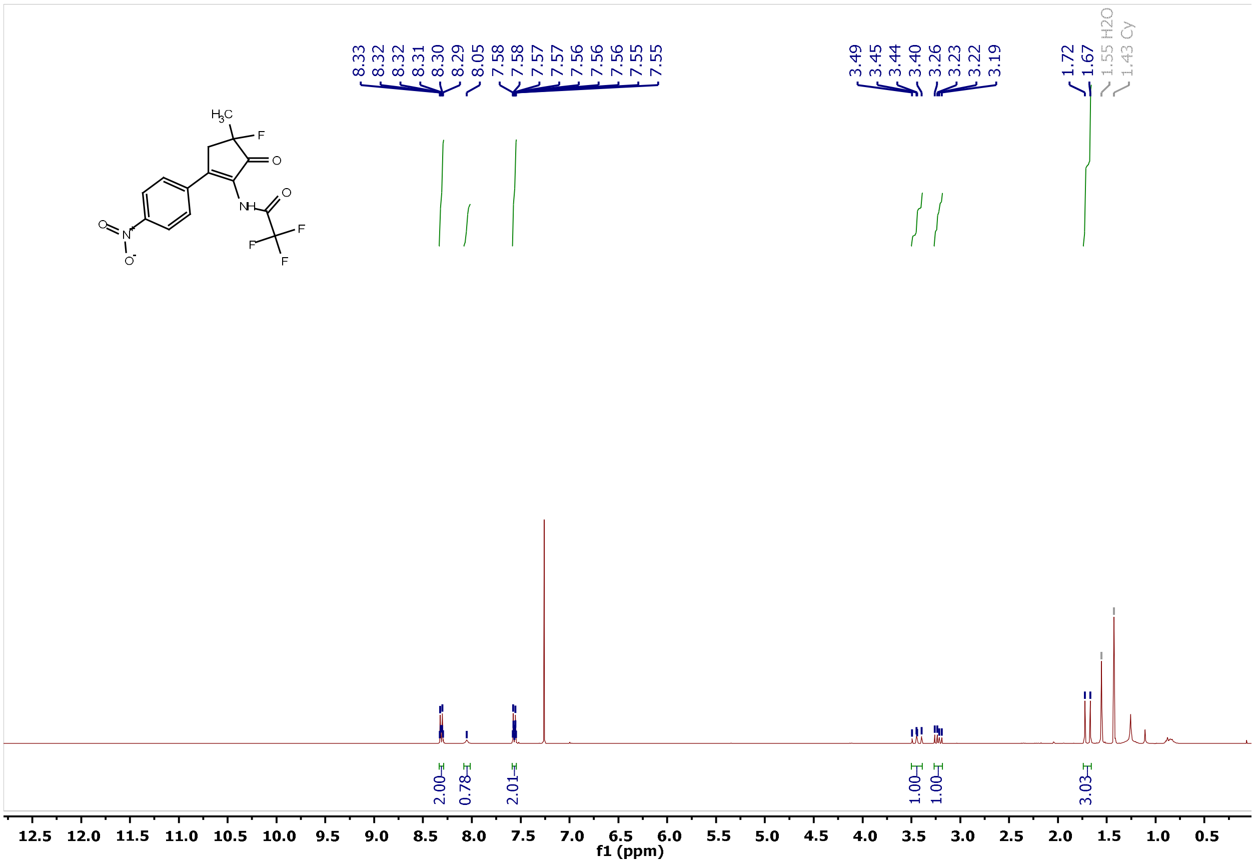

Figure S108. ^{13}C NMR spectrum of **4g** ($CDCl_3$, 151 MHz)

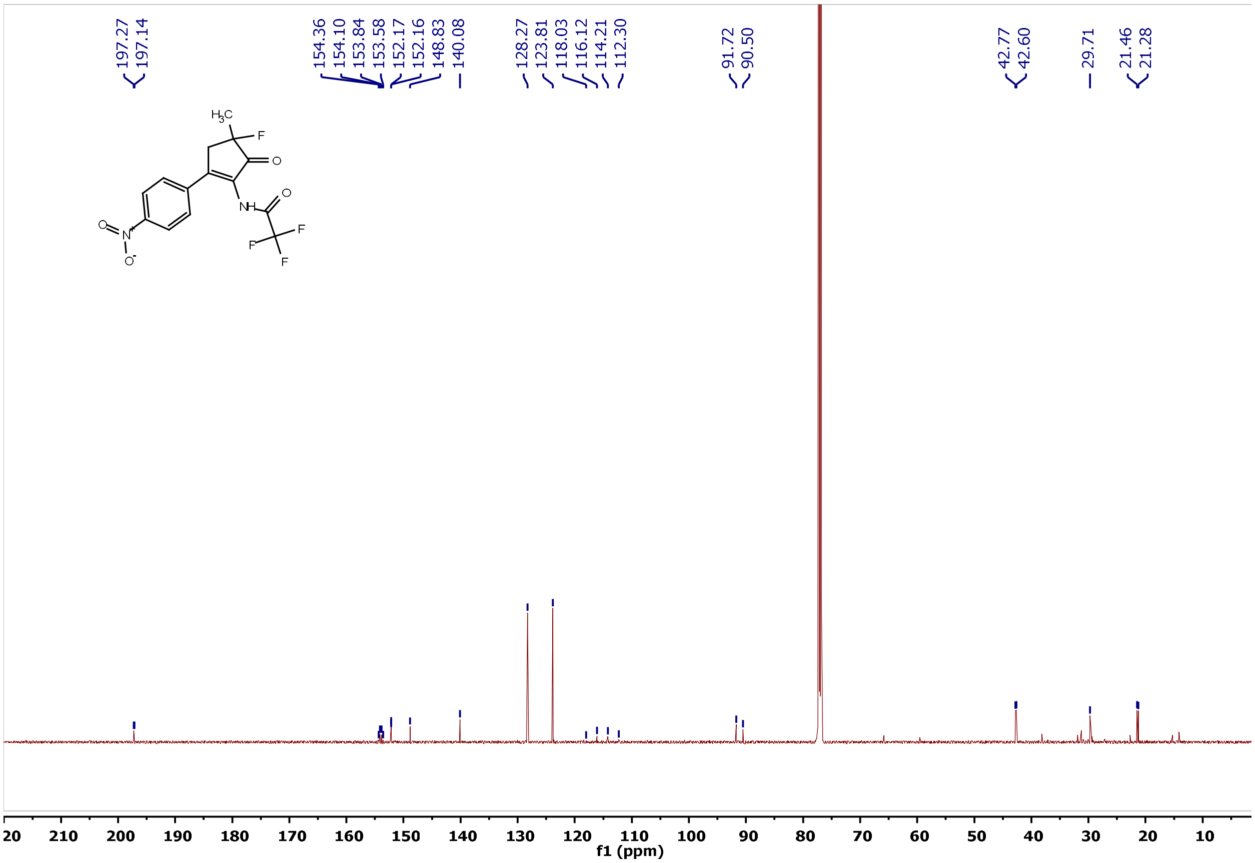

Figure S109. ^{19}F NMR spectrum of **4g** ($CDCl_3$, 377 MHz)

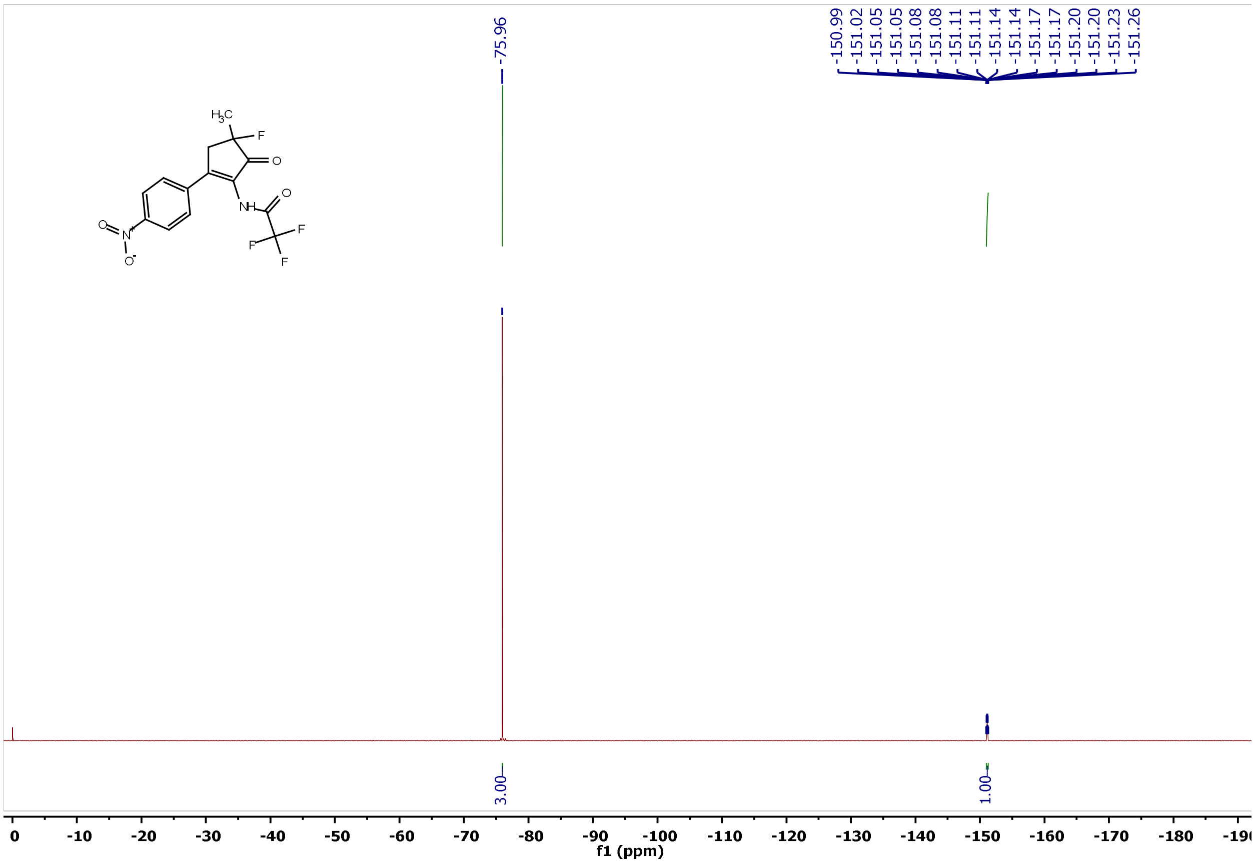

Figure S110. ^{1}H NMR spectrum of **4h** ($CDCl_3$, 401 MHz)

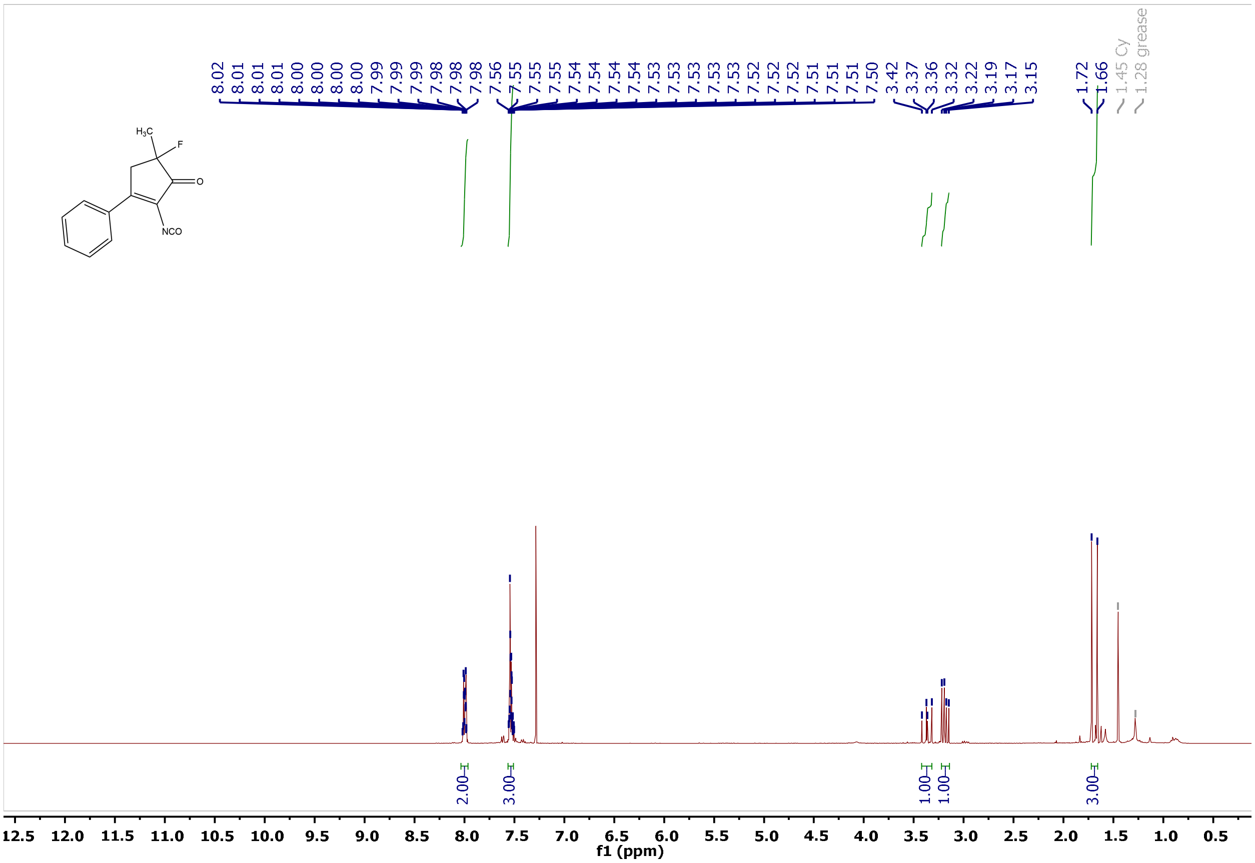

Figure S111. ^{13}C NMR spectrum of **4h** ($CDCl_3$, 101 MHz)

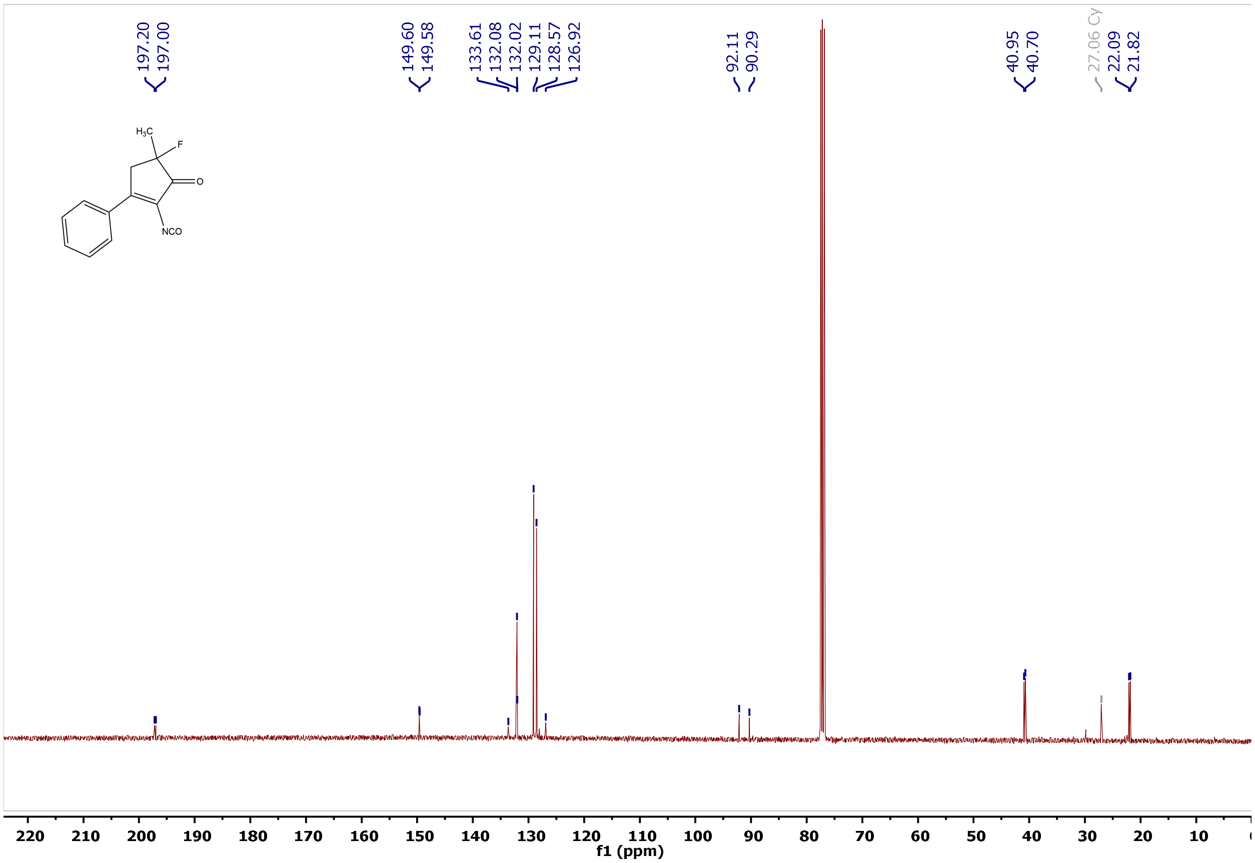

Figure S112. ^{19}F NMR spectrum of **4h** ($CDCl_3$, 377 MHz)

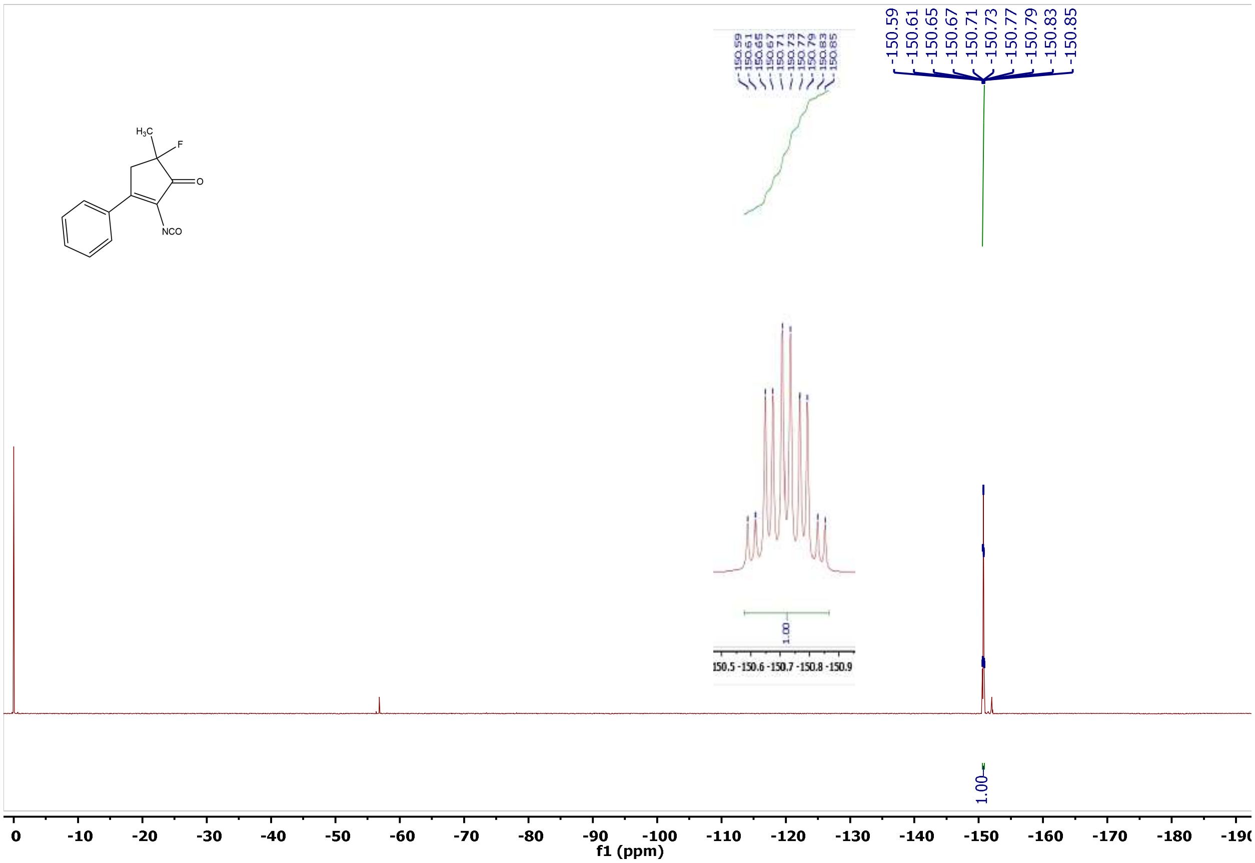

Figure S113. ^{1}H NMR spectrum of **4i** ($CDCl_3$, 401 MHz)

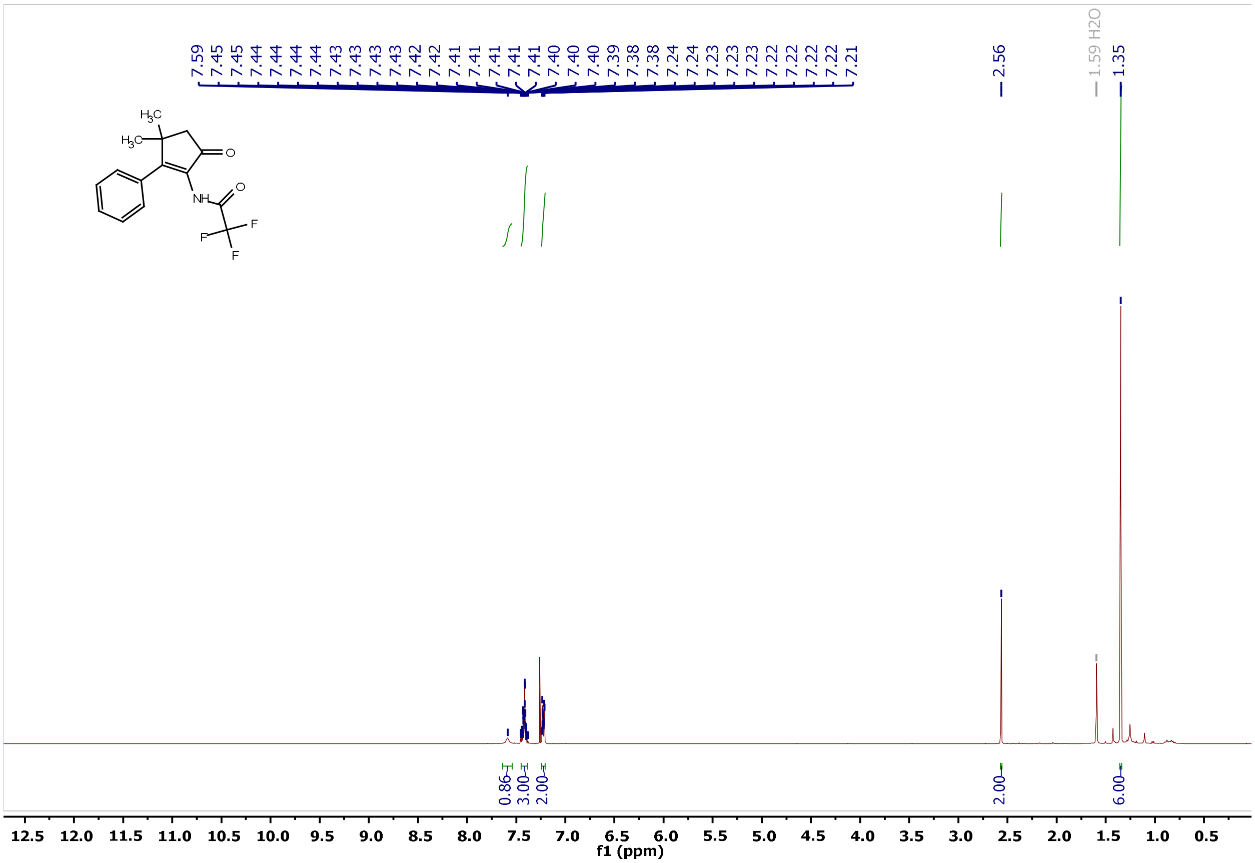

Figure S114. ^{13}C NMR spectrum of **4i** ($CDCl_3$, 101 MHz)

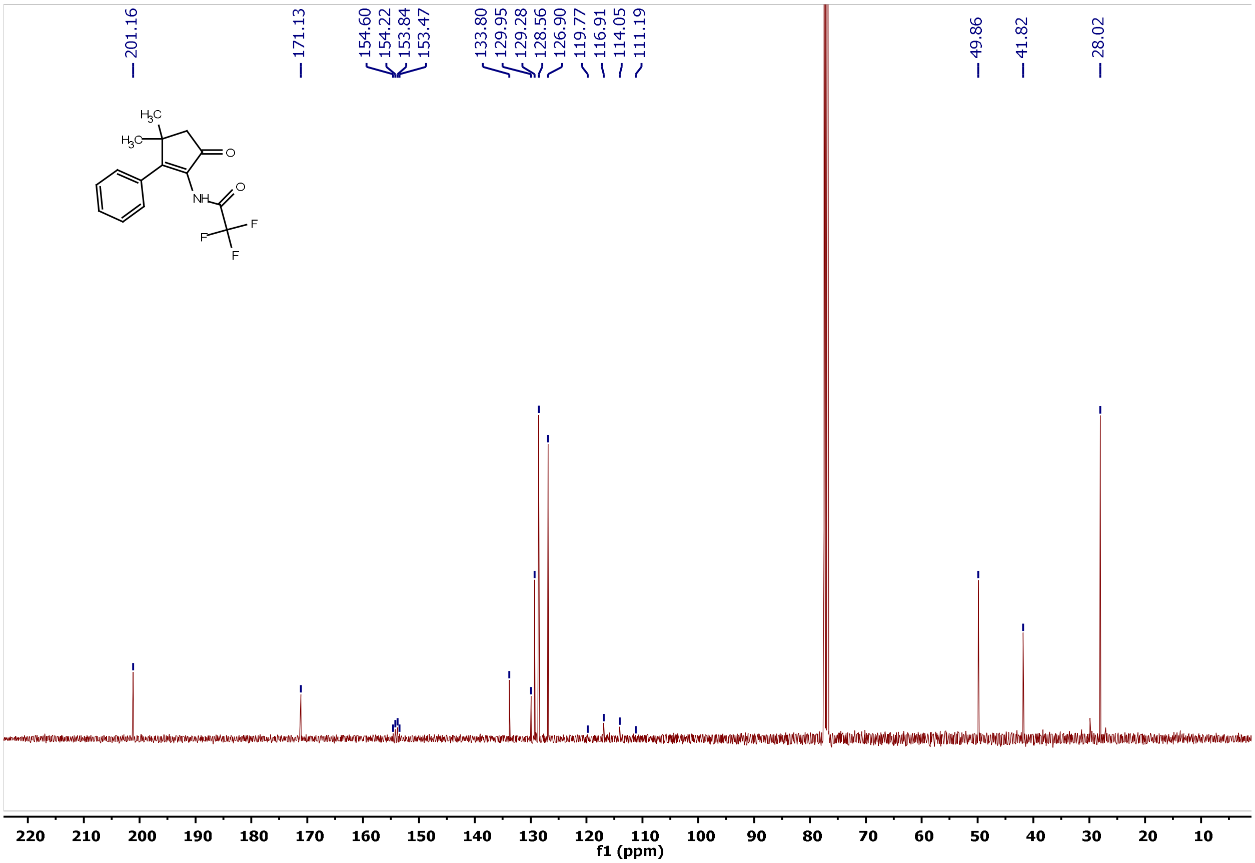

Figure S115. ^{19}F NMR spectrum of **4i** ($CDCl_3$, 377 MHz)

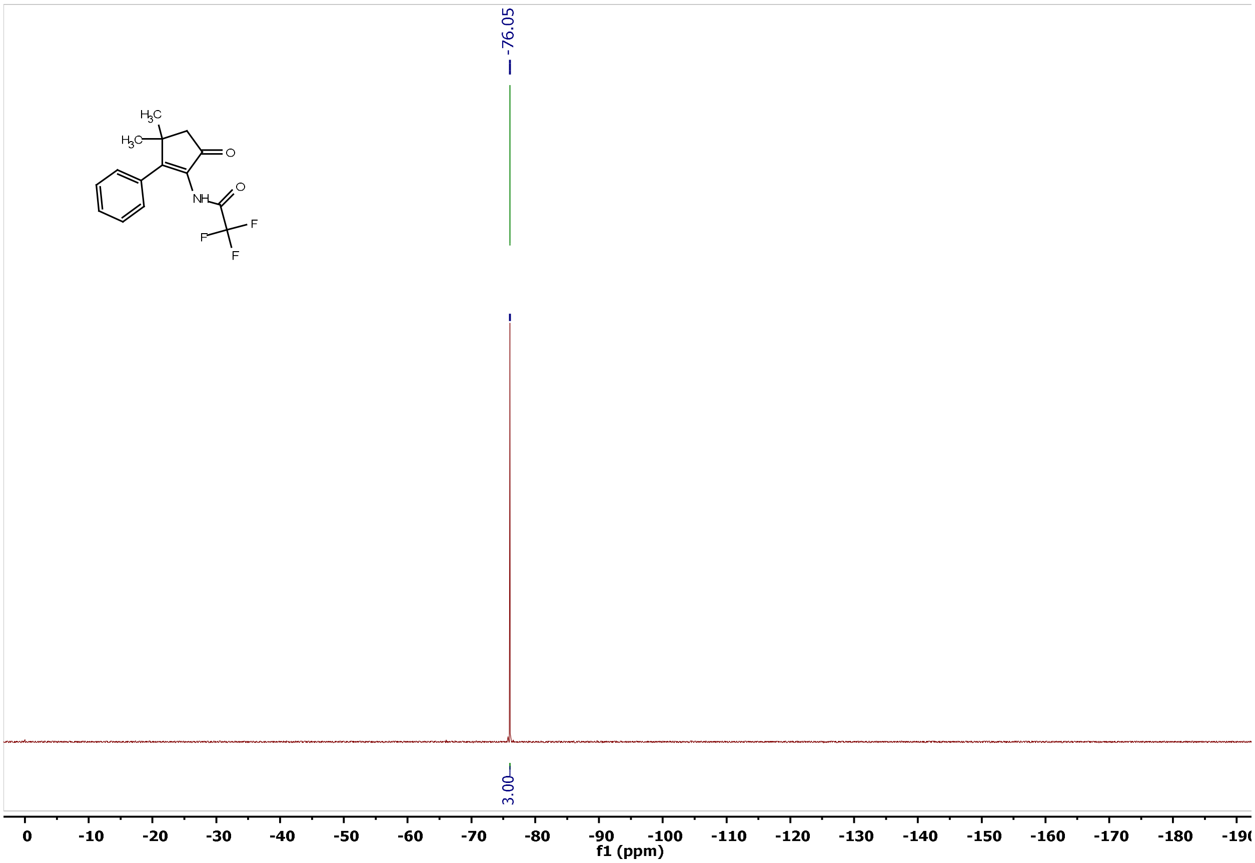

Figure S116. ^{1}H NMR spectrum of **5f** ($CDCl_3$, 401 MHz)

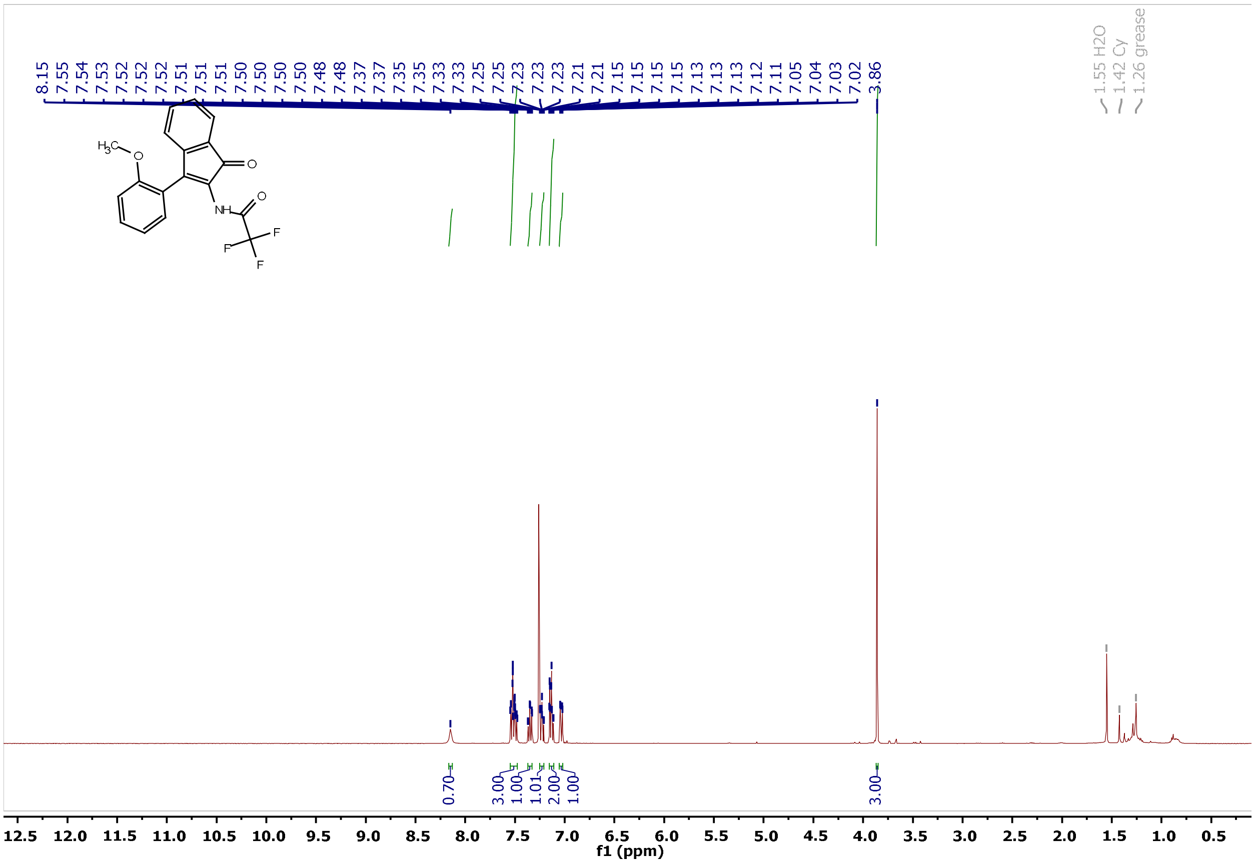

Figure S117. ^{13}C NMR spectrum of **5f** ($CDCl_3$, 126 MHz)

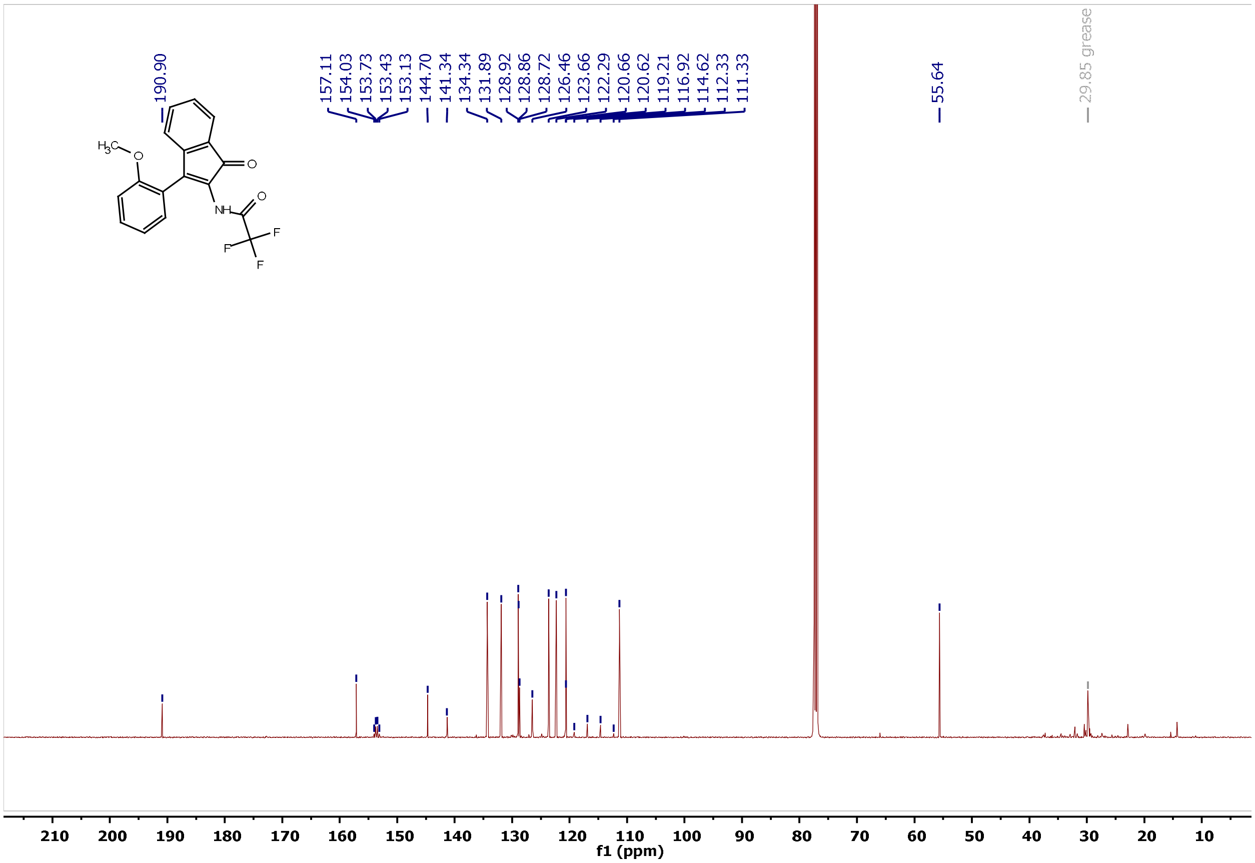

Figure S118. ^{19}F NMR spectrum of **5f** ($CDCl_3$, 377 MHz)

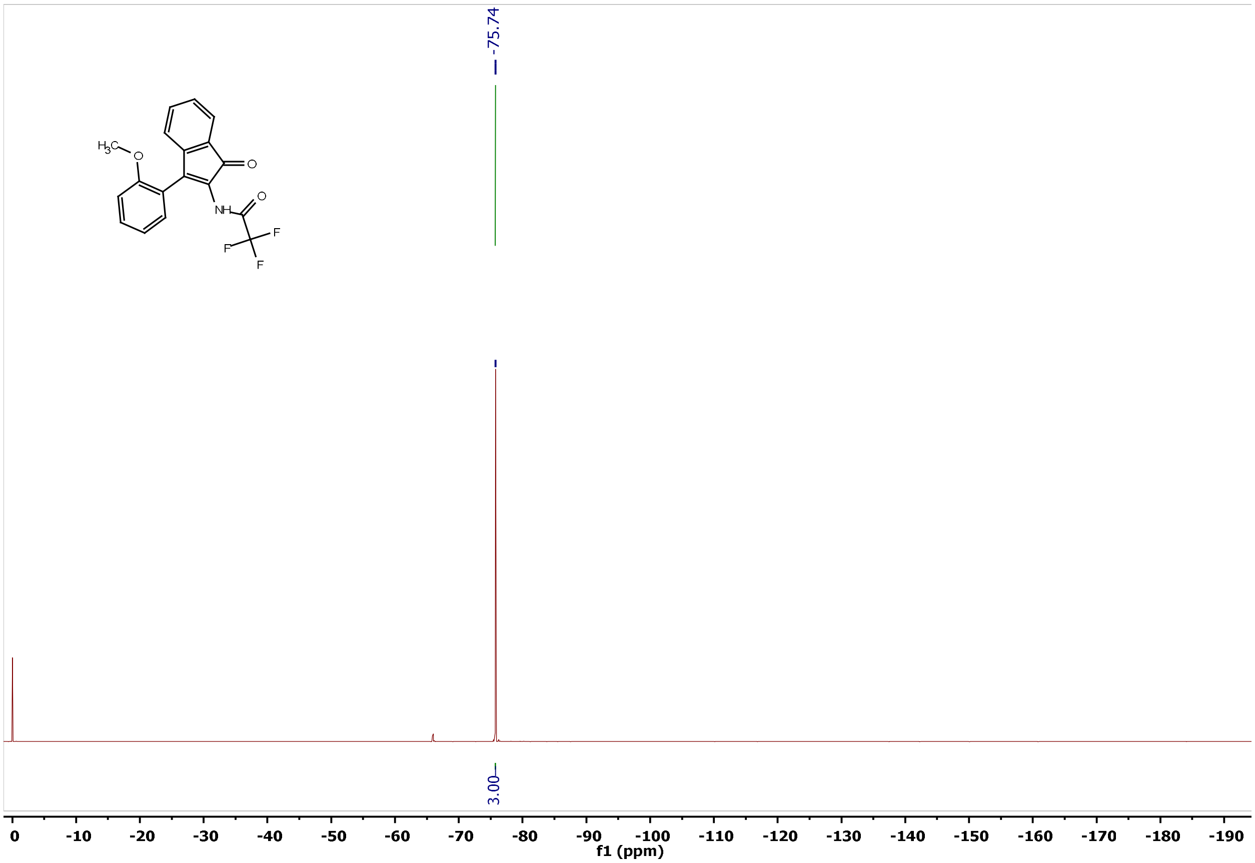

Figure S119. ^{1}H NMR spectrum of **5a** ($CDCl_3$, 401 MHz)

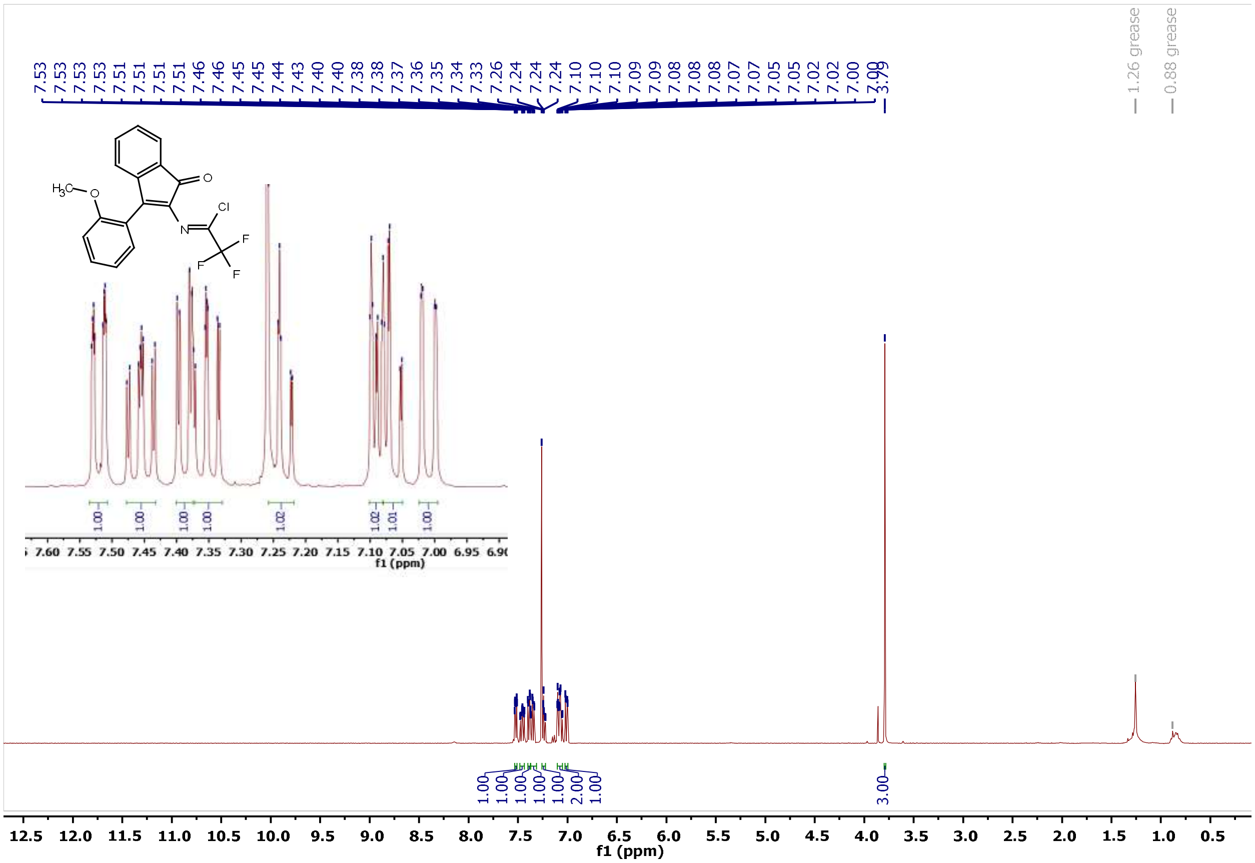

Figure S120. ^{13}C NMR spectrum of **5a** ($CDCl_3$, 151 MHz)

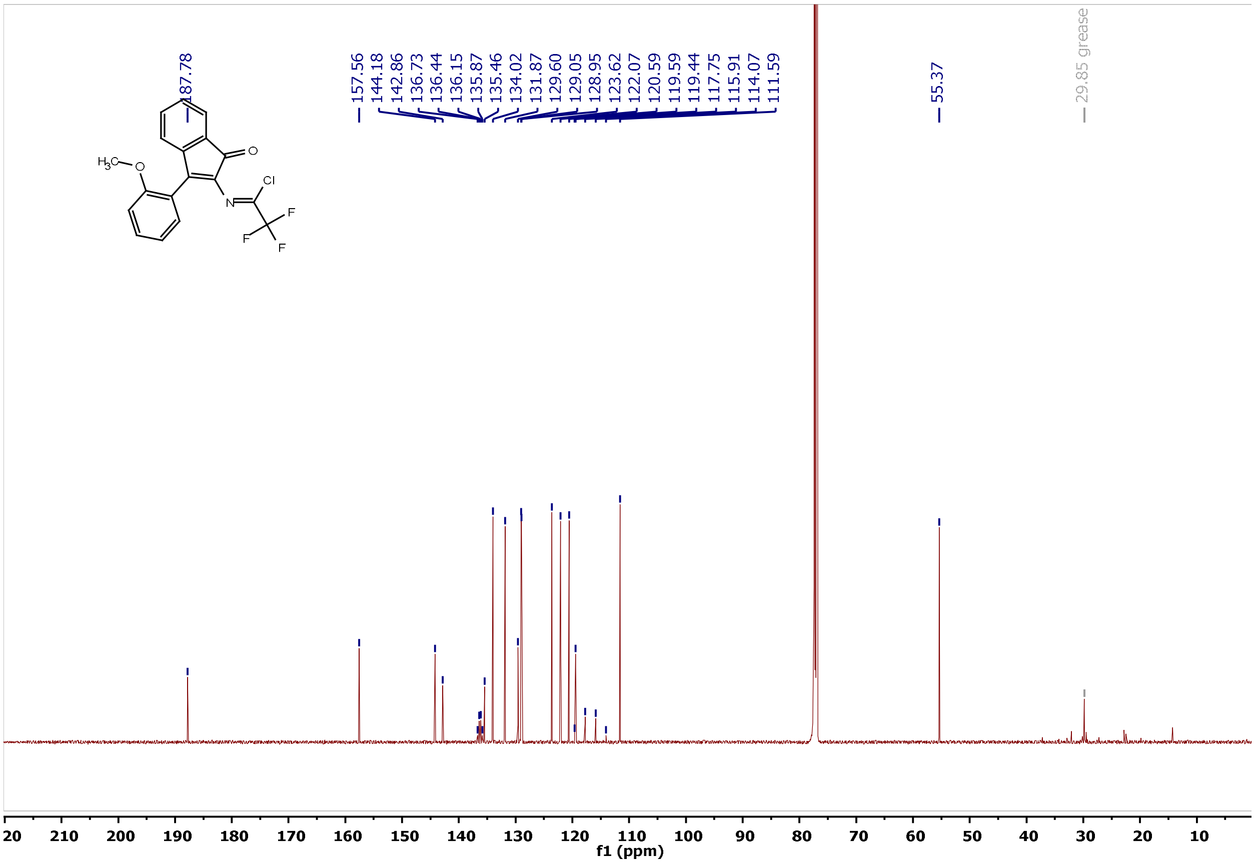

Figure S121. ^{19}F NMR spectrum of **5a** ($CDCl_3$, 377 MHz)

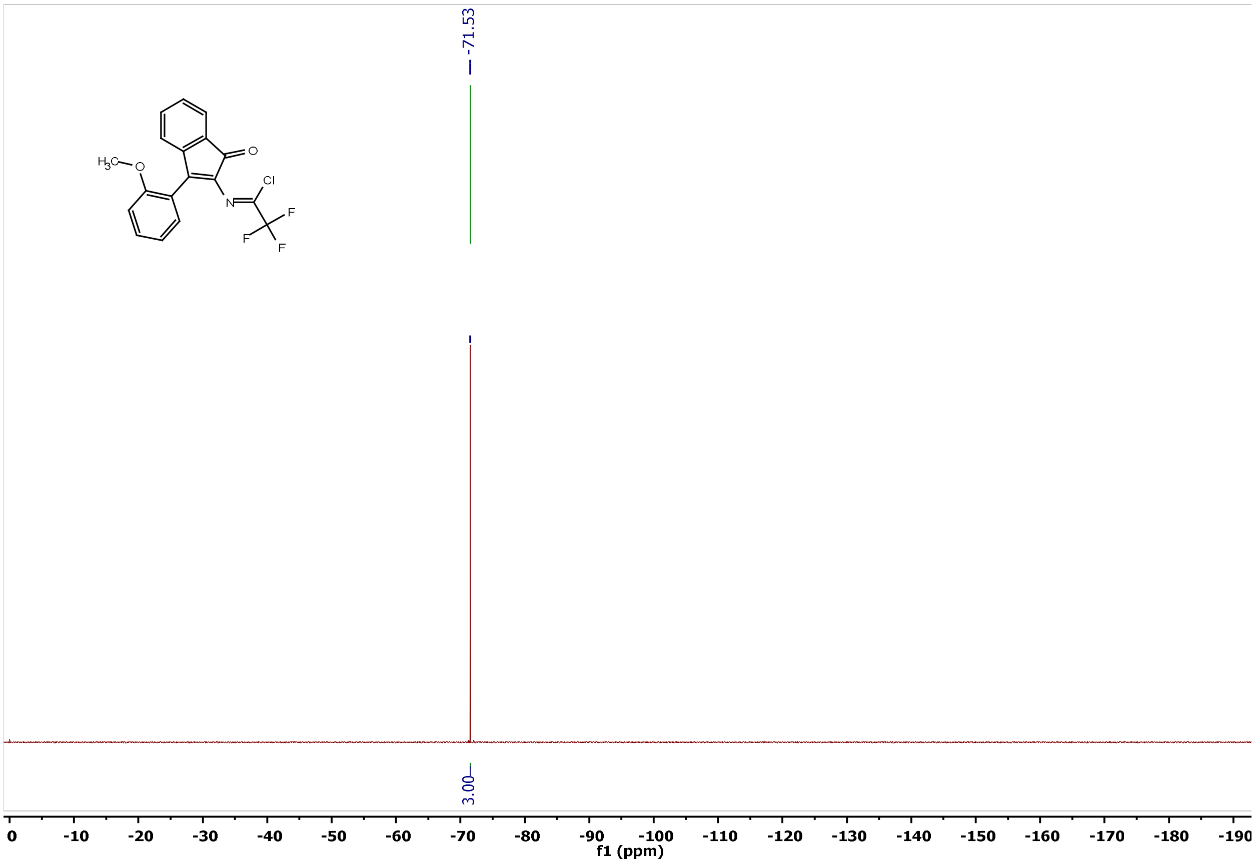

Figure S122. ^{1}H NMR spectrum of **5b** ($CDCl_3$, 401 MHz)

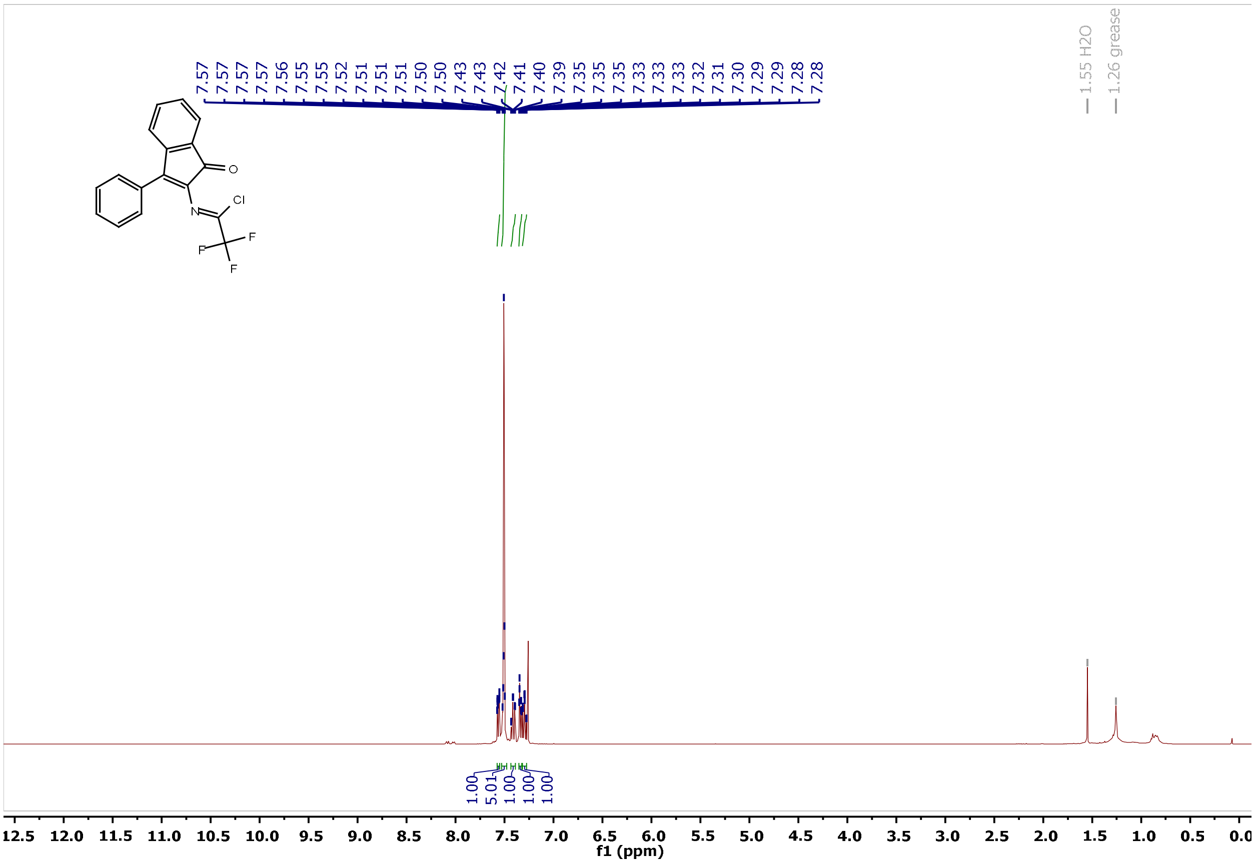

Figure S123. ^{13}C NMR spectrum of **5b** ($CDCl_3$, 101 MHz)

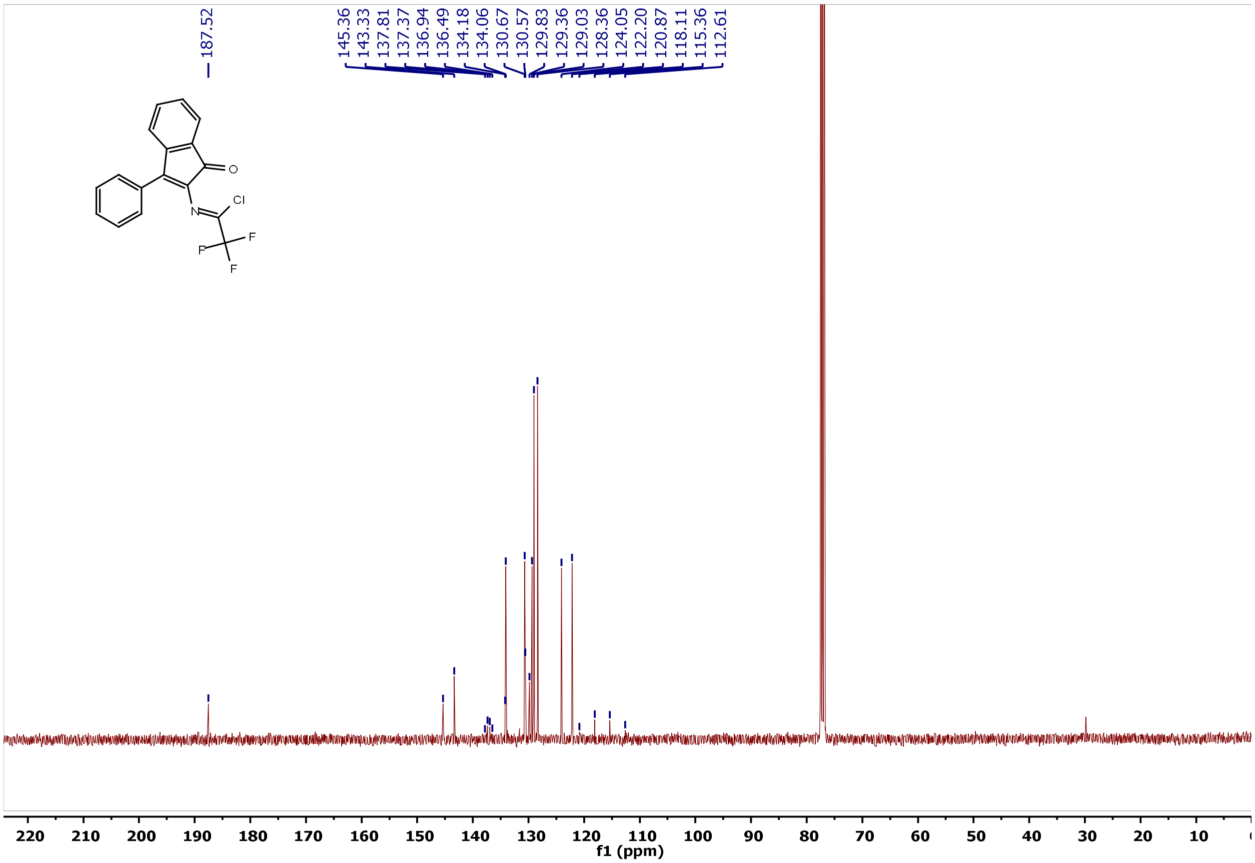

Figure S124. ^{19}F NMR spectrum of **5b** ($CDCl_3$, 471 MHz)

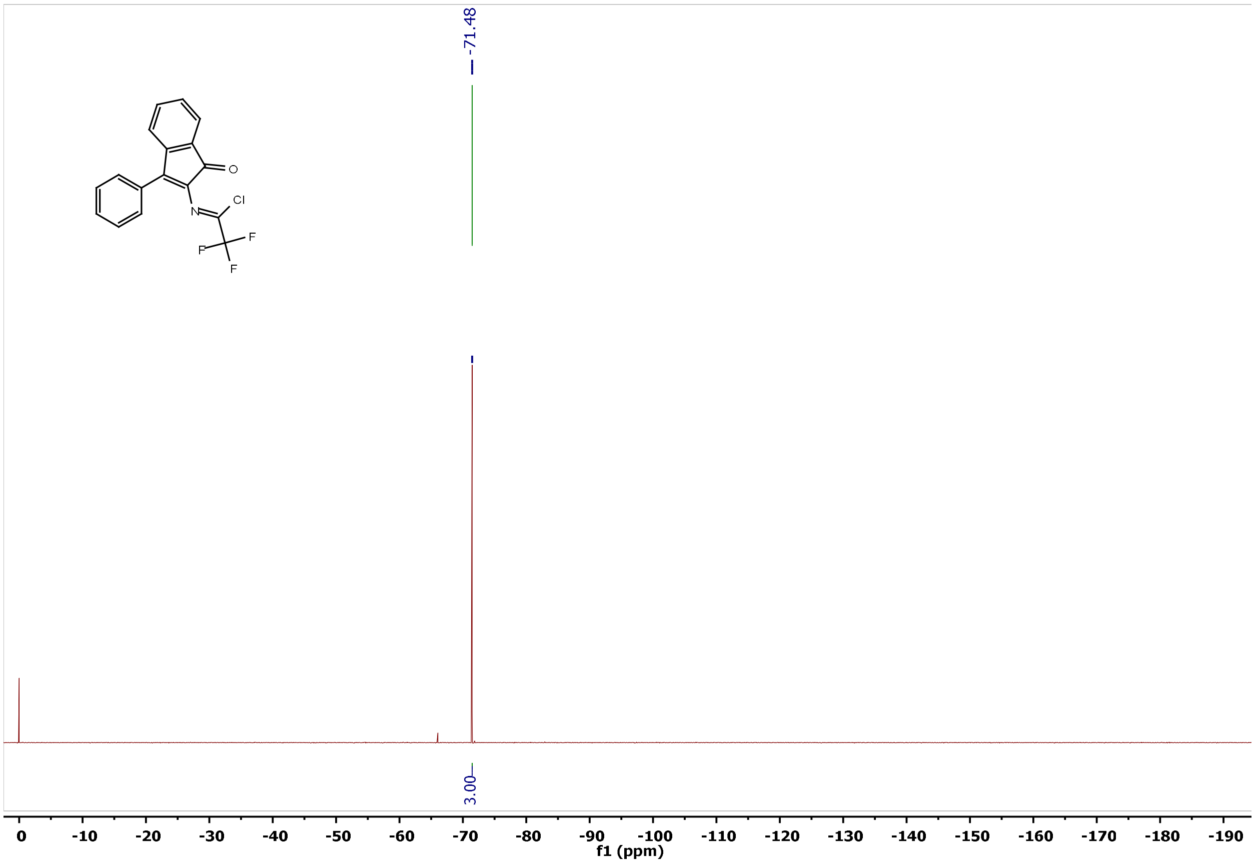

Figure S125. ^{1}H NMR spectrum of **5c** ($CDCl_3$, 401 MHz)

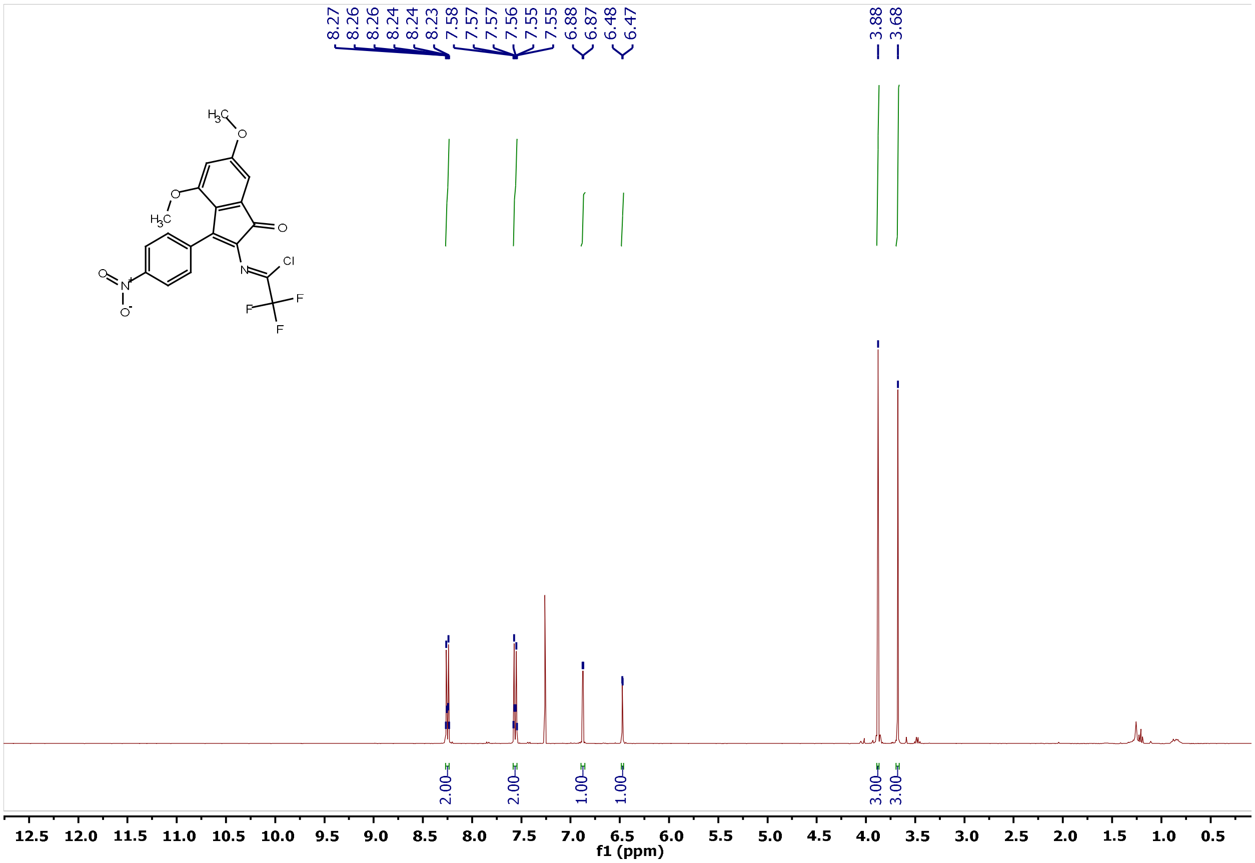

Figure S126. ^{13}C NMR spectrum of **5c** ($CDCl_3$, 126 MHz)

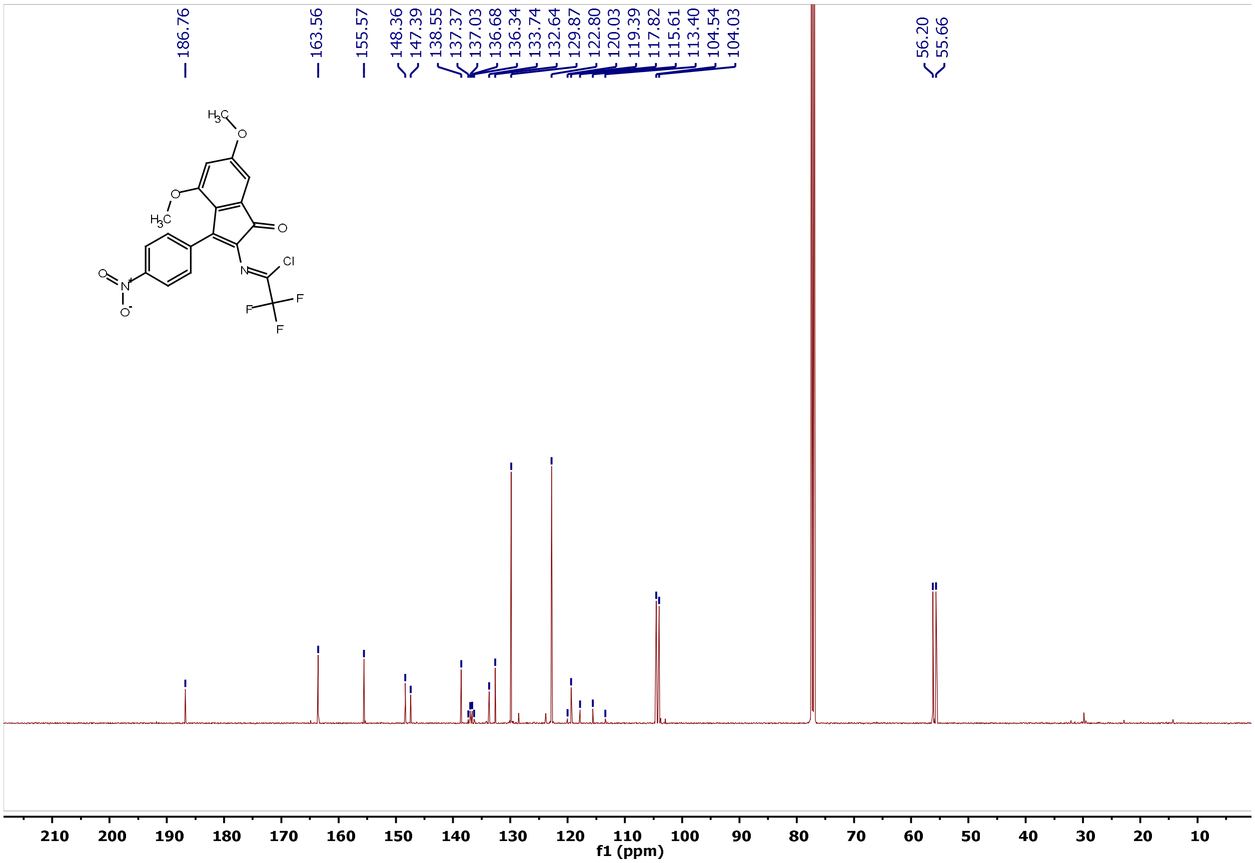

Figure S127. ^{19}F NMR spectrum of **5c** ($CDCl_3$, 377 MHz)

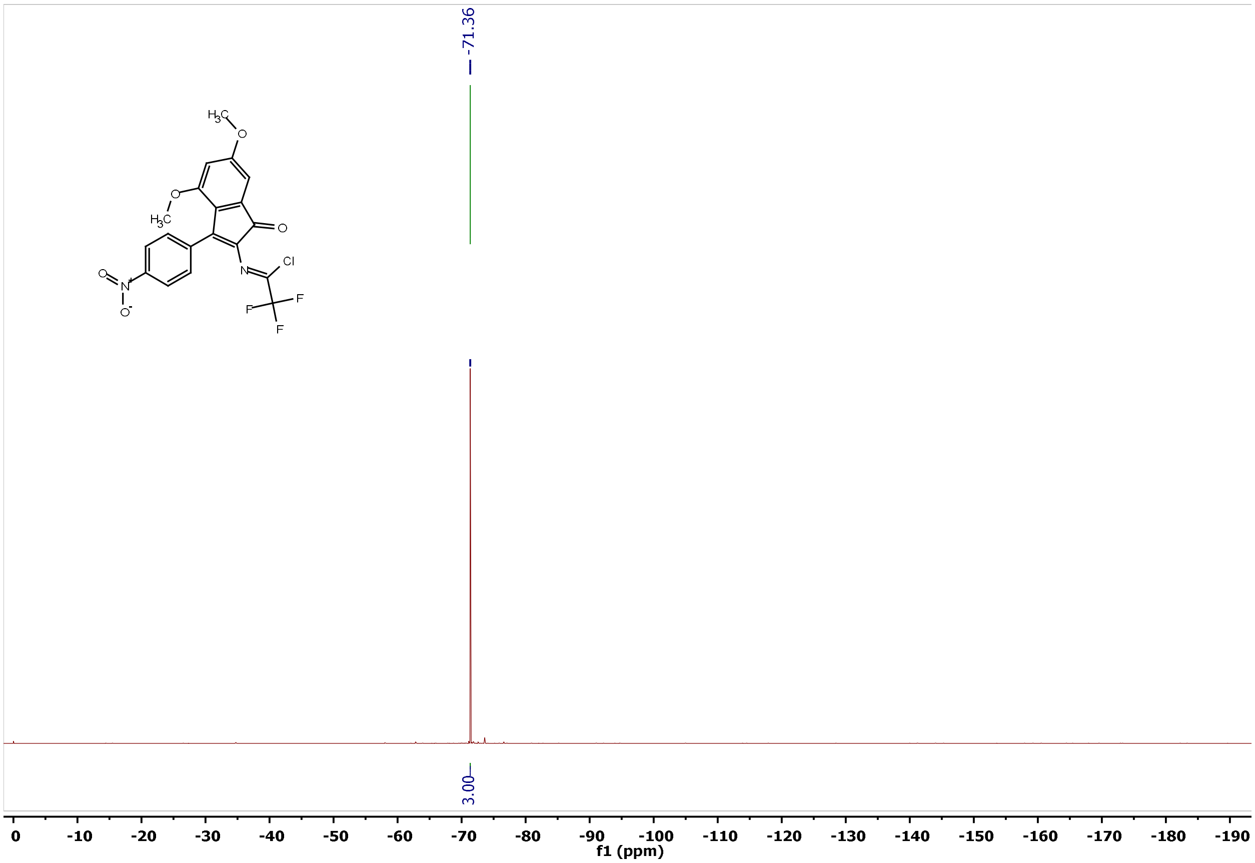

Figure S128. ¹H NMR spectrum of **5d** ($CDCl_3$, 401 MHz)

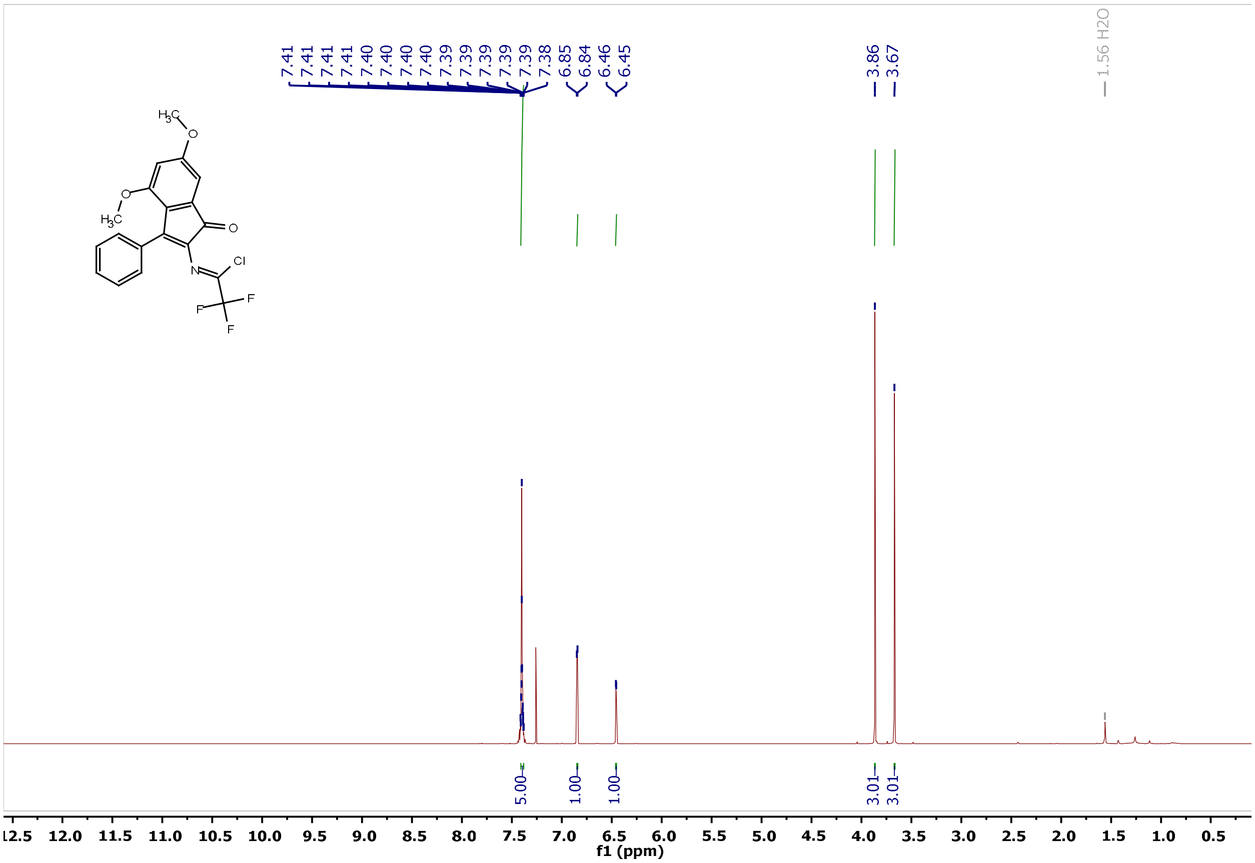

Figure S129. ^{13}C NMR spectrum of **5d** ($CDCl_3$, 101 MHz)

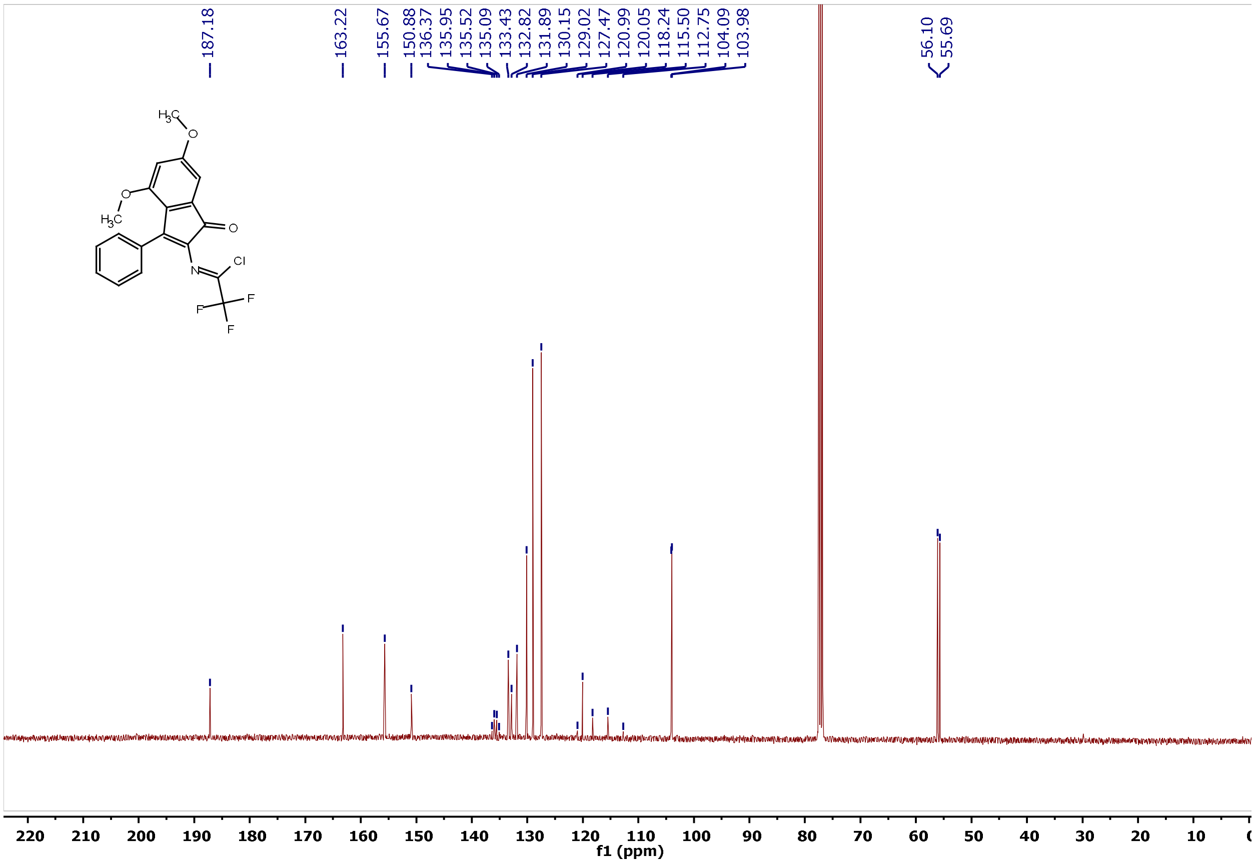

Figure S130. ^{19}F NMR spectrum of **5d** ($CDCl_3$, 377 MHz)

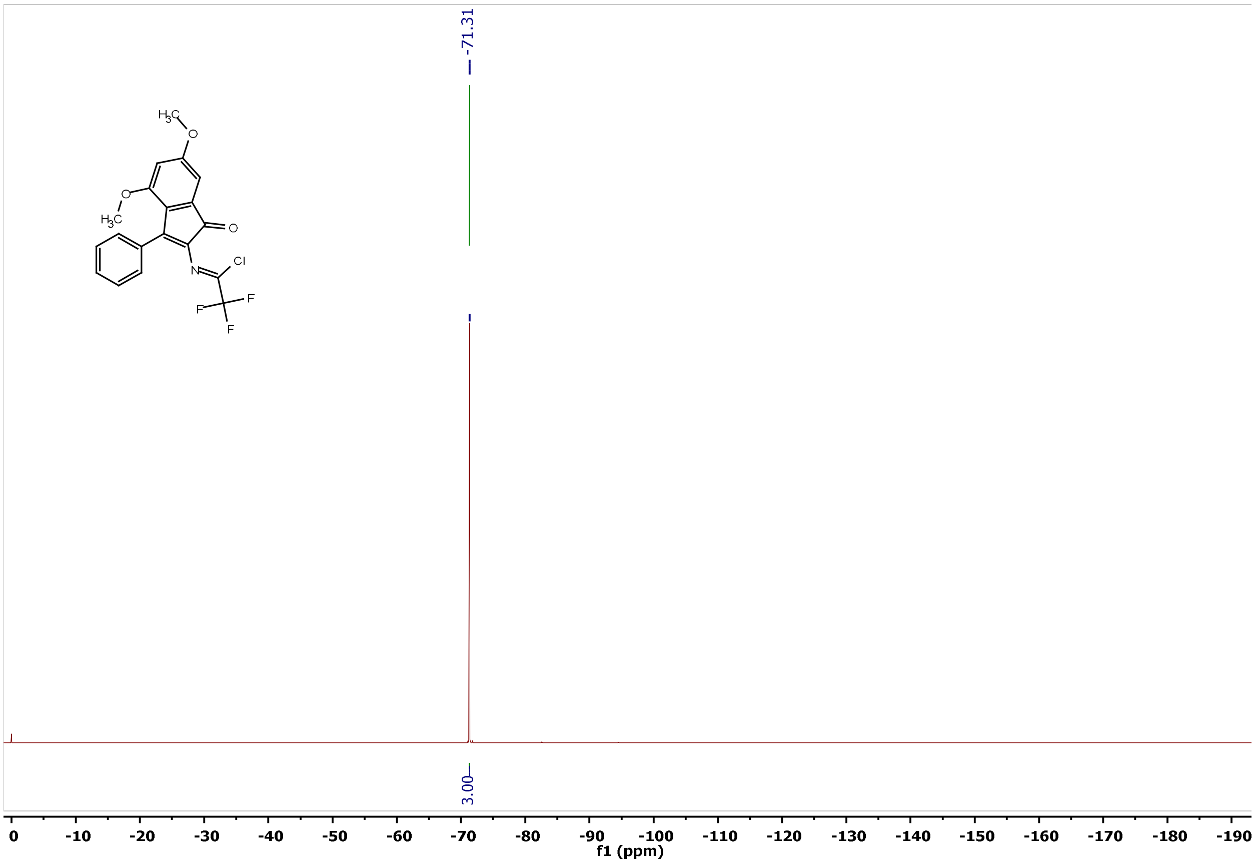

Figure S131. ^{1}H NMR spectrum of **5e** ($CDCl_3$, 401 MHz)

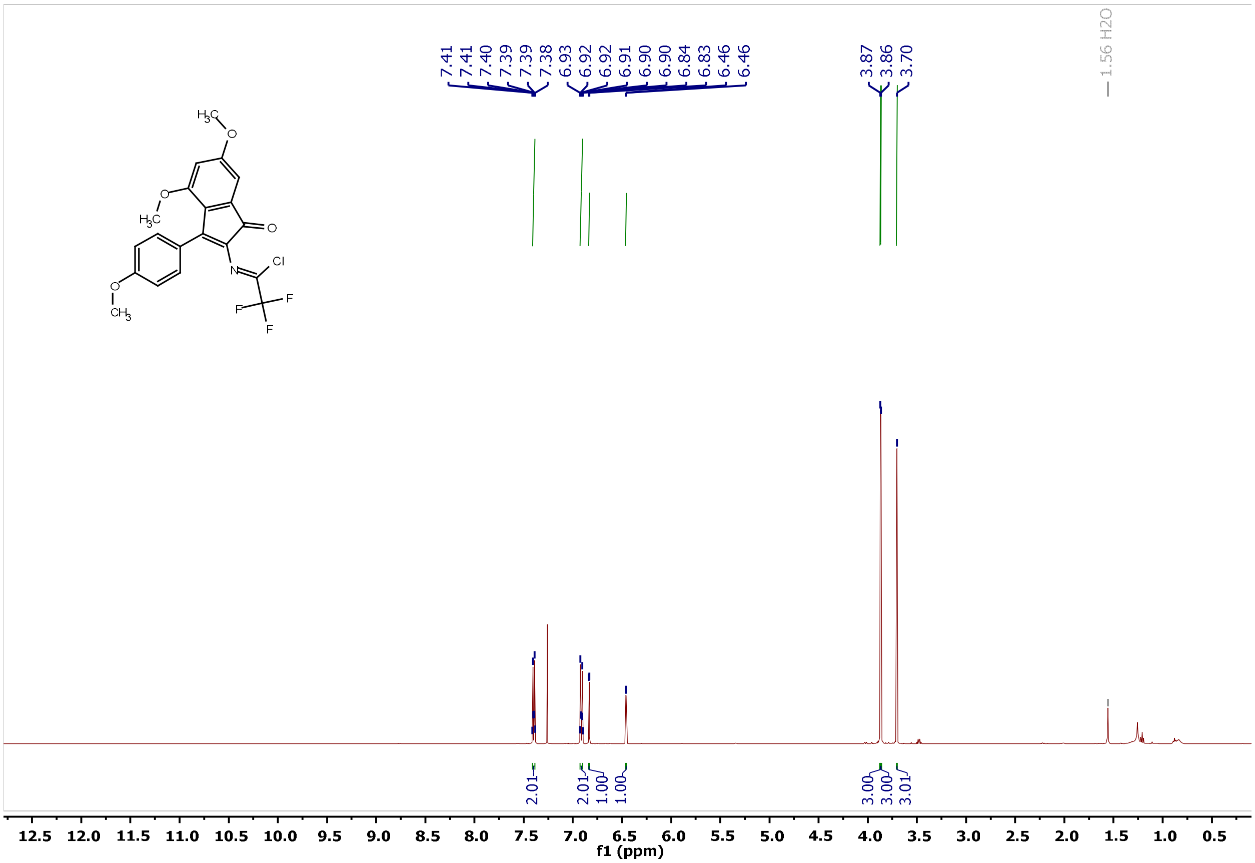

Figure S132. ^{13}C NMR spectrum of **5e** ($CDCl_3$, 126 MHz)

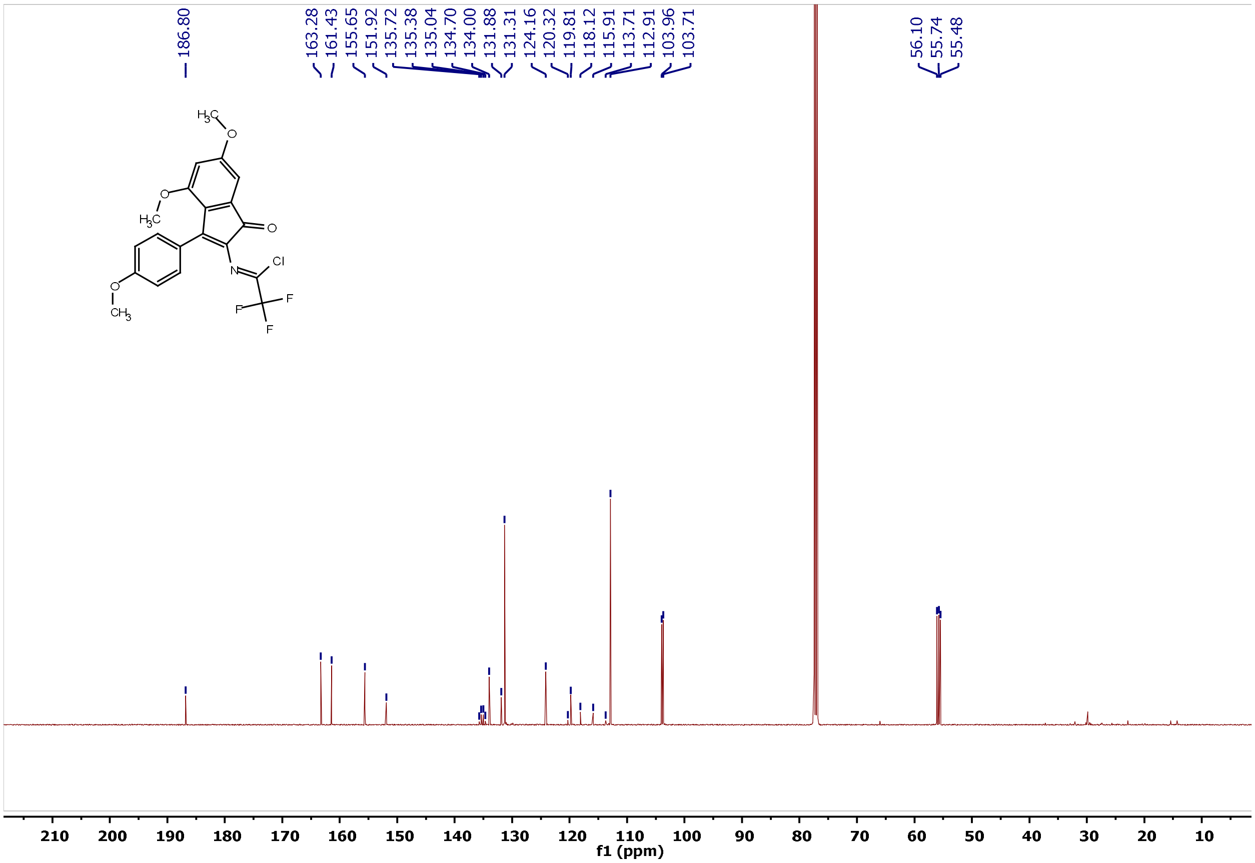

Figure S133. ^{19}F NMR spectrum of **5e** ($CDCl_3$, 377 MHz)

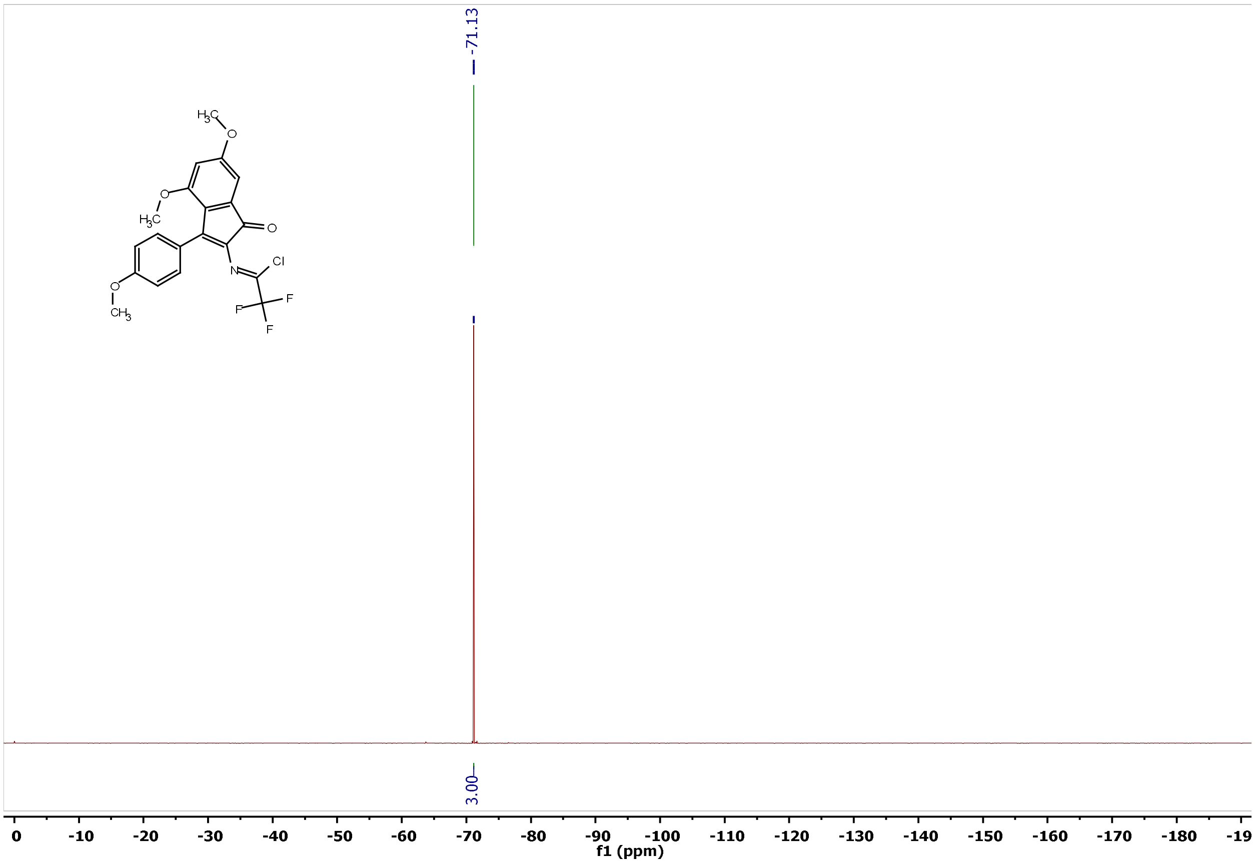

Figure S134. ^{1}H NMR spectrum of **6a** ($CDCl_3$, 401 MHz)

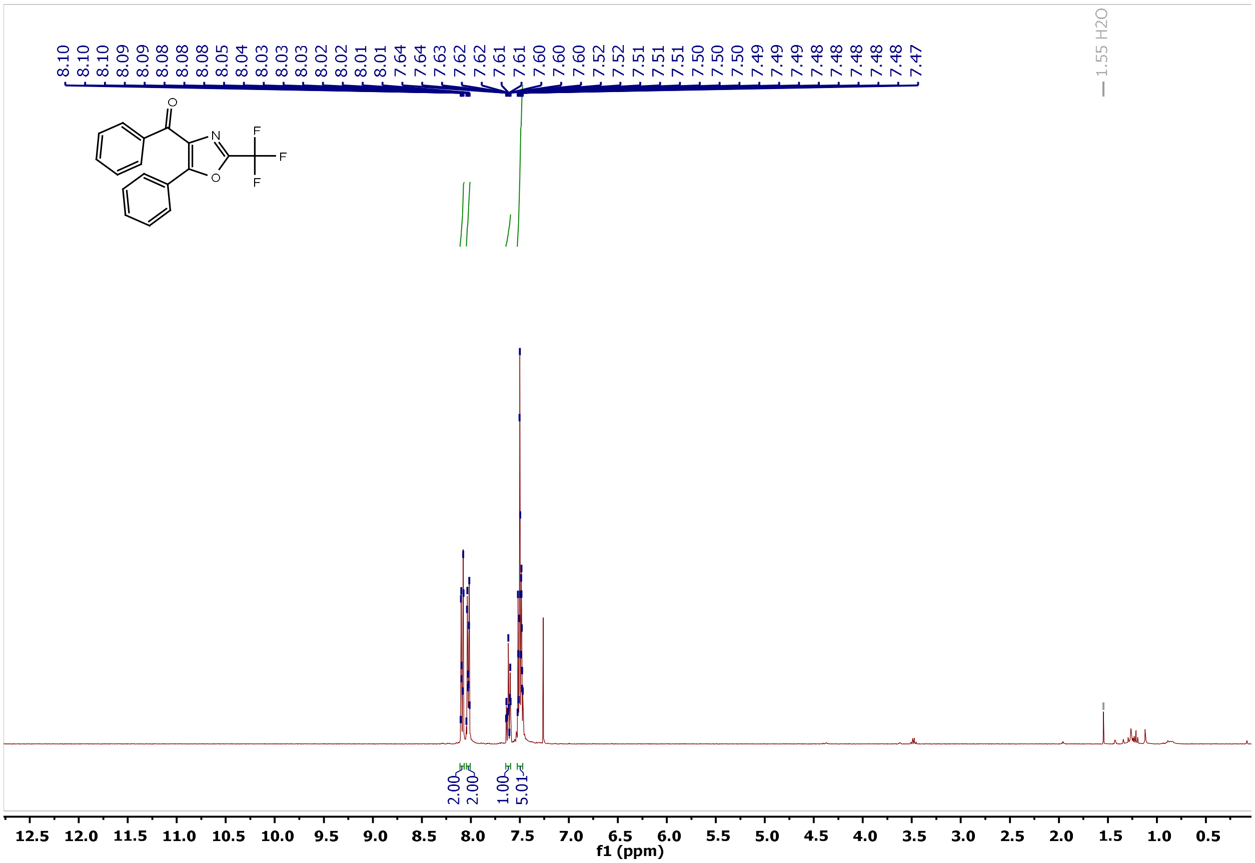

Figure S135. ^{13}C NMR spectrum of **6a** ($CDCl_3$, 101 MHz)

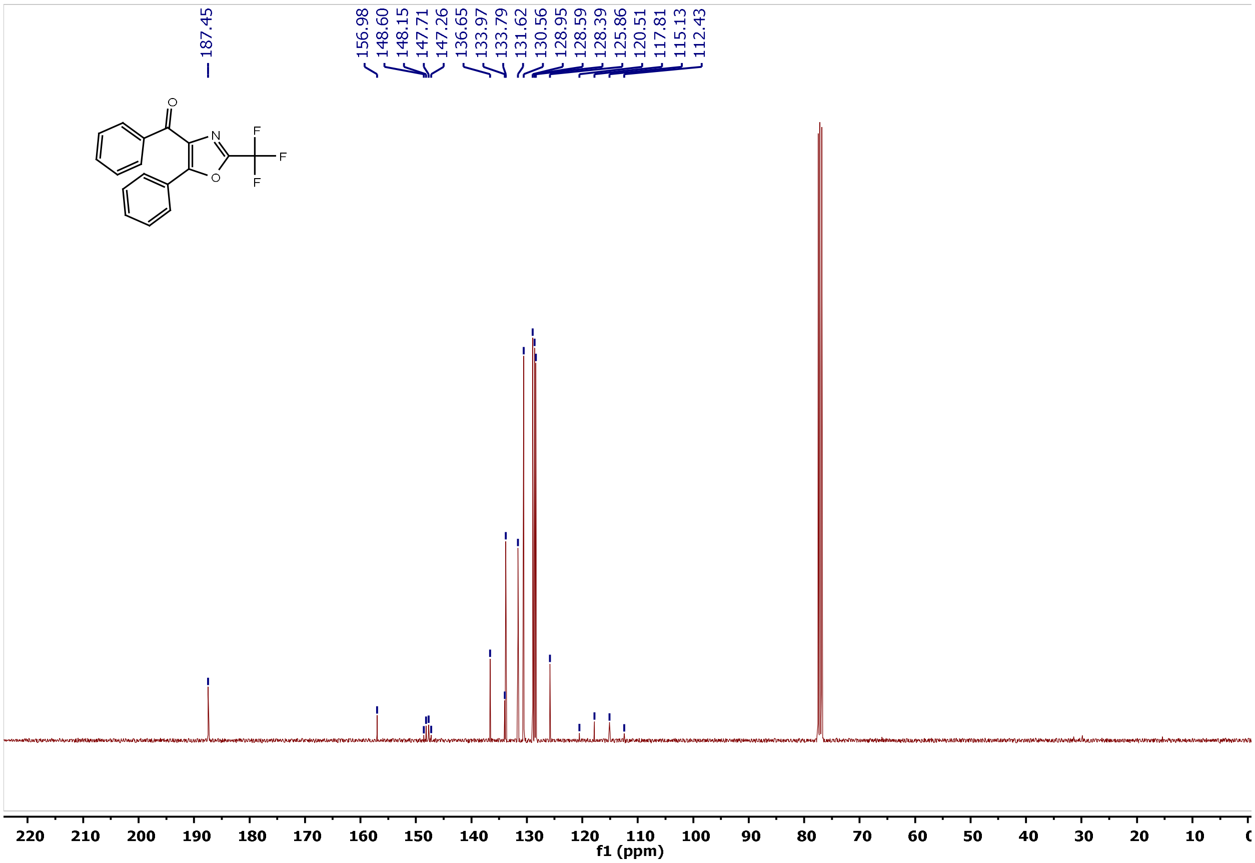

Figure S136. ^{19}F NMR spectrum of **6a** ($CDCl_3$, 377 MHz)

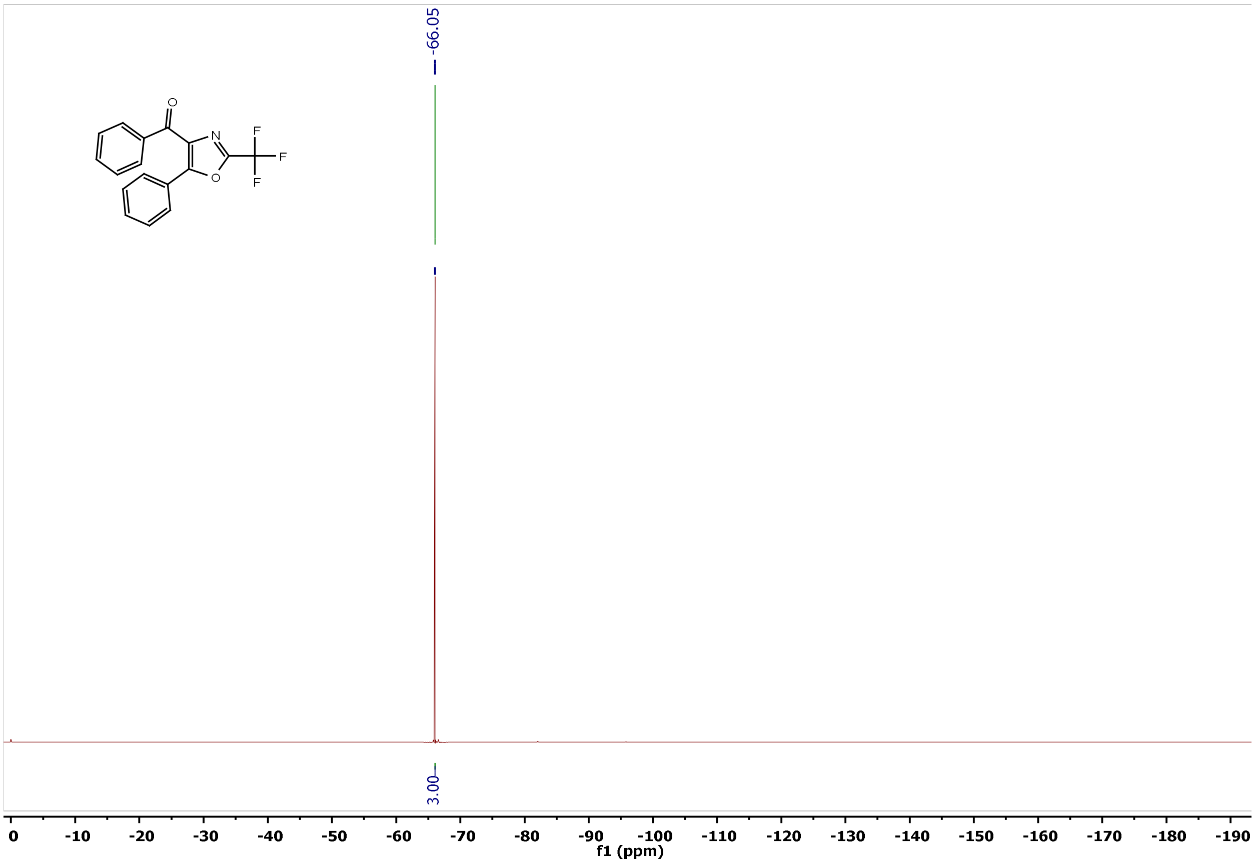

Figure S137. ^{1}H NMR spectrum of **6b** ($CDCl_3$, 401 MHz)

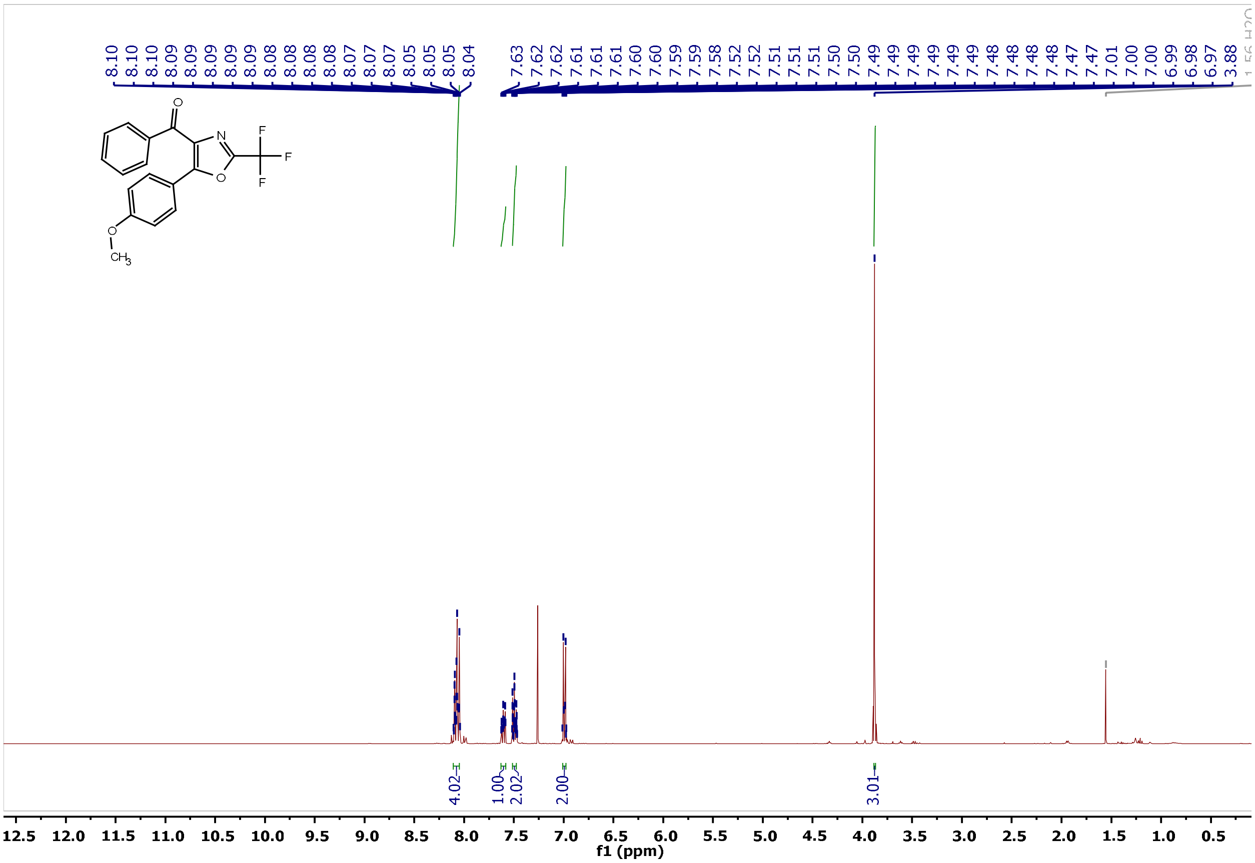

Figure S138. ^{13}C NMR spectrum of **6b** ($CDCl_3$, 101 MHz)

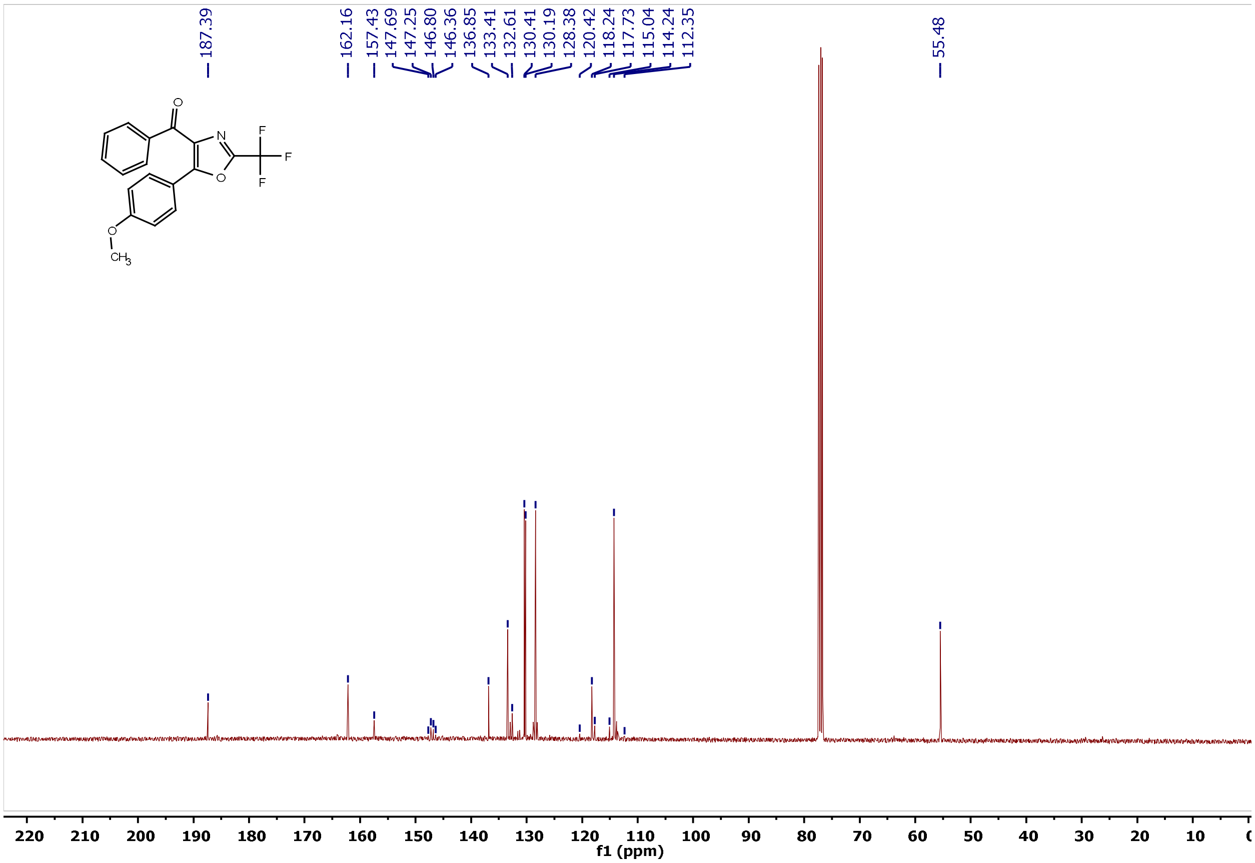

Figure S139. ^1H-^{13}C HMBC spectrum of **6b**

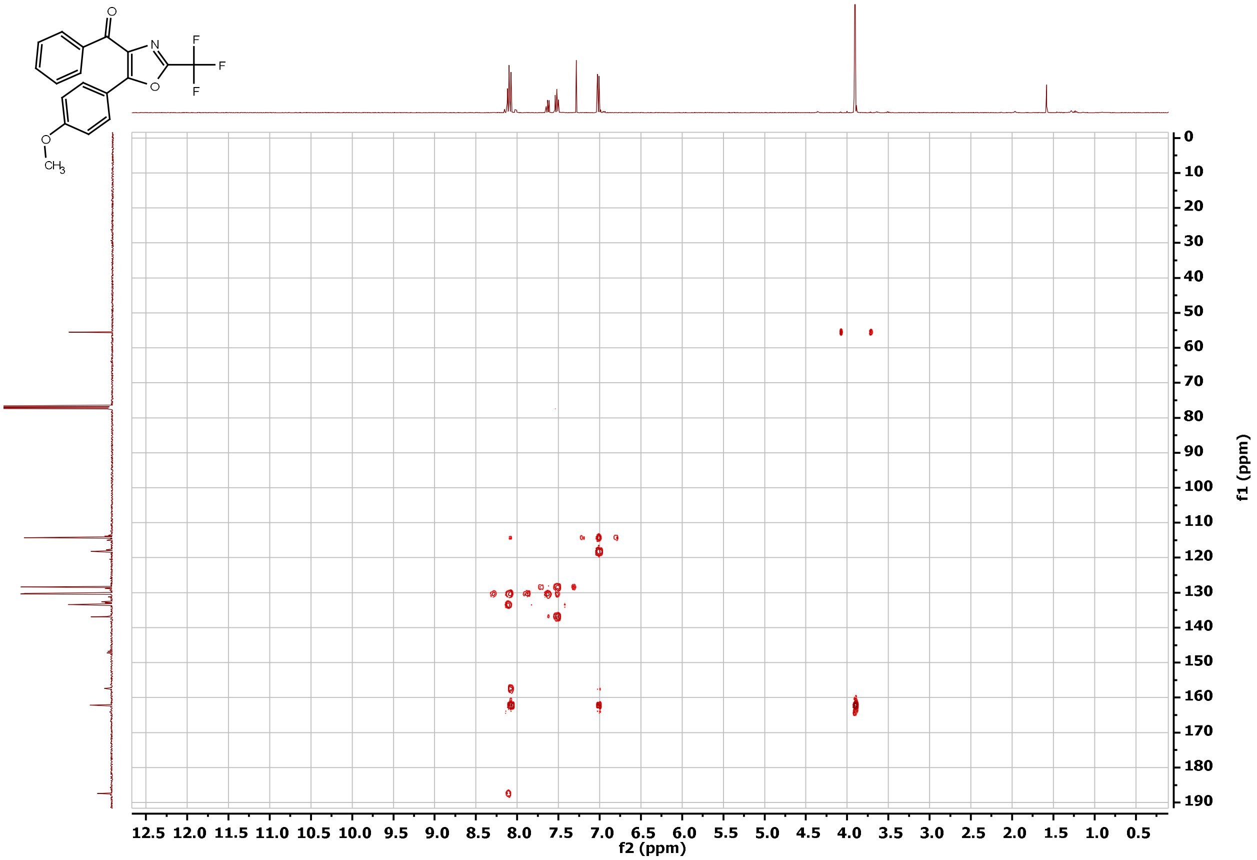

Figure S140. ^{19}F NMR spectrum of **6b** ($CDCl_3$, 377 MHz)

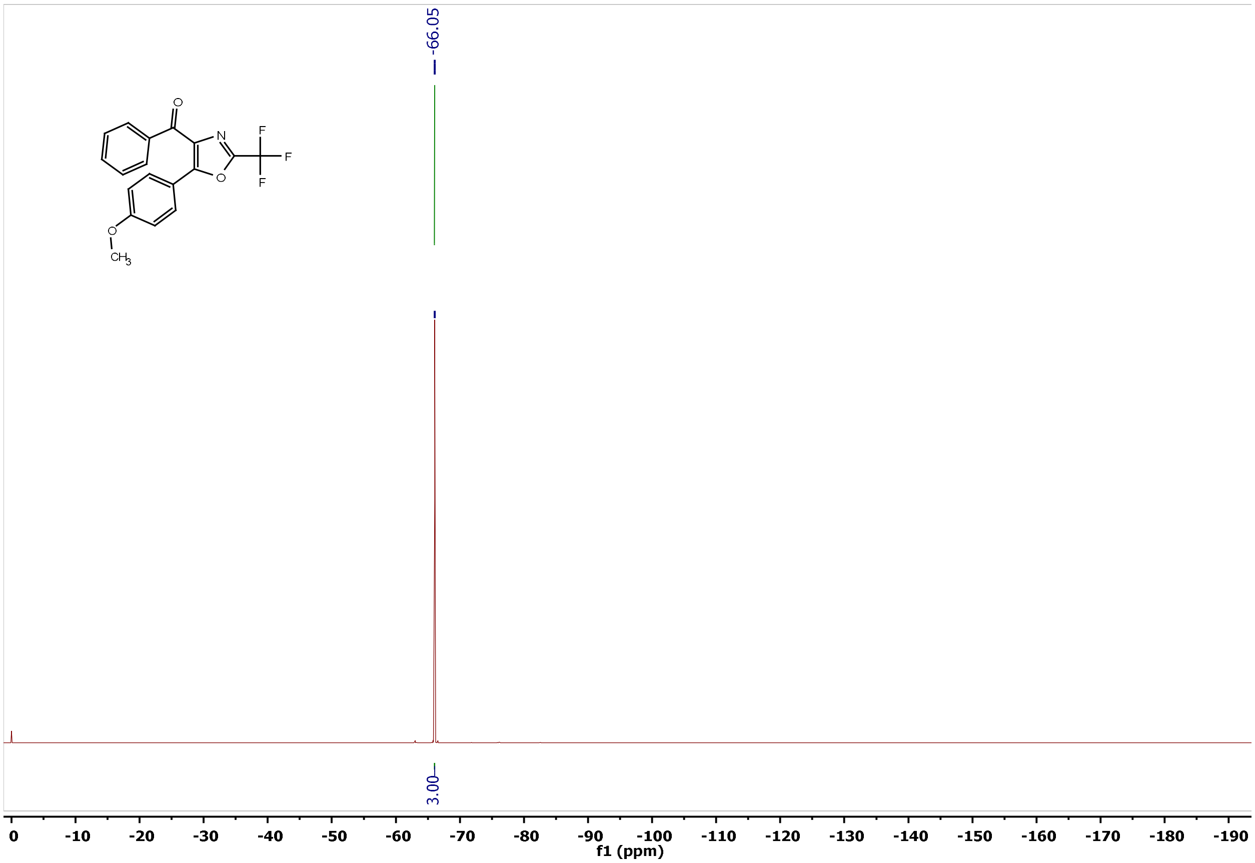

Figure S141. ^{1}H NMR spectrum of **6c** ($CDCl_3$, 401 MHz)

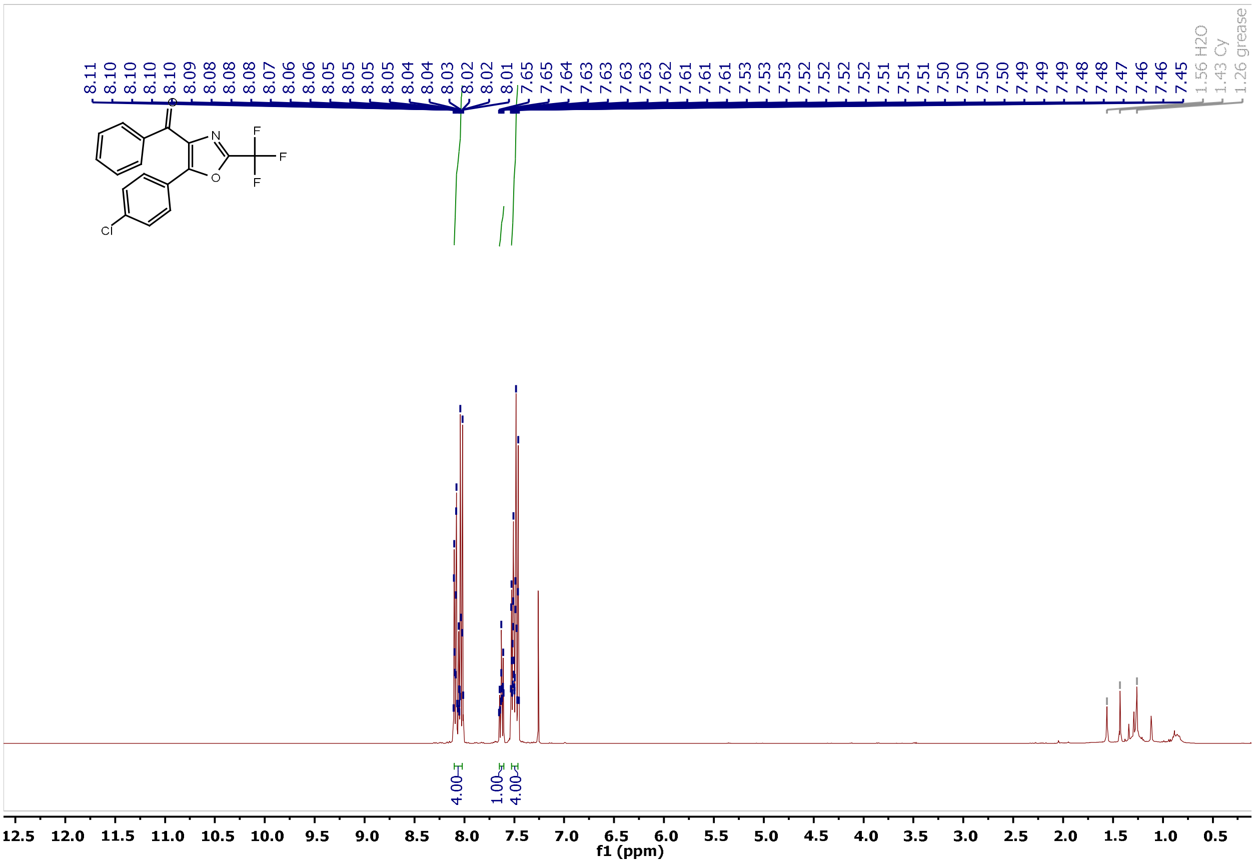

Figure S142. ^{13}C NMR spectrum of **6c** ($CDCl_3$, 101 MHz)

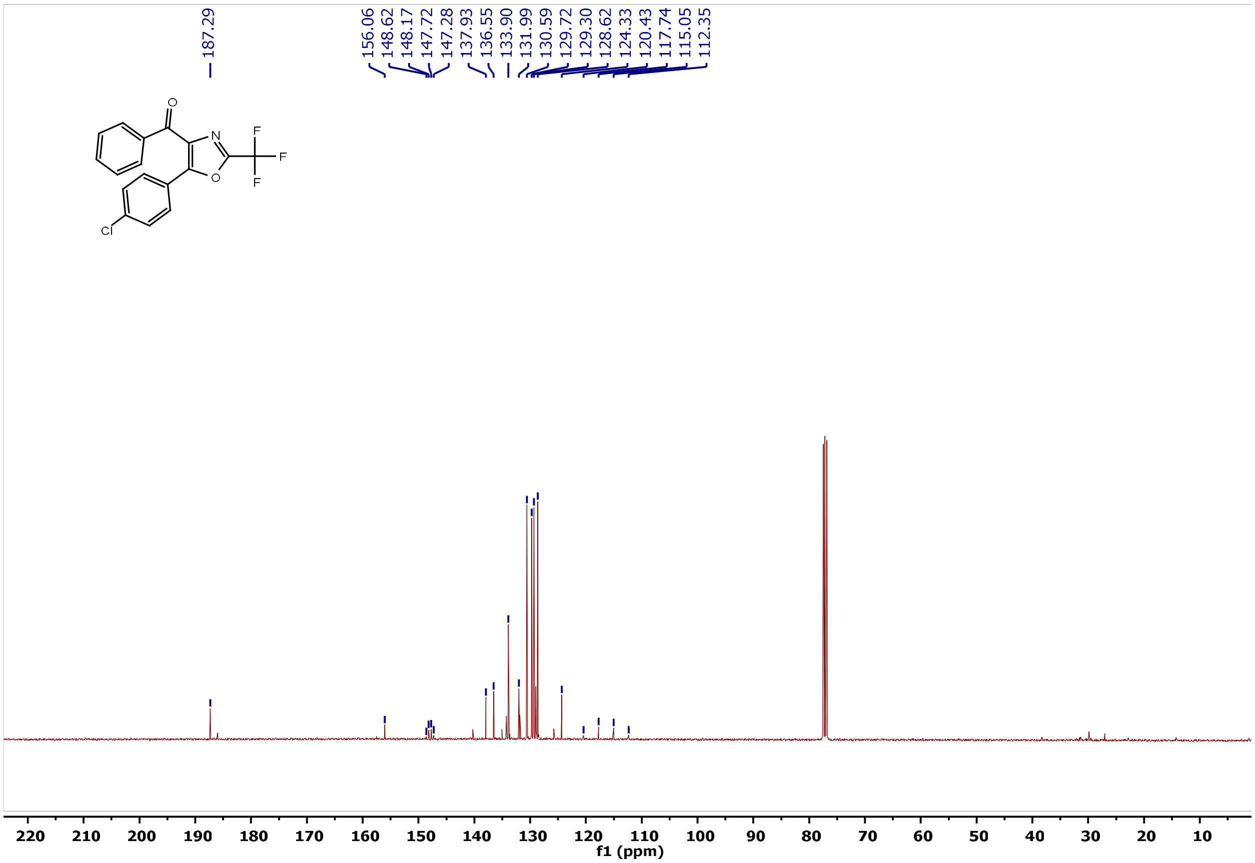

Figure S143. ^{19}F NMR spectrum of **6c** ($CDCl_3$, 377 MHz)

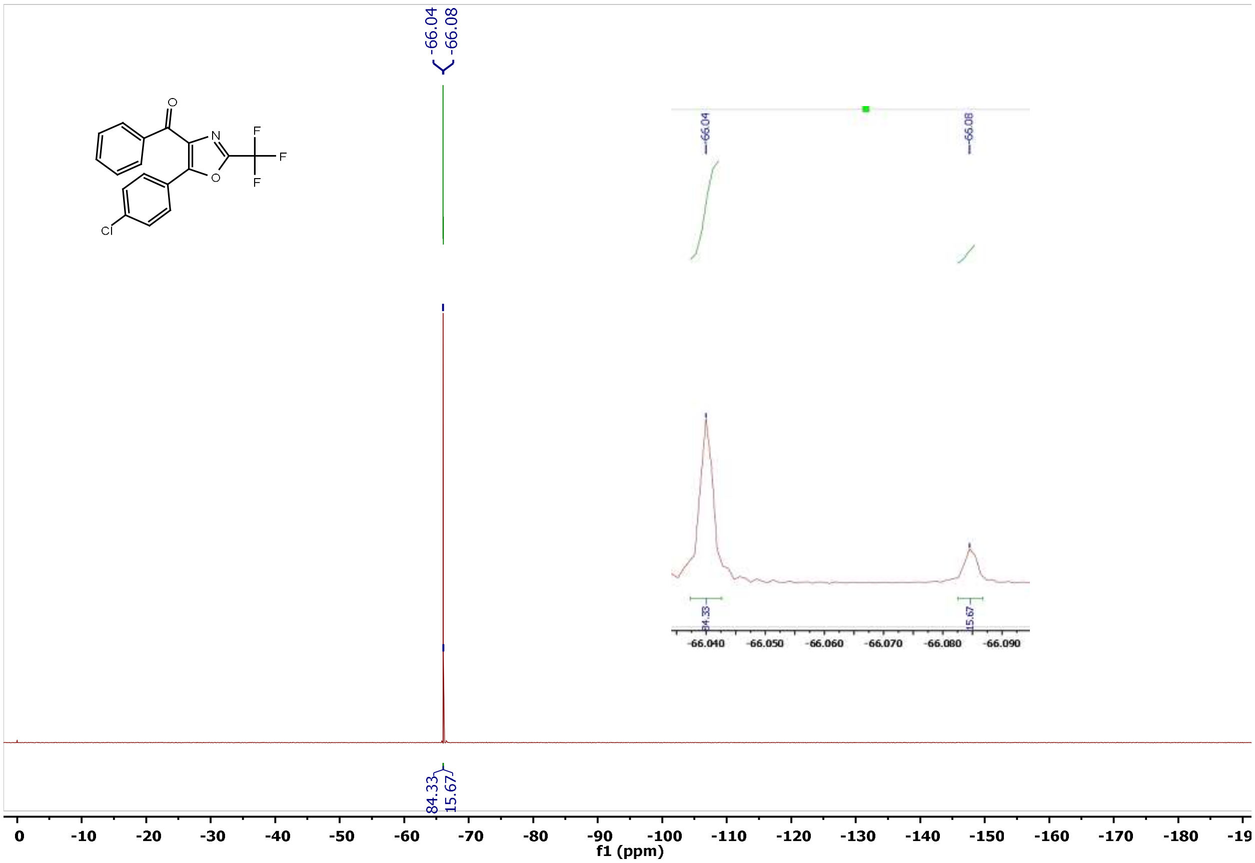

Figure S144. ^{1}H NMR spectrum of **6d** ($CDCl_3$, 401 MHz)

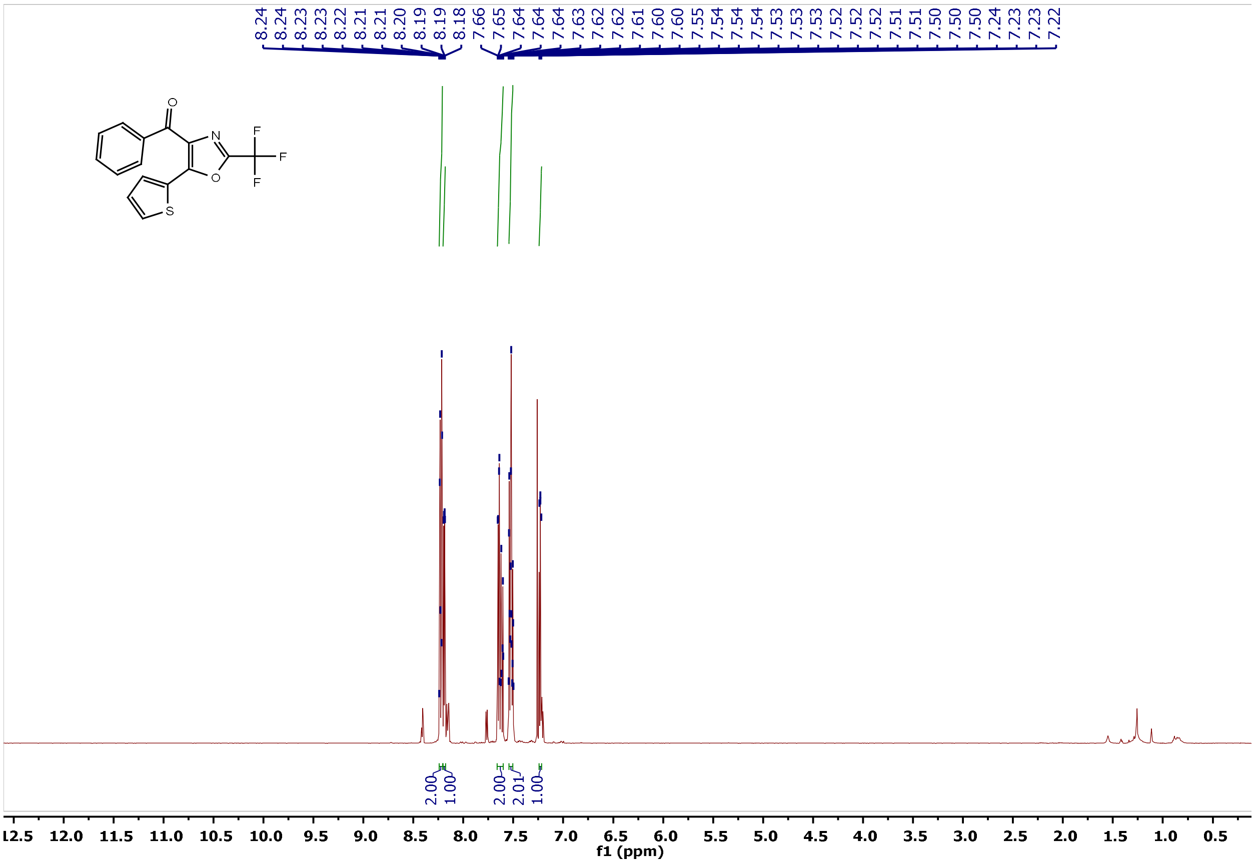

Figure S145. ^{13}C NMR spectrum of **6d** ($CDCl_3$, 101 MHz)

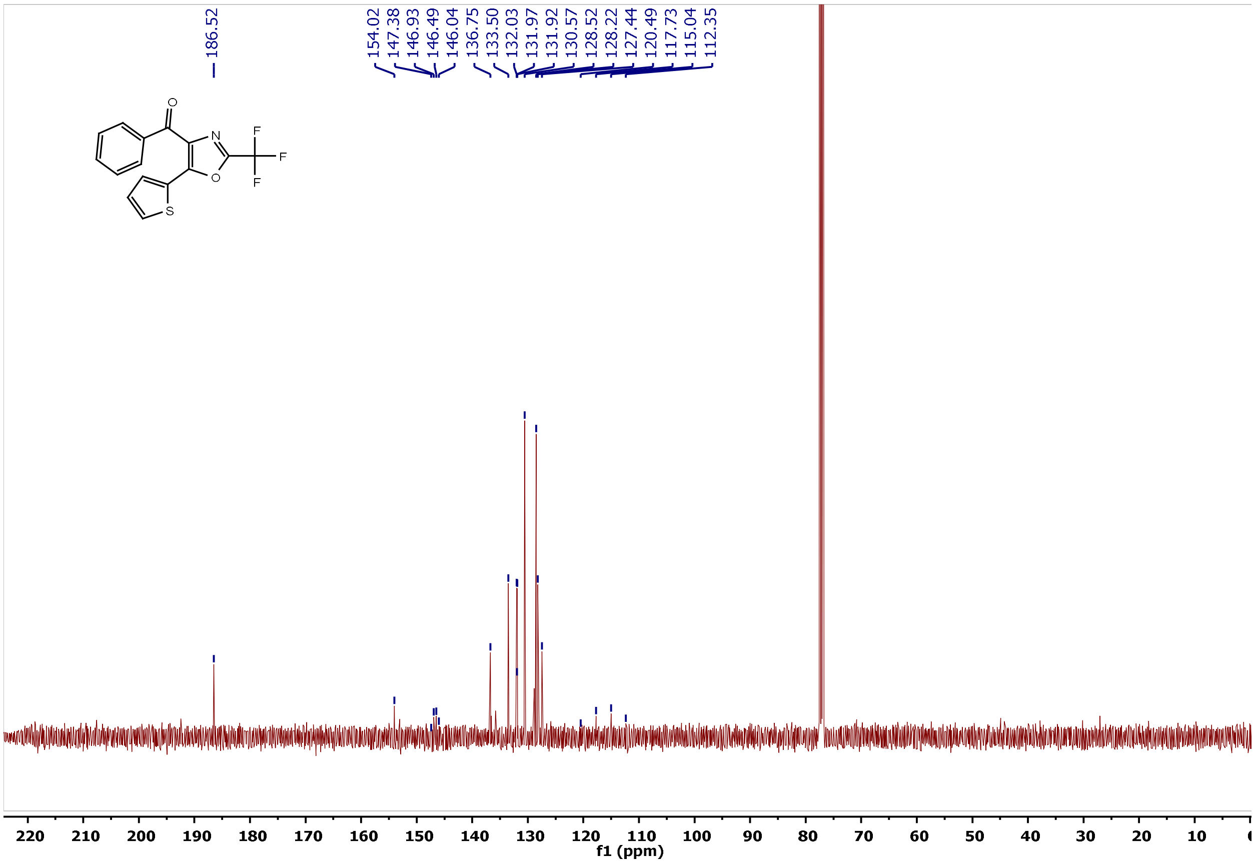

Figure S146. ^{19}F NMR spectrum of **6d** ($CDCl_3$, 377 MHz)

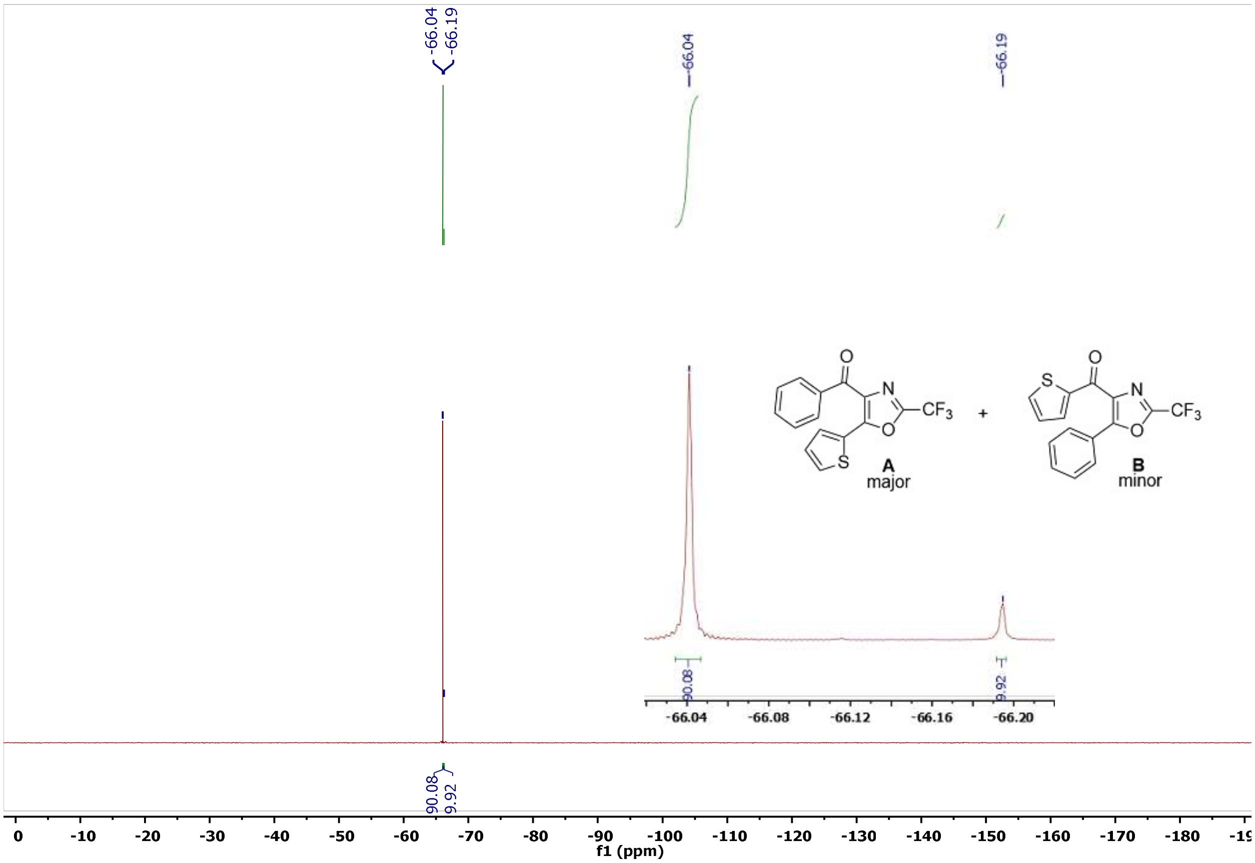

Figure S147. ^{1}H NMR spectrum of **6e** ($CDCl_3$, 401 MHz)

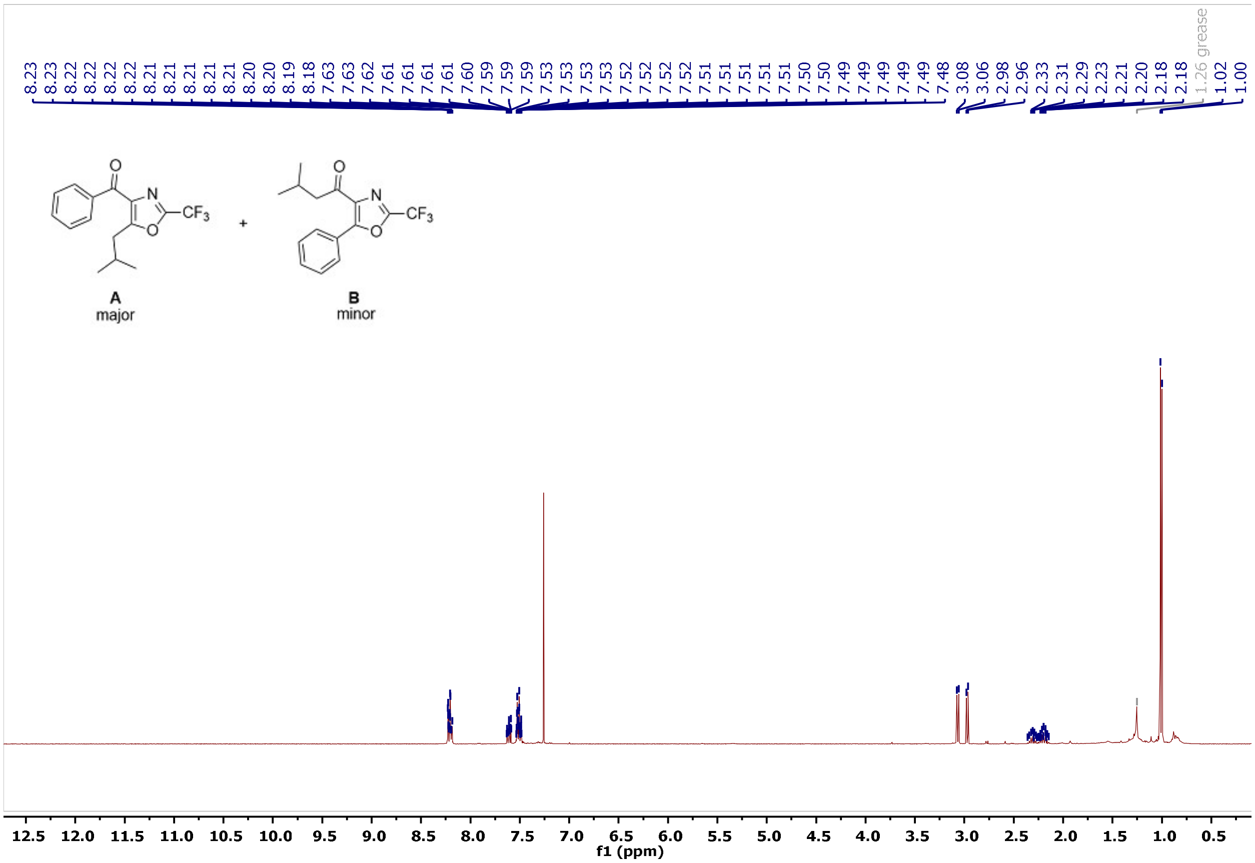

Figure S148. ^{13}C NMR spectrum of **6e** ($CDCl_3$, 126 MHz)

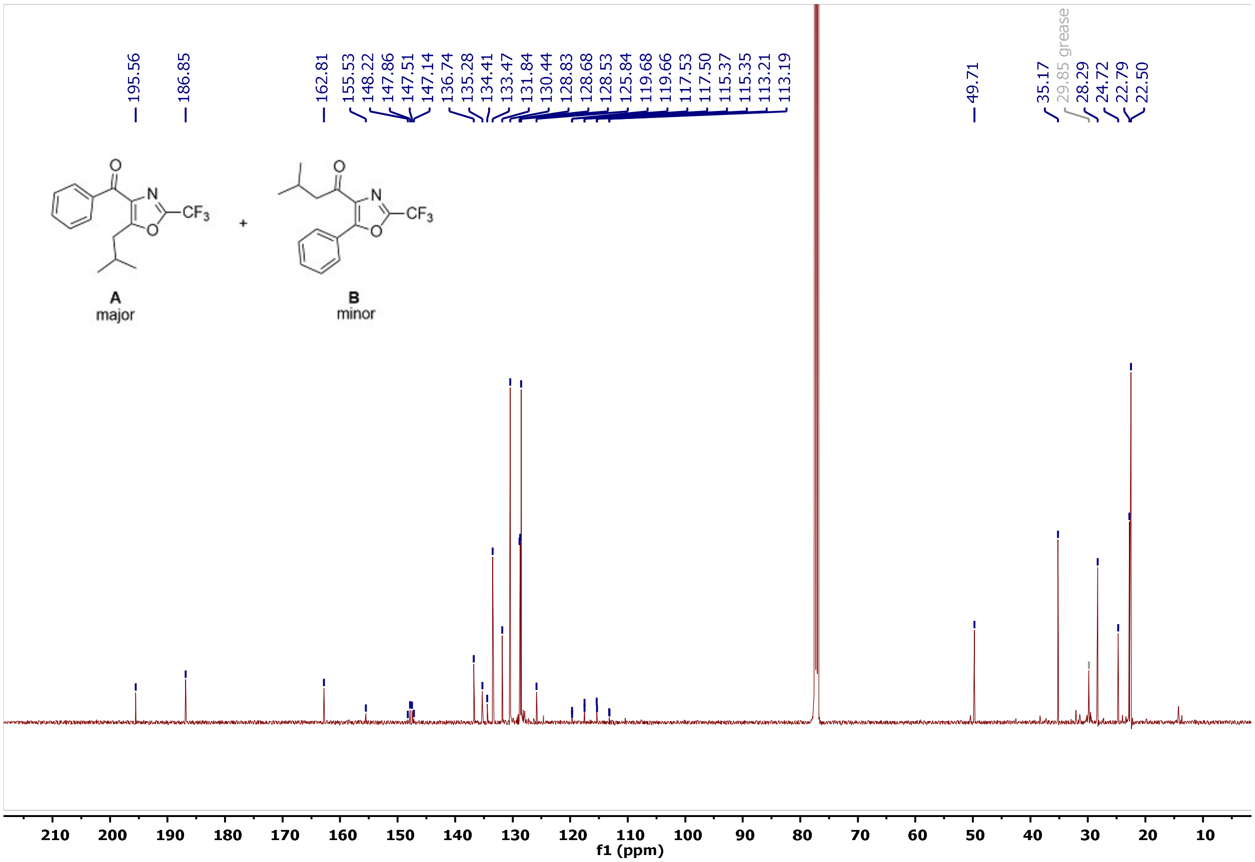

Figure S149. ^{19}F NMR spectrum of **6e** ($CDCl_3$, 377 MHz)

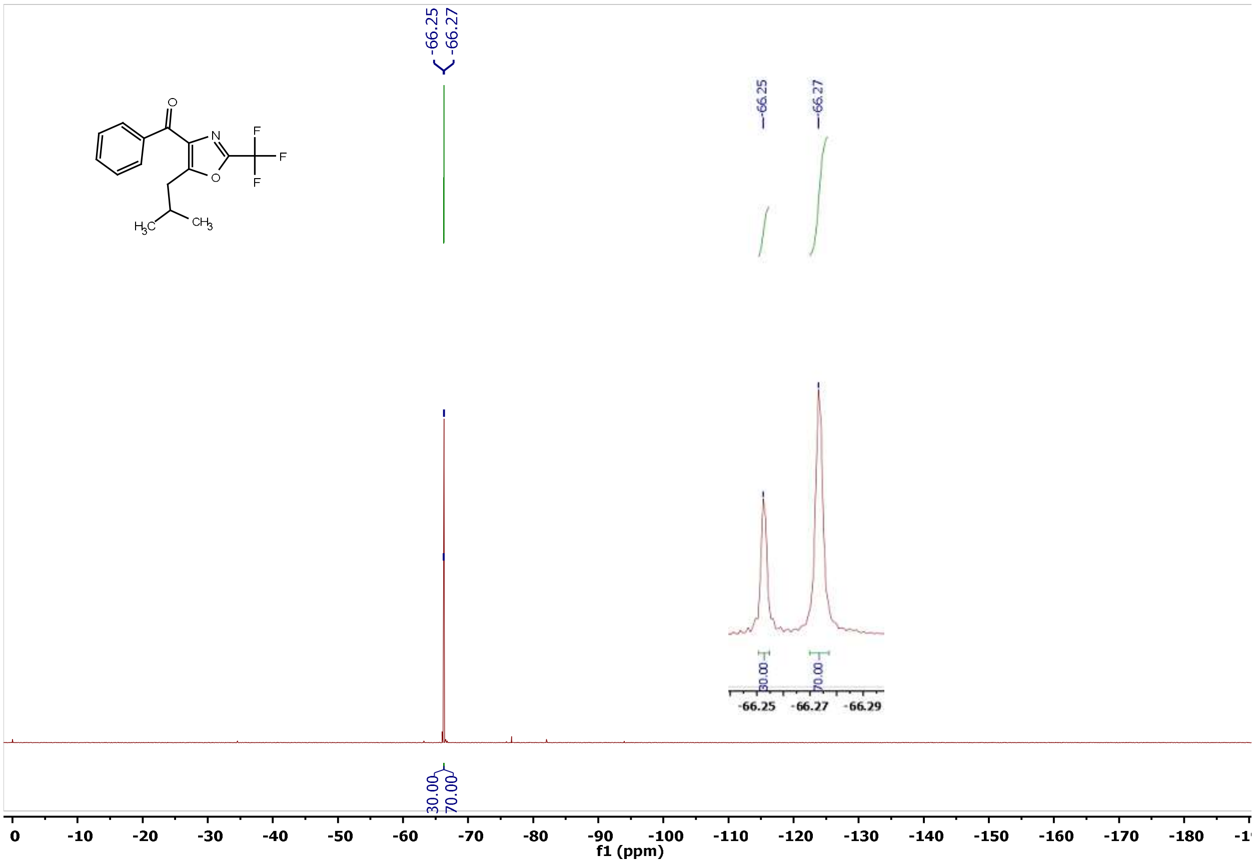

Figure S150. ^{1}H NMR spectrum of **6f** ($CDCl_3$, 401 MHz)

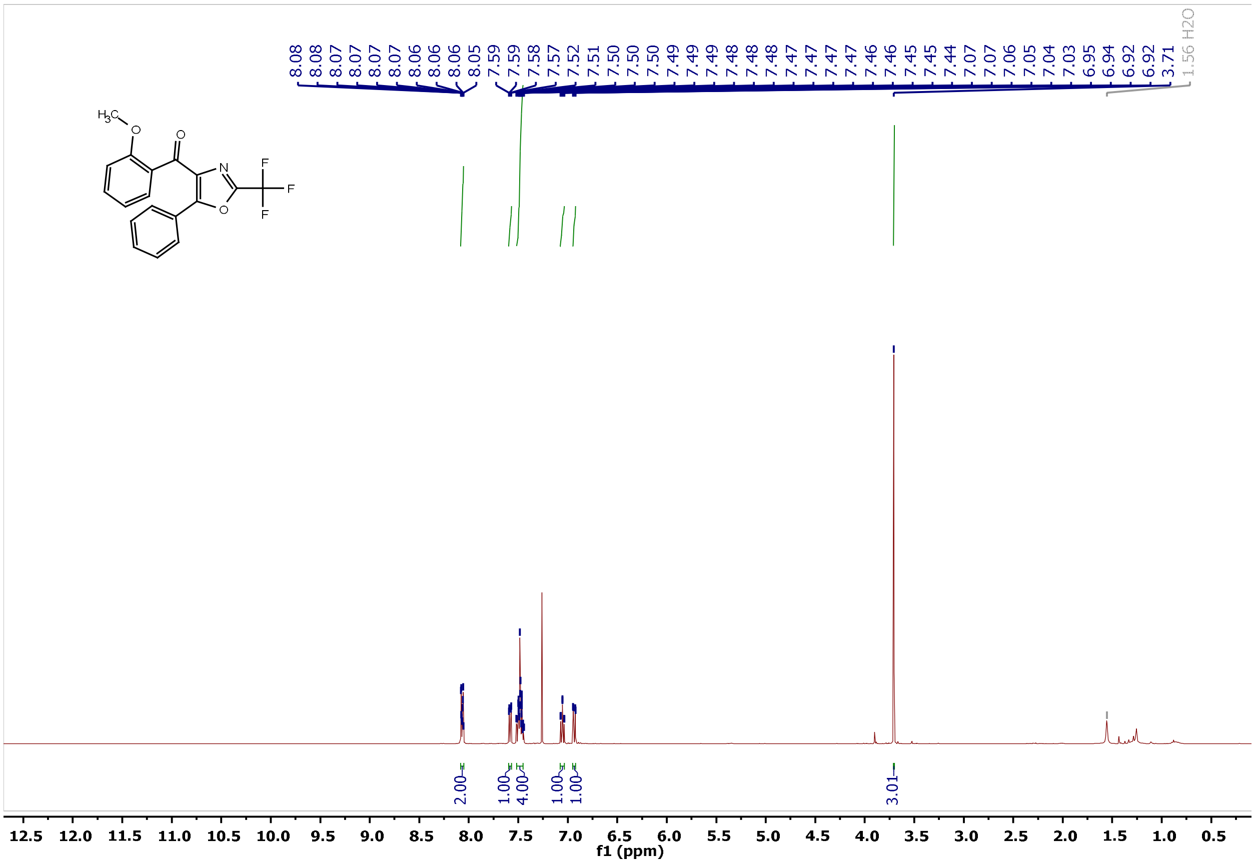

Figure S151. ^{13}C NMR spectrum of **6f** ($CDCl_3$, 101 MHz)

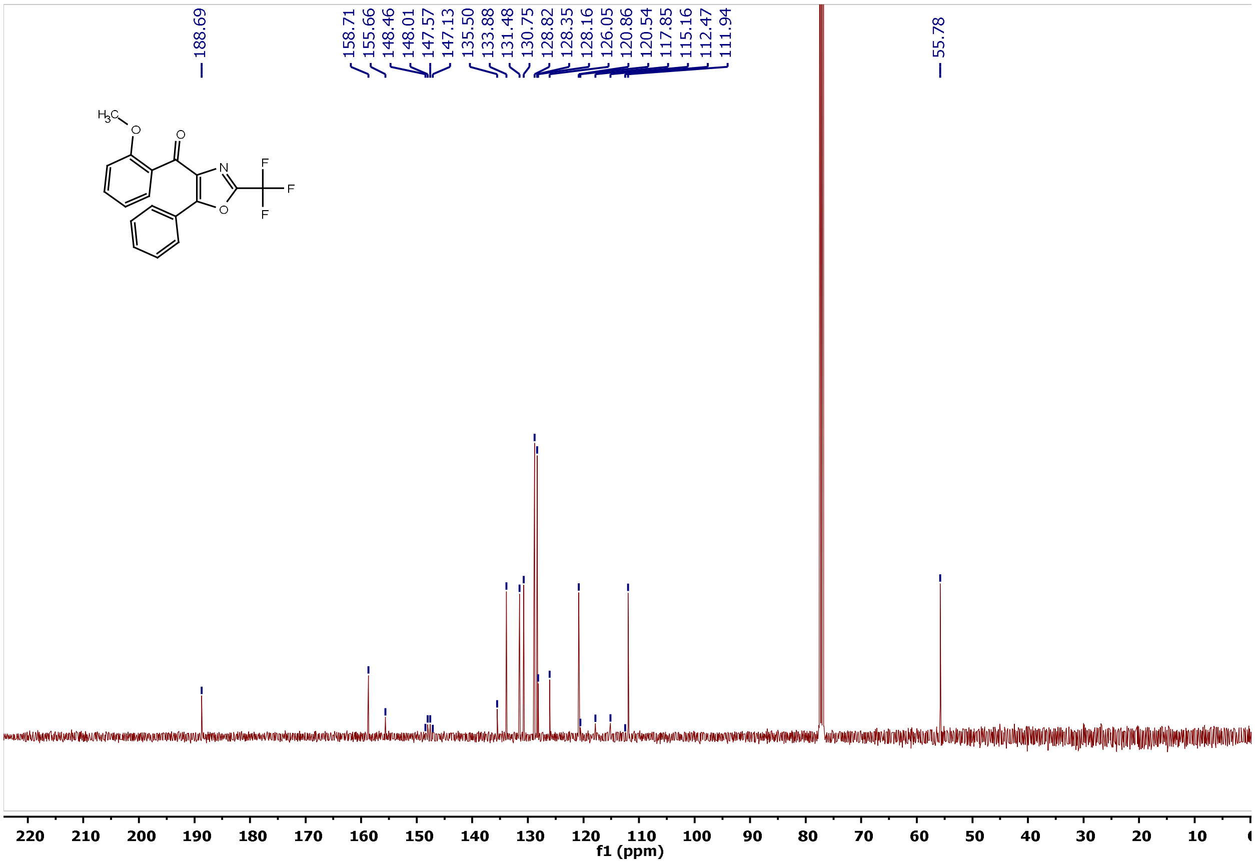

Figure S152. ^{19}F NMR spectrum of **6f** ($CDCl_3$, 377 MHz)

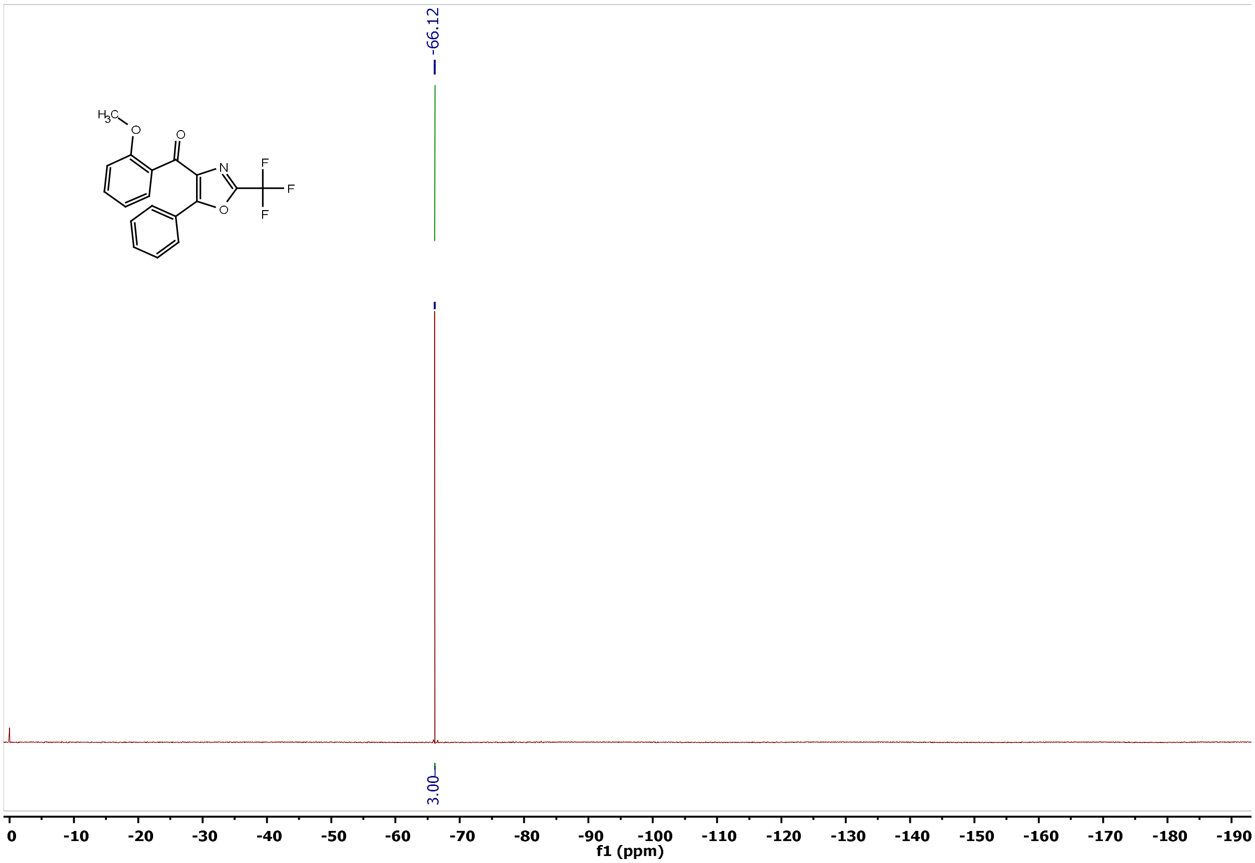

Figure S153. ^{1}H NMR spectrum of **6g** ($CDCl_3$, 401 MHz)

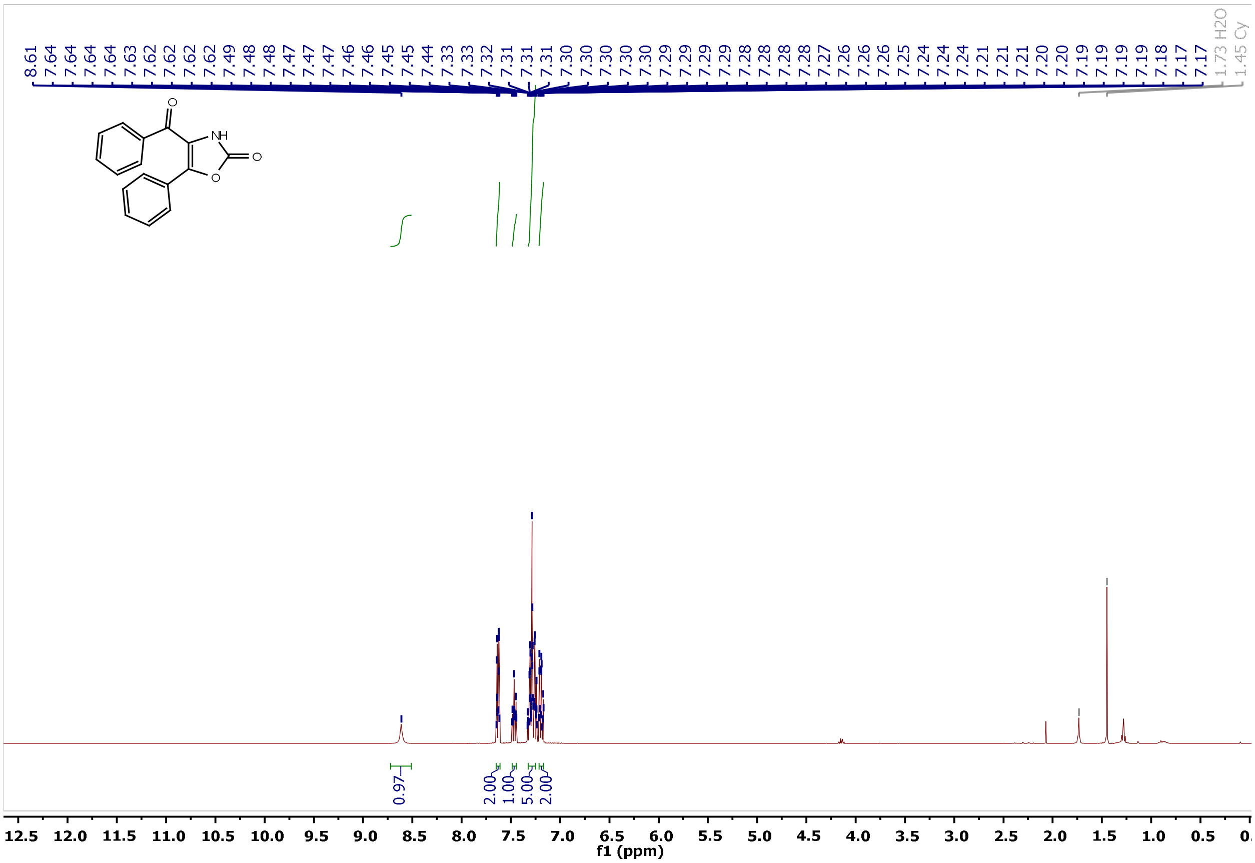

Figure S154. ^{13}C NMR spectrum of **6g** ($CDCl_3$, 101 MHz)

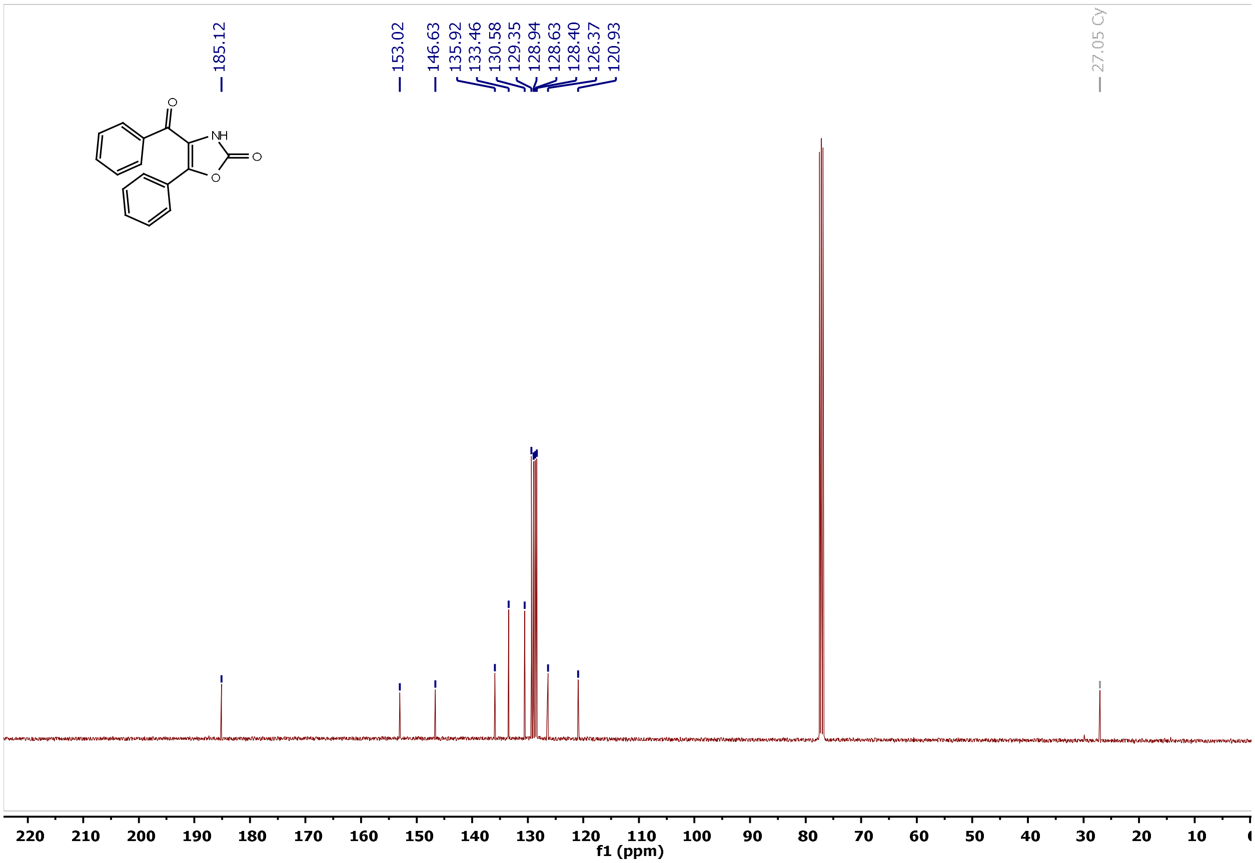

Figure S155. ^{1}H NMR spectrum of **7a** ($CDCl_3$, 401 MHz)

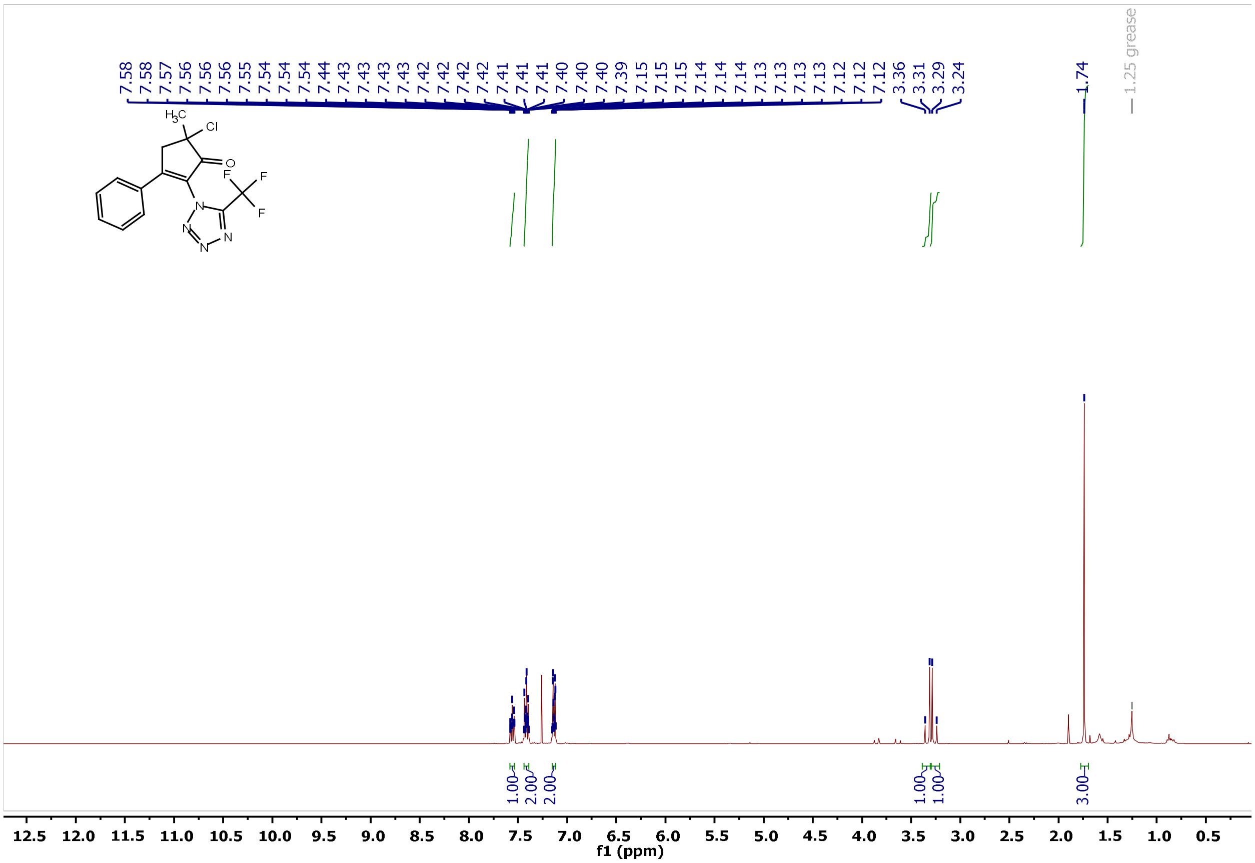

Figure S156. ^{13}C NMR spectrum of **7a** ($CDCl_3$, 101 MHz)

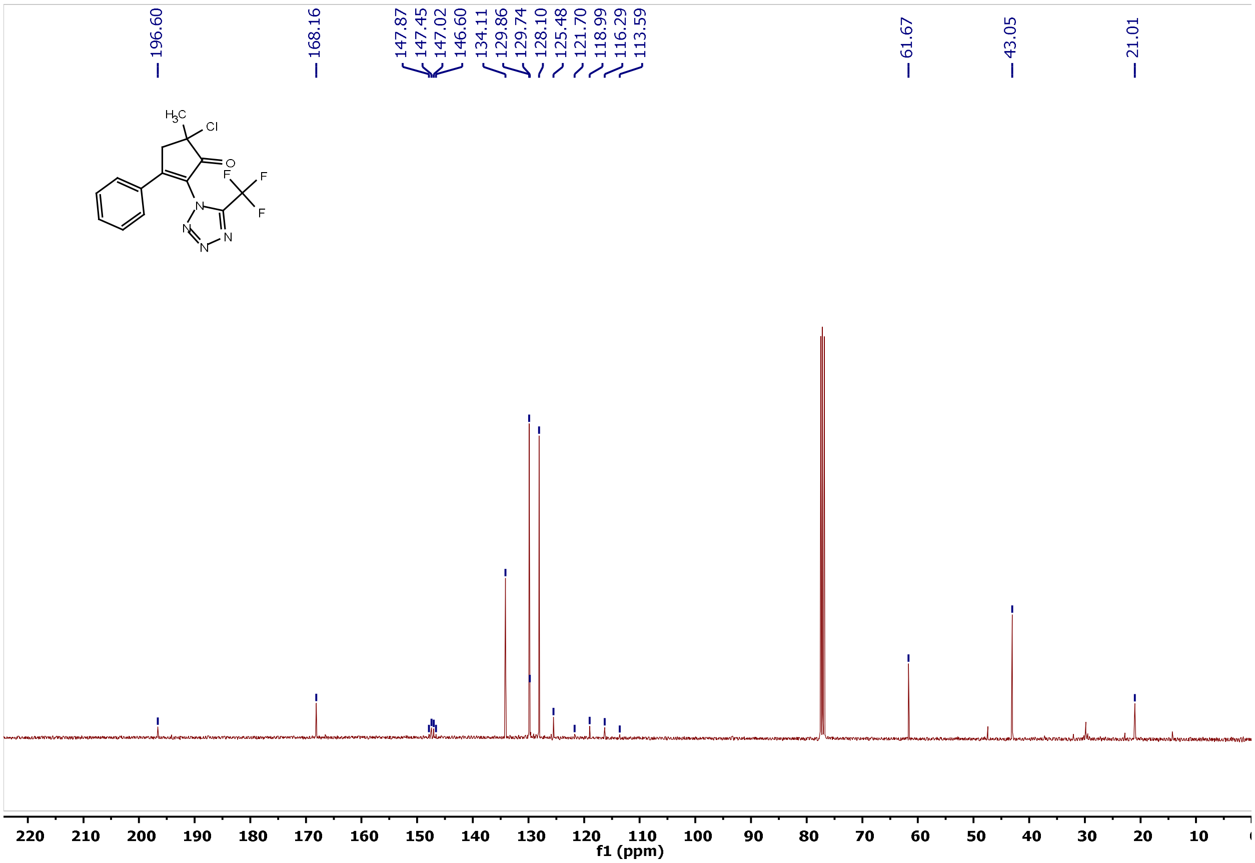

Figure S157. ^{19}F NMR spectrum of **7a** ($CDCl_3$, 377 MHz)

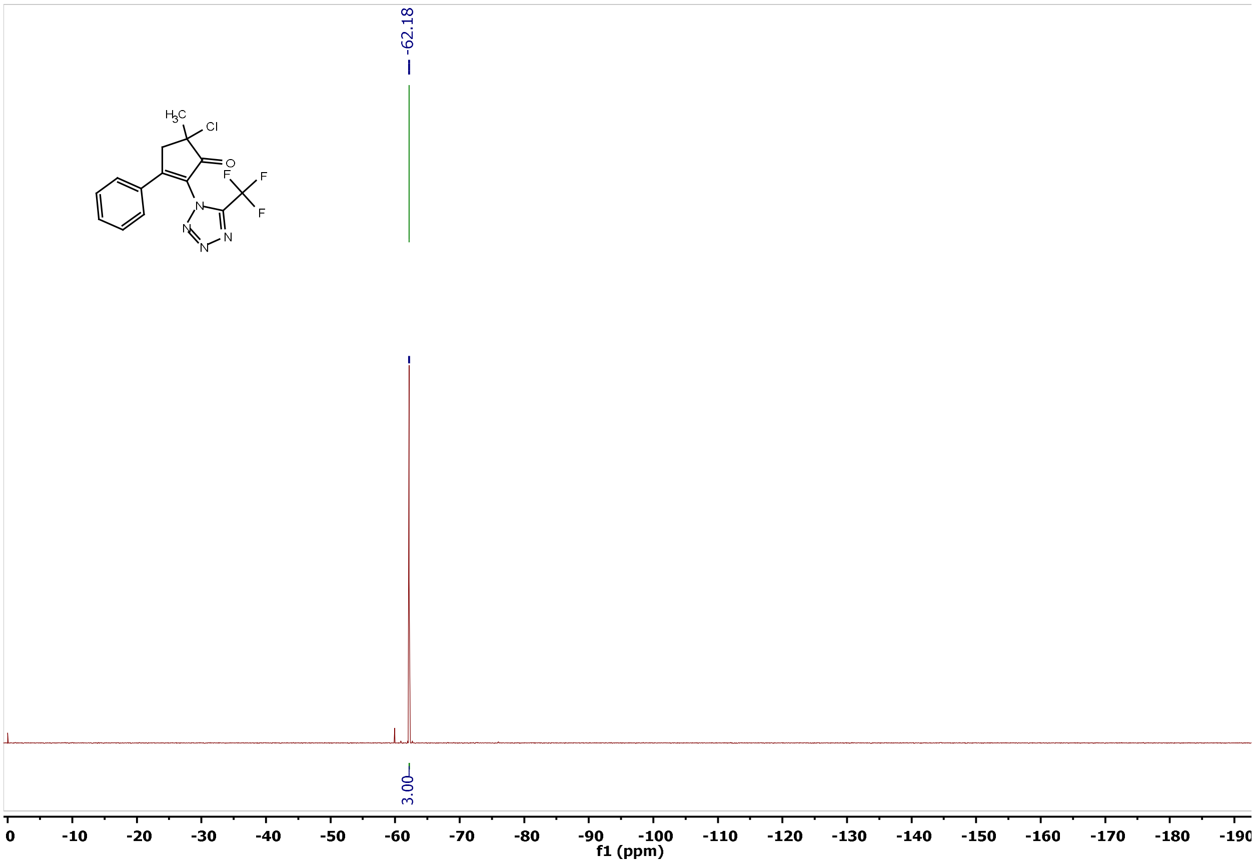

Figure S158. ^{1}H NMR spectrum of **7b** ($CDCl_3$, 401 MHz)

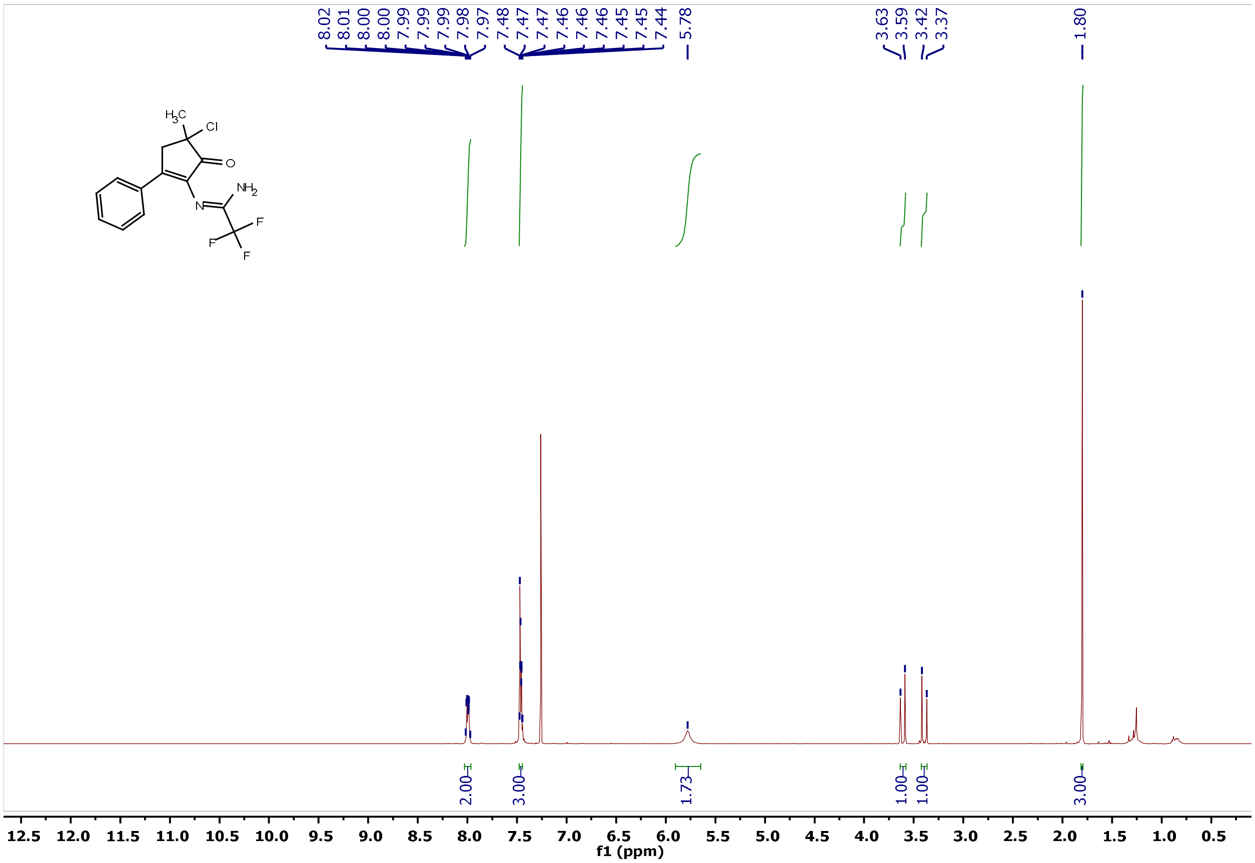

Figure S159. ^{13}C NMR spectrum of **7b** ($CDCl_3$, 101 MHz)

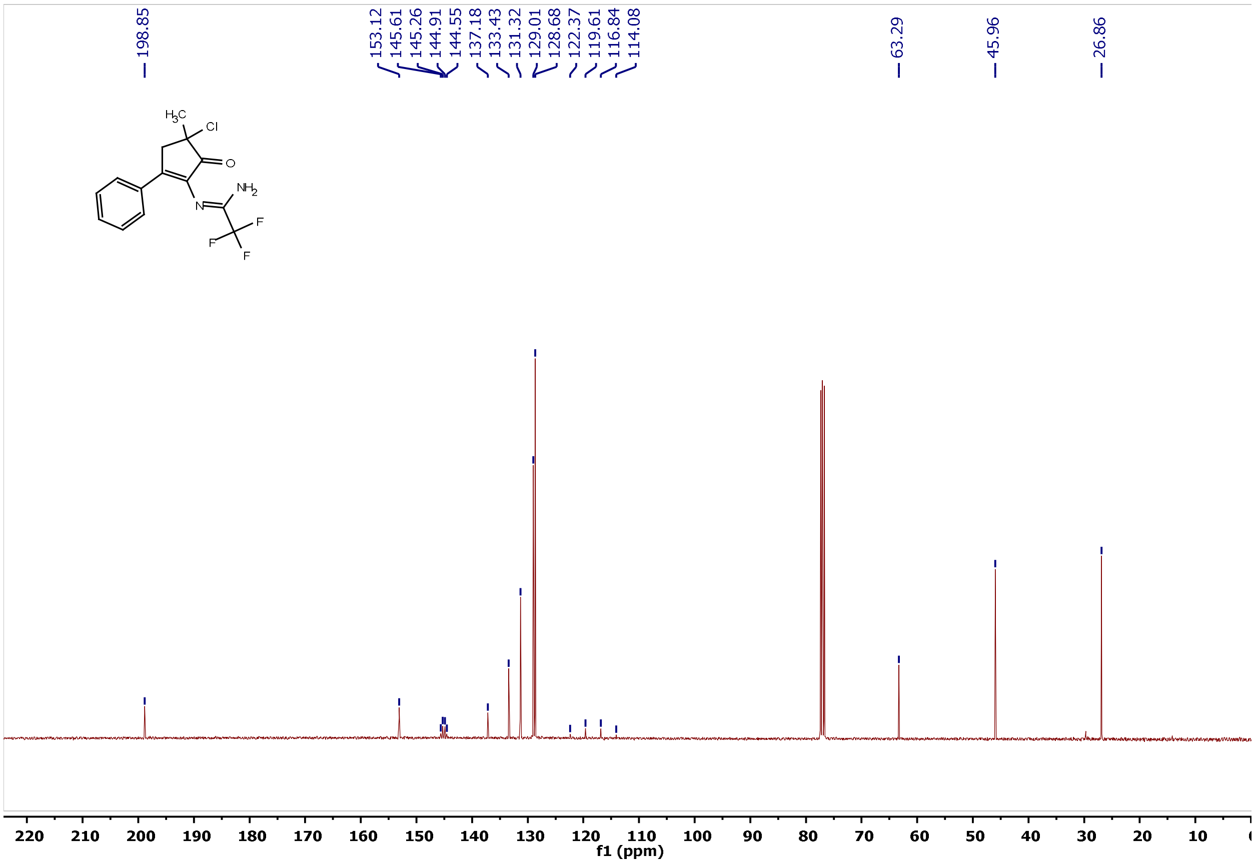

Figure S160. ^{19}F NMR spectrum of **7b** ($CDCl_3$, 377 MHz)

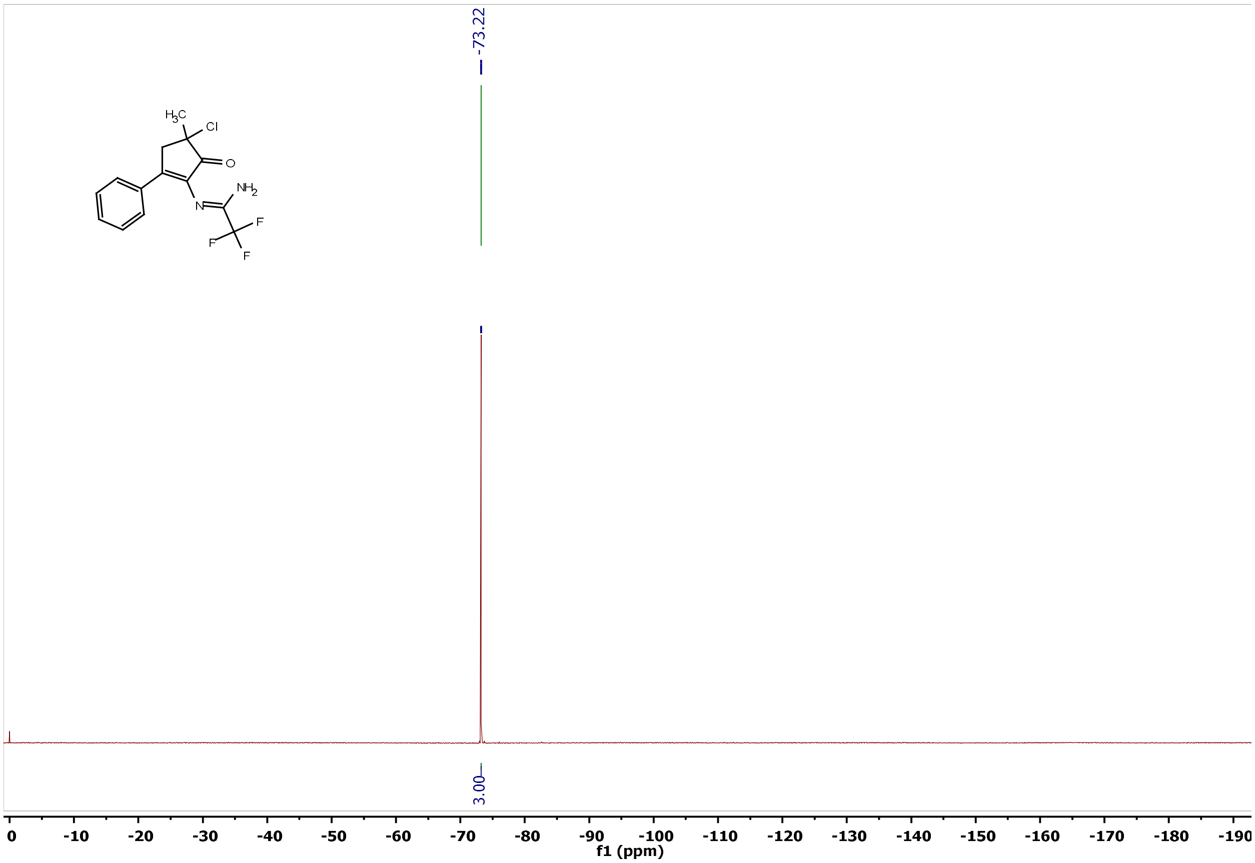

Figure S161. ^{1}H NMR spectrum of **7c** (d_6-Acetone, 400 MHz)

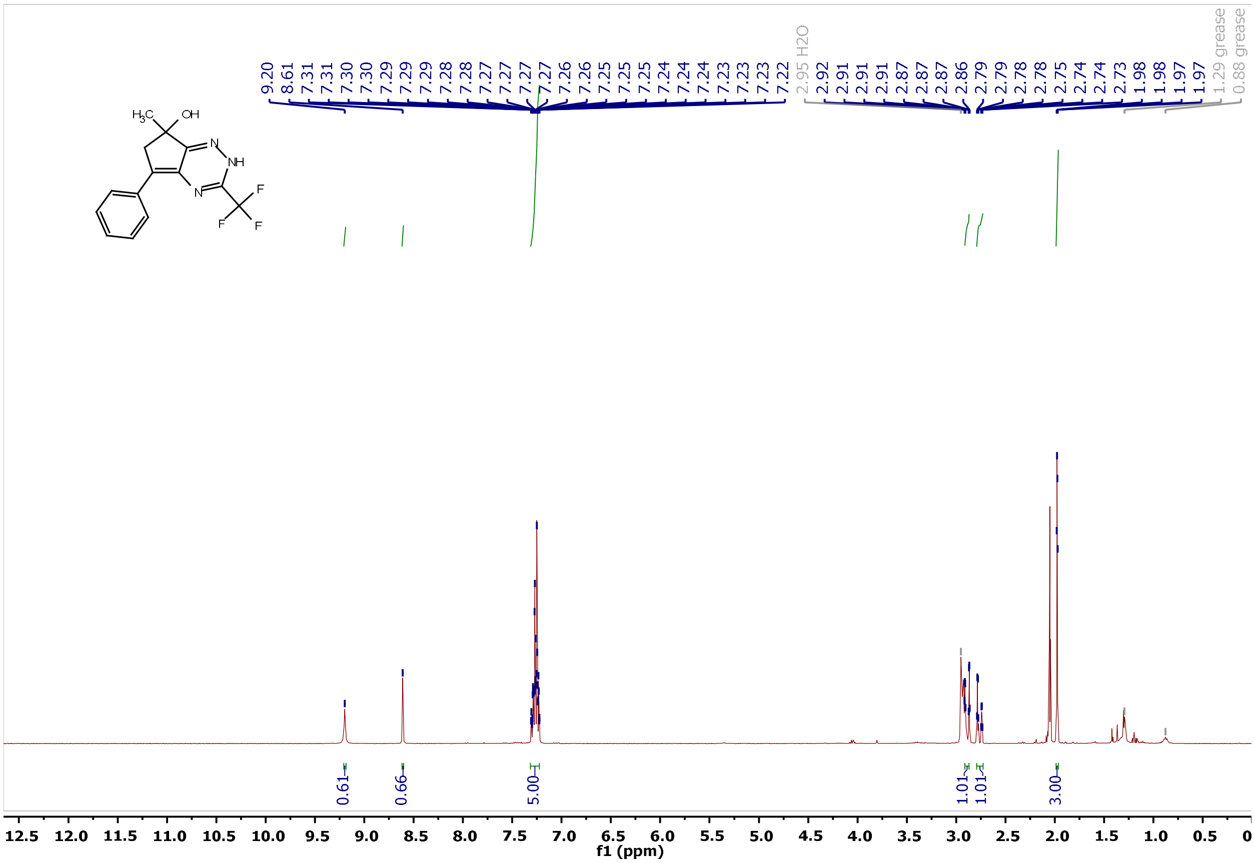

Figure S162. ^{13}C NMR spectrum of **7c** (d_6-Acetone, 400 MHz)

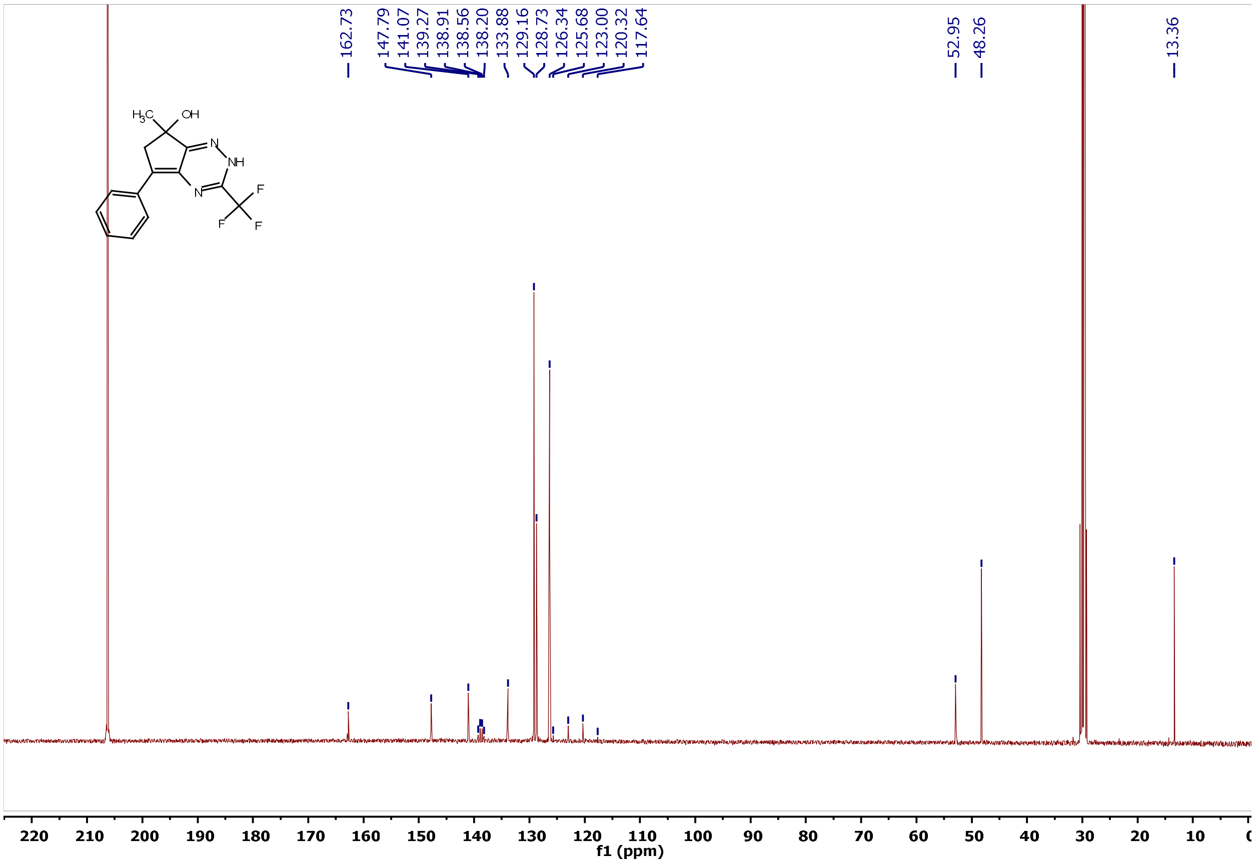

Figure S163. ^{19}F NMR spectrum of **7c** (d_6-Acetone, 376 MHz)

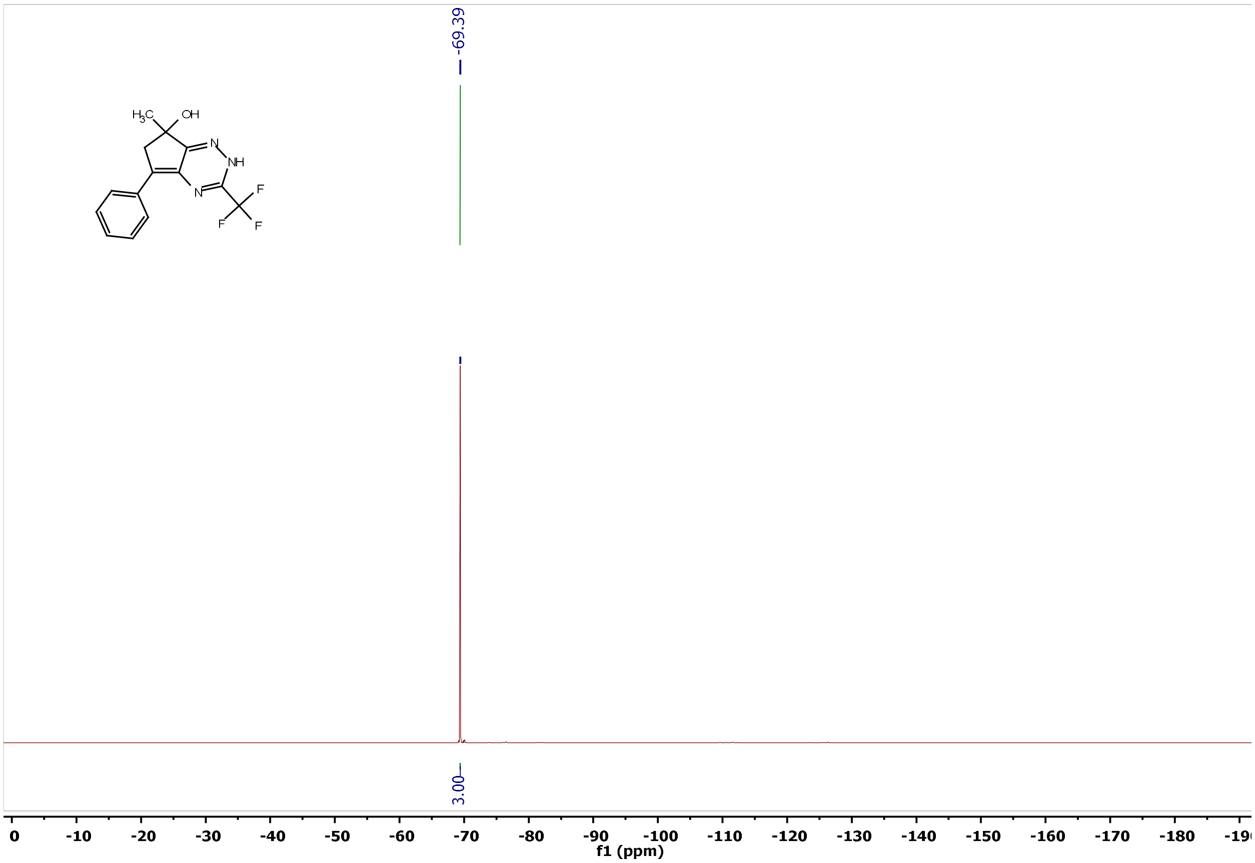

Supplement: RA-014-D4RA01707B-s001 [file RA-014-D4RA01707B-s001.pdf]
